# Design and Synthesis of (2-*oxo*-1,2-Dihydroquinolin-4-yl)-1,2,3-triazole Derivatives via Click Reaction: Potential Apoptotic Antiproliferative Agents

**DOI:** 10.3390/molecules26226798

**Published:** 2021-11-10

**Authors:** Essmat M. El-Sheref, Mohammed A. I. Elbastawesy, Alan B. Brown, Ahmed M. Shawky, Hesham A. M. Gomaa, Stefan Bräse, Bahaa G. M. Youssif

**Affiliations:** 1 Chemistry Department, Faculty of Science, Minia University, El-Minia 61519, Egypt; 2 Department of Pharmaceutical Organic Chemistry, Faculty of Pharmacy, Al-Azhar University, Assiut 71524, Egypt; mohamedali.pharm.ast@azhar.edu.eg; 3 Program in Chemistry, Florida Institute of Technology, 150 W University Blvd, Melbourne, FL 32901, USA; abrown@fit.edu; 4 Science and Technology Unit (STU), Umm Al-Qura University, Makkah 21955, Saudi Arabia; amesmail@uqu.edu.sa; 5 Pharmacology Department, College of Pharmacy, Jouf University, Sakaka 72314, Saudi Arabia; hasoliman@ju.edu.sa; 6 Institute of Organic Chemistry, Karlsruhe Institute of Technology, 76131 Karlsruhe, Germany; 7 Institute of Biological and Chemical Systems (IBCS-FMS), Karlsruhe Institute of Technology, 76344 Eggenstein-Leopoldshafen, Germany; 8 Pharmaceutical Organic Chemistry Department, Faculty of Pharmacy, Assiut University, Assiut 71526, Egypt

**Keywords:** click, azido, quinolones, triazole, anti-proliferative, apoptosis

## Abstract

A mild and versatile method based on Cu-catalyzed [2+3] cycloaddition (Huisgen-Meldal-Sharpless reaction) was developed to tether 3,3’-((4-(prop-2-yn-1-yloxy)phenyl)methylene)*bis*(4-hydroxyquinolin-2(1*H*)-ones) with 4-azido-2-quinolones in good yields. This methodology allowed attaching three quinolone molecules via a triazole linker with the proposed mechanism. The products are interesting precursors for their anti-proliferative activity. Compound **8g** was the most active one, achieving IC_50_ = 1.2 ± 0.2 µM and 1.4 ± 0.2 µM against MCF-7 and Panc-1 cell lines, respectively. Moreover, cell cycle analysis of cells MCF-7 treated with **8g** showed cell cycle arrest at the G2/M phase (supported by Caspase-3,8,9, Cytochrome C, BAX, and Bcl-2 studies). Additionally, significant pro-apoptotic activity is indicated by annexin V-FITC staining.

## 1. Introduction

**Figure 1:**
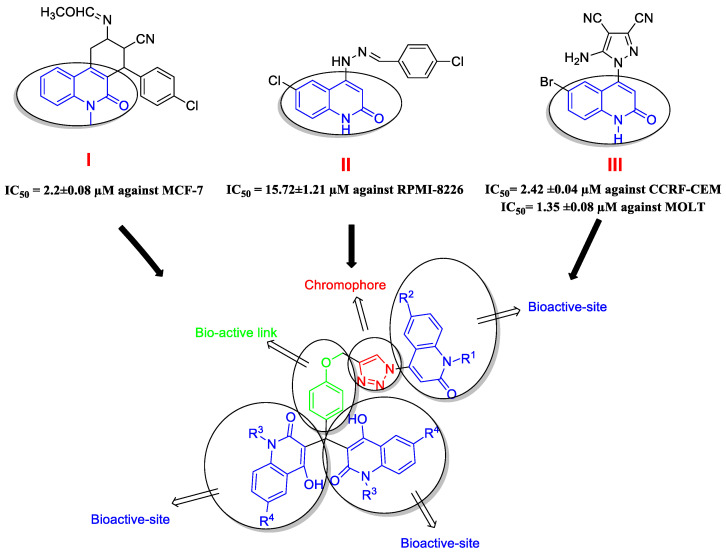
Bioactive sites and chromophore group in the designed compounds **8a**–**l**.

One of the most common heterocycles in drug development is the quinolone (oxo-quinoline) ring system [1,2]. Quinolone derivatives have been widely used in medicinal chemistry due to their unique structure, which exhibits a variety of pharmacological activities [3], especially antibacterial [4] and anti-cancer [5], with a promising role in the improvement of anti-cancer drug resistance [6]. Many quinolone derivatives exhibit excellent results in various operations, including growth inhibition via cell cycle arrest, apoptosis, and angiogenesis inhibition (Compounds **I**, **II**, and **III**, Figure 1) [7,8,9].

Triazole derivatives have a wide range of biological activities [10]. Furthermore, some drugs currently in clinical use are based on triazoles’ backbone, especially 1,2,3 triazole moiety [11,12]. Combining the quinolone scaffold with the triazole moiety will result in new leads with complementary activities and/or multiple pharmacological goals, and/or one component will counterbalance the side effects caused by the other [13,14]. Click chemistry is a simple method for assembling new molecular entities. The wide scope of CuAAC is firmly demonstrated by its use in different areas of life and material sciences fields, including drug discovery and bioconjugation [15,16].

Based on the preceding discussion and our efforts to produce quinoline derivatives as anti-cancer agents [17,18], as well as our work in the synthesis of heterocyclic molecules with potential biological activity [19], we used click chemistry to design and synthesize two bioactive sites in one molecule (Figure 1). The first is made up of three molecules of 4-hydroxy-2-quinolones, while the second is made up of a triazole moiety connected by a phenoxide linker. The newly synthesized compounds were tested for anti-proliferative efficacy against four different cancer cell lines. The most active ones were investigated further for their possible apoptotic activity utilizing extrinsic and intrinsic apoptotic indicators.

## 2. Results

### 2.1. Chemistry Section

**Scheme 1:**
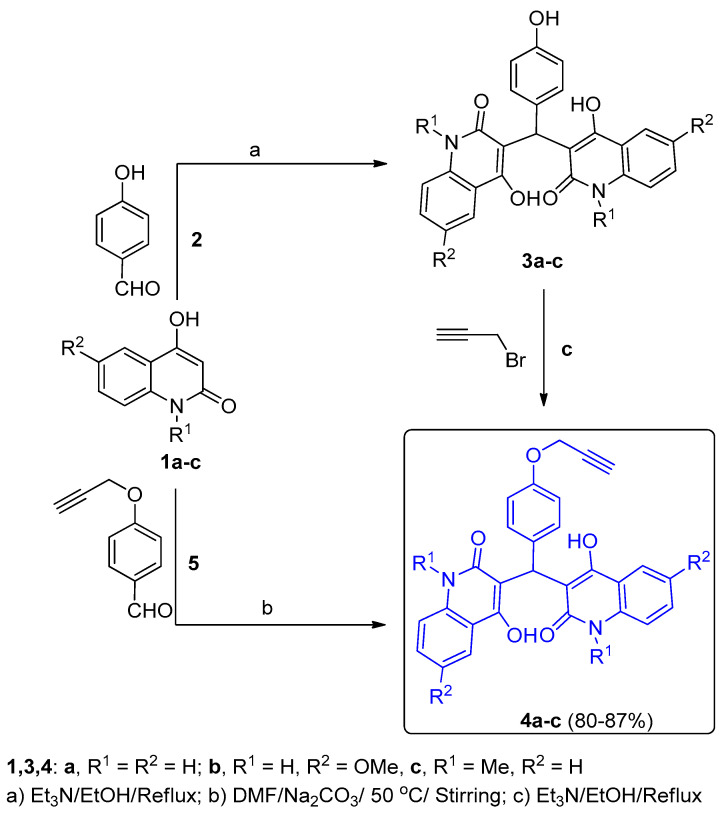
Synthesis of *bis*(4-hydroxy-quinolin-2(1*H*)-ones) **4a**–**c**.

**Scheme 2:**
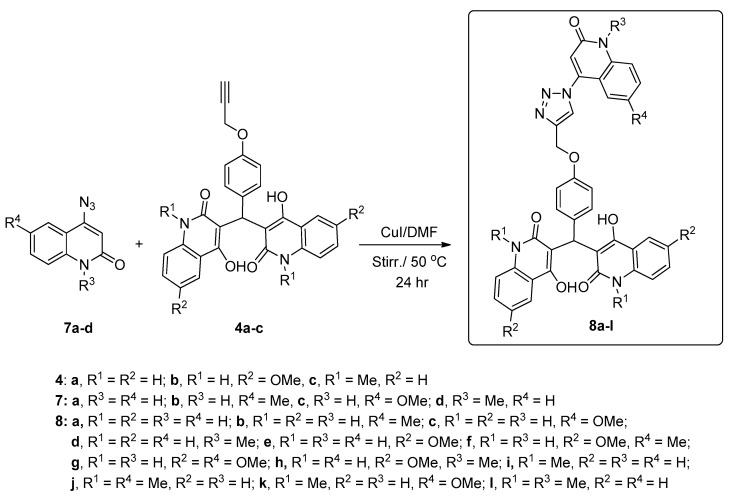
Synthesis of the new target compounds **8a**–**l**.

Here, in our present work, we synthesized a new various classes of quinolones/triazoles (Scheme 1 and Scheme 2) as 3,3’-((4-((1-(2-*oxo*-1,2-dihydroquinolin-4-yl)-1*H*-1,2,3-triazol-4-yl)methoxy)phenyl)methylene)*bis*(4-hydroxyquinolin-2(1*H*)-ones) **8a**–**l** from 4-azido-2-quinolinones **7a**–**d** and 3,3’-((4-(prop-2-yn-1-yloxy)phenyl)methylene)*bis*(4-hydroxy-quinolin-2(1*H*)-ones) **4a**–**c** by a Cu-catalyzed [3+2] cycloaddition, as shown in Scheme 2. We initially examined the synthesis of novel terminal alkynes **4a**–**c** via interaction between 4-hydroxy-quinoline-2(1*H*)-ones **1a**–**c** [20,21] and *p*-hydroxybenzaldehyde (**2**) (molar ratio 2:1) in absolute ethanol catalyzed with triethyl amine under refluxing conditions to yield 3,3’-((4-hydroxy-phenyl)methylene)*bis*(4-hydroxyquinolin-2(1*H*)-ones) **3a**–**c**. In DMF, the resulting phenol compound interacts with propargyl bromide to produce our terminal alkynes **4a**–**c** in good yield.

4-hydroxy-2-quinolinones **1a**–**c**, on the other hand, reacted with 4-(prop-2-yn-1-yloxy)benzaldehyde (**5**) in a molar ratio (2:1) to generate our desired terminal alkynes **4a**–**c**, as indicated in Scheme 1.

**Figure 2:**
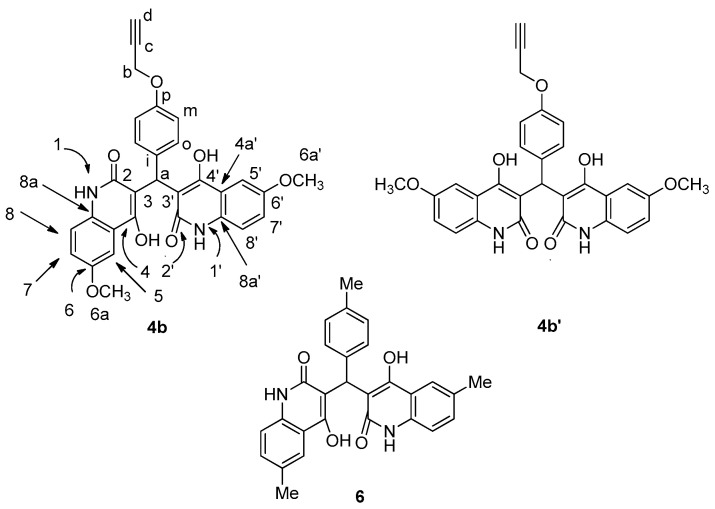
The structure of compound **4b**, its tautomeric structure **4b’**, and analog compounds **6**.

To confirm the structures of the obtained products 3,3’-(4-(prop-2-yn-1-yloxy)phenyl)methylene)-*bis*(4-hydroxy-quinolin-2(1*H*)-ones) **4a**–**c**, the elemental analysis declares that the acquired molecular formula for **4a**–**c** is generated from two molecules of compounds **1a**–**c** and one molecule of aldehyde **5** with the elimination of H_2_O molecule or one molecule of **3a**–**c** and one molecule of propargyl bromide with HBr molecule elimination. We discuss here the assignment of compound **4b,** which was expected to be 3,3’-((4-(prop-2-yn-1-yloxy)phenyl)methylene)*bis*-(4-hydroxy-6-methoxyquinolin-2(1*H*)-one) as an example for the new terminal alkynes **4a**–**c** (Figure 2).

The ^1^H NMR spectrum showed a characteristic four broad singlet signals at δ_H_ = 13.39, 12.90, 12.19, and 12.08 ppm, integrating four D_2_O exchangeable protons assigned as OH-4/4’, OH-4’/4, NH-1/1’, and NH-1’/1, respectively. Additionally, a singlet at δ_H_ = 6.14 ppm (1H) corresponded to q-CH-(H-a), which further confirmed from ^13^C NMR with a characteristic singlet at δ_C_ = 34.69 ppm (Table 1). The two methoxy groups with six protons exhibit a singlet at δ_H_ = 3.77 ppm, which further confirmed from ^13^C NMR with a characteristic singlet at δ_C_ = 55.41 ppm (C-6a, 6a’). Furthermore, the 1,4-disubstituted system was observed for the phenyl ring as doublet–doublet at δ_H_ = 7.02 (d, *J* = 8.5 Hz; 2H, H-o) and δ_H_ = 6.89 (d, *J* = 8.8 Hz; 2H, H-m), and both of them give ^1^H-^1^H-COSY correlation with each other and HMBC correlation with H-a and H-o (Table 1). In its ^13^C NMR spectra, C-8/8’ also gives HMBC correlation with a 2H doublet at δ_H_ 7.26 ppm, assigned as H-6/6’; its attached carbon appears at δ_C_ = 154.80 ppm, grounds as C-2,2’,4,4’. The 6H methyl singlet at δ_H_ = 3.77 must be H-6a/6a’ and gives HSQC correlation to carbon at δ_C_ = 55.41 ppm. C-a gives HMBC correlation to a 1H singlet at δ_H_ = 6.14, assigned as H-a; its attached carbon appears at δ_C_ = 34.69 ppm. Additionally, the four lines between δ_C_ = 166–160 give HMBC correlation with H-a and are assigned on chemical shift. Additionally, compound **4b** has a tautomeric form **4b’** in which the two quinolinone rings are in the same direction. However, from ^1^H NMR spectra, it is clear that the two OH groups, the two NH groups, and H-5 and H-5’ are not equivalent: that means, the two quinolinone rings are spectroscopically non-equivalent because their rotation is hindered, so that the compound does not possess a σ-plane. The simplest rationale is that the tautomer populations of the two quinolinones rings differ (tautomeric equilibration being relatively fast). Additionally, the two quinolinone-NH, which were assigned as N-1/1’, give HSQC with a proton at δ_H_ 12.08 ppm, and the other N-1’/1 give HSQC with a proton at δ_H_ 12.19 ppm, which confirms that the two quinolone rings are not equivalent in comparison with the analogous compound **6** prepared previously [22]. Although the two quinolinone rings are stated to be spectroscopically non-equivalent, some of their signals co-resonate because their rotation is hindered, so that the compound does not possess a σ plane and must be assigned as **4b**, not **4b’**.

Secondly, 4-azido-2-quinolinones **7a**–**d** reacted with 3,3’-((4-(prop-2-yn-1-yloxy)phenyl)methylene)*bis*(4-hydroxyquinolin-2(1*H*)-ones) **4a**–**c** in DMF as a solvent in molar ration (1.2:1) and catalyzed by 10 mol% of CuI to give 3,3’-((4-((1-(2-*oxo*-1,2-dihydroquinolin-4-yl)-1*H*-1,2,3-triazol-4-yl)methoxy)phenyl)methylene)-*bis*(4-hydroxyquinolin-2(1*H*)-one) **8a**–**l** in an excellent yields (Scheme 2).

**Figure 3:**
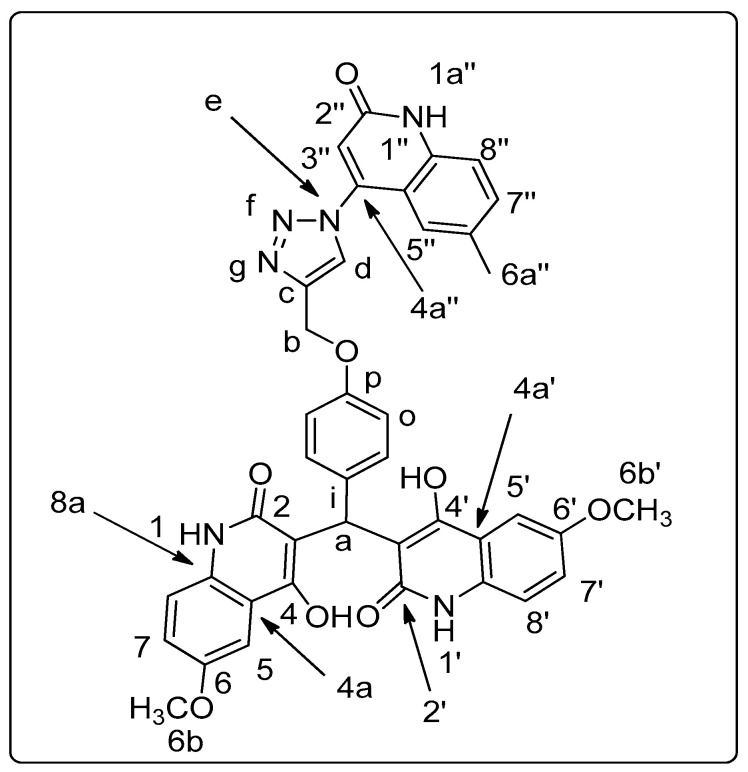
Structure of compound **8f**.

Different spectral data (^1^H NMR, ^13^C NMR, 2D NMR, and ^15^N NMR; see Supplementary Materials) addition to mass spectrometry, as well as elemental analyses, were used to confirm the structures of all new compounds **8a**–**l**. For example, compound **8f** was assigned as 3,3’-((4-((1-(6-methyl-2-*oxo*-1,2-dihydroquinolin-4-yl)-1*H*-1,2,3-triazol-4-yl)methoxy)phenyl)methylene)-*bis*(4-hydroxy-6-methoxyquinolin-2(1*H*)-one). It exhibited a molecular formula of C_40_H_32_N_6_O_8_, representing a product from one molecule of **4b** and one molecule of **7b** without elimination. Compound **8f** was further confirmed by mass spectrometry with *m*/*z* = 724 and elemental analysis (Figure 3), which confirm that compound **8f** is resulting from the reaction of compound **4b** with 4-azido-6-methyl-2-quinolinone **7b** in molar ratio (1:1) without elimination.

The 6-methyl-4-triazolo-2-quinolinone substructure (including the triazole ring) is assigned by analogy with compound **8b**. In both compounds, the two OH groups, the two NH groups, and H-5 and H-5’ are not equivalent: the simplest rationale is that the tautomer populations of the two quinolinone rings differ (tautomeric equilibration being relatively fast). Consistent with this idea, N-1 and N-1’, C-2 and C-2’, C-3 and C-3’, and C-4 and C-4’ are also non-equivalent in both samples; C-4a and C-4a’ are non-equivalent in compound **4b**, and C-8a and C-8a’ are non-equivalent in compound **8f** (Table 2, Exp. Section). The ^1^H-NMR spectrum of **8f** showed five protons that appeared as broad signals at δ_H_ 13.41, 12.91, 12.20, and 12.09 ppm for the quinolinone rings as two hydroxyl groups OH-4/4’, OH-4’/4, quinolinone-N-1/1’,1″ and quinolinone-N-1’/1, respectively. These two protons were exactly assigned by analogy with **4b** (Figure 2) and 2D NMR (Table 2). Additionally, there were five doublets at δ_H_ 7.47 (d, *J* = 8.3 Hz; H-7″), 7.40 (d, *J* = 9.2 Hz; 2H, H-8,8’), and 7.38 (d, *J* = 8.4 Hz; 2H, H-5/5’, 8″), in addition to a 1,4-disubstituted doublet-doublet at δ_H_ 7.05 (d, *J* = 8.2, Hz; 2H, H-o) and 7.01 (d, *J* = 8.2 Hz; 2H, H-m), and four singlets at δ_H_ 7.22, 6.81, 5.27, 3.82, and 2.29 ppm, which were assigned as H-5’’, H-a, H-3″, H-a, H-b, H-6b, 6b’, and H-6a″, respectively. The ^13^C NMR spectrum for **8f** showed that four signals appeared at δ_C_ 165.85, 164.13, 161.84, and 160.84 ppm, assigned as C-2,2’, C-4,4’, and C-2″,4″, respectively. Additionally, the signal at δ_C_ 60.85, which gives HSQC correlation with the proton at δ_H_ 5.27, was assigned as C-b (-O-CH_2_), in addition to two signals at 55.40 and 20.50 ppm, which was assigned for two methoxy groups (C-6b, 6b’) and methyl group (C-6a″), respectively. Furthermore, we see four types of nitrogen in the ^15^N NMR spectrum; one of them appears at δ_N_ 247.4, which indicates an sp^3^ nitrogen; it was assigned as N-e and gave HMBC correlation with a proton at δ_H_ 8.84 and 6.81 (H-d, H-3″) but gives no HSQC correlation. The second nitrogen atom resonated at δ_N_ 151.6 ppm, which assigned as N-1″ and also gives HMBC correlation with a proton at δ_H_ 7.38 and 6.81 (H-5/5’, 8″ and H-3″). The last two nitrogen at δ_N_ 144.2 and 142.8 ppm., which they assigned as N-1’/1 and N-1/1’, and they give HSQC correlation with the attached protons at δ_H_ 12.09 and 12.20, respectively.

### 2.2. Pharmacological Assays

#### 2.2.1. Cell Viability Assay

A human mammary gland epithelial cell line was used in the cell viability assay (MCF-10A). MCF-10A cells were incubated with compounds **8a**–**l** for four days, and cell viability was determined using an MTT assay [23,24]. There were no cytotoxic effects in any of the compounds tested, and most of the compounds tested had more than 84% viability at 50 µM; Table 3 shows the results.

#### 2.2.2. Cytotoxic Activity and Evaluation of IC_50_

Tested **8a**–**l** compounds were assessed for their anti-proliferative activity against four human cancer cell lines, including the pancreatic cancer cell line (Panc-1), breast cancer cell line (MCF-7), colon cancer cell line (HT-29), and epithelial cancer cell line (A-549) using propidium iodide fluorescence assay [25,26] and the reference doxorubicin. Results (IC_50_) are illustrated in Table 3.

From the results, it is clear that the electronic effect of the substitution (**R^2^** and/or **R^4^**) on the phenyl groups of quinoline moieties is important for the antiproliferative activity. Compounds **8c**, **8e**, **8f**, and **8g** (**R^2^** and/or **R^4^** = OCH_3_) were the most active compounds against the tested four cell lines with average IC_50_ in the range of 1.575 to 3.850 μM. Compound **8g** (**R^1^** = **R^3^** = H, **R^2^** = **R^4^** = OCH_3_) showed the utmost antiproliferative activity with average IC_50_ = 1.575 μM compared to the reference doxorubicin with average IC_50_ = 1.136 μM. Compounds **8e** (**R^1^** = **R^3^** = **R^4^** = H, **R^2^** = OCH_3_, average IC_50_ = 1.875) and **8c** (**R^1^** = **R^2^** = **R^3^** = H, **R^4^** = OCH_3_, average IC_50_ = 3.850) showed higher IC_50_ (less potent) than **8g**. Replacement of OCH_3_ group either by H or CH_3_ resulted in a decrease in the antiproliferative activity. For example, compounds **8a** (**R^1^** = **R^2^** = **R^3^** = H, **R^4^** = H, average IC_50_ = 5.575 μM) and **8b** (**R^1^** = **R^2^** = **R^3^** = H, **R^4^** = CH_3_, average IC_50_ = 7.40 μM) showed a 1.5-fold and 2-fold decrease in activity relative to **8c**, respectively.

Another significant factor influencing the efficacy of these compounds is the position of substituent on the scaffold studied, which had a major impact on its cytotoxic activity. Generally, triazoles **8a**–**c** and **8e**–**g** (**R^1^** and/or **R^3^** = H) showed superior anti-proliferative activity compared to their N-methyl counterparts **8d**, **8h**, **8i**, and **8j**–**l** ((**R^1^** and/or **R^3^** = CH_3_), Table 3. For example, the unsubstituted derivative **8a** (**R^1^** = **R^2^** = **R^3^** = **R^4^** = H) showed an average IC_50_ of 5.575 µM against the tested cell lines. Replacement of the NH group by N-CH_3_, **8l** (**R^1^** = **R^3^** = CH_3_, **R^2^** = **R^4^** = H, average IC_50_ = 24.725 μM) resulted in at least 4-fold reduction of the average IC_50_ value. In addition, the average IC_50_ values of **8i**–**k** (**R^1^** and/or **R^3^** = CH_3_) were the lowest among the compounds tested, indicating the significance of the NH group for anti-proliferative action.

#### 2.2.3. Apoptosis Assay

To discover the pro-apoptotic capability of our target compounds, the utmost active compounds **8e**, **8f**, and **8g** were tested for their potential ability to induce apoptosis in a MCF-7 breast cancer cell line.

##### Activation of Proteolytic Caspases Cascade

**Figure 4:**
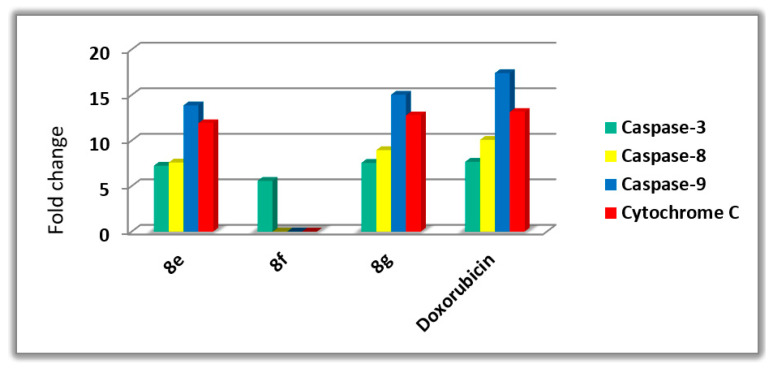
Effects of compounds **8e**, **8f**, and **8g**, and doxorubicin on active Caspases 3, 8, 9, and Cytochrome C in MCF-7 breast cancer cell line.

Caspases play an important role in initiating and completing the apoptotic process [27]. Caspase 3 is an essential caspase that cleaves multiple proteins in cells, resulting in apoptotic cell death [28]. The effects of compounds **8e**, **8f**, and **8g** on Caspase 3 were investigated and compared to that of doxorubicin. The findings revealed that when compared to control cells, the studied compounds increased the level of active Caspase 3 by 5.5–7.5-fold and that **8e**, **8f**, and **8g** had unprecedented control over Caspase 3 protein level expression (475.20 ± 4.27, and 365.60 ± 3.20, and 489.2 ± 4.13 pg/mL, respectively) compared to doxorubicin (503.2 ± 4.22 pg/mL). Compared to control untreated cells, the most active compound **8g** showed a 7.45-fold increase in active Caspase 3 levels, Figure 4.

Compounds **8e** and **8g** were also tested for their effects on Caspases 8 and 9. The results showed that compound **8g** raises Caspase 8 and 9 levels by 8.94- and 15.03-fold, respectively. When compared to control cells, compound **8e** showed a 7.58- and 13.86-fold increase in Caspase 8 and 9, respectively, indicating activation of both the intrinsic and extrinsic pathways, with the intrinsic pathway having a stronger effect since Caspase 9 levels were higher [29].

##### Cytochrome C Assay

The amount of cytochrome C in a cell plays an important role in caspase activation and the initiation of the intrinsic apoptosis pathway [29,30]. Table 4 shows the effects of testing triazole derivatives **8e** and **8g** as cytochrome C activators in the MCF-7 human breast cancer cell line. **8e** and **8g** resulted in around 11.90- and 12.76-fold higher levels of cytochrome C expression than untreated control cells. The findings add to the proof that apoptosis can be attributed to cytochrome C overexpression and activation of the intrinsic apoptotic pathway triggered by the tested compounds.

##### Bax and Bcl-2 Levels Assay

Using doxorubicin as a control, the most active caspase activators **8e** and **8g** were further investigated for their impact on Bax and Bcl-2 levels in the MCF-7 breast cancer cell line [31,32]. Table 5 shows that **8e** and **8g** elicited a significant increase in Bax levels as compared to doxorubicin. **8g** demonstrated a comparable induction of Bax (264.90 pg/mL) relative to doxorubicin (276 pg/mL), 32-fold higher than the control untreated breast cancer cell, followed by compound **8e** (247.65 pg/mL and 30-fold change). Finally, compound **8g** reduced Bcl-2 protein levels in MCF-7 cells to 1.085 ng/mL, followed by compound **8e** (1.278 ng/mL) and doxorubicin (0.98 ng/mL).

##### Flow Cytometric Cell Cycle Analysis

**Figure 5:**
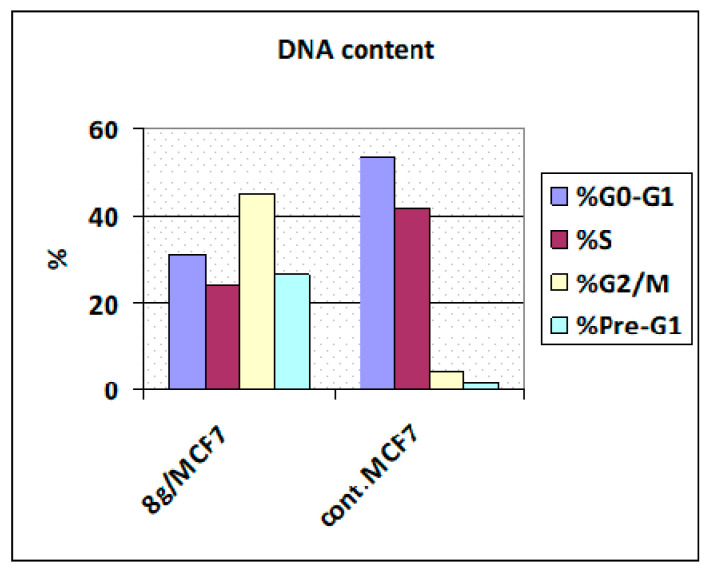
Cell cycle analysis in MCF-7 cell line treated with compound **8g**.

**Figure 6:**
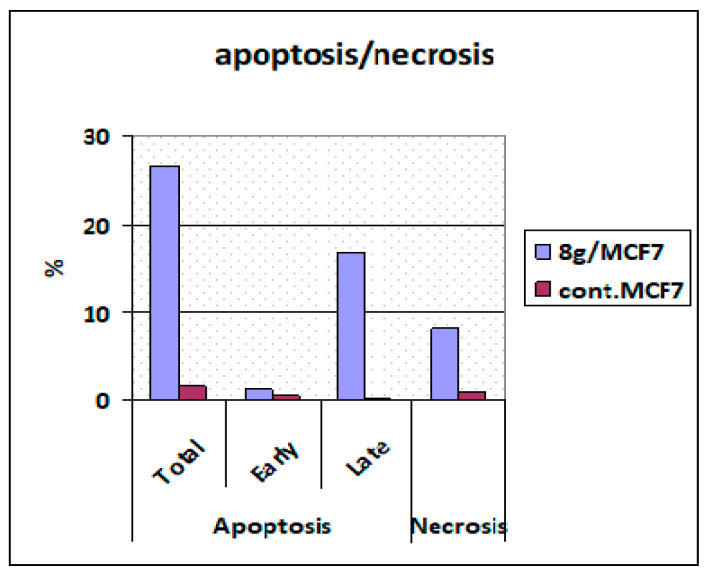
Percentage of apoptosis and necrosis for compound **8g** in MCF-7 cell line.

**Figure 7:**
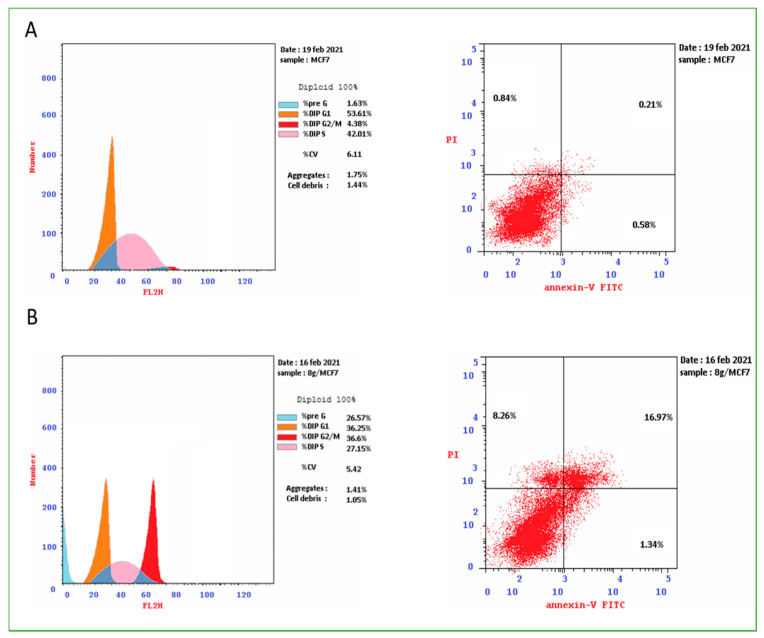
Cell cycle analysis and Apoptosis induction analysis using Annexin V/PI of control untreated MCF-7cell line (**A**) and **8g** (**B**).

Following treatment with the most active compound, **8g**, a cell cycle analysis was performed on the MCF-7 cancer cell line [33,34]. Figure 5 shows that the percentage of MCF-7 cell line cells in the G0/G1 phase was 53.61% in control and 36.25% after treatment with compound **8g**, whereas the percentage of cells in the S phase was reduced with compound **8g** (27.15%) compared to the control (42.01%). Compared to the control, the percentage of MCF-7 cell line in the G2/M process increased to 36.6% after treatment with **8g** (4.38%). Furthermore, the percentage of apoptotic cells in the Pre-G1 process increased from 1.63% in control untreated MCF-7 cells to 26.57% and 22.17% in cells treated with **8g** and doxorubicin, respectively (Figure 6 and Figure 7). According to the findings, **8g** primarily displayed cell cycle arrest at the G2/M phases. Furthermore, the investigated compound is anti-proliferative rather than cytotoxic, resulting in programmed cell death and cell cycle arrest.

## 3. Conclusions

A series of novel conjugated heterocycles containing three quinolinone moieties could be synthesized straightforwardly. The propargylated core of the phenoxide linker with two molecules of quinolinone could be equipped with another tethered molecule by reacting with 4-azido-2-quinolones via a Meldal–Sharpless click reaction. The target products 3,3’-((4-(prop-2-yn-1-yloxy)phenyl)methylene)*bis*(4-hydroxy-quinolin-2(1*H*)-ones) were obtained in good to very good yields and were characterized by different spectroscopic and elemental methods including ^1^H NMR, ^13^C NMR, 2D NMR, MS, and elemental microanalyses.

The antiproliferative activity of the new compounds was tested against a panel of cancer cell lines. Compound **8g** demonstrated the highest antiproliferative activity with an average IC_50_ = 1.575 M compared to doxorubicin (IC_50_ = 1.136 M). The results show that the electronic effect of the substitution, as well as its position on the scaffold studied, have a significant impact on the activity of these compounds.

The most active compounds were further investigated for caspase, cytochrome C activation, Bax activation, and Bcl-2 downregulation compared to doxorubicin. Compounds **8e** and **8g** increased the levels of active Caspase 3, 8, and 9, indicating that both the intrinsic and extrinsic pathways were activated, with the intrinsic pathway having a stronger effect due to higher Caspase 9 levels. Compound **8g** exhibited cell cycle arrest at the G2/M phase with a significant pro-apoptotic activity.

## 4. Experimental

### 4.1. Chemistry

Melting points were determined on Stuart electrothermal melting point apparatus and were uncorrected. All reactions were monitored with thin-layer chromatography (TLC) on Merck alumina-backed TLC plates and visualized under UV light. NMR spectra were measured in DMSO-*d*_6_ on a Bruker AV-400 spectrometer (400 MHz for ^1^H, 100 MHz for ^13^C, and 40.54 MHz for ^15^N), at the Chemistry Department, Florida Institute of Technology, 150 W University Blvd, Melbourne, FL 32901, USA, and Chemistry Department, Faculty of Science, Cairo University, Cairo, Egypt. Chemical shifts are expressed in δ (ppm) versus internal Tetramethylsilane (TMS) = 0 ppm for ^1^H and ^13^C, and external liquid ammonia = 0 ppm for ^15^N. Coupling constants are stated in Hz. Correlations were established using ^1^H-^1^H COSY, ^1^H-^13^C, and ^1^H-^15^N HSQC and HMBC experiments. Chemical shifts (δ) are reported in parts per million (ppm) relative to Tetramethylsilane (TMS) as an internal standard, and the coupling constants (*J*) are reported in Hertz (Hz). Splitting patterns are denoted as follows: singlet (s), broad (b), doublet (d), multiplet (m), triplet (t), quartet (q), broad of singlet (bs), doublet of doublets (dd), doublet of triplets (dt), triplet of doublets (td), and doublet of a quartet (dq). Mass spectra were recorded on a Finnigan Fab 70 eV at Al-Azhar University, Egypt. Elemental analyses were carried out on a Perkin Elmer device at the Microanalytical Institute of Organic Chemistry, Karlsruhe Institute of Technology, Karlsruhe, Germany.

#### 4.1.1. Starting Materials

4-Hydroxy-quinoline-2(1*H*)-ones **1a**–**c** [20,21], 3,3’-((4-hydroxy-phenyl)methylene) *bis*(4-hydroxyquinolin-2(1*H*)-ones) **3a**–**c** [22], 4-(prop-2-yn-1-yloxy)benzaldehyde **5** [35], and 4-azido-2-quinolinones **7a**–**d** [36] were synthesized according to the literature. 4-hydroxybenzaldehyde (**5**) and propargyl bromide (Aldrich) were used as received.

#### 4.1.2. General Procedure for the Synthesis of Compounds **4a**–**c**

Method A: A mixture of 4-hydroxy-2-quinolinones **1a**–**c** (2 mmol), *p*-hydroxy-benzaldehyde (**2**) (1 mmol), and 0.5 mL of Et_3_N in 20 mL absolute ethanol was refluxed for 6 h. The resulting white precipitate was left to cool and then poured into ice-cold water to give **3a**–**c**. The precipitate collected by filtration was dried well and then reacted with propargyl bromide in DMF in a molar ratio (1:2), and the reaction mixture was monitored by TLC and then poured into about 200 g ice to give **4a**–**c**, which was collected by filtration.

Method B: A mixture of 4-hydroxy-2-quinolinones **1a**–**c** (2 mmol), 4-(prop-2-yn-1-yloxy)benzaldehyde (**5**) (1 mmol), and 0.5 mL of Et_3_N in 20 mL absolute ethanol was refluxed for 4h. The resulting white precipitate was left to cool and then filtered off to give **4a**–**c** in excellent yields.

*3,3’-((4-(Prop-2-yn-1-yloxy)phenyl)methylene)bis(4-hydroxyquinolin-2(1H)-one)* **4a**. Colorless powder (85%), m.p. 296–98 °C; ^1^H NMR (DMSO-*d*_6_): δ_H_ = 13.12 (bs; 1H, OH-4/4’), 12.67 (bs; 1H, OH-4’/4), 12.24 (bs; 1H, NH-1/1’), 12.21 (bs; 1H, NH-1’/1), 7.98 (bs; 1H, H-5/5’), 7.95 (bd, *J* = 6.6 Hz; 1H, H-5’/5), 7.61 (dd, *J* = 7.6, 7.6 Hz; 2H, H-7, 7’), 7.45 (d, *J* = 8.2 Hz; 2H, H-8, 8’), 7.30 (bs; 2H, H-6, 6’), 7.03 (d, *J* = 8.5 Hz; 2H, H-o), 6.88 (d, *J* = 8.7 Hz; 2H, H-m), 6.14 (s; 1H, H-a), 4.76 (d, *J* = 1.8 Hz; 2H, H-b), 3.55 ppm (bs; 1H, H-d), ^13^C NMR (DMSO-*d*_6_): δ_C_ = 165.90 (C-2/2’), 163.40 (C-2’/2), 161.00 (C-4, 4’), 155.35 (C-p), 137.00 (C-8a, 8a’), 131.10 (C-7, 7’), 130.16 (C-i), 127.31 (C-o), 123.15 (C-5, 5’), 122.52 (C-6, 6’), 115.87 (C-8, 8’), 114.36 (C-m), 110.80 (C-4a, 4a’), 79.41 (C-c), 78.01 (C-d), 55.34 (C-b), 34.50 ppm (C-a), ^15^N NMR (DMSO-*d*_6_): δ_N_ = 144.8 ppm (N-1, 1’). Anal. Calcd for C_28_H_20_N_2_O_5_: C, 72.41; H, 4.34; N, 6.03; Found: C, 72.59; H, 4.28; N, 6.18.

*3,3’-((4-(Prop-2-yn-1-yloxy)phenyl)methylene)bis(4-hydroxy-6-methoxy-quinolin-2(1H)-one)***4b**. Colorless powder (90%), m.p. 283–85 °C; NMR (DMSO-*d*_6_) (Table 1). Anal. Calcd for C_30_H_24_N_2_O_7_: C, 68.70; H, 4.61; N, 5.34; Found: C, 68.63; H, 4.55; N, 5.49.

*3,3’-((4-(Prop-2-yn-1-yloxy)phenyl)methylene)bis(4-hydroxy-1-methylquinolin-2(1H)-one)***4c**. Colorless powder (78%), m.p. 280–82 °C; ^1^H NMR (DMSO-*d*_6_): δ_H_ = 12.86 (bs, 1H; OH-4/4’), 12.52 (bs, 1H; OH-4’/4), 8.05 (bs, 2H; H-5, 5’), 7.71 (ddd, *J* = 7.3, 7.3, 0.9 Hz, 2H; H-7, 7’), 7.66 (d, *J* = 8.3 Hz, 2H; H-8, 8’), 7.37 (dd, *J* = 7.3, 7.2 Hz, 2H; H-6, 6’), 6.98 (d, *J* = 8.6 Hz, 2H; H-o), 6.85 (d, *J* = 8.8 Hz, 2H; H-m), 6.23 (s, 1H; H-a), 4.75 (d, *J* = 2.2 Hz, 2H; H-b), 3.72 (bs, 6H; H-1a, 1a’), 3.56 (t, *J* = 2.2 Hz, 1H; H-d), ^13^C NMR (DMSO-*d*_6_): δ_C_ = 164.75 (b; C-2, 2’), 160.58 (b; C-4, 4’), 155.32 (C-p), 138.10 (C-8a, 8a’), 131.54 (C-7, 7’), 130.12 (C-i), 127.28 (C-o), 123.60 (C-5, 5’), 122.70 (C-6, 6’), 116.90 (C-4a, 4a’), 115.29 (C-8, 8’), 114.35 (C-m), 110.12 (b; C-3, 3’), 79.42 (C-c), 78.02 (C-d), 55.31 (C-b), 35.98 (C-a), 30.12 ppm (b; C-1a, 1a’), ^15^N NMR (DMSO-*d*_6_): δ_N_ = 138.7 ppm (N-1, 1’). Anal. Calcd for C_30_H_24_N_2_O_5_: C, 73.16; H, 4.91; N, 5.69; Found: C, 73.25; H, 4.81; N, 5.55.

#### 4.1.3. General Procedure for the Synthesis of Compounds **8a**–**l**

A mixture of terminal alkynes **4a**–**c** (1.1 mmol) in 20 mL dimethylformamide (DMF) and CuI (10 mmol%) was stirred for 10 min at room temperature. Then, 4-azido compounds **7a**–**d** (1.0 mmol) were added to the mixture. The reaction mixture was allowed to stir at 50 °C for 24 h, and TLC was used to monitor the reaction. After completion, the mixture was diluted with 50 mL H_2_O. The precipitate was filtered off to give **8a**–**l** in excellent yields.

*3,3’-((4-((1-(2-oxo-1,2-dihydroquinolin-4-yl)-1H-1,2,3-triazol-4-yl)methoxy)phenyl)-methylene)bis(4-hydroxyquinolin-2(1H)-one)***8a**. Colorless powder, (75%), m.p. 280–82 °C; ^1^H NMR (DMSO-*d*_6_): δ_H_ = 13.18 (bs; 1H, OH-4/4’), 12.69 (bs; 1H, OH-4’/4), 12.29 (bs; 3H, NH-1, 1’, 1″), 8.86 (s; 1H, H-d), 7.96 (m; 2H, H-5, 5’), 7.63 (bd; 3H, H-7, 7’, 7″), 7.46 (bs; 4H, H-5″, 8, 8’, 8″), 7.26 (m; 2H, H-6, 6’), 7.24 (dd, *J* = 7.4, 7.4 Hz; 1H, H-6″), 7.05 (m; 2H, H-o), 6.86 (m; 2H, H-m), 6.16 (s; 1H, H-a), 5.27 ppm (s; 2H, H-b), ^13^C NMR (DMSO-*d*_6_): δ_C_ = 161.13, 160.77 (C-2, 2’, 2″, 4, 4’, 4″), 155.89 (C-p), 143.41, 143.25 (C-c), 139.18, 136.82 (C-8a, 8a’, 8a″), 131.61 (C-7, 7’, 7″), 130.83 (C-i), 127.22 (C-o), 123.74 (C-5″), 122.89 (C-5,5’), 122.34 (C-6, 6’, 6″), 117.48 (C-m), 116.37 (C-3, 3’, 3″), 115.69 (C-8, 8’, 8″), 114.22 (C-4a, 4a’, 4a″), 60.63 (C-b), 35.10 ppm (C-a), ^15^N NMR (DMSO-*d*_6_): δ_N_ = 247.50 (N-e or g), 152.50 (N-1″), 144.90 ppm (N-1,1’). EI-MS (*m*/*z*, %): 650 (M^+^, 60). Anal. Calcd for C_37_H_26_N_6_O_6_: C, 68.30; H, 4.03; N, 12.92. Found: C, 68.44; H, 3.98; N, 13.02.

*3,3’-((4-((1-(6-Methyl-2-oxo-1,2-dihydroquinolin-4-yl)-1H-1,2,3-triazol-4-yl)methoxy)phenyl)methylene)bis(4-hydroxyquinolin-2(1H)-one)***8b**. Colorless powder, (80%), m.p. 230–32 °C; ^1^H NMR (DMSO-*d*_6_): δ_H_ = 13.17 (bs, 1H; OH-4/4’), 12.68 (bs, 1H; OH-4’/4), 12.20 (bs, 3H; NH-1, 1’, 1″), 8.83 (s, 1H; H-d), 7.96 (m, 2H; H-5, 5’), 7.60 (t, *J* = 7.3, 2H; H-7, 7’), 7.46 (t, *J* = 6.6, 3H; H-8, 8’, 7″), 7.38 (d, *J* = 8.4, 1H; H-8″), 7.30 (b, 2H; H-6, 6’), 7.22 (s, 1H; H-5″), 7.06 (d, *J* = 8.3, 2H; H-o), 7.01 (d, *J* = 8.4, 2H; H-m), 6.80 (s, 1H; H-3″), 6.16 (s, 1H; H-a), 5.26 (s, 2H; H-b), 2.28 ppm (s, 3H; H-6a″), ^13^C NMR (DMSO-*d*_6_): δ_C_ = 160.84 (C-2, 2’, 2″, 4, 4’, 4″), 156.09 (C-p), 143.42, 143.35 (C-c, 4″), 137.50 (C-8a″), 137.01 (C-8a, 8a’), 133.14 (C-7″), 131.73 (C-7, 7’), 131.09 (C-i), 127.43 (C-6″), 126.50 (C-d), 123.15 (C-5, 5’, 5″), 122.49 (C-6, 6’), 117.70 (C-3, 3’, 3″), 115.88 (C-8, 8’, 8″), 114.38 (C-o, m, 4a, 4a’, 4a″), 60.85 (C-b), 34.51 (C-a), 20.50 ppm (C-6a″), ^15^N NMR (DMSO-*d*_6_): δ_N_ = 247.2 (N-e), 151.8 (N-1″), 144.96 ppm (N-1, 1’), N-f, g n/o. EI-MS (*m*/*z*, %): 664 (M^+^, 28). Anal. Calcd for C_38_H_28_N_6_O_6_: C, 68.67; H, 4.25; N, 12.64. Found: C, 68.55; H, 4.33; N, 12.71.

*3,3’-((4-((1-(6-Methoxy-2-oxo-1,2-dihydroquinolin-4-yl)-1H-1,2,3-triazol-4-yl)methoxy)phenyl)methylene)bis(4-hydroxyquinolin-2(1H)-one)***8c**. Colorless powder, (82%), m.p. 255–57 °C; ^1^H NMR (DMSO-*d*_6_): δ_H_ = 13.17 (bs, 1H; OH-4/4’), 12.68 (bs, 1H; OH-4’/4), 12.19 (bs, 3H; NH-1, 1’, 1″), 8.88 (s, 1H; H-d), 7.96 (m, 2H; H-5, 5’), 7.61 (m, 2H; H-7, 7’), 7.43 (bd, *J* = 9.0, 3H; H-8, 8’, 8″), 7.32 (bd, *J* = 8.3, 3H; H-6, 6’, 7″), 7.03 (bd, *J* = 9.7, 4H; H-o,m), 6.92 (bs, 1H; H-5″), 6.85 (s, 1H; H-3″), 6.15 (bs, 1H; H-a), 5.27 (s, 2H; H-b), 3.67 ppm (s, 3H; H-6b″), ^13^C NMR(DMSO-*d*_6_): δ_C_ = 166.40 (b), 164.85 (b; C-2, 2’), 161.38 (b; C-4, 4’), 160.54 (C-2″), 156.09 (C-p), 154.50 (C-6″), 143.50 (C-c), 143.10 (C-4″), 136.98 (C-8a, 8a’), 134.07 (C-8a″), 131.08 (C-7, 7’), 130.01 (b; C-i), 127.43 (C-o), 126.41 (C-d), 123.14 (C-5, 5’), 122.53 (C-6, 6’), 121.03 (C-7″), 117.95 (C-3″), 117.43 (C-8″), 115.88 (C-8, 8’), 114.84 (C-4a″), 114.38 (C-m), 111.38 (b; C-4a, 4a’), 110.15 (b; C-3), 105.51 (C-5″), 60.84 (C-b), 55.35 (C-6b″), 34.51 ppm (b; C-a), ^15^NNMR (DMSO-*d*_6_): δ_N_ = 248.1 (N-e), 151.0 (N-1″), 144.6 ppm (N-1, 1’), N-f, g n/o. EI-MS (*m*/*z*, %): 680 (M^+^, 27). Anal. Calcd for C_38_H_28_N_6_O_7_: C, 67.05; H, 4.15; N, 12.35. Found: C, 66.97; H, 4.01; N, 12.43.

*3,3’-((4-((1-(1-Methly-2-oxo-1,2-dihydroquinolin-4-yl)-1H-1,2,3-triazol-4-yl)methoxy)phenyl)methylene)bis(4-hydroxyquinolin-2(1H)-one)***8d**. Colorless powder, (70%), m.p. 259–61 °C; ^1^H NMR (DMSO-*d*_6_): δ_H_ = 13.18 (bs, 1H; OH-4/4’), 12.68 (bs, 1H; OH-4’/4), 12.25 (bs, 2H; NH-1, 1’), 8.84 (s, 1H; H-d), 7.96 (m, 2H; H-5, 5’), 7.75 (t, *J* = 7.2, 1H; H-7″), 7.70 (d, *J* = 8.2, 1H; H-8″), 7.61 (m, 2H; H-7, 7’), 7.44 (m, 3H; H-8, 8’, 5″), 7.32 (m, 3H; H-6, 6’, 6″), 7.04 (m, 4H; H-o,m), 6.89 (s, 1H; H-3″), 6.16 (bs, 1H; H-a), 5.27 (s, 2H; H-b), 3.71 ppm (s, 3H; H-1a″), ^13^C NMR (DMSO-*d*_6_): δ_C_ = 161.27 (b; C-4, 4’), 160.29 (C-2″), 156.14 (C-p), 143.46 (C-c), 142.62 (C-4″), 140.10 (C-8a″), 136.98 (b; C-8a, 8a’), 132.27 (C-7″), 131.08 (C-7, 7’), 129.97 (b; C-i), 127.50 (C-o), 126.72 (C-d), 124.45 (C-5″), 123.11 (C-5, 5’), 122.71 (C-6, 6’, 6″), 117.18 (C-3″), 115.86 (C-8, 8’), 115.52 (C-8″), 114.43 (C-m), 114.84 (C-4a″), 60.87 (C-b), 34.60 (b; C-a), 29.55 ppm (C-1a″). C-2, 2’, 3, 3’, 4a, 4a’ n/o; ^15^N NMR (DMSO-*d*_6_): δ_N_ = 246.7 (N-e), 147.1 (N-1″), 144.7 ppm (N-1, 1’), N-f, g n/o. EI-MS (*m*/*z*, %): 664 (M^+^, 28). Anal. Calcd for C_38_H_28_N_6_O_6_: C, 68.67; H, 4.25; N, 12.64. Found: C, 68.58; H, 4.19; N, 12.77.

*3,3’-((4-((1-(2-oxo-1,2-dihydroquinolin-4-yl)-1H-1,2,3-triazol-4-yl)methoxy)phenyl)-methylene)bis(4-hydroxy-6-methoxyquinolin-2(1H)-one)***8e**. Colorless powder, (77%), m.p. 269–71 °C; ^1^H NMR (DMSO-*d*_6_): δ_H_ = 13.41 (bs, 1H; OH-4/4’), 12.90 (bs, 1H; OH-4’/4), 12.27 (s, 1H; NH-1″), 12.20 (bs, 1H; NH-1/1’), 12.09 (bs, 1H; NH-1’/1), 8.85 (s, 1H; H-d), 7.64 (dd, *J* = 7.5, 7.5, 1H; H-7″), 7.47 (d, *J* = 8.0, 1H; H-8″), 7.45 (d, *J* = 7.8, 1H; H-5″), 7.40 (m, 3H; H-8, 8’, 5/5’), 7.33 (m, 1H; H-5’/5), 7.25 (d, *J* = 7.3, 2H; H-7, 7’), 7.23 (m, 1H; H-6″), 7.03 (m, 4H; H-o,m), 6.85 (s, 1H; H-3″), 6.15 (s, 1H; H-a), 5.26 (s, 2H; H-b), 3.82 ppm (s, 6H; H-6b, 6b’), ^13^C NMR(DMSO-*d*_6_): δ_C_ = 164.13 (b; C-2,2’), 161.86 (b; C-4,4’), 160.99 (C-2″), 156.09 (C-p), 154.82 (C-6,6’), 143.64 (C-c), 143.42 (C-4″), 139.41 (C-8a″), 131.85 (C-7″), 131.51 (b; C-8a, 8a’), 130.08 (b; C-i), 127.40 (C-o), 126.50 (C-d), 123.98 (C-5″), 122.58 (C-6″), 120.83 (C-7,7’), 117.69 (C-8,8’), 117.47 (C-3″), 116.71 (b; C-4a, 4a’), 115.92 (C-8″), 114.45 (C-m,4a″), 111.77 (b; C-3, 3’), 103.82 (C-5, 5’), 60.87 (C-b), 55.41 (C-6b, 6b’), 34.72 ppm (C-a), ^15^N NMR (DMSO-*d*_6_): δ_N_ = 247.5 (N-e), 152.2 (N-1″), 144.1 (N-1’/1), 143.0 ppm (N-1/1‘), N-f, g n/o. EI-MS (*m*/*z*, %): 710 (M^+^, 28). Anal. Calcd for C_39_H_30_N_6_O_8_: C, 65.91; H, 4.25; N, 11.83. Found: C, 66.09; H, 4.33; N, 11.79.

*3,3’-((4-((1-(6-Methyl-2-oxo-1,2-dihydroquinolin-4-yl)-1H-1,2,3-triazol-4-yl)methoxy)phenyl)methylene)bis(4-hydroxy-6-methoxyquinolin-2(1H)-one)***8f**. Colorless powder, (82%), m.p. 264–66 °C; NMR (DMSO-*d*_6_) (Table 2). EI-MS (*m*/*z*, %): 724 (M^+^, 22). Anal. Calcd for C_40_H_32_N_6_O_8_: C, 66.29; H, 4.45; N, 11.60. Found: C, 66.33; H, 4.42; N, 11.57.

*3,3’-((4-((1-(6-Methoxy-2-oxo-1,2-dihydroquinolin-4-yl)-1H-1,2,3-triazol-4-yl)methoxy)phenyl)methylene)bis(4-hydroxy-6-methoxyquinolin-2(1H)-one)***8g**. Colorless powder, (83%), m.p. 270–72 °C; ^1^H NMR (DMSO-*d*_6_): δ_H_ = 13.41 (bs, 1H; OH-4/4’), 12.91 (bs, 1H; OH-4’/4), 12.21 (bs, 2H; NH-1/1’, 1″), 12.11 (bs, 1H; NH-1’/1), 8.89 (s, 1H; H-d), 7.42 (m, 4H; H-5/5’, 8, 8’, 8″), 7.33 (m, 2H; H-5’/5, 7″), 7.26 (m, 2H; H-7, 7’), 7.03 (m, 4H; H-o,m), 6.92 (s, 1H; H-5″), 6.85 (s, 1H; H-3″), 6.15 (bs, 1H; H-a), 5.27 (s, 2H; H-b), 3.82 (s, 6H; H-6b, 6b’), 3.67 ppm (s, 3H; H-6b″), ^13^C NMR (DMSO-*d*_6_): δ_C_ = 165.84 (b), 164.16 (b; C-2, 2’), 161.83 (b), 160.93 (b; C-4, 4’), 160.52 (C-2″), 156.05 (C-p), 154.81 (C-6, 6’), 154.50 (C-6″), 143.48 (C-c), 143.09 (C-4″), 134.07 (C-8a″), 131.58 (b; C-8a, 8a’), 130.09 (b; C-i), 127.40 (C-o), 126.41 (C-d), 121.04 (C-7″), 120.84 (C-7, 7’), 117.95 (C-3″), 117.44, 116.69 (b; C-8, 8’, 8″), 114.83 (C-4a″), 114.38 (C-m), 111.75 (b; C-3, 3’), 110.23 (b; C-4a, 4a’), 105.50 (C-5″), 103.80 (C-5, 5’), 60.84 (C-b), 55.35, 55.11 (b; C-6b, 6b’, 6b″), 34.71 ppm (b; C-a), ^15^N NMR (DMSO-*d*_6_): δ_N_ = 247.3 (N-e), 151.0 (N-1″), 144.4 (N-1’/1), 143.0 ppm (N-1/1‘), N-f, g n/o. EI-MS (*m*/*z*, %): 740 (M^+^, 26). Anal. Calcd for C_40_H_32_N_6_O_9_: C, 64.86; H, 4.35; N, 11.35. Found: C, 64.80; H, 4.44; N, 11.21.

*3,3’-((4-((1-(1-Methly-2-oxo-1,2-dihydroquinolin-4-yl)-1H-1,2,3-triazol-4-yl)methoxy)phenyl)methylene)bis(4-hydroxy-6-methoxyquinolin-2(1H)-one)***8h**. Colorless powder, (69%), m.p. 260–62 °C; ^1^H NMR (DMSO-*d*_6_): δ_H_ = 13.41 (bs, 1H; OH-4/4’), 12.89 (bs, 1H; OH-4’/4), 12.19 (b, 1H; NH-1/1’), 12.08 (b, 1H; NH-1’/1), 8.84 (s, 1H; H-d), 7.77 (dd, *J* = 7.5, 7.5, 1H; H-7″), 7.71 (d, *J* = 8.3, 1H; H-8″), 7.41 (m, 4H; H-5″,8,8’,5/5’), 7.33 (m, 2H; H-6″,5’/5), 7.26 (bd, *J* = 6.8, 2H; H-7, 7’), 7.02 (m, 4H; H-o,m), 6.94 (s, 1H; H-3″), 6.15 (s, 1H; H-a), 5.27 (s, 2H; H-b), 3.82 (bs, 6H; H-6b, 6b’), 3.72 ppm (s, 3H; H-1a″), ^13^C NMR (DMSO-*d*_6_): δ_C_ = 164.14 (C-2,2’), 162.29 (C-4, 4’), 160.29 (C-2″), 156.09 (C-p), 154.78 (C-6, 6’), 143.42 (C-c), 142.62 (C-4″), 140.10 (C-8a″), 132.26 (C-7″), 131.42 (C-8a, 8a’), 130.09 (C-i), 127.39 (C-o), 126.70 (C-d), 124.44 (C-5″), 122.71 (C-6″), 120.83 (C-7, 7’), 117.47 (C-8, 8’), 117.18 (C-3″, 4a, 4a’), 115.52 (C-8″), 114.41 (C-m, 4a″), 103.82 (C-5, 5’), 60.89 (C-b), 55.41 (C-6b, 6b’), 34.64 (C-a), 29.56 ppm (C-1a″). C-3, 3’ n/o, ^15^N NMR (DMSO-*d*_6_): δ_N_ = 246.5 (N-e), 147.1 (N-1″), 144.2 ppm (N-1, 1’), N-f, g n/o. EI-MS (*m*/*z*, %): 724 (M^+^, 37). Anal. Calcd for C_40_H_32_N_6_O_8_: C, 66.29; H, 4.45; N, 11.60. Found: C, 66.22; H, 4.33; N, 11.75.

*3,3’-((4-((1-(2-oxo-1,2-dihydroquinolin-4-yl)-1H-1,2,3-triazol-4-yl)methoxy)phenyl)-methylene)bis(4-hydroxy-1-methylquinolin-2(1H)-one)***8i**. Colorless powder, (72%), m.p. 265–67 °C; ^1^H NMR (DMSO-*d*_6_): δ_H_ = 12.87 (bs, 1H; OH-4/4’), 12.54 (bs, 1H; OH-4’/4), 12.27 (bs, 1H; NH-1″), 8.85 (s, 1H; H-d), 8.07 (bs, 2H; H-5, 5’), 7.71 (m, 2H; H-7, 7’), 7.64 (m, 3H; H-8, 8’, 7″), 7.46 (m, 2H; H-5″, 8″), 7.39 (b, 2H; H-6, 6’), 7.23 (t, *J* = 7.6, 1H; H-6″), 7.01 (m, 4H; H-o,m), 6.84 (s, 1H; H-3″), 6.25 (bs, 1H; H-a), 5.25 (s, 2H; H-b), 3.73 ppm (bs, 6H; H-1a, 1a’), ^13^C NMR (DMSO-*d*_6_): δ_C_ = 165.77 (b), 164.20 (b; C-2, 2’), 160.97 (C-4, 4’, 2″), 156.11 (C-p), 143.62 (C-4″), 143.40 (C-c), 139.41 (C-8a″), 138.14 (C-8a, 8a’), 131.84 (C-7, 7’, 7″), 131.58 (C-i), 127.41 (C-o), 126.51 (C-d), 123.97 (C-5″), 123.61 (C-5, 5’), 122.74 (C-6, 6’), 122.56 (C-6″), 117.69 (C-3″), 116.94 (b; C-4a, 4a’), 115.90 (C-8″), 115.34 (C-8, 8’), 114.44 (C-m), 110.92 (b; C-3, 3’), 60.84 (C-b), 36.00 (C-a), 30.18 ppm (C-1a, 1a’), ^15^N NMR (DMSO-*d*_6_): δ_N_ = 247.5 (N-e), 152.2 ppm (N-1″), N-1, 1’, f, g n/o. EI-MS (*m*/*z*, %): 678 (M^+^, 35). Anal. Calcd for C_39_H_30_N_6_O_6_: C, 69.02; H, 4.46; N, 12.38. Found: C, 68.99; H, 4.35; N, 12.55.

*3,3’-((4-((1-(6-Methyl-2-oxo-1,2-dihydroquinolin-4-yl)-1H-1,2,3-triazol-4-yl)methoxy)phenyl)methylene)bis(4-hydroxy-1-methylquinolin-2(1H)-one)***8j**. Colorless powder, (77%), m.p. 222–24 °C; ^1^H NMR (DMSO-*d*_6_): δ_H_ = 12.92 (bs, 1H; OH-4/4’), 12.50 (bs, 1H; OH-4’/4), 12.22 (bs, 1H; NH-1″), 8.83 (s, 1H; H-d), 8.03 (b, 2H; H-5, 5’), 7.65 (m, 4H; H-7, 7’, 8, 8’), 7.42 (bd, *J* = 8.6 Hz, 1H; H-7″), 7.35 (d, *J* = 8.2 Hz, 3H; H-6, 6’, 8″), 7.20 (s, 1H; H-5″), 6.99 (m, 4H; H-o, m), 6.80 (s, 1H; H-3″), 6.23 (bs, 1H; H-a), 5.25 (s, 2H; H-b), 3.71 (bs, 6H; H-1a, 1a’), 2.25 ppm (s, 3H; H-6a″), ^13^C NMR (DMSO-*d*_6_): δ_C_ = 165.58 (b), 164.36 (b; C-2, 2’, 2″), 160.84 (C-4, 4’, 4″), 158.06 (C-p), 143.39 (C-c), 138.08 (C-8a, 8a’), 137.50 (C-8a″), 133.08 (C-7″), 131.68, 131.47 (C-7, 7’), 129.91 (b; C-i), 127.39 (C-o, 6″), 126.48 (C-d), 123.57 (C-5, 5’), 123.12 (C-5″), 122.64 (C-6, 6’), 117.67 (C-3″), 116.90 (b; C-4a, 4a’), 115.87 (C-8″), 115.20 (C-8, 8’), 114.36, 114.32 (C-m, 4a″), 110.49 (b), 109.92 (b; C-3, 3’), 60.81 (C-b), 36.00 (C-a), 30.07 (b; C-1a, 1a’), 20.47 ppm (C-6a″), ^15^N NMR (DMSO-*d*_6_): δ_N_ = 247.9 (N-e), 151.9 ppm (N-1″), N-1, 1’, f, g n/o. EI-MS (*m*/*z*, %): 692 (M^+^, 10). Anal. Calcd for C_40_H_32_N_6_O_6_: C, 69.35; H, 4.66; N, 12.13. Found: C, 69.44; H, 4.55; N, 12.22.

*3,3’-((4-((1-(6-Methoxy-2-oxo-1,2-dihydroquinolin-4-yl)-1H-1,2,3-tri-azol-4-yl)methoxy)phenyl)methylene)bis(4-hydroxy-1-methylquinolin-2(1H)-one))***8k**. Colorless powder, (71%), m.p. 235–37 °C; ^1^H NMR (DMSO-*d*_6_): δ_H_ = 12.84 (bs, 1H; OH-4/4’) 12.55 (bs, 1H; OH-4’/4), 12.44 (bs, 1H; NH-1″), 8.86 (s, 1H; H-d), 8.02 (bs, 2H; H-5, 5’), 8.87(m, 4H; H-7, 7’, 8, 8’), 7.85 (d, 1H; H-7″), 7.39 (d, 3H; H-6, 6’, 8″), 7.31 (s, 1H; H-5″), 6.92 (m, 4H; H-o, m), 6.65 (s, 1H; H-3″), 6.23 (bs, 1H; H-a), 5.42 (s, 2H; H-b), 3.09 (bs, 6H; H-1a, 1a’), 2.49 ppm (s, 3H; H-6a″), ^13^C NMR (DMSO-*d*_6_): δ_C_ = 160.27 (C-4, 4’, 4″), 156.12 (C-p), 143.52 (C-c), 138.08 (C-8a, 8a’), 132.20 (C-8a″), 131.51 (C-7″), 130.43, 130.07 (C-7, 7’), 127.44 (b; C-i), 126.73 (C-o, 6″), 124.45 (C-5, 5’), 123.60 (C-5″), 122.68 (C-6, 6’), 117.11 (C-3″), 117.51 (b; C-4a, 4a’), 115.42 (C-8″), 115.23 (C-8, 8’), 114.36 (C-m), 60.81 (C-b), 36.05 (C-a), 30.09 ppm (b; C-1a, 1a’). EI-MS (*m*/*z*, %): 708 (M^+^, 21). Anal. Calcd for C_40_H_32_N_6_O_7_: C, 67.79; H, 4.55; N, 11.86. Found: C, 67.88; H, 4.66; N, 12.01.

*3,3’-((4-((1-(1-Methyl-2-oxo-1,2-dihydroquinolin-4-yl)-1H-1,2,3-triazol-4-yl)methoxy)phenyl)methylene)bis(4-hydroxy-1-methylquinolin-2(1H)-one)***8l**. Colorless powder, (66%), m.p. 220–22 °C; ^1^H NMR (DMSO-*d*_6_): δ_H_ = 12.15 (bs, 2H; OH-4/4’, OH-4’/4), 12.21 (bs, 1H; NH-1″), 8.86 (s, 1H; H-d), 8.02 (bs, 2H; H-5, 5’), 7.61 (m, 4H; H-7, 7’, 8, 8’), 7.39 (d, 1H; H-7″), 7.31 (d, 3H; H-6, 6’, 8″), 7.28 (s, 1H; H-5″), 6.98 (m, 4H; H-o, m), 6.90 (s, 1H; H-3″), 6.23 (bs, 1H; H-a), 5.24 (s, 2H; H-b), 2.31 (bs, 6H; H-1a, 1a’), 2.29 ppm (s, 3H; H-6a″), ^13^C NMR (DMSO-*d*_6_): δ_C_ = 160.65 (C-4, 4’, 4″), 156.16 (C-p), 143.17 (C-c), 138.16 (C-8a, 8a’), 134.16 (C-8a″), 131.58 (C-7″), 131.58, 130.07 (C-7, 7’), 127.51 (b; C-i), 126.50 (C-o, 6″), 123.68 (C-5, 5’), 122.75 (C-5″), 121.06 (C-6, 6’), 117.98 (C-3″), 117.51 (b; C-4a, 4a’), 115.30 (C-8″), 115.20 (C-8, 8’), 114.88, 114.40 (C-m, 4a″), 105.56 (b; C-3, 3’), 60.88 (C-b), 55.40 (C-6b″), 36.10 (C-a), 30.19 ppm (b; C-1a, 1a’. EI-MS (*m*/*z*, %): 692 (M^+^, 18). Anal. Calcd for C_40_H_32_N_6_O_6_: C, 69.35; H, 4.66; N, 12.13. Found: C, 69.43; H, 4.51; N, 12.21.

### 4.2. Pharmacological Assays

#### 4.2.1. Cytotoxic Activity Using MTT Assay and Evaluation of IC_50_

##### MTT Assay

An MTT assay was performed to investigate the effect of the synthesized compounds on mammary epithelial cells (MCF-10A) at a concentration of 50 µM for the tested compound. The cells were propagated in a medium consisting of Ham’s F-12 medium/Dulbecco’s modified Eagle’s medium (DMEM) (1:1) supplemented with 10% fetal calf serum, 2 mM glutamine, insulin (10 μg/mL), hydrocortisone (500 ng/mL), and epidermal growth factor (20 ng/mL). Trypsin ethylenediamine tetra acetic acid (EDTA) was used to pass the cells every 2–3 days. Ninety-six-well flat-bottomed cell culture plates were used to seed the cells at a density of 104 cells mL^−1^. The medium was aspirated from all the wells of culture plates after 24 h followed by the addition of synthesized compounds (in 200 μL medium to yield a final concentration of 0.1% (*v*/*v*) dimethylsulfoxide) into individual wells of the plates. Four wells were designated to a single compound. The plates were allowed to incubate at 37 °C for 96 h. Afterward, the medium was aspirated, and 3-[4,5-dimethylthiazol-2-yl]-2,5-diphenyltetrazolium bromide (MTT) (0.4 mg/mL) in the medium was added to each well and subsequently incubated for 3 h. The medium was aspirated, and 150 μL dimethyl sulfoxide (DMSO) was added to each well. The plates were vortexed, followed by the measurement of absorbance at 540 nm on a microplate reader. The results were presented as inhibition (%) of proliferation compared to controls comprising 0.1% DMSO.

##### Assay for the Anti-Proliferative Effect

To explore the anti-proliferative potential of compounds, propidium iodide fluorescence assay was performed using different cell lines such as Panc-1 (pancreas cancer cell line), MCF-7 (breast cancer cell line), HT-29 (colon cancer cell line), and A-549 (epithelial cancer cell line), respectively. To calculate the total nuclear DNA, a fluorescent dye (propidium iodide, PI) is used to attach to the DNA, thus offering a quick and precise technique. PI cannot pass through the cell membrane, and its signal intensity can be considered as directly proportional to the quantity of cellular DNA. Cells whose cell membranes are damaged or have changed permeability are counted as dead ones. The assay was performed by seeding the cells of different cell lines at a density of 3000–7500 cells/well (in 200 μL medium) in culture plates followed by incubation for 24 h at 37 °C in humidified 5%CO2/95% air atmospheric conditions. The medium was removed; the compounds were added to the plates at 10 μM concentrations (in 0.1% DMSO) in triplicates, followed by incubation for 48h. DMSO (0.1%) was used as a control. After incubation, the medium was removed, followed by the addition of PI (25 μL, 50 μg/mL in water/medium) to each well of the plates. At −80 °C, the plates were allowed to freeze for 24 h, followed by thawing at 25 °C. A fluorometer (Polar-Star BMG Tech, Beijing, China) was used to record the readings at excitation and emission wavelengths of 530 and 620 nm for each well. The percentage cytotoxicity of compounds was calculated using the following formula:

$$\%\;\mathrm{Cytotoxicity}=\frac{A_{C}-A_{TC}}{A_{C}}\times 100$$

where *A_TC_* = absorbance of treated cells and *A_C_* = absorbance of control. Doxorubicin was used as a positive control in the assay.

#### 4.2.2. Caspase 3 Activation Assay

All reagents were allowed to reach room temperature before use. All liquid reagents were gently mixed before use. The number of 8-well strips needed for the assay was determined. These were inserted into the frame(s) for current use. A total of 100 μL of the Standard Diluent Buffer was added to the zero standard wells. Well(s) reserved for chromogen blank were left empty. A total of 100 μL of standards and controls or diluted samples was added to the appropriate microtiter wells. The sample dilution chosen was optimized for each experimental system. The side of the plate was gently tapped on to mix. Wells were covered with a plate cover and incubated for 2 h at room temperature. The solution was thoroughly aspirated or decanted from the wells and the liquid was discarded; wells were washed four times. A total of 100 μL of Caspase 3 (Active) Detection Antibody solution was pipetted into each well except the chromogen blank(s). The side of the plate was gently tapped on to mix. The plate was covered with a plate cover and incubated for 1 h at room temperature. The solution was thoroughly aspirated or decanted from wells and the liquid was discarded; wells were washed four times. A total of 100 μL Anti-Rabbit IgG HRP Working Solution was added to each well except the chromogen blank(s).

The working dilution was prepared as described in Preparing IgG HRP. Wells were covered with the plate cover and incubated for 30 min at room temperature. The solution was thoroughly aspirated or decanted from the wells and the liquid was discarded. Wells were washed four times. A total of 100 μL of Stabilized Chromogen was added to each well. The liquid in the wells began to turn blue. It was incubated for 30 min at room temperature and in the dark. The incubation time for chromogen substrate was determined by the microtiter plate reader used.

Many plate readers have the capacity to record a maximum optical density (O.D.) of 2.0. The O.D. values were monitored, and the substrate reaction was stopped before the O.D. of the positive wells exceeds the limits of the instrument. The O.D. values at 450 nm could only be read after the Stop Solution had been added to each well. A total of 100 μL of Stop Solution was added to each well. The side of the plate was gently tapped to mix. The solution in the wells changed from blue to yellow. The absorbance of each well was read at 450 nm, having blanked the plate reader against a chromogen blank composed of 100 μL each of Stabilized Chromogen and Stop Solution. The plate was read within 2 h after adding the Stop Solution. A curve fitting software was used to generate the standard curve. A four-parameter algorithm provided the best standard curve fit. The concentrations for unknown samples and controls from the standard curve were read. Value(s) obtained for sample(s) by the appropriate dilution factor were multiplied to correct for the dilution in step 3. Samples producing signals greater than that of the highest standard were diluted in Standard Diluent Buffer and reanalyzed.

#### 4.2.3. Caspase 8 Activation Assay

Cells were obtained from American Type Culture Collection; cells were grown in RPMI 1640 containing 10% fetal bovine serum at 37 °C, stimulated with the compounds to be tested for caspase8, and lysed with Cell Extraction Buffer. This lysate was diluted in Standard Diluent Buffer over the range of the assay and measured for human active Caspase 8 content. (cells were plated in a density of 1.2–1.8 × 10,000 cells/well in a volume of 100 µL complete growth medium + 100 µL of the tested compound per well in a 96-well plate for 24 h before the enzyme assay for Tubulin). The absorbance of each microwell was read on a spectro-photometer at 450 nm. A standard curve was prepared from seven human Caspase 8 standard dilutions, and human Caspase 8 concentration was determined.

#### 4.2.4. Bax Activation Assay

All reagents, except the human Bax-α Standard, were brought to room temperature for at least 30 min before opening. The human Bax-α Standard solution was not be left at room temperature for more than 10 min. All standards, controls, and samples were run in duplicate. The Assay Layout Sheet was used to determine the number of wells to be used, and any remaining wells were put with the desiccant back into the pouch, and the ziplock was sealed. Unused wells were stored at 4 °C. A total of 100 μL of Assay Buffer was pipetted into the S0 (0 pg/mL standard) wells. A total of 100 μL of Standards #1 through #6 was pipetted into the appropriate wells. A total of 100 μL of the samples was pipetted into the appropriate wells. The plate was gently tapped to mix the contents. The plate was sealed and incubated at room temperature on a plate shaker for 1 h at ~500 rpm. The contents of the wells were emptied and washed by adding 400 μL of wash solution to every well. The wash was repeated four more times for a total of five washes. After the final wash, the wells were emptied or aspirated and firmly the plate on a lint-free paper towel was tapped to remove any remaining wash buffer. A total of 100 μL of yellow antibody was pipetted into each well, except the Blank. The plate was sealed and incubated at room temperature on a plate shaker for 1 h at ~500 rpm. The contents of the wells were emptied and washed by adding 400 μL of wash solution to every well. The wash was repeated four more times for a total of five washes. After the final wash, the wells were emptied or aspirated, and the plate was firmly tapped on a lint-free paper towel to remove any remaining wash buffer. A total of 100 μL of blue Conjugate was added to each well, except the Blank. The plate was sealed and incubated at room temperature on a plate shaker for 30 min at ~500 rpm. The contents of the wells were emptied and washed by adding 400 μL of wash solution to every well. The wash was repeated four more times for a total of five washes. After the final wash, the wells were emptied or aspirated and the plate was firmly tapped on a lint-free paper towel to remove any remaining wash buffer. A total of 100 μL of Substrate Solution was pipetted into each well. It was incubated for 30 min at room temperature on a plate shaker at ~500 rpm. A total of 100 μL Stop Solution was pipetted into to each well. The plate reader was blanked against the Blank wells, and the optical density was read at 450 nm. The average net optical density (OD) bound for each standard and sample was calculated by subtracting the average Blank OD from the average OD for each standard and sample. Using linear graph paper, the Average Net OD for each standard versus Bax concentration in each standard were plotted. A straight line was approximated through the points. The concentration of Bax in the unknowns could be determined by interpolation.

#### 4.2.5. Bcl-2 Inhibition Assay

All reagents were thoroughly mixed without foaming before use. The microwells were washed twice with approximately 300 μL Wash Buffer per well with the thorough aspiration of microwell contents between washes. Care was taken not to scratch the surface of the microwells. After the last wash, the wells were emptied and microwell strips were tapped on an absorbent pad or paper towel to remove excess Wash Buffer. The microwell strips were used immediately after washing or placed upside down on a wet absorbent paper for not longer than 15 min. Wells were not allowed to dry. A total of 100 μL of Sample Diluent was added in duplicate to all standard wells and the blank wells. Standard (1:2 dilution) in duplicate was prepared ranging from 32 to 0.5 ng/mL. A total of 100 μL of Sample Diluent, in duplicate, was added to the blank wells. A total of 80 μL of Sample Diluent, in duplicate, was added to the sample wells. A total of 20 μL of each sample, in duplicate, was added to the designated wells. A total of 50 μL of diluted biotin was added, conjugated to all wells, including the blank wells. It was covered with a plate cover and incubated at room temperature on a microplate shaker at 100 rpm for 2 h. The plate cover was removed and the wells were emptied. Microwell strips were washed times as described in Step 2. A total of 100 μL of diluted Streptavidin-HRP was added to all wells, including the blank wells. It was covered with a plate cover and incubated at room temperature on a microplate shaker at 100 rpm for 1 h. The plate cover was removed and the wells were emptied. Microwell strips were washed three times, as described in Step 2. A total of 100 μL of mixed TMB Substrate Solution was pipetted into all wells, including the blanks. The microwell strips were incubated at room temperature (18 °C to 25 °C) for about 15 min, on a rotator set at 100 rpm. Direct exposure to intense light was avoided. The point at which the substrate reaction is stopped was determined by the ELISA reader. Many ELISA readers record absorbance only up to 2.0 O.D. Therefore, the color development within individual microwells was watched by the person running the assay, and the substrate reaction was stopped before positive wells were no longer properly detectable. The enzyme reaction was stopped by quickly pipetting 100 μL of Stop Solution into each well, including the blank wells. It was important that the Stop Solution was spread quickly and uniformly throughout the microwells to inactivate the enzyme completely. Results were read immediately after the Stop Solution was added or within one hour if the microwell strips were stored at 2–8 °C in the dark. The absorbance of each microwell was read on a spectrophotometer using 450 nm as the primary wavelength.

#### 4.2.6. Cytochrome C Assay

Cells were obtained from American Type Culture Collection, grown in RPMI 1640 containing 10% fetal bovine serum at 37 °C, stimulated with the compounds to be tested for cytochrome C, and lysed with Cell Extraction Buffer. This lysate was diluted in Standard Diluent Buffer over the range of the assay and measured for cytochrome C content. (cells were plated in cells/well in a volume of 100 µL complete growth medium + 100 µL of the tested compound + 50 µL of 1× biotin-conjugated antibody + 100 µL of 1× streptavidin-HRP + 100 µL TMB substrate soln of per well in a 96-well plate for 24 h before assay).

#### 4.2.7. Cell Apoptosis Assay

Apoptosis was determined by flow cytometry based on the annexin V-fluorescein isothiocyanate (FITC) and propidium iodide (PI) staining kit (BD Pharmingen, San Diego, USA). Apoptotic cells were defined as Annexin-V-positive. Cells were grown to approximately ~70% confluency and exposed to the tested compounds (8 μmol/L) for 24 h. Treated cells were trypsinized, washed twice with PBS, and transferred into microcentrifuge tubes for centrifugation at 1000 rpm for 5 min at room temperature, and then resuspended in binding buffer; 5 μL of FITC and PI were added to per Eppendorf tube, and cells were vortexed and incubated for 15 min at room temperature in the dark. Subsequently, cells were analyzed by flow cytometry (Becton Dickinson, Franklin Lakes, NJ, USA).

## Figures and Tables

**Table 1:** Spectrum data for compound **4b**.

**^1^H NMR**	**^1^H-^1^HCOSY**		**Assignment**
13.39 (bs; 1H)			OH-4/4’
12.90 (bs; 1H)			OH-4’/4
12.19 (bs; 1H)			NH-1/1’
12.08 (bs; 1H)			NH-1’/1
7.40 (d, *J* = 9.0; 2H)	7.26		H-8, 8’
7.40 (bs; 1H)			H-5/5’
7.33 (bs; 1H)			H-5’/5
7.26 (bd, *J* = 8.6; 2H)	7.40		H-7, 7’
7.02 (d, *J* = 8.5; 2H)	6.89		H-o
6.89 (d, *J* = 8.8; 2H)	7.02		H-m
6.14 (s; 1H)			H-a
4.76 (d, *J* = 2.0; 2H)	3.55		H-b
3.77 (s; 6H)			H-6a, 6a’
3.55 (bs; 1H)	4.76		H-d
**^15^N NMR**	**HSQC**	**HMBC**	**Assignment**
144.2	12.08	7.40	N-1/1’
142.9	12.19	7.40	N-1’/1
**^13^C NMR**	**HSQC**	**HMBC**	**Assignment**
165.76, 164.10		6.14	C-2, 2’
161.84, 160.91		6.14	C-4, 4’
155.31		7.02, 6.89, 4.76	C-p
154.80		7.40, 7.26, 3.77	C-6, 6’
131.52		7.40, 7.26	C-8a, 8a’
130.29		6.89, 6.14	C-i
127.29	7.02	7.02, 6.14	C-o
120.63	7.26		C-7, 7’
117.46	7.40	7.40	C-8, 8’
117.21, 116.70		7.40, 7.33	C-4a, 4a’
114.35	6.89	6.89, 6.89	C-m
111.74, 111.25		6.14	C-3, 3’
103.82	7.40, 7.33		C-5, 5’
79.43		4.76	C-c
78.00	3.55	3.55	C-d
55.41	3.77		C-6a, 6a’
55.34	4.76		C-b
34.69	6.14	7.02, 6.14	C-a

**Table 2:** Spectrum data for compound **8f**.

**^1^H NMR**	**^1^H-^1^HCOSY**		**Assignment**
13.41 (bs; 1H)			OH-4/4’
12.91 (bs; 1H)			OH-4’/4
12.20 (s; 2H)	6.81		NH-1/1’, 1″
12.09 (bs; 1H)			NH-1’/1
8.84 (s; 1H)			H-d
7.47 (d, *J* = 8.3; 1H)	7.38, 7.22		H-7″
7.40 (d, *J* = 9.2; 2H)	7.26		H-8, 8’
7.38 (d, *J* = 8.4; 2H)	7.47, 7.26, 2.29		H-5/5’, 8″
7.33 (m; 1H)	7.26		H-5’/5
7.26 (d, *J* = 8.1; 2H)	7.40, 7.38, 7.33		H-7, 7’
7.22 (s; 1H)	7.47, 2.29		H-5″
7.05 (d, *J* = 8.2; 2H)	7.01, 6.15		H-o
7.01 (d, *J* = 8.2; 2H)	7.05		H-m
6.81 (s; 1H)	12.20		H-3″
6.15 (bs; 1H)	7.05		H-a
5.27 (s; 2H)			H-b
3.82 (s; 6H)			H-6b, 6b’
2.29 (s; 3H)	7.40, 7.38, 7.26		H-6a″
**^13^C NMR**	**HSQC**	**HMBC**	**Assignment**
165.85, 164.13		6.15	C-2, 2’
161.84			C-4, 4’
160.84			C-2″, 4″
156.06		7.05, 5.27	C-p
154.81		7.40, 3.82	C-6, 6’
143.42		8.84, 7.40, 7.22, 6.81, 5.27	C-c
137.50		7.47, 7.22	C-8a″
133.15	7.47	7.47, 7.22	C-7″
131.73, 131.51		7.38, 7.26, 2.29	C-8a, 8a’
130.07			C-i
127.40	7.05		C-o, 6″
126.50	8.84	8.84,3 7.01, 5.27	C-d
123.15	7.22	7.47, 7.05, 2.29	C-5″
120.82	7.26	7.01	C-7, 7’
117.69	6.81	7.05, 6.813	C-3″
117.45	7.40	7.403	C-8, 8’
116.69	7.38, 7.33		C-4a, 4a’
115.89	7.38		C-8″
114.38	7.01	7.38, 6.81	C-m, 4a″
111.86, 110.18		6.15	C-3, 3’
103.81	7.38, 7.33		C-5, 5’
60.85	5.27		C-b
55.40	3.82	3.823	C-6b, 6b’
34.71	6.15		C-a
20.50	2.29	7.47, 7.22, 2.293	C-6a″
**^15^N NMR**	**HSQC**	**HMBC**	**Assignment**
247.4		8.84, 6.81	N-e
151.6	12.20	7.38, 6.81	N-1″
144.2	12.09		N-1’/1
142.	12.20		N-1/1‘
N-f, g n/o.			

**Table 3:** Anti-proliferative activity of compounds **8a**–**l** and doxorubicin.

Comp.	Cell Viability% (50 µM)	Anti-Proliferative Activity IC_50_ ± SEM (µM)				
		A-549	MCF-7	Panc-1	HT-29	Average (IC_50_)
**8a**	89	5.7 ± 0.4	5.3 ± 0.8	5.6 ± 0.6	5.7 ± 0.4	5.575
**8b**	87	7.6 ± 0.8	6.9 ± 0.8	7.6 ± 0.6	7.5 ± 0.4	7.400
**8c**	91	3.9 ± 0.5	3.2 ± 0.08	4.1 ± 0.2	4.2 ± 0.2	3.850
**8d**	89	12.9 ± 0.8	11.5 ± 1.1	11.6 ± 0.8	11.4 ± 1.2	11.850
**8e**	85	2.1 ± 0.2	1.5 ± 0.7	1.7 ± 0.1	2.2 ± 0.4	1.875
**8f**	87	2.9 ± 0.5	2.6 ± 0.8	3.1 ± 0.2	3.4 ± 0.2	3.000
**8g**	84	1.9 ± 0.2	1.2 ± 0.2	1.4 ± 0.2	1.8 ± 0.4	1.575
**8h**	90	10.7 ± 2.5	10.6 ± 2.9	10.8 ± 1.9	10.8 ± 1.6	10.725
**8i**	90	17.9 ± 0.3	17.8 ± 1.6	17.6 ± 1.5	17.9 ± 1.1	17.800
**8j**	81	22.5 ± 0.2	22.1 ± 0.1	22.4 ± 0.2	22.9 ± 0.6	22.474
**8k**	90	14.5 ± 2.6	13.6 ± 2.2	14.6 ± 2.9	14.8 ± 1.4	14.375
**8l**	96	25.3 ± 2.5	24.9 ± 1.8	24.5 ± 2.3	24.2 ± 1.4	24.725
Doxorubicin	--	1.21 ± 0.80	0.90 ± 0.62	1.41 ± 0.58	1.01 ± 0.82	1.136

**Table 4:** Effects of compounds **8e**, **8f**, **8g**, and doxorubicin on active Caspases 3, 8, 9, and cytochrome C in MCF-7 breast cancer cell line.

Compound No.	Caspase 3		Caspase 8		Caspase 9		Cytochrome C	
	Conc (pg/mL)	Fold Change	Conc (ng/mL)	Fold Change	Conc (ng/mL)	Fold Change	Conc (ng/mL)	Fold Change
**8e**	475.20 ± 4.27	7.23	1.29	7.58	12.89	13.86	0.548	11.90
**8f**	365.60 ± 3.20	5.57	ND	ND	ND	ND	ND	ND
**8g**	489.20 ± 4.13	7.54	1.52	8.94	13.98	15.03	0.587	12.76
Doxorubicin	503.20 ± 4.22	7.66	1.75	10.07	16.23	17.40	0.604	13.13
Control	65.64	1	0.17	1	0.93	1	0.046	1

ND: Not determined.

**Table 5:** Bax and Bcl-2 levels for compounds **8e**, **8g,** and Doxorubicin in MCF-7 breast cancer cell line.

Compound No.	Bax		Bcl-2	
	Conc (pg/mL)	Fold Change	Conc (ng/mL)	Fold Reduction
**8e**	247.65	29.98	1.278	3.97
**8g**	264.90	32.07	1.085	4.68
Doxorubicin	276.19	33.42	0.983	5.17
Cont.	8.26	1	5.086	1.00

## Data Availability

Not applicable.

## Supplementary Materials

The following are available online, Spectral data of all newly synthesized compounds.

## Sample Availability

Samples of the compounds are not available from the authors.
