# Supplementary material for: Design and Synthesis of (2-oxo-1,2-Dihydroquinolin-4-yl)-1,2,3-triazole Derivatives via Click Reaction: Potential Apoptotic Antiproliferative Agents
Source: Molecules. 2021 Nov 10;26(22):6798. doi: 10.3390/molecules26226798 (PMC8620910; doi:10.3390/molecules26226798)
Supplement: Supplementary file 1 [file molecules-26-06798-s001.zip › molecules-1422350-supplementary.pdf]

Supplementary Materials

Design and Synthesis of (2-*oxo*-1,2-Dihydroquinolin-4-yl)-1,2,3-triazole Derivatives via Click Reaction: Potential Apoptotic Antiproliferative Agents

Essmat M. El-Sheref [1,*], Mohammed A. I. Elbastawesy [2], Alan B. Brown [3], Ahmed M. Shawky [4], Hesham A. M. Gomaa [5], Stefan Bräse [6,7,*] and Bahaa G. M. Youssif [8,*]

[1] Chemistry Department, Faculty of Science, Minia University, El-Minia 61519, Egypt
[2] Department of Pharmaceutical Organic Chemistry, Faculty of Pharmacy, Al-Azhar University, Assiut 71524, Egypt; mohamedali.pharm.ast@azhar.edu.eg
[3] Program in Chemistry, Florida Institute of Technology, 150 W University Blvd, Melbourne, FL 32901, USA; abrown@fit.edu
[4] Science and Technology Unit (STU), Umm Al-Qura University, Makkah 21955, Saudi Arabia; amesmail@uqu.edu.sa
[5] Pharmacology Department, College of Pharmacy, Jouf University, Sakaka 72314, Saudi Arabia; hasoliman@ju.edu.sa
[6] Institute of Organic Chemistry, Karlsruhe Institute of Technology, 76131 Karlsruhe, Germany
[7] Institute of Biological and Chemical Systems (IBCS-FMS), Karlsruhe Institute of Technology, 76344 Eggenstein-Leopoldshafen, Germany
[8] Pharmaceutical Organic Chemistry Department, Faculty of Pharmacy, Assiut University, Assiut 71526, Egypt
* Correspondence: essmat_elsheref@mu.edu.eg (E.M.E.-S.); braese@kit.edu (S.B.); bgyoussif@ju.edu.sa (B.G.M.Y.); Tel: +20-1064890489 (E.M.E.-S.); +49-721-608-42902 (S.B.); +20-1098294419 (B.G.M.Y.)

Spectrum data for Compound 4a

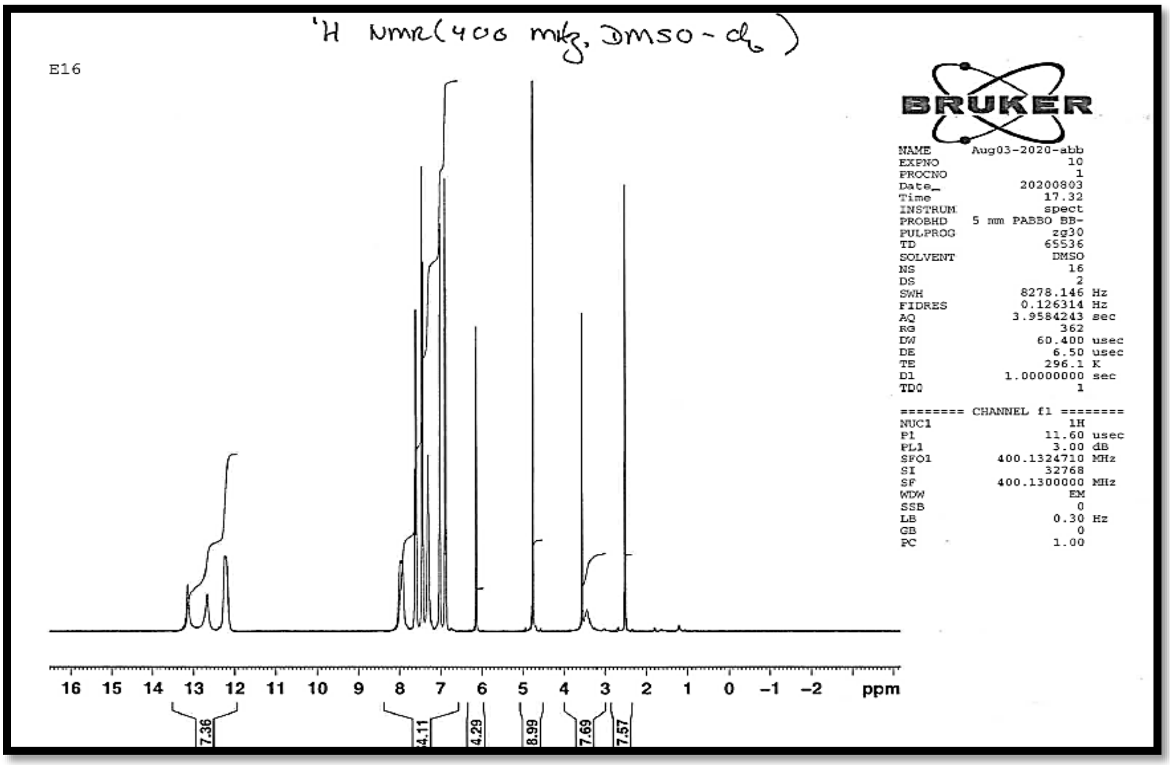

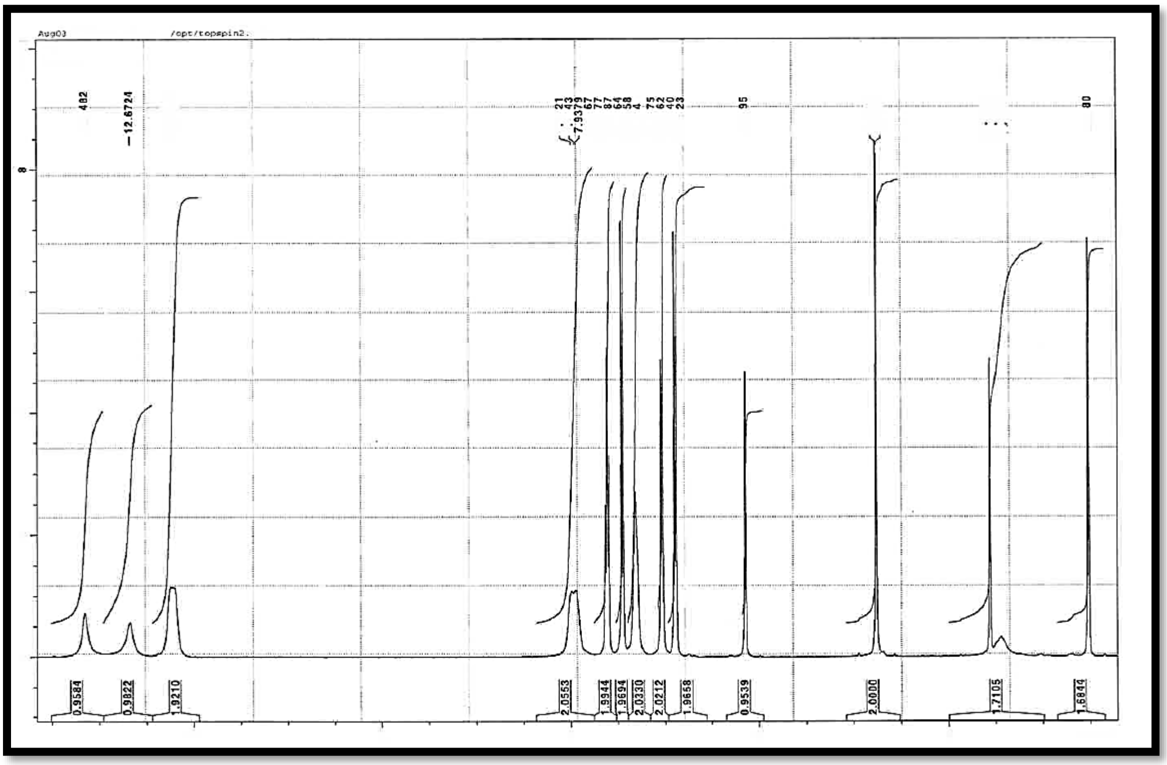

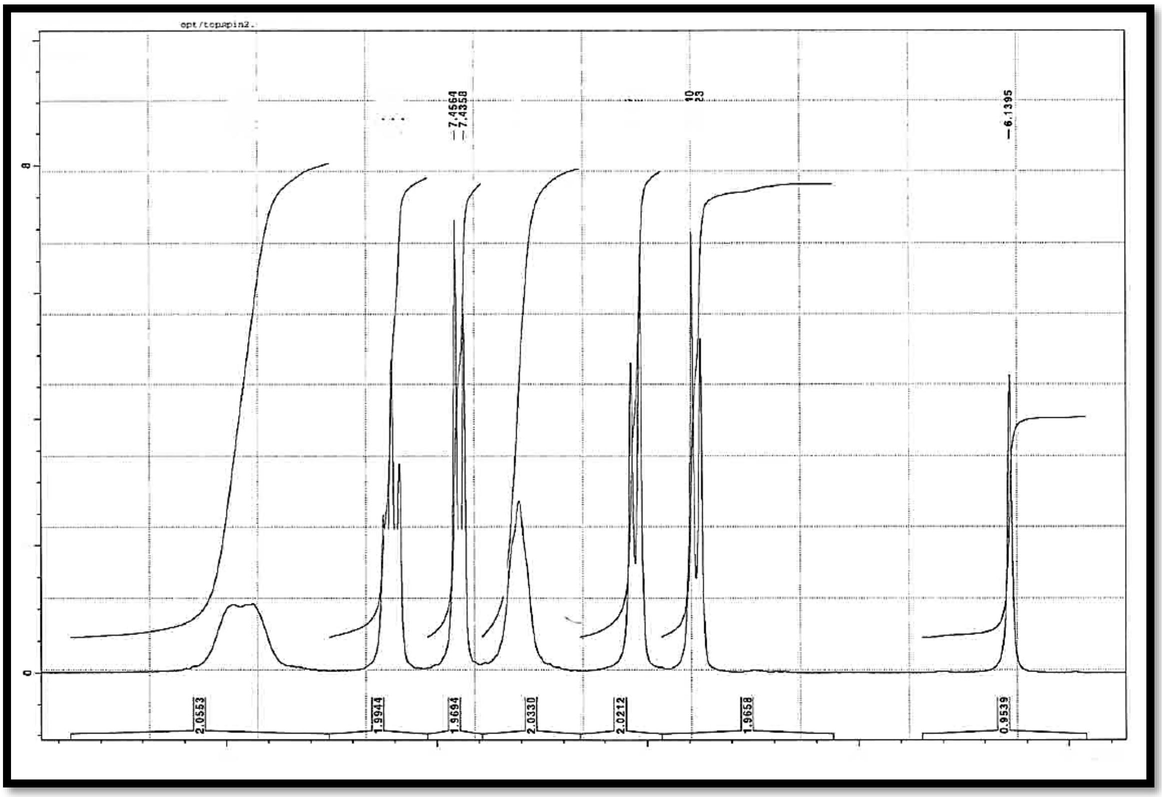
opt/topspin2.
7.4564
7.4358
6.1395
2.0553
1.9944
1.9694
2.0330
2.0212
1.9658
0.9539

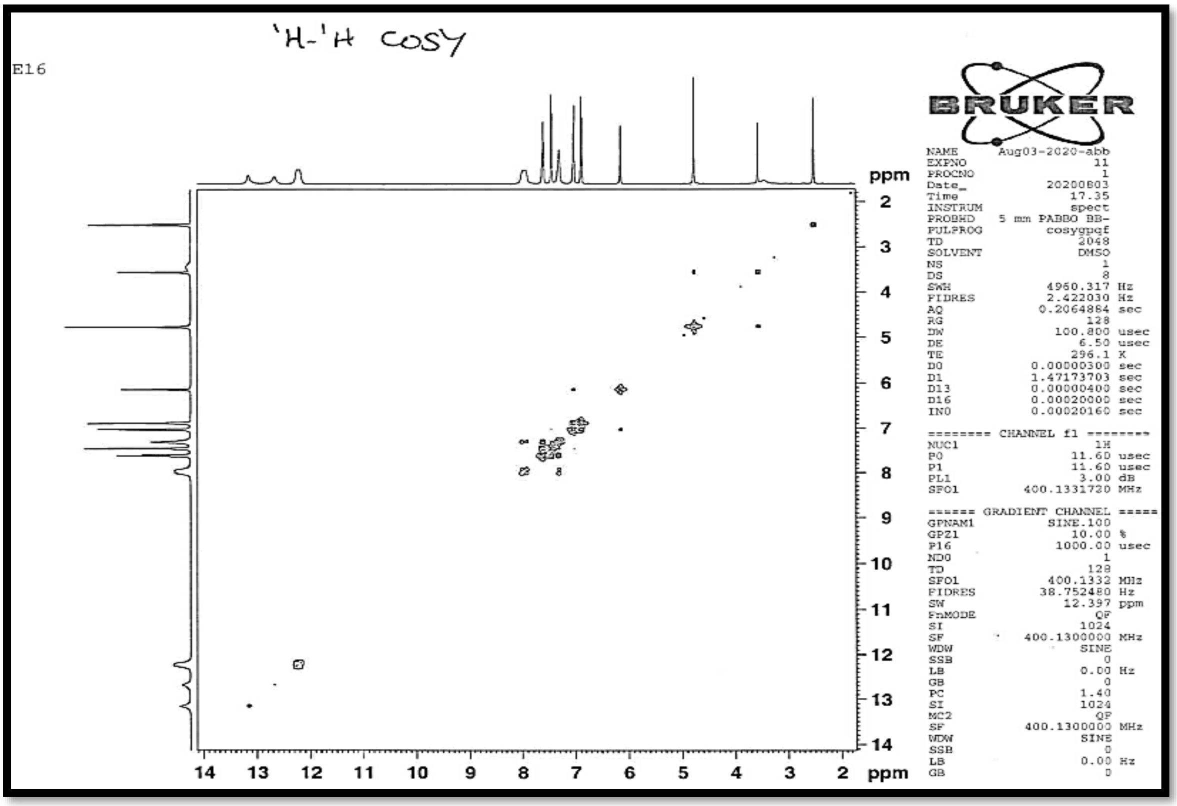
'H-'H COSY
E16
BRUKER
ppm
14 13 12 11 10 9 8 7 6 5 4 3 2 ppm
2 3 4 5 6 7 8 9 10 11 12 13 14
NAME Aug03-2020-abb
EXPNO 11
PROCNO 1
Date_ 20200803
Time 17.35
INSTRUM spect
PROBHD 5 mm PABBO BB-
PULPROG cosygpqf
TD 2048
SOLVENT DMSO
NS 1
DS 8
SWH 4960.317 Hz
FIDRES 2.422030 Hz
AQ 0.2064884 sec
RG 128
DW 100.800 usec
DE 6.50 usec
TE 296.1 K
D0 0.00000300 sec
D1 1.47173703 sec
D13 0.00000400 sec
D16 0.00020000 sec
IN0 0.00020160 sec
======== CHANNEL f1 ========
NUC1 1H
P0 11.60 usec
P1 11.60 usec
PL1 3.00 dB
SFO1 400.1331720 MHz
====== GRADIENT CHANNEL =====
GPNAM1 SINE.100
GPZ1 10.00 %
P16 1000.00 usec
ND0 1
TD 128
SFO1 400.1332 MHz
FIDRES 38.752480 Hz
SW 12.397 ppm
FnMODE QF
SI 1024
SF 400.1300000 MHz
WDW SINE
SSB 0
LB 0.00 Hz
GB 0
PC 1.40
SI 1024
MC2 QF
SF 400.1300000 MHz
WDW SINE
SSB 0
LB 0.00 Hz
GB 0

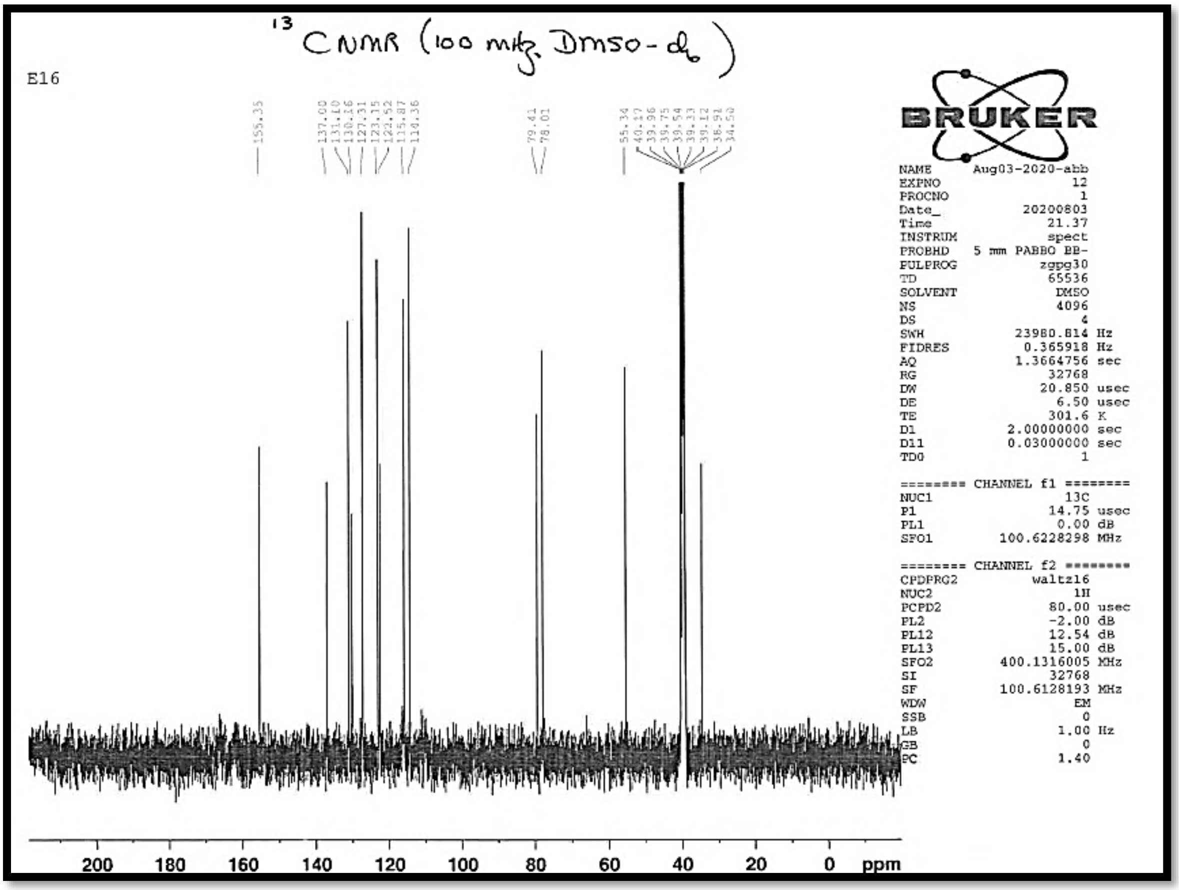
13C NMR (100 mHz, DMSO-d6)
E16
155.35
137.00
131.10
130.16
127.31
123.15
122.52
115.87
114.36
79.41
78.01
55.34
40.17
39.96
39.75
39.54
39.33
39.12
38.91
34.50
BRUKER
NAME Aug03-2020-abb
EXPNO 12
PROCNO 1
Date_ 20200803
Time 21.37
INSTRUM spect
PROBHD 5 mm PABBO BB-
PULPROG zgpg30
TD 65536
SOLVENT DMSO
NS 4096
DS 4
SWH 23980.814 Hz
FIDRES 0.365918 Hz
AQ 1.3664756 sec
RG 32768
DW 20.850 usec
DE 6.50 usec
TE 301.6 K
D1 2.00000000 sec
D11 0.03000000 sec
TD0 1
======== CHANNEL f1 ========
NUC1 13C
P1 14.75 usec
PL1 0.00 dB
SFO1 100.6228298 MHz
======== CHANNEL f2 ========
CPDPRG2 waltz16
NUC2 1H
PCPD2 80.00 usec
PL2 -2.00 dB
PL12 12.54 dB
PL13 15.00 dB
SFO2 400.1316005 MHz
SI 32768
SF 100.6128193 MHz
WDW EM
SSB 0
LB 1.00 Hz
GB 0
PC 1.40
200 180 160 140 120 100 80 60 40 20 0 ppm

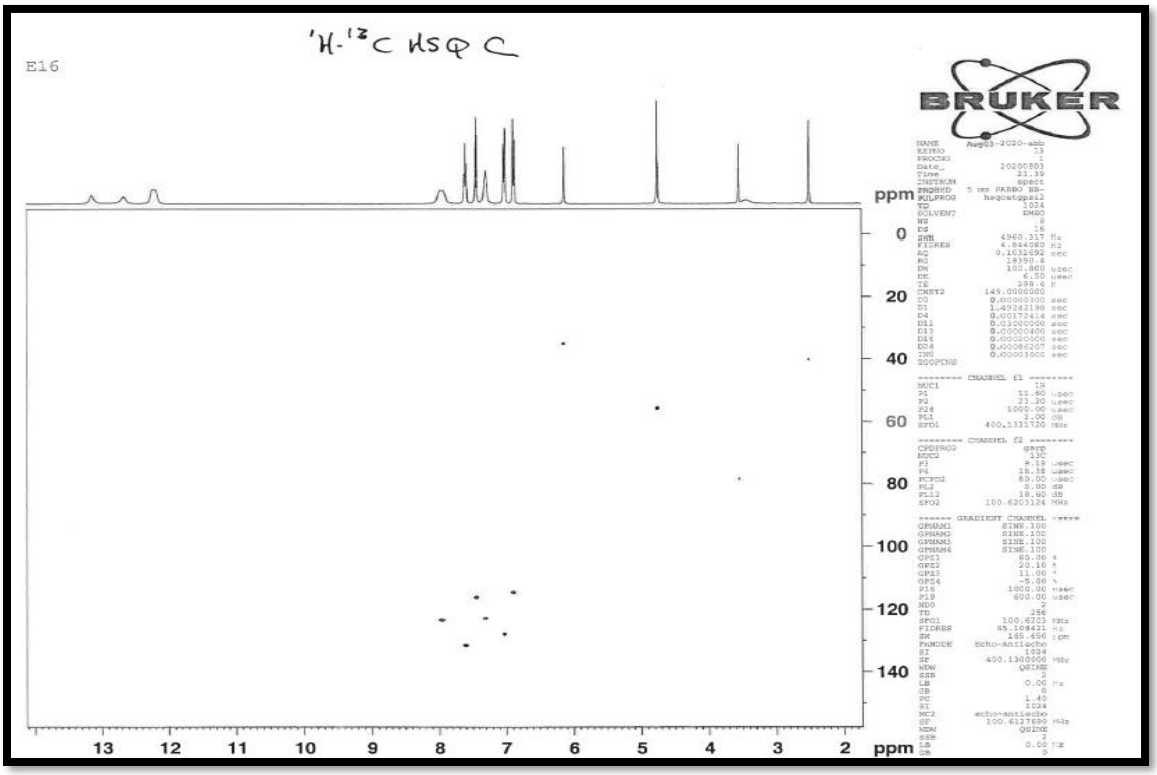
1H-13C HSQC
E16
BRUKER
ppm
0 20 40 60 80 100 120 140
13 12 11 10 9 8 7 6 5 4 3 2 ppm

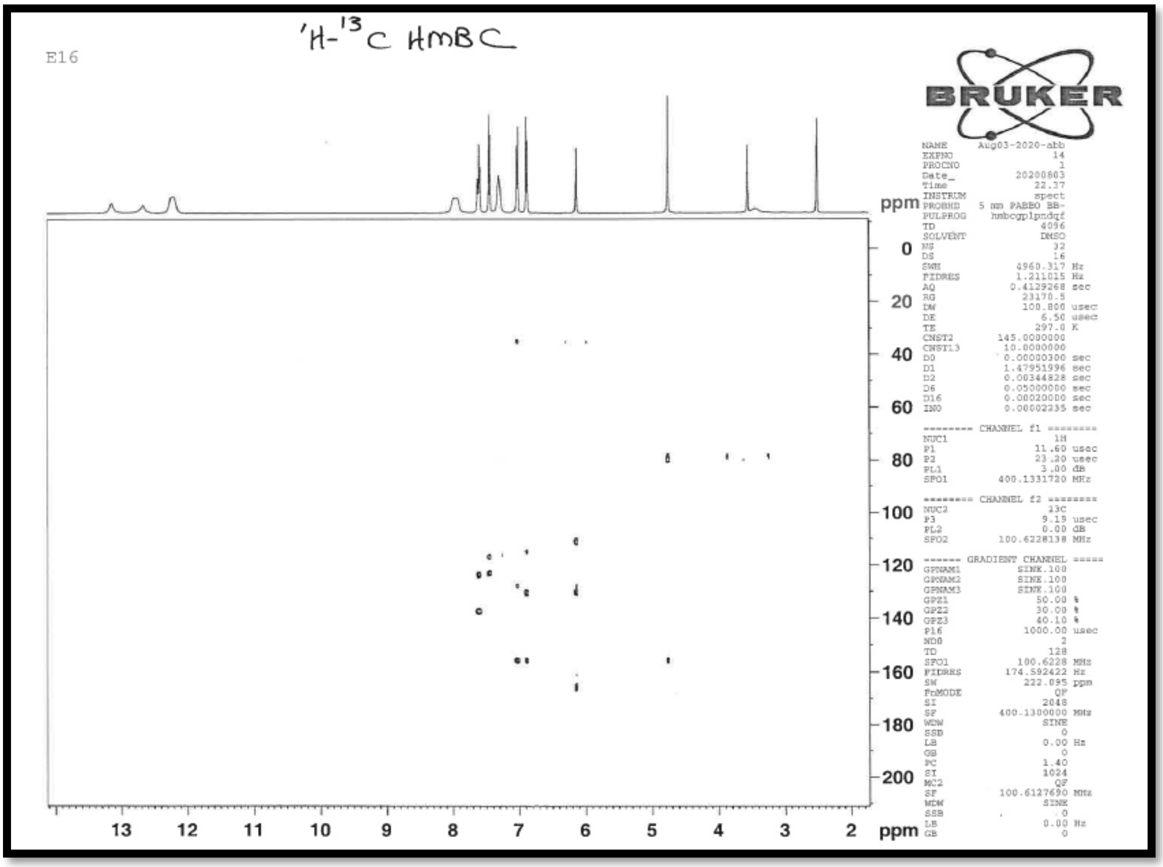

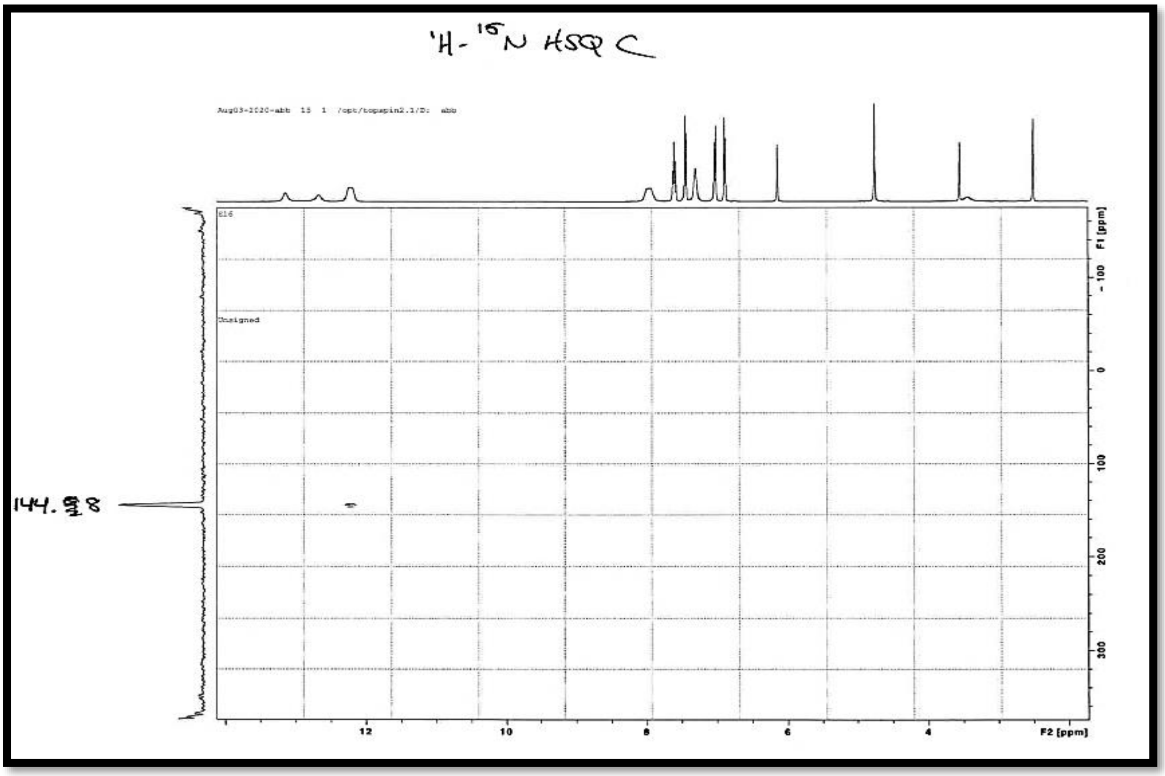

Spectrum data for Compound 4b

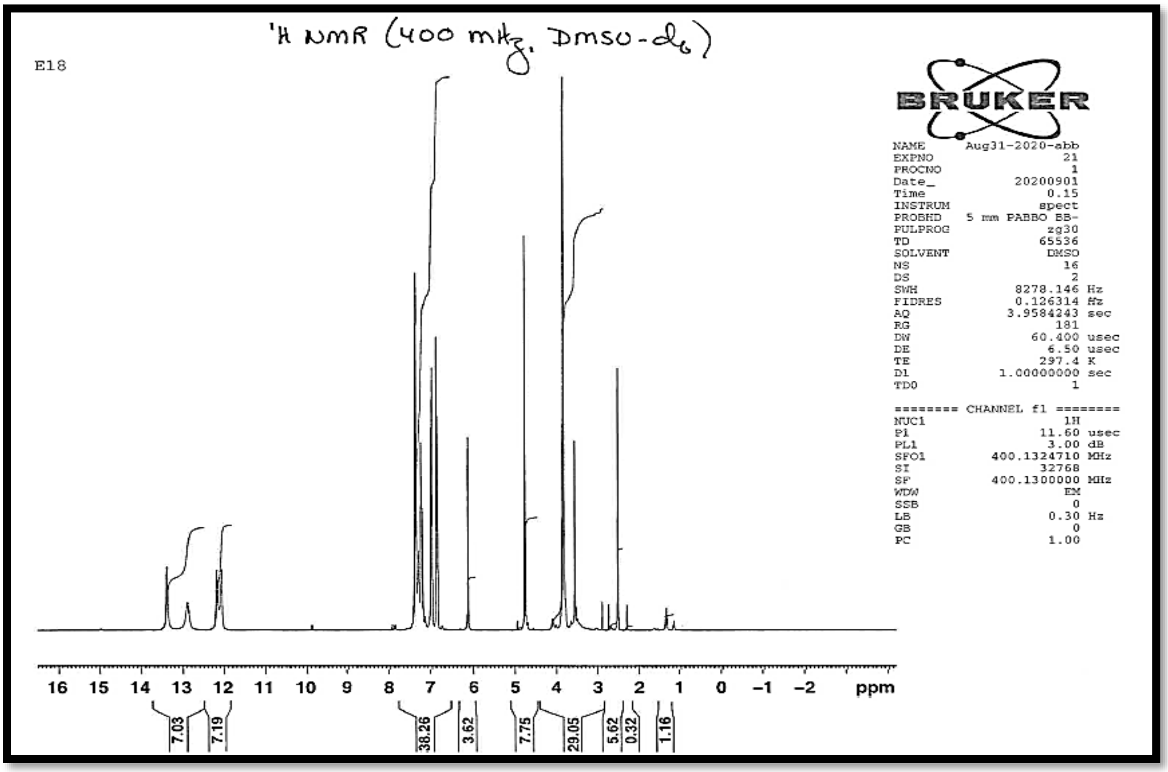

'H NMR (400 mHz, DMSO-d6)
E18
BRUKER
NAME Aug31-2020-abb
EXPNO 21
PROCNO 1
Date_ 20200901
Time 0.15
INSTRUM spect
PROBHD 5 mm PABBO BB-
PULPROG zg30
TD 65536
SOLVENT DMSO
NS 16
DS 2
SWH 8278.146 Hz
FIDRES 0.126314 Hz
AQ 3.9584243 sec
RG 181
DW 60.400 usec
DE 6.50 usec
TE 297.4 K
D1 1.00000000 sec
TD0 1
======== CHANNEL f1 ========
NUC1 1H
P1 11.60 usec
PL1 3.00 dB
SFO1 400.1324710 MHz
SI 32768
SF 400.1300000 MHz
WDW EM
SSB 0
LB 0.30 Hz
GB 0
PC 1.00
16 15 14 13 12 11 10 9 8 7 6 5 4 3 2 1 0 −1 −2 ppm
7.03
7.19
38.26
3.62
7.75
29.05
5.62
0.32
1.16

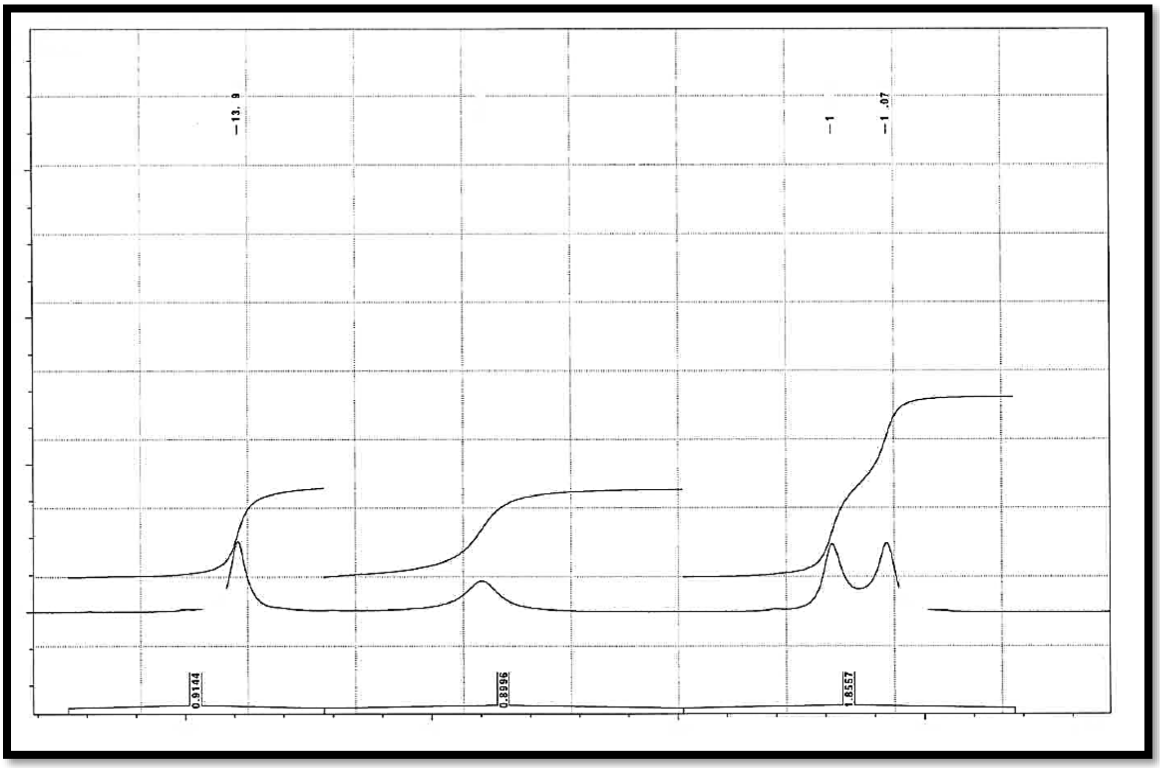

−13. 9
−1
−1 .07
0.9144
0.8996
1.8557

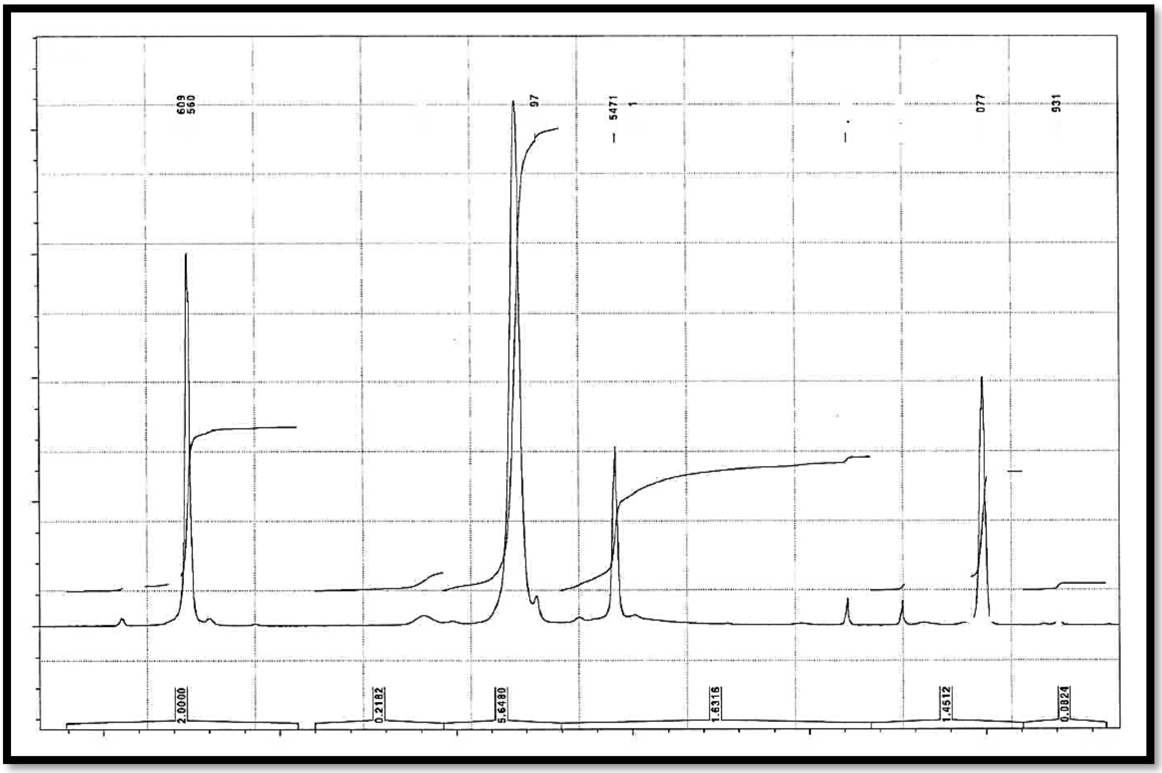

609
560
97
5471
1
077
931
2.0000
0.2182
5.6480
1.8316
1.4512
0.0824

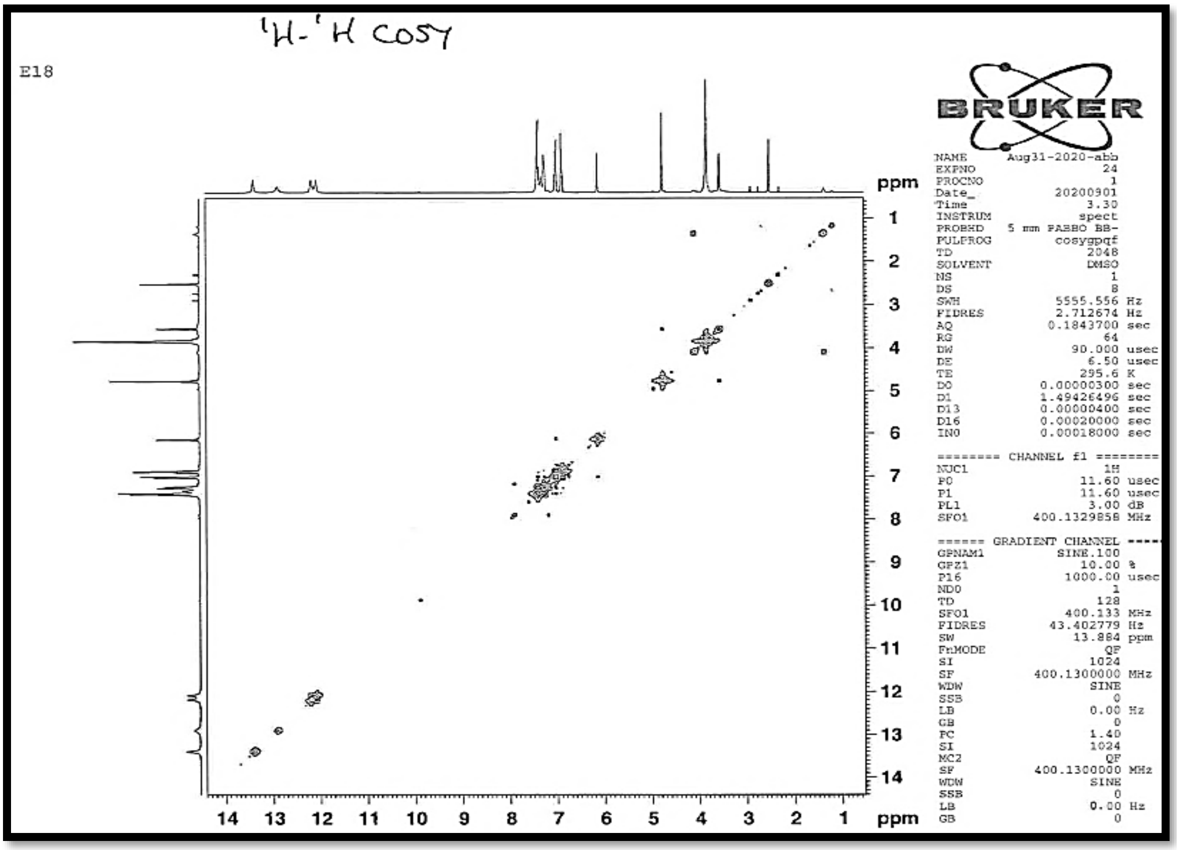

1H-1H COSY
E18
ppm
14 13 12 11 10 9 8 7 6 5 4 3 2 1 ppm
1 2 3 4 5 6 7 8 9 10 11 12 13 14
BRUKER
NAME Aug31-2020-abb
EXPNO 24
PROCNO 1
Date_ 20200901
Time 3.30
INSTRUM spect
PROBHD 5 mm PABBO BB-
PULPROG cosygpqf
TD 2048
SOLVENT DMSO
NS 1
DS 8
SWH 5555.556 Hz
FIDRES 2.712674 Hz
AQ 0.1843700 sec
RG 64
DW 90.000 usec
DE 6.50 usec
TE 295.6 K
D0 0.00000300 sec
D1 1.49426496 sec
D13 0.00000400 sec
D16 0.00020000 sec
IN0 0.00018000 sec
======== CHANNEL f1 ========
NUC1 1H
P0 11.60 usec
P1 11.60 usec
PL1 3.00 dB
SFO1 400.1329858 MHz
====== GRADIENT CHANNEL -----
GPNAM1 SINE.100
GPZ1 10.00 %
P16 1000.00 usec
ND0 1
TD 128
SFO1 400.133 MHz
FIDRES 43.402779 Hz
SW 13.884 ppm
FnMODE QF
SI 1024
SF 400.1300000 MHz
WDW SINE
SSB 0
LB 0.00 Hz
GB 0
PC 1.40
SI 1024
MC2 QF
SF 400.1300000 MHz
WDW SINE
SSB 0
LB 0.00 Hz
GB 0

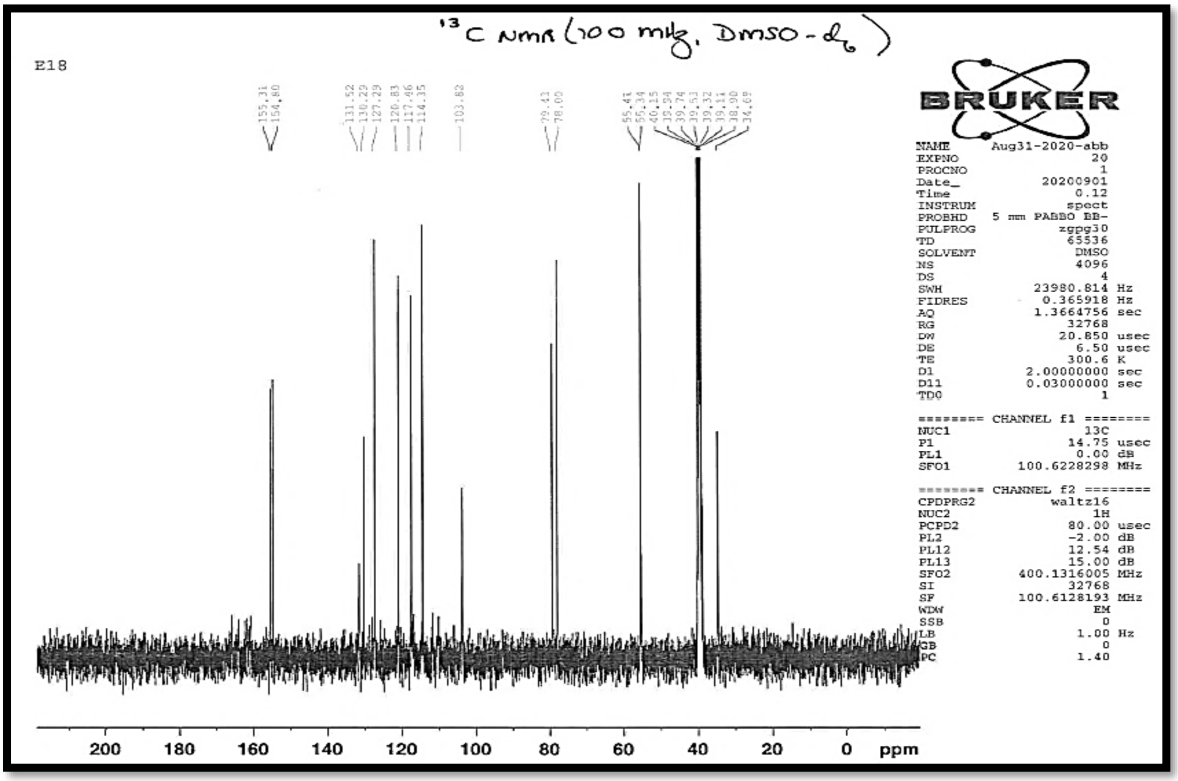
13C NMR (100 MHz, DMSO-d6)
E18
155.31
154.80
131.52
130.29
127.29
120.83
117.46
114.35
103.82
79.43
78.00
55.43
55.34
BRUKER
NAME Aug31-2020-abb
EXPNO 20
PROCNO 1
Date_ 20200901
Time 0.12
INSTRUM spect
PROBHD 5 mm PABBO BB-
PULPROG zgpg30
TD 65536
SOLVENT DMSO
NS 4096
DS 4
SWH 23980.814 Hz
FIDRES 0.365918 Hz
AQ 1.3664756 sec
RG 32768
DW 20.850 usec
DE 6.50 usec
TE 300.6 K
D1 2.00000000 sec
D11 0.03000000 sec
TD0 1
======== CHANNEL f1 ========
NUC1 13C
P1 14.75 usec
PL1 0.00 dB
SFO1 100.6228298 MHz
======== CHANNEL f2 ========
CPDPRG2 waltz16
NUC2 1H
PCPD2 80.00 usec
PL2 -2.00 dB
PL12 12.54 dB
PL13 15.00 dB
SFO2 400.1316005 MHz
SI 32768
SF 100.6128193 MHz
WDW EM
SSB 0
LB 1.00 Hz
GB 0
PC 1.40
200
180
160
140
120
100
80
60
40
20
0
ppm

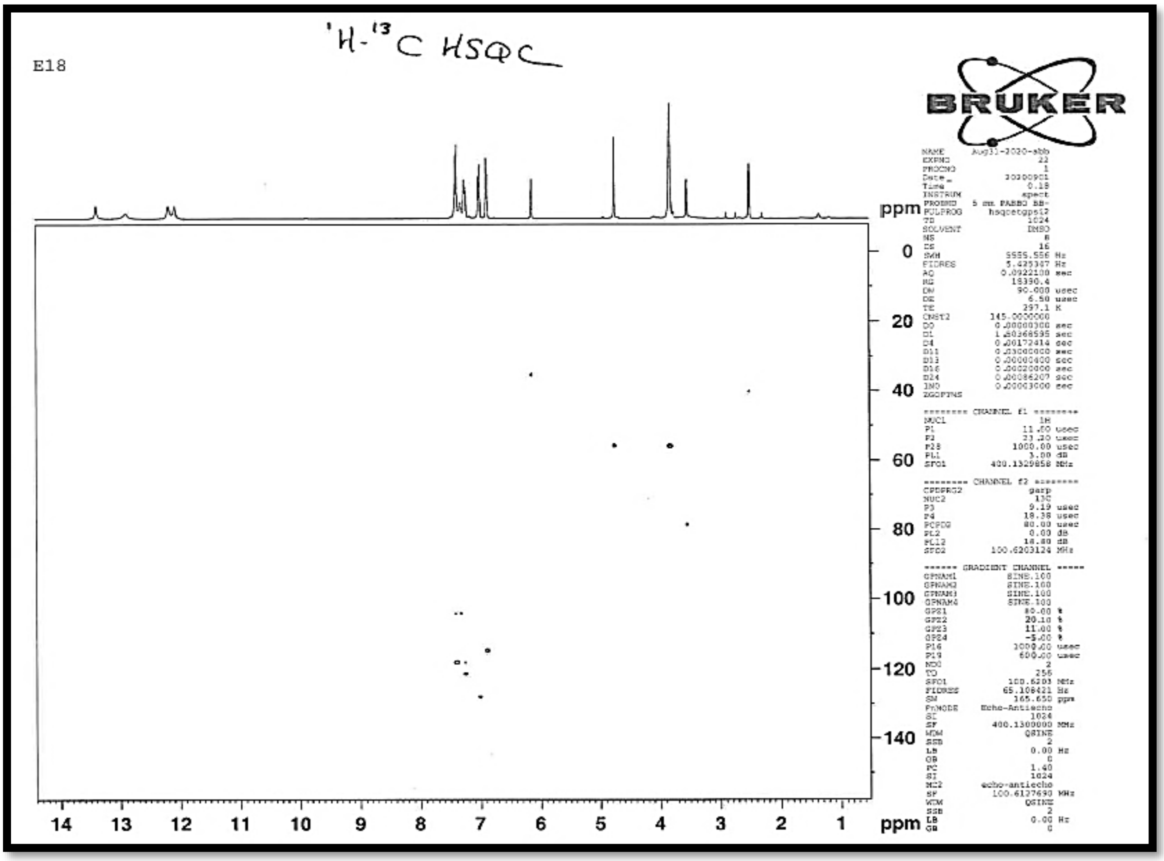
1H-13C HSQC
E18
BRUKER
ppm
0
20
40
60
80
100
120
140
14
13
12
11
10
9
8
7
6
5
4
3
2
1
ppm

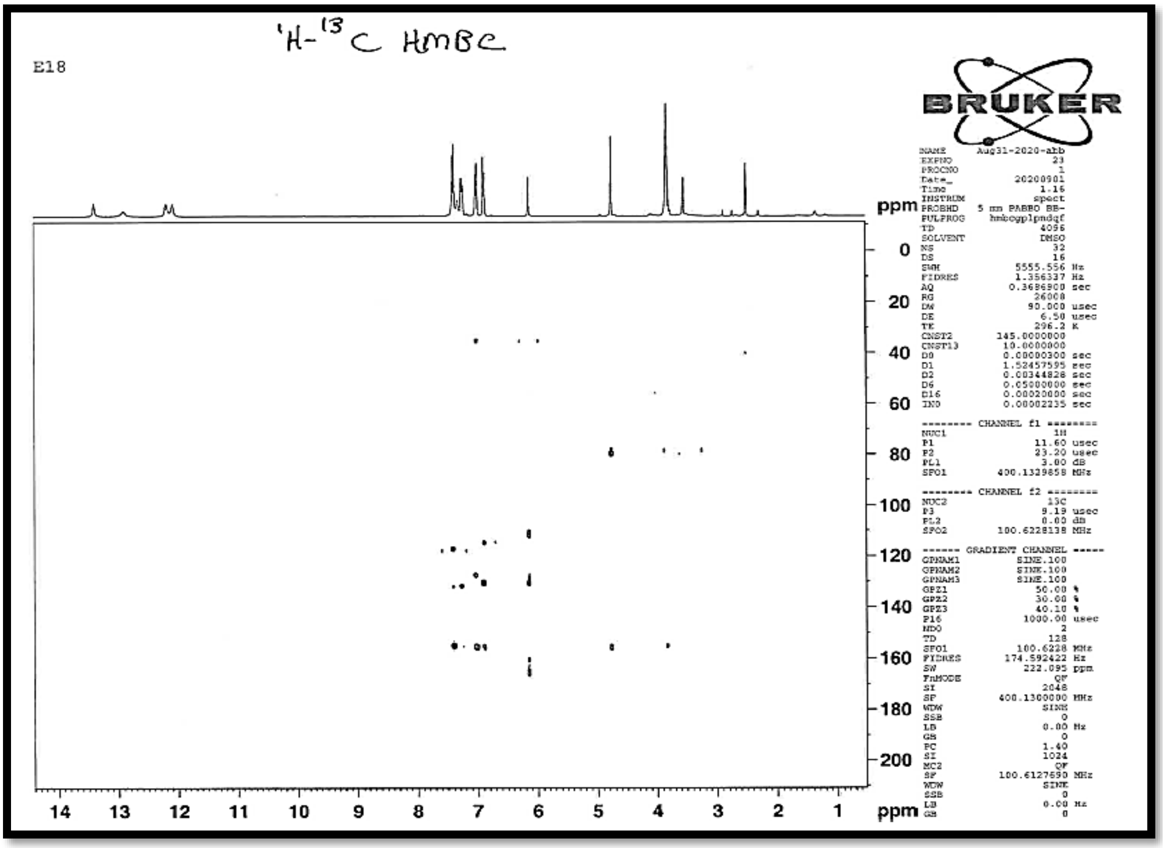

'H-13C HMBC
E18
BRUKER
ppm
0
20
40
60
80
100
120
140
160
180
200
14 13 12 11 10 9 8 7 6 5 4 3 2 1 ppm

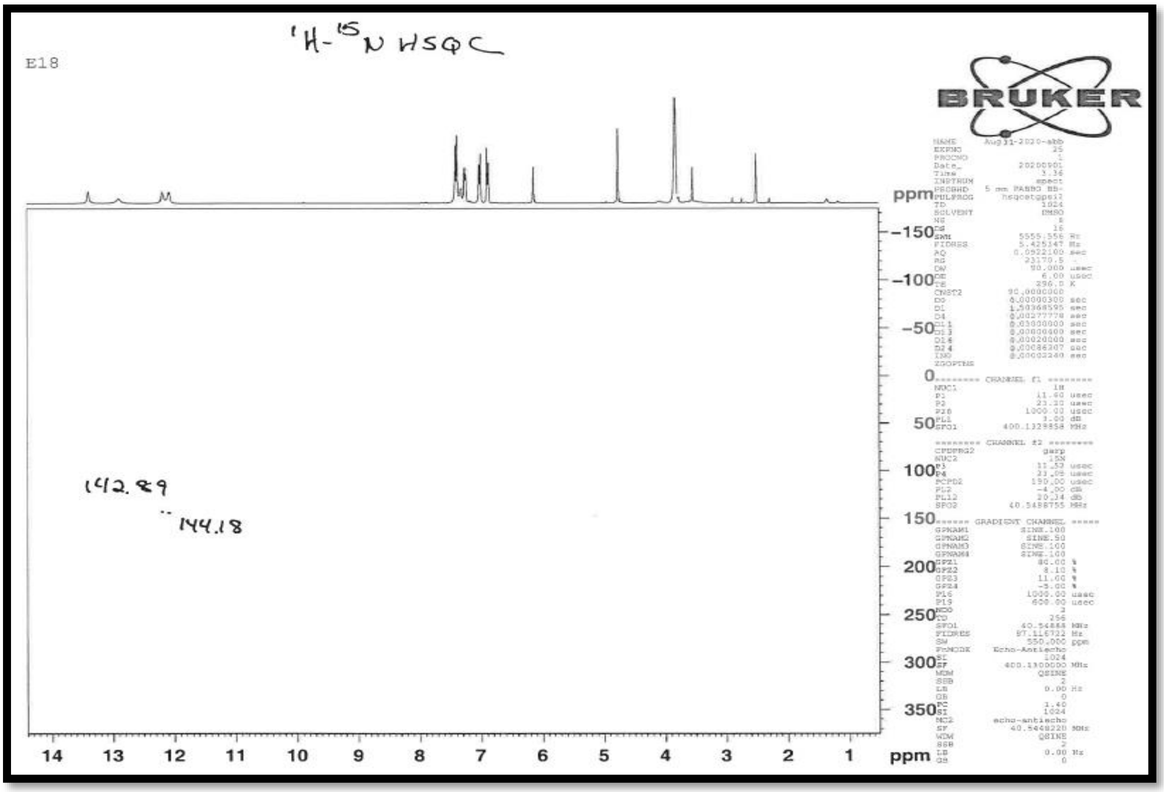

'H-15N HSQC
E18
BRUKER
ppm
-150
-100
-50
0
50
100
150
200
250
300
350
142.89
144.18
14 13 12 11 10 9 8 7 6 5 4 3 2 1 ppm

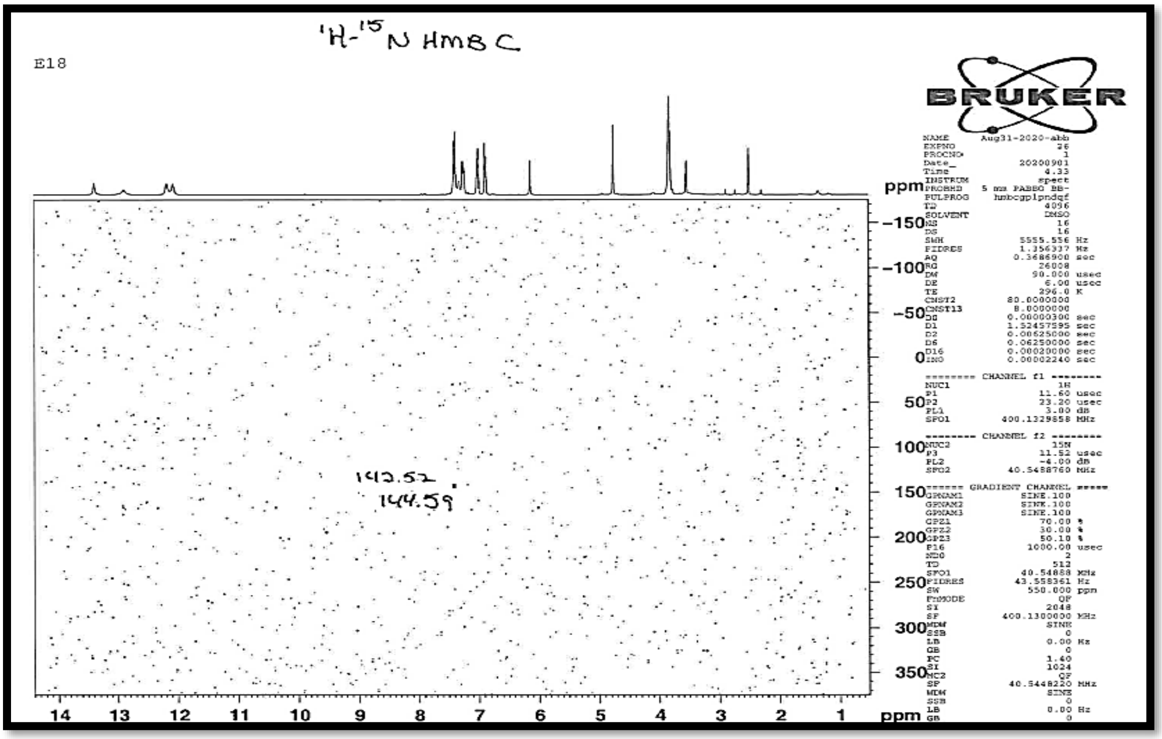

Spectrum data for Compound 4c

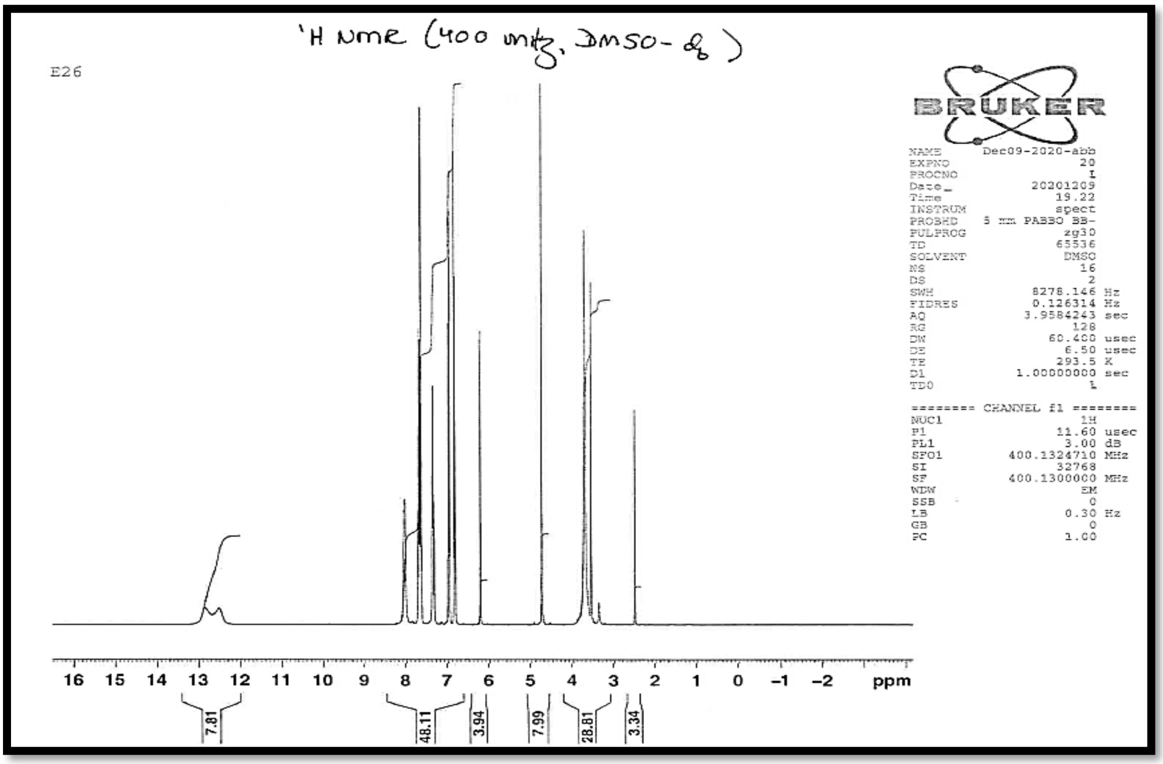

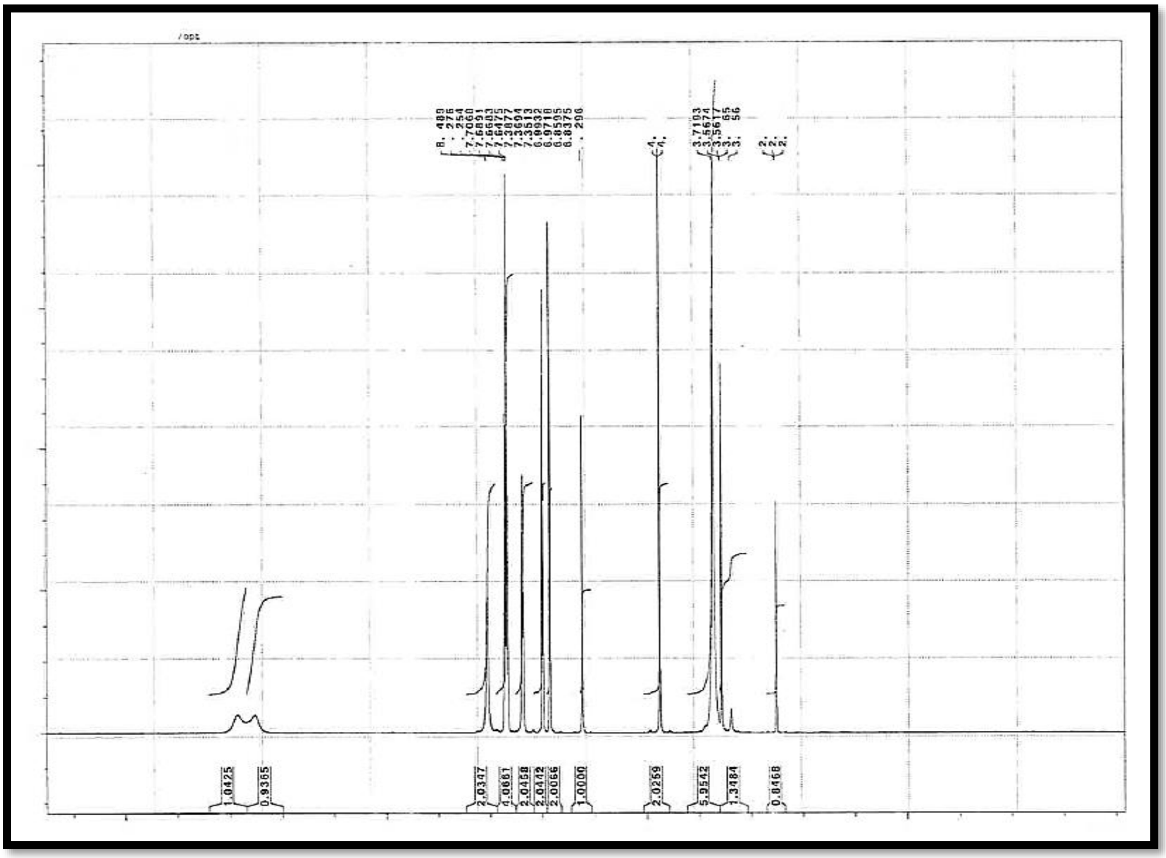
/opt
1.0425
0.9365
2.0347
4.0661
2.0458
2.0442
2.0066
1.0000
2.0259
5.9542
1.3484
0.8468

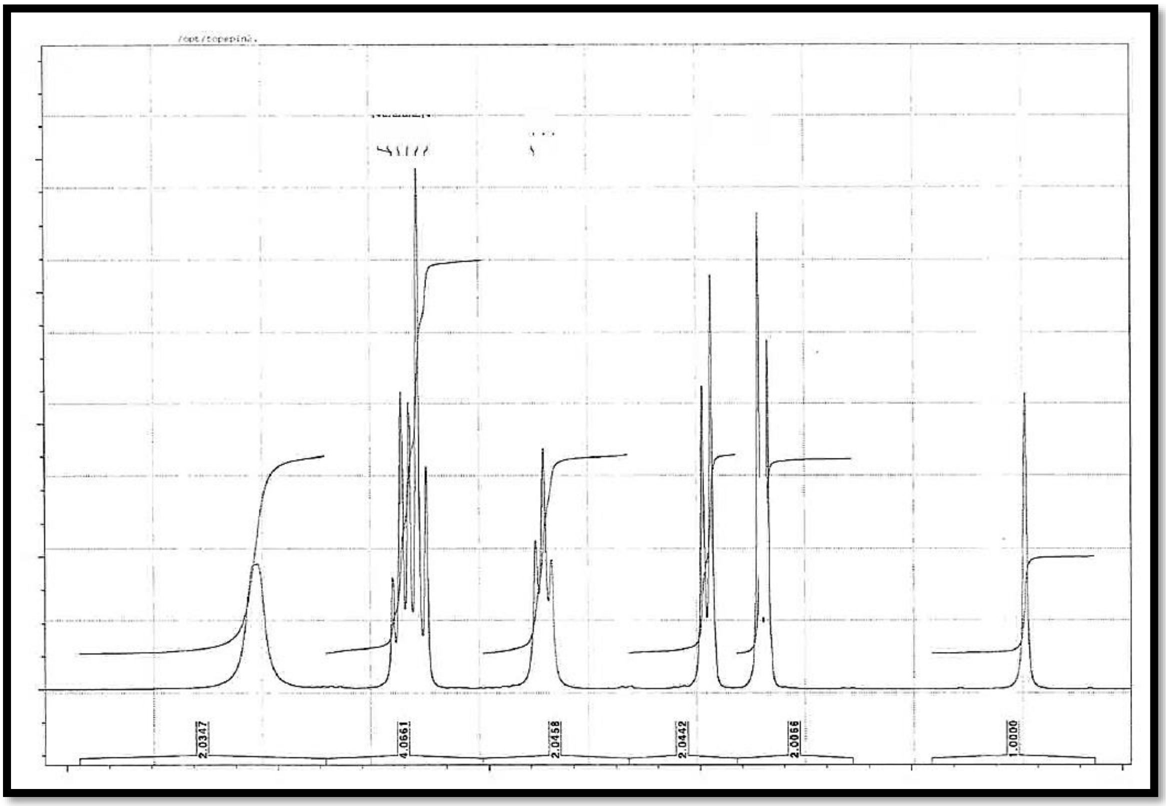
/opt/topspin2.
2.0347
4.0661
2.0458
2.0442
2.0066
1.0000

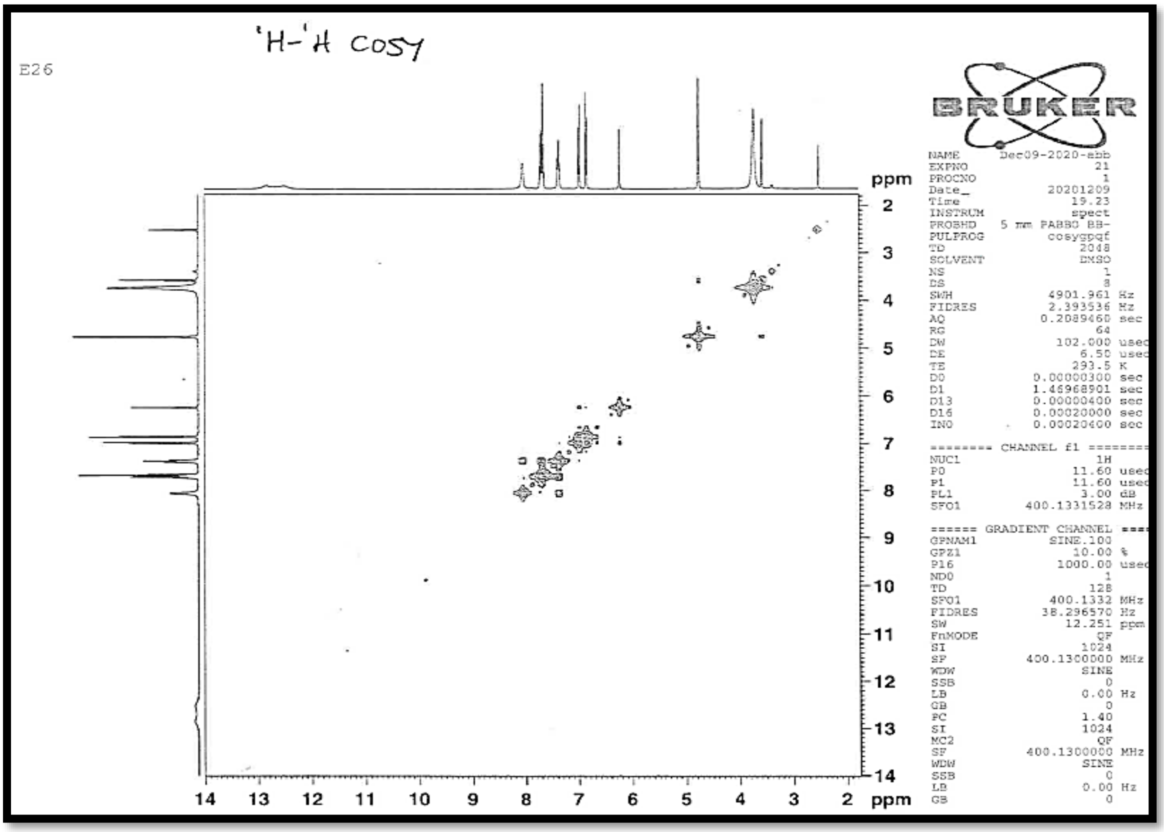

'H-'H COSY
E26
BRUKER
NAME Dec09-2020-abb
EXPNO 21
PROCNO 1
Date_ 20201209
Time 19.23
INSTRUM spect
PROBHD 5 mm PABBO BB-
PULPROG cosygpqf
TD 2048
SOLVENT DMSO
NS 1
DS 8
SWH 4901.961 Hz
FIDRES 2.393536 Hz
AQ 0.2089460 sec
RG 64
DW 102.000 usec
DE 6.50 usec
TE 293.5 K
D0 0.00000300 sec
D1 1.46968901 sec
D13 0.00000400 sec
D16 0.00020000 sec
IN0 0.00020400 sec
======== CHANNEL f1 ========
NUC1 1H
P0 11.60 usec
P1 11.60 usec
PL1 3.00 dB
SFO1 400.1331528 MHz
====== GRADIENT CHANNEL =====
GPNAM1 SINE.100
GPZ1 10.00 %
P16 1000.00 usec
ND0 1
TD 128
SFO1 400.1332 MHz
FIDRES 38.296570 Hz
SW 12.251 ppm
FnMODE QF
SI 1024
SF 400.1300000 MHz
WDW SINE
SSB 0
LB 0.00 Hz
GB 0
PC 1.40
SI 1024
MC2 QF
SF 400.1300000 MHz
WDW SINE
SSB 0
LB 0.00 Hz
GB 0
ppm

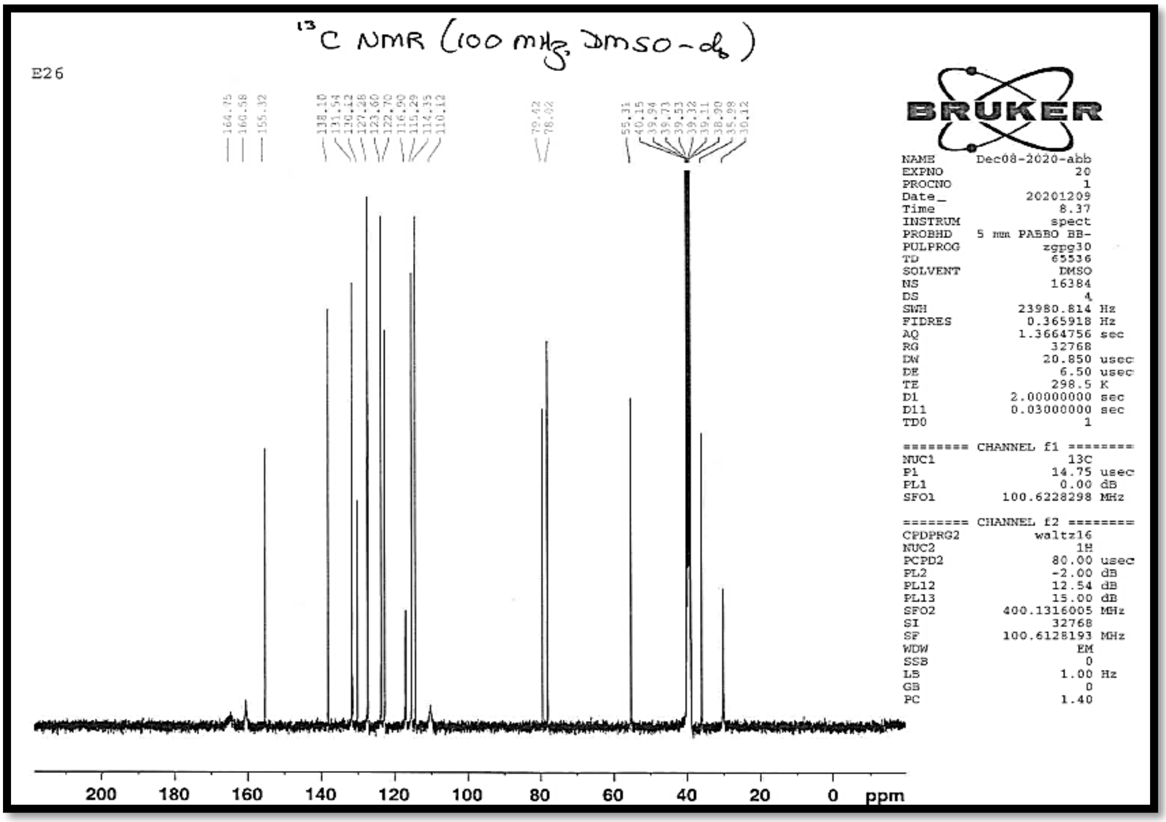

13C NMR (100 mHz, DMSO-d6)
E26
164.75
160.58
155.32
138.10
131.54
130.12
127.86
123.60
122.70
116.90
115.29
114.35
110.12
79.42
78.02
55.31
40.15
39.94
39.73
39.53
39.32
39.11
38.90
35.99
30.12
BRUKER
NAME Dec08-2020-abb
EXPNO 20
PROCNO 1
Date_ 20201209
Time 8.37
INSTRUM spect
PROBHD 5 mm PABBO BB-
PULPROG zgpg30
TD 65536
SOLVENT DMSO
NS 16384
DS 4
SWH 23980.814 Hz
FIDRES 0.365918 Hz
AQ 1.3664756 sec
RG 32768
DW 20.850 usec
DE 6.50 usec
TE 298.5 K
D1 2.00000000 sec
D11 0.03000000 sec
TD0 1
======== CHANNEL f1 ========
NUC1 13C
P1 14.75 usec
PL1 0.00 dB
SFO1 100.6228298 MHz
======== CHANNEL f2 ========
CPDPRG2 waltz16
NUC2 1H
PCPD2 80.00 usec
PL2 -2.00 dB
PL12 12.54 dB
PL13 15.00 dB
SFO2 400.1316005 MHz
SI 32768
SF 100.6128193 MHz
WDW EM
SSB 0
LB 1.00 Hz
GB 0
PC 1.40
ppm

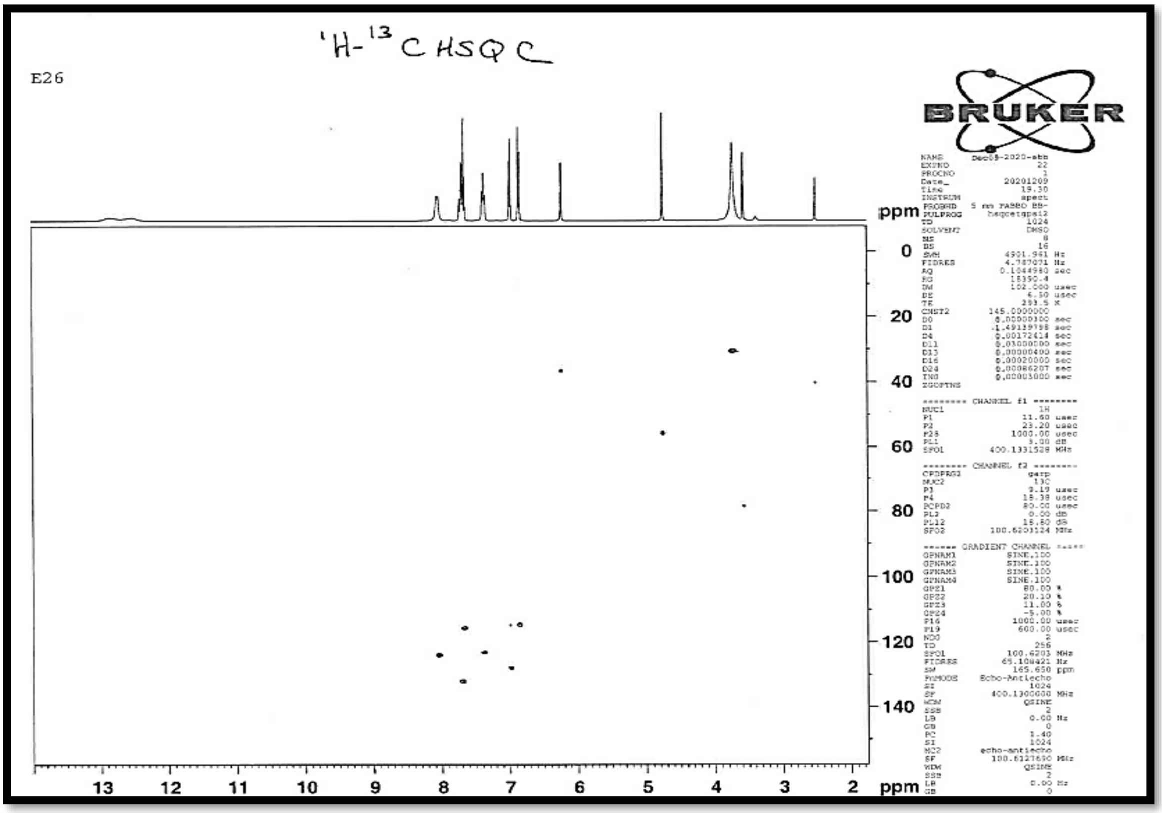

'H-13C HSQC
E26
ppm
0
20
40
60
80
100
120
140
13 12 11 10 9 8 7 6 5 4 3 2 ppm

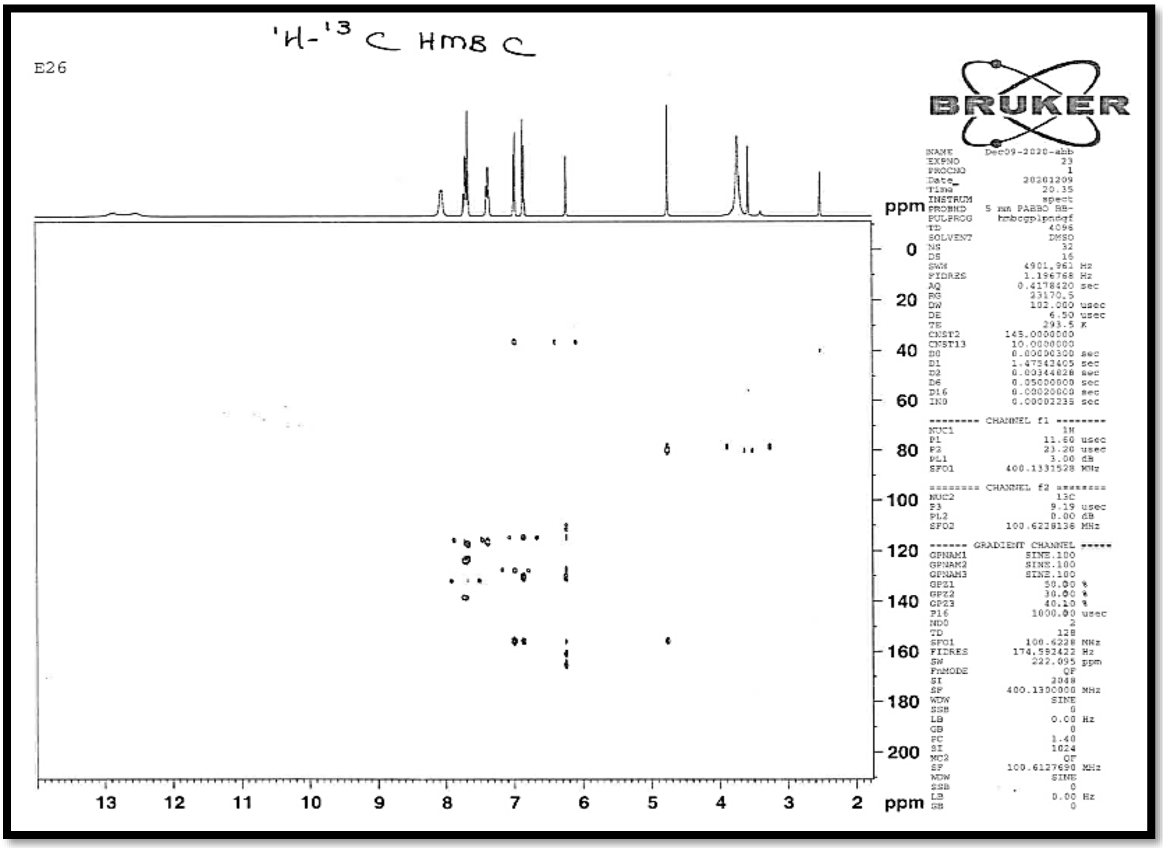

'H-13C HMBC
E26
ppm
0
20
40
60
80
100
120
140
160
180
200
13 12 11 10 9 8 7 6 5 4 3 2 ppm

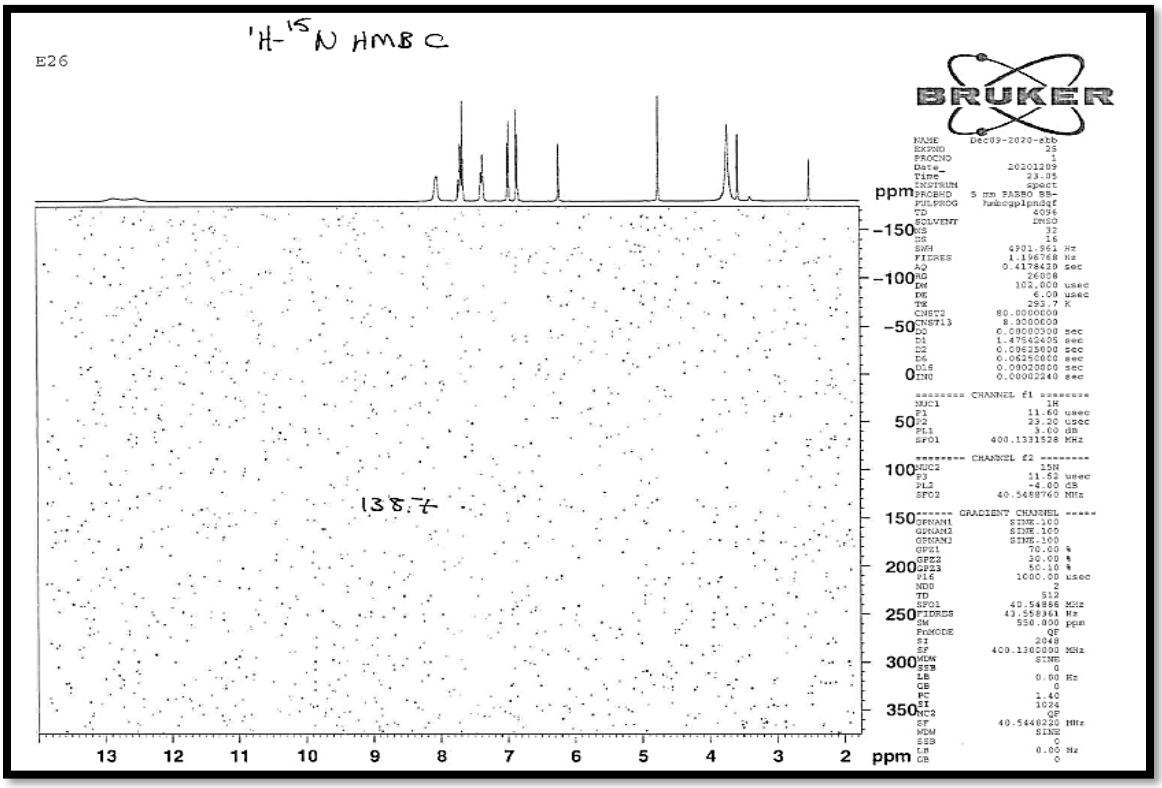

Spectrum data for Compound 4d

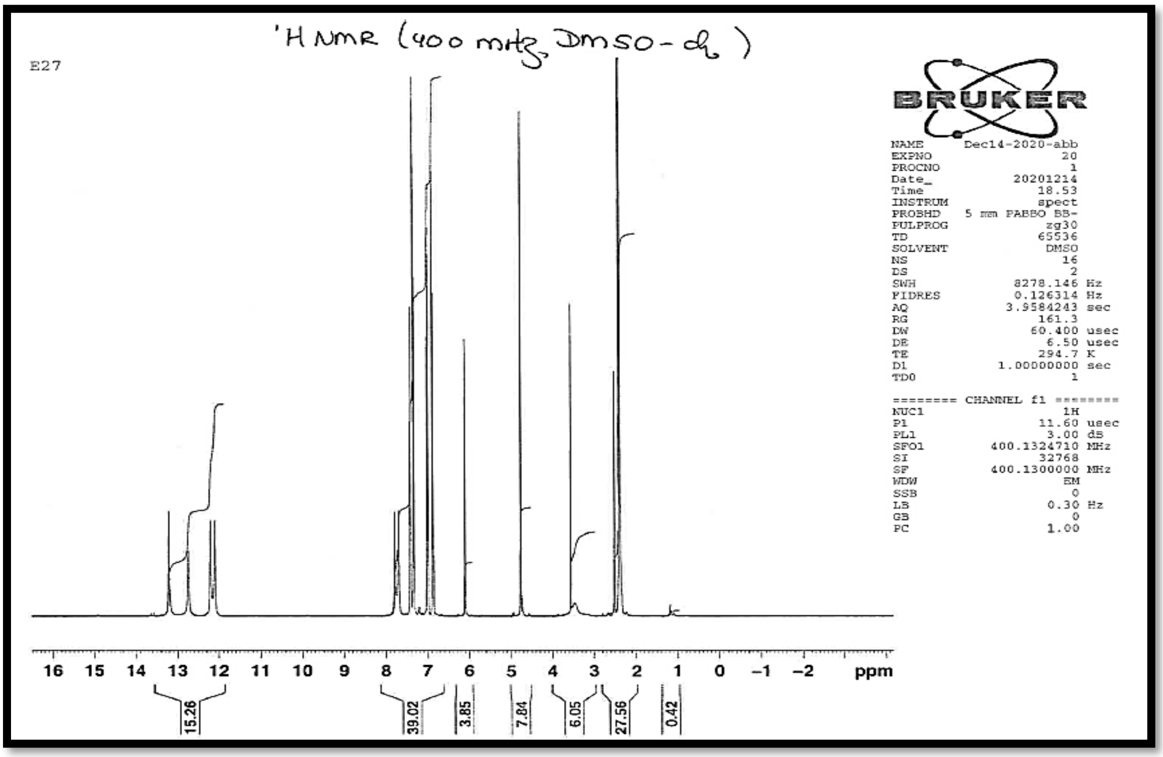

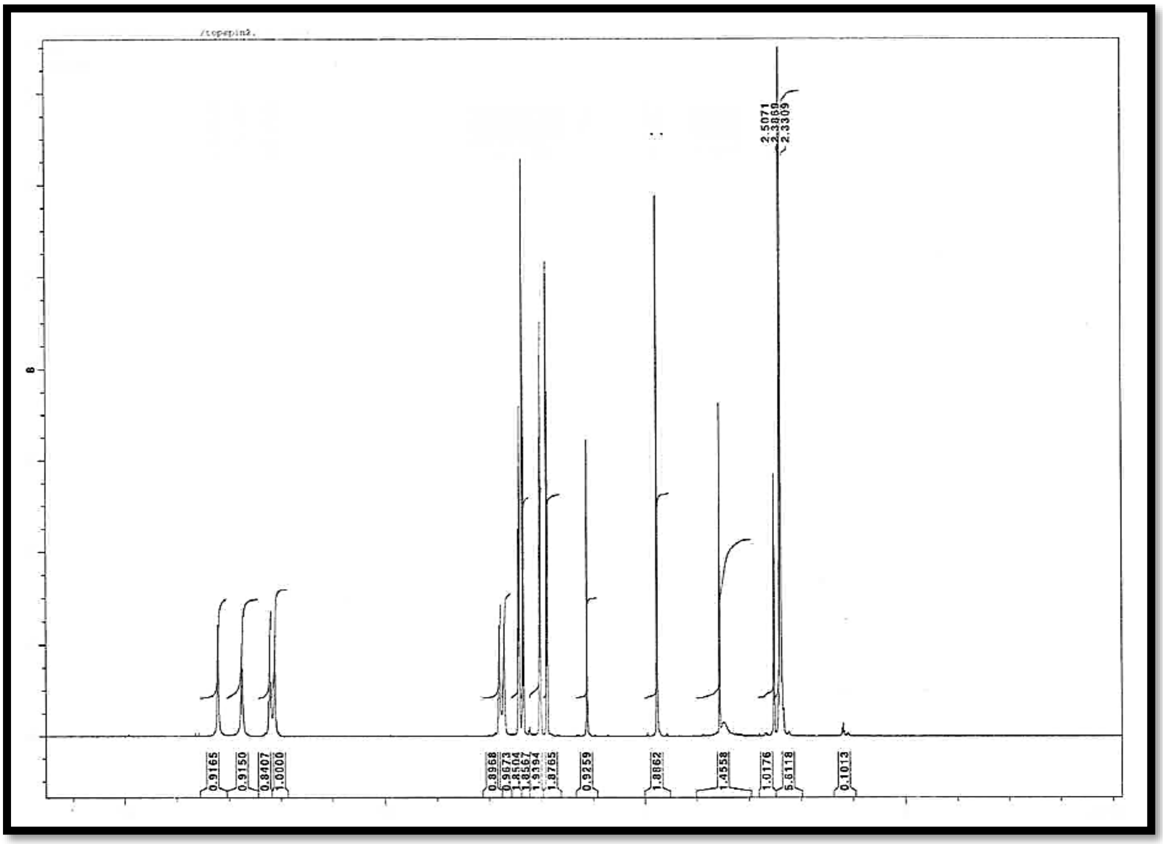
2.5071
2.3309
0.9165
0.9150
0.8407
1.0000
0.8968
0.9673
1.8567
1.9394
1.8765
0.9259
1.8862
1.4558
1.0176
5.6118
0.1013

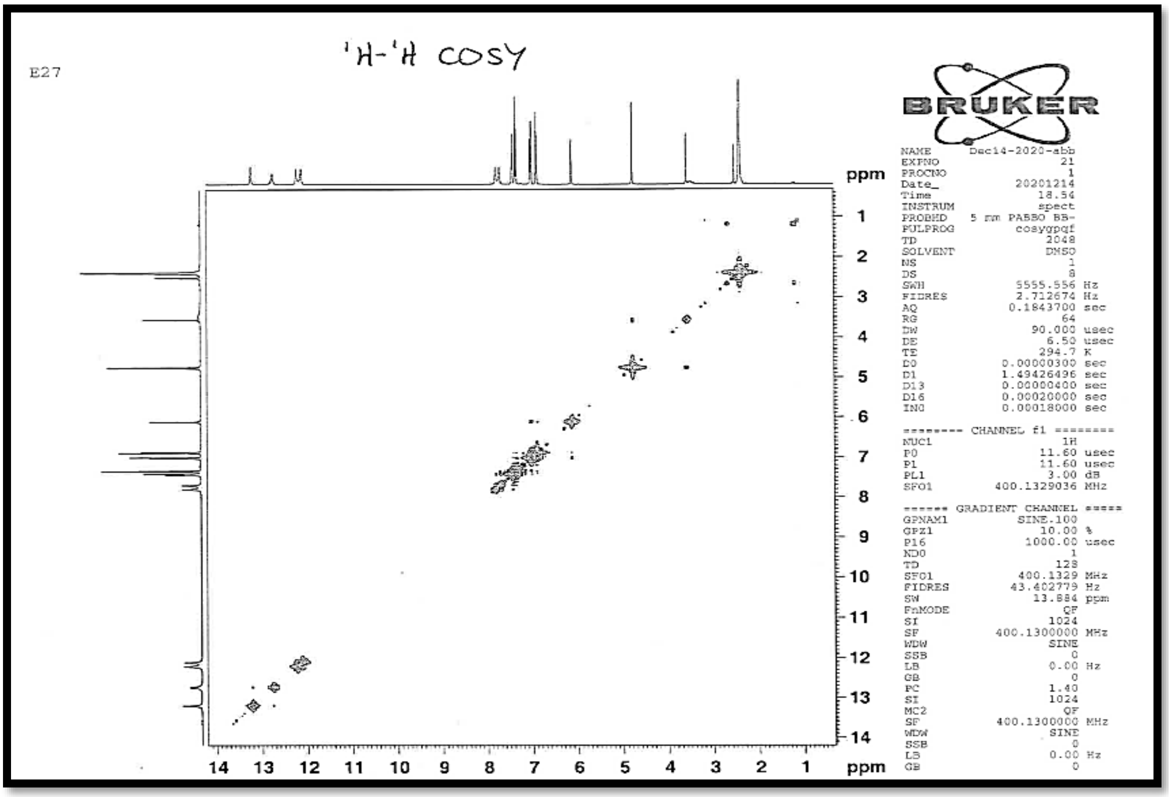
E27
¹H-¹H COSY
BRUKER
ppm
14 13 12 11 10 9 8 7 6 5 4 3 2 1 ppm
NAME Dec14-2020-abb
EXPNO 21
PROCNO 1
Date_ 20201214
Time 18.54
INSTRUM spect
PROBHD 5 mm PABBO BB-
PULPROG cosygpqf
TD 2048
SOLVENT DMSO
NS 1
DS 8
SWH 5555.556 Hz
FIDRES 2.712674 Hz
AQ 0.1843700 sec
RG 64
DW 90.000 usec
DE 6.50 usec
TE 294.7 K
D0 0.00000300 sec
D1 1.49426496 sec
D13 0.00000400 sec
D16 0.00020000 sec
IN0 0.00018000 sec
======== CHANNEL f1 ========
NUC1 1H
P0 11.60 usec
P1 11.60 usec
PL1 3.00 dB
SFO1 400.1329036 MHz
====== GRADIENT CHANNEL =====
GPNAM1 SINE.100
GPZ1 10.00 %
P16 1000.00 usec
ND0 1
TD 128
SFO1 400.1329 MHz
FIDRES 43.402779 Hz
SW 13.884 ppm
FnMODE QF
SI 1024
SF 400.1300000 MHz
WDW SINE
SSB 0
LB 0.00 Hz
GB 0
PC 1.40
SI 1024
MC2 QF
SF 400.1300000 MHz
WDW SINE
SSB 0
LB 0.00 Hz
GB 0

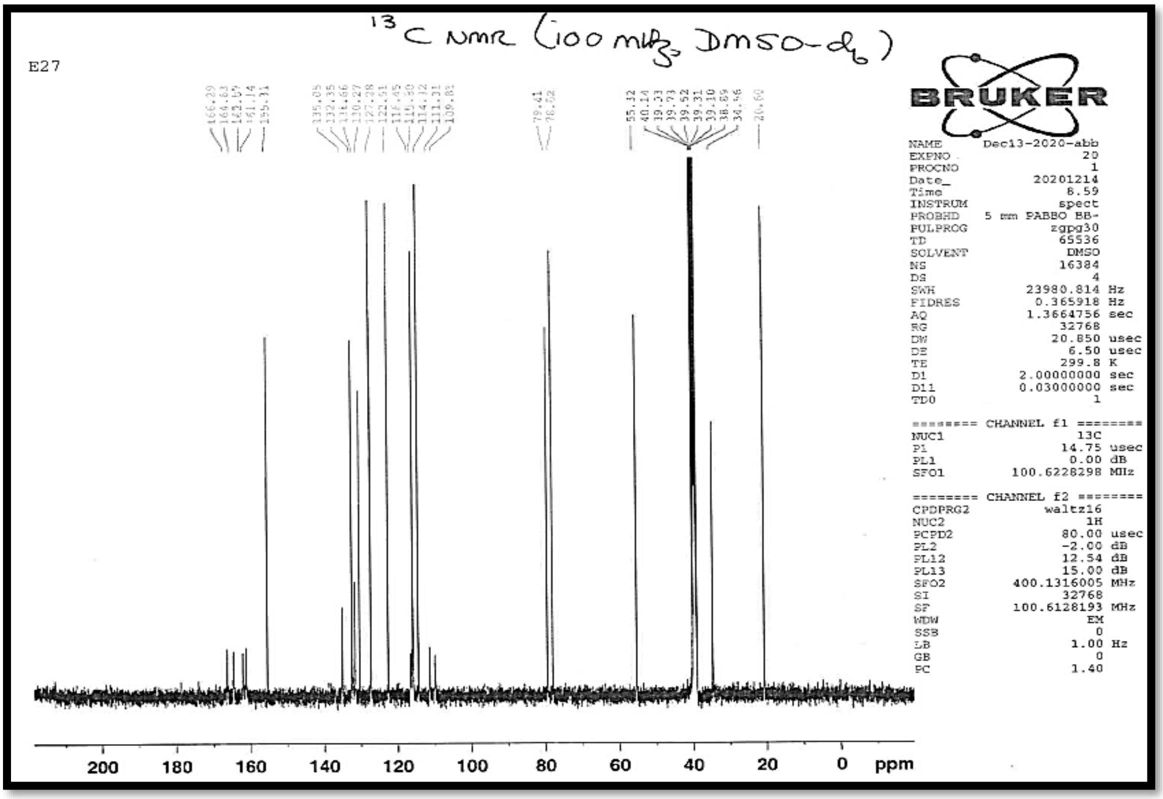
13C NMR (100 MHz, DMSO-d6)
E27
166.29
164.63
161.14
155.31
135.05
132.35
131.66
130.27
127.38
122.51
116.45
115.80
114.32
111.31
109.81
79.41
76.52
55.32
40.14
39.93
39.73
39.52
39.31
39.10
38.89
34.46
20.60
BRUKER
NAME Dec13-2020-abb
EXPNO 20
PROCNO 1
Date_ 20201214
Time 8.59
INSTRUM spect
PROBHD 5 mm PABBO BB-
PULPROG zgpg30
TD 65536
SOLVENT DMSO
NS 16384
DS 4
SWH 23980.814 Hz
FIDRES 0.365918 Hz
AQ 1.3664756 sec
RG 32768
DW 20.850 usec
DE 6.50 usec
TE 299.8 K
D1 2.00000000 sec
D11 0.03000000 sec
TD0 1
======== CHANNEL f1 ========
NUC1 13C
P1 14.75 usec
PL1 0.00 dB
SFO1 100.6228298 MHz
======== CHANNEL f2 ========
CPDPRG2 waltz16
NUC2 1H
PCPD2 80.00 usec
PL2 -2.00 dB
PL12 12.54 dB
PL13 15.00 dB
SFO2 400.1316005 MHz
SI 32768
SF 100.6128193 MHz
WDW EM
SSB 0
LB 1.00 Hz
GB 0
PC 1.40
200
180
160
140
120
100
80
60
40
20
0
ppm

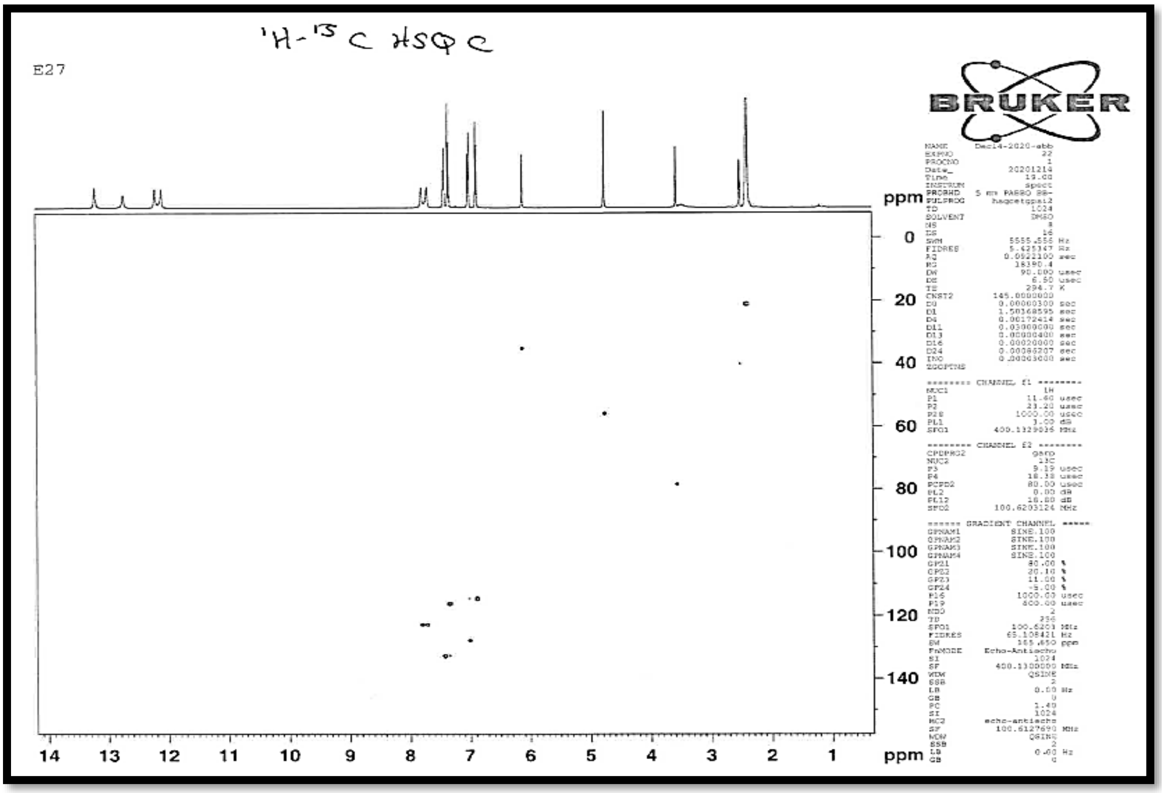
1H-13C HSQC
E27
BRUKER
ppm
0
20
40
60
80
100
120
140
14
13
12
11
10
9
8
7
6
5
4
3
2
1
ppm

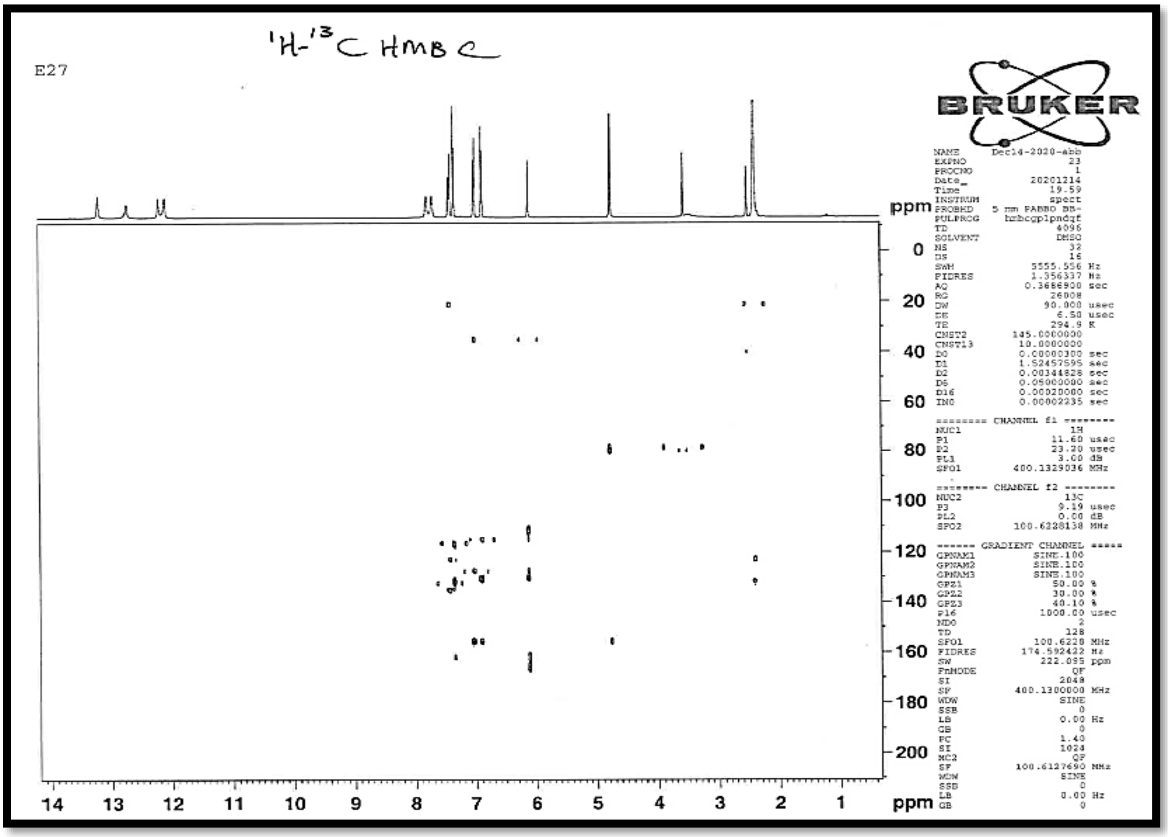
E27
1H-13C HMBC
BRUKER
ppm

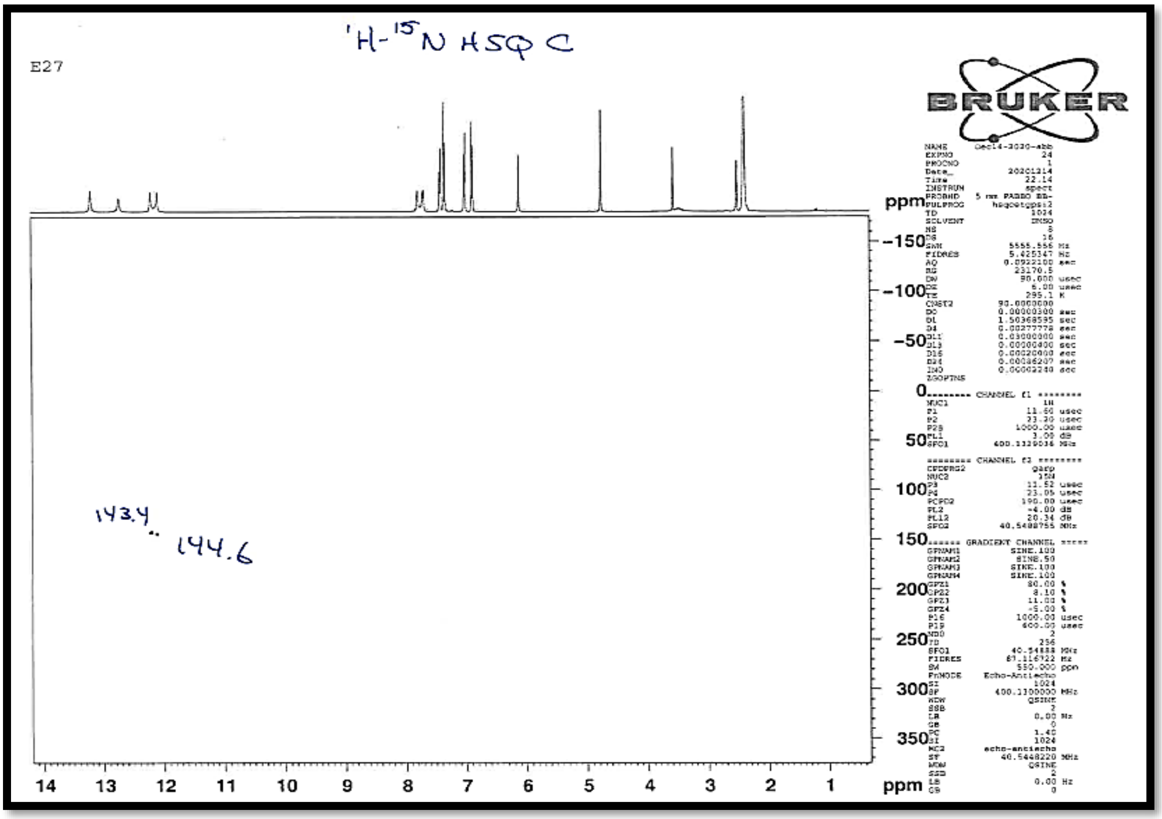
E27
1H-15N HSQC
BRUKER
143.4
144.6
ppm

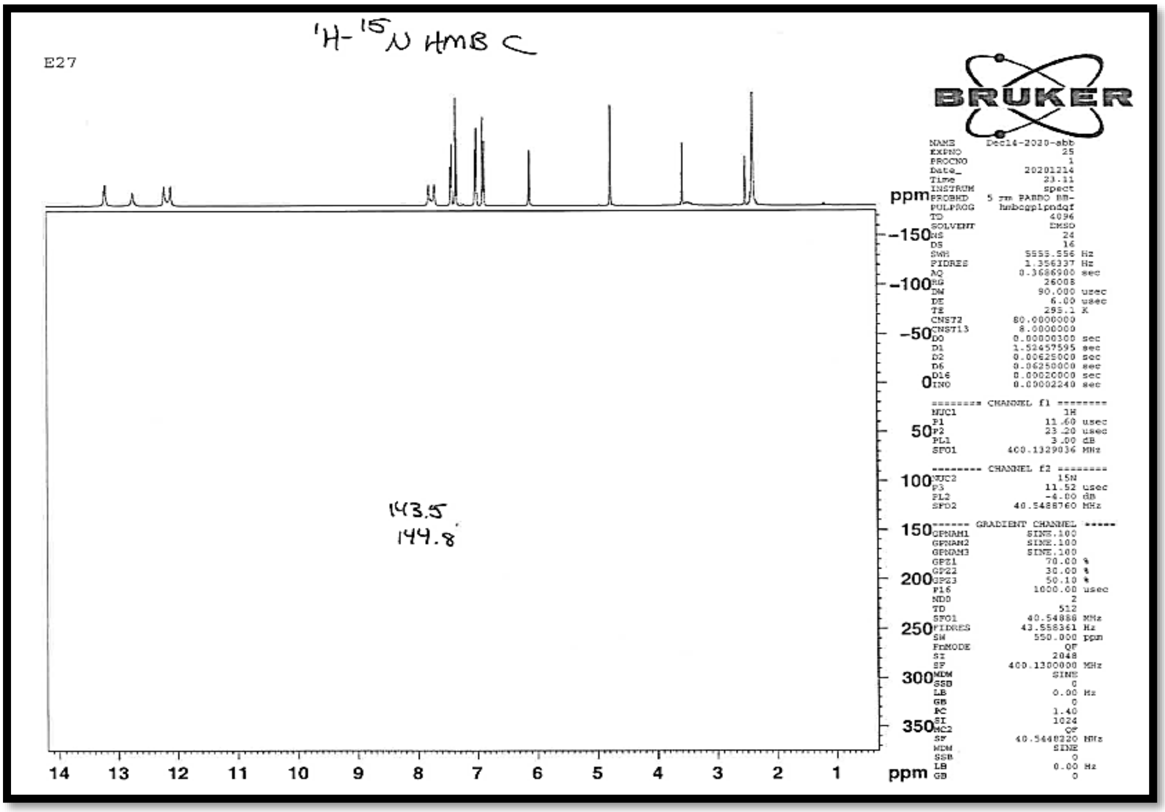

Spectrum data for Compound 8a

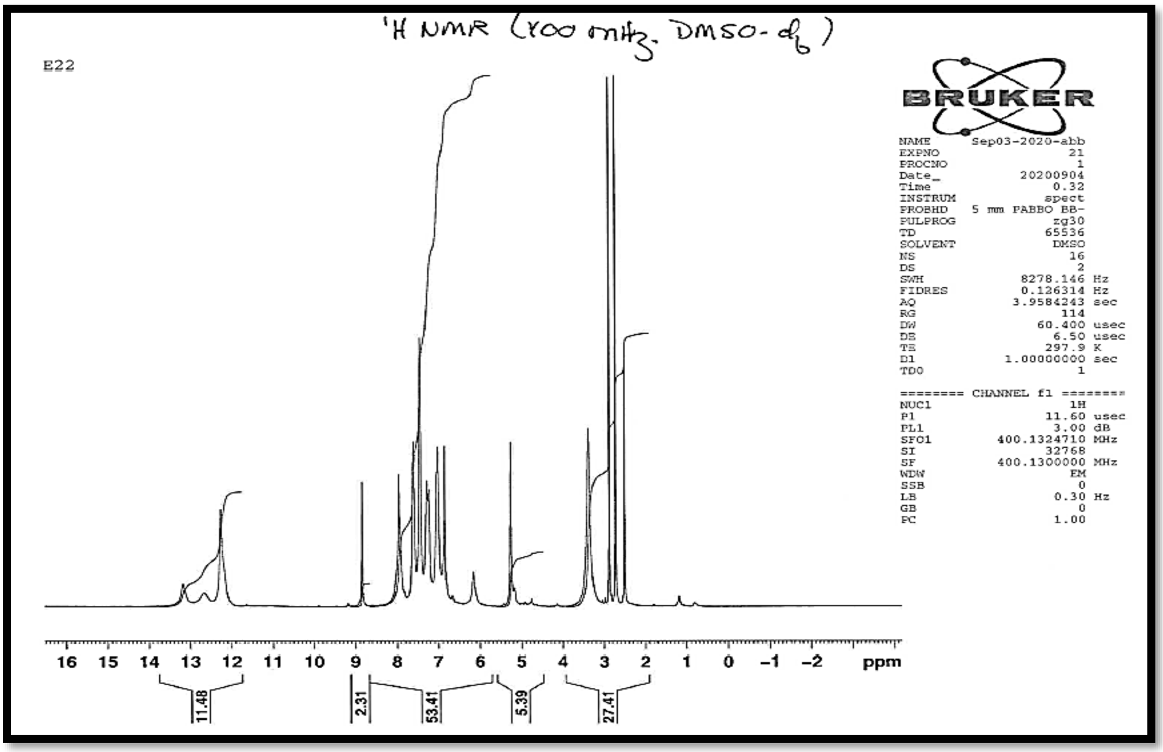

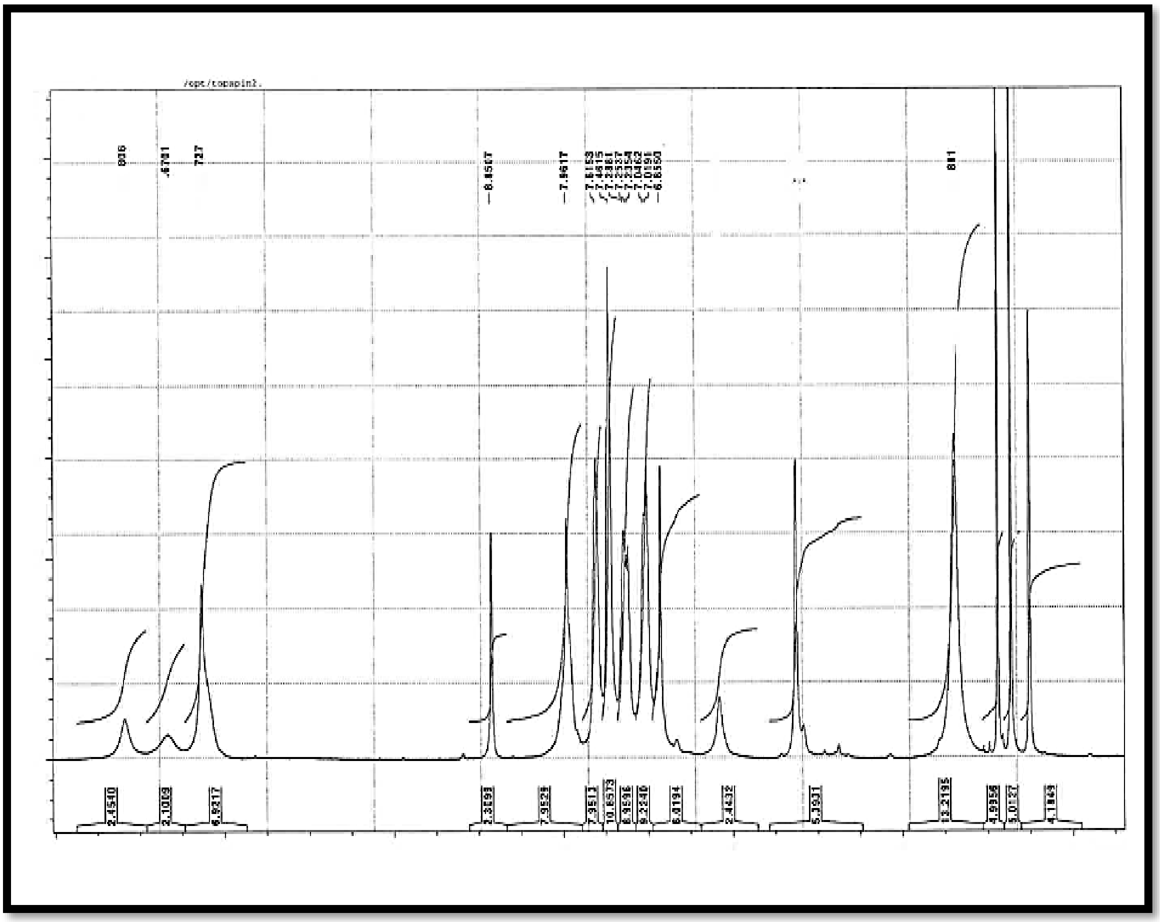

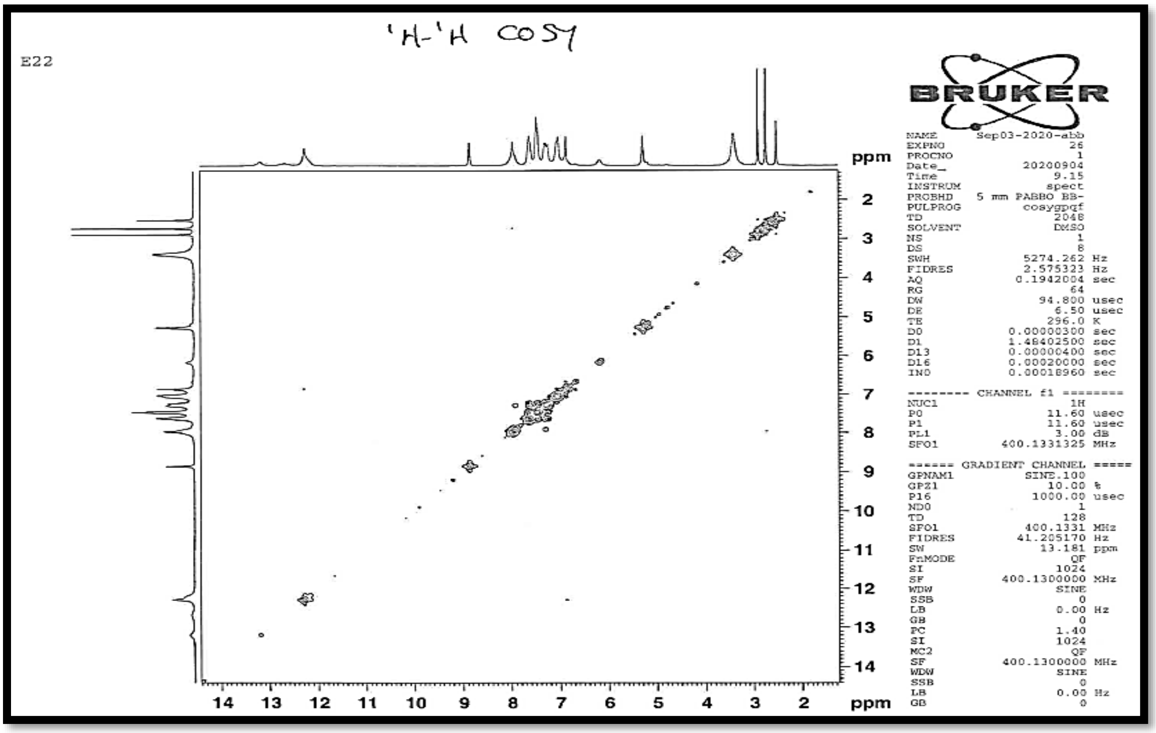

1H-1H COSY
E22
BRUKER
NAME Sep03-2020-abb
EXPNO 26
PROCNO 1
Date_ 20200904
Time 9.15
INSTRUM spect
PROBHD 5 mm PABBO BB-
PULPROG cosygpqf
TD 2048
SOLVENT DMSO
NS 1
DS 8
SWH 5274.262 Hz
FIDRES 2.575323 Hz
AQ 0.1942004 sec
RG 64
DW 94.800 usec
DE 6.50 usec
TE 296.0 K
D0 0.00000300 sec
D1 1.48402500 sec
D13 0.00000400 sec
D16 0.00020000 sec
IN0 0.00018960 sec
======== CHANNEL f1 ========
NUC1 1H
P0 11.60 usec
P1 11.60 usec
PL1 3.00 dB
SFO1 400.1331325 MHz
====== GRADIENT CHANNEL =====
GPNAM1 SINE.100
GPZ1 10.00 %
P16 1000.00 usec
ND0 1
TD 128
SFO1 400.1331 MHz
FIDRES 41.205170 Hz
SW 13.181 ppm
FnMODE QF
SI 1024
SF 400.1300000 MHz
WDW SINE
SSB 0
LB 0.00 Hz
GB 0
PC 1.40
SI 1024
MC2 QF
SF 400.1300000 MHz
WDW SINE
SSB 0
LB 0.00 Hz
GB 0
ppm

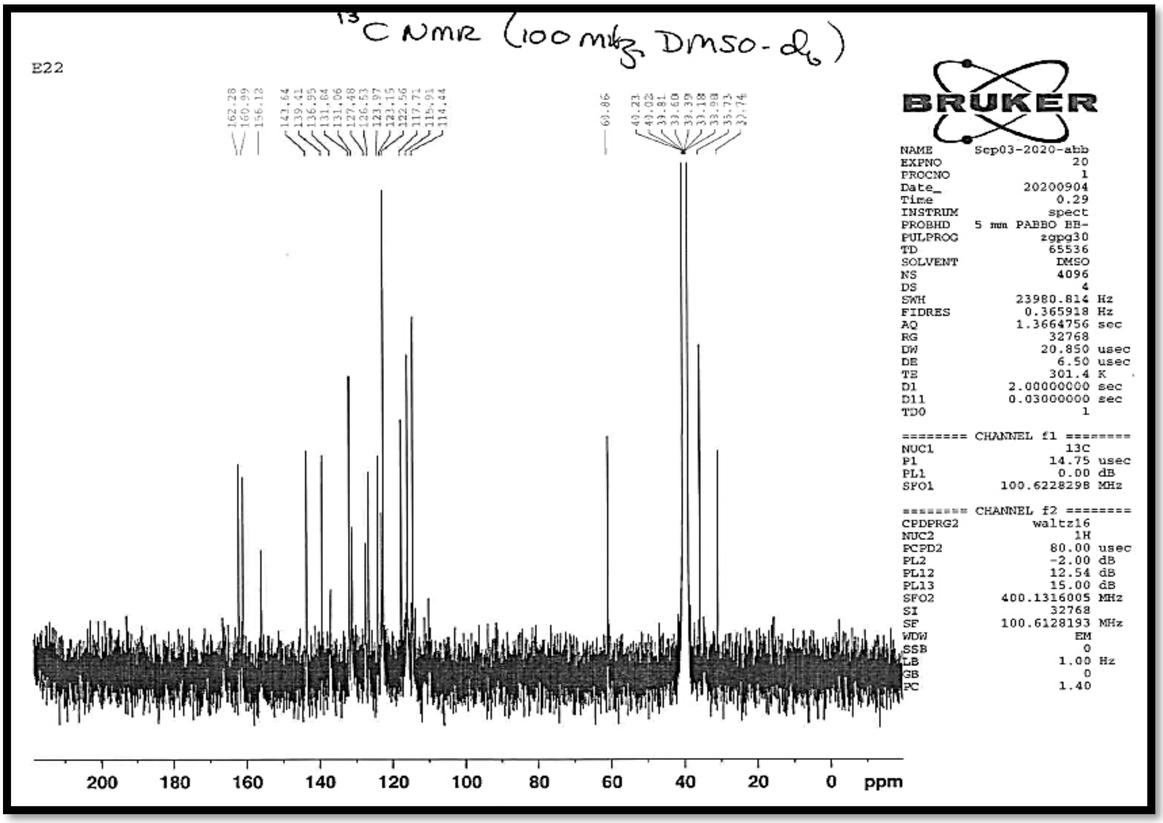
E22
13C NMR (100 MHz, DMSO-d6)
162.28 160.99 156.12
143.64 139.41 136.95 131.84 131.06 127.48 126.53 123.97 123.15 122.56 117.71 115.91 114.44
60.86
40.23 40.02 39.81 39.60 39.39 39.18 38.98 35.73 32.74
BRUKER
NAME Sep03-2020-abb
EXPNO 20
PROCNO 1
Date_ 20200904
Time 0.29
INSTRUM spect
PROBHD 5 mm PABBO BB-
PULPROG zgpg30
TD 65536
SOLVENT DMSO
NS 4096
DS 4
SWH 23980.814 Hz
FIDRES 0.365918 Hz
AQ 1.3664756 sec
RG 32768
DW 20.850 usec
DE 6.50 usec
TE 301.4 K
D1 2.00000000 sec
D11 0.03000000 sec
TD0 1
======== CHANNEL f1 ========
NUC1 13C
P1 14.75 usec
PL1 0.00 dB
SFO1 100.6228298 MHz
======== CHANNEL f2 ========
CPDPRG2 waltz16
NUC2 1H
PCPD2 80.00 usec
PL2 -2.00 dB
PL12 12.54 dB
PL13 15.00 dB
SFO2 400.1316005 MHz
SI 32768
SF 100.6128193 MHz
WDW EM
SSB 0
LB 1.00 Hz
GB 0
PC 1.40
200 180 160 140 120 100 80 60 40 20 0 ppm

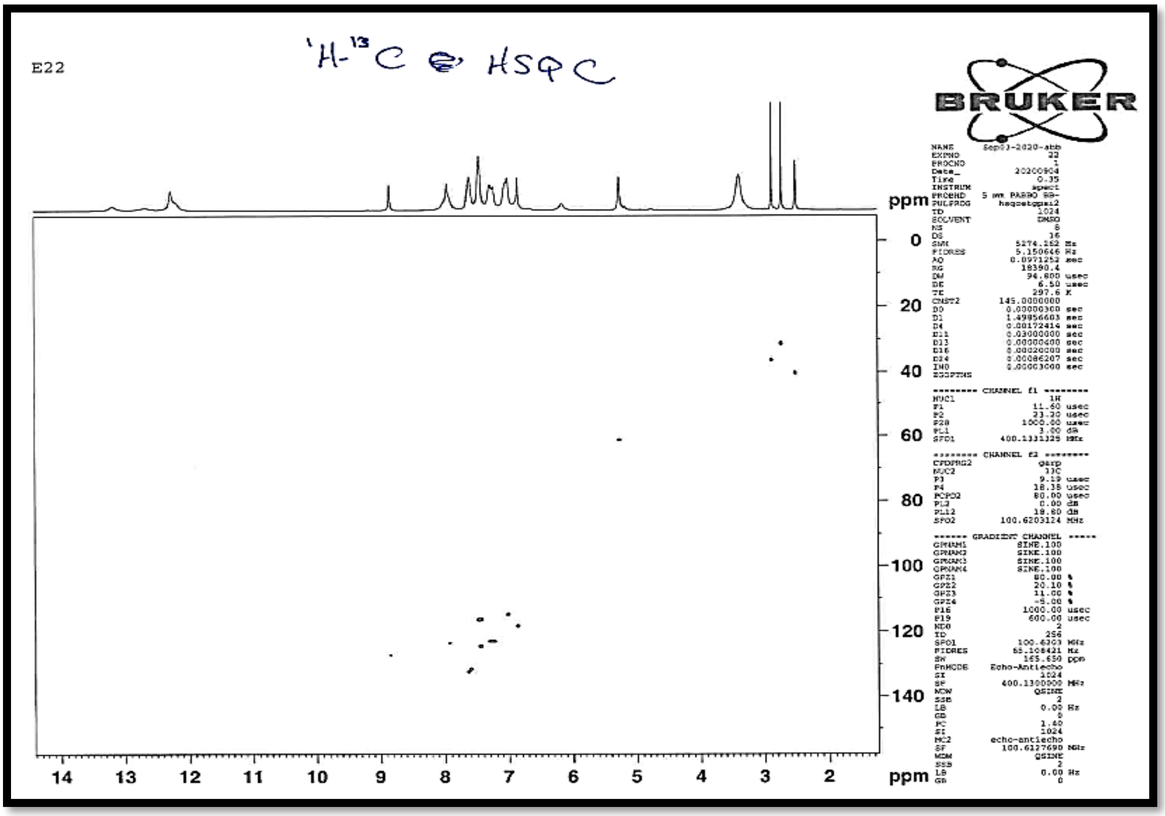
E22
1H-13C HSQC
BRUKER
ppm
0 20 40 60 80 100 120 140
14 13 12 11 10 9 8 7 6 5 4 3 2 ppm

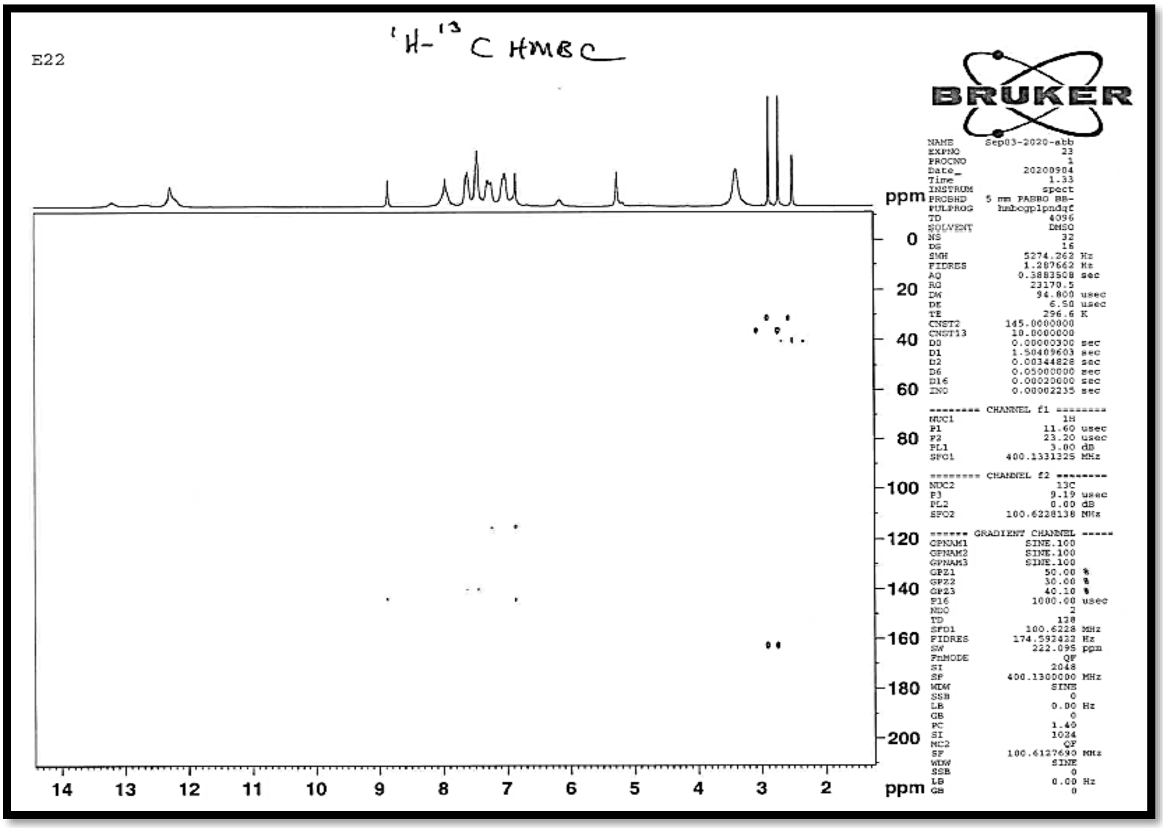
E22
¹H-¹³C HMBC
BRUKER
ppm

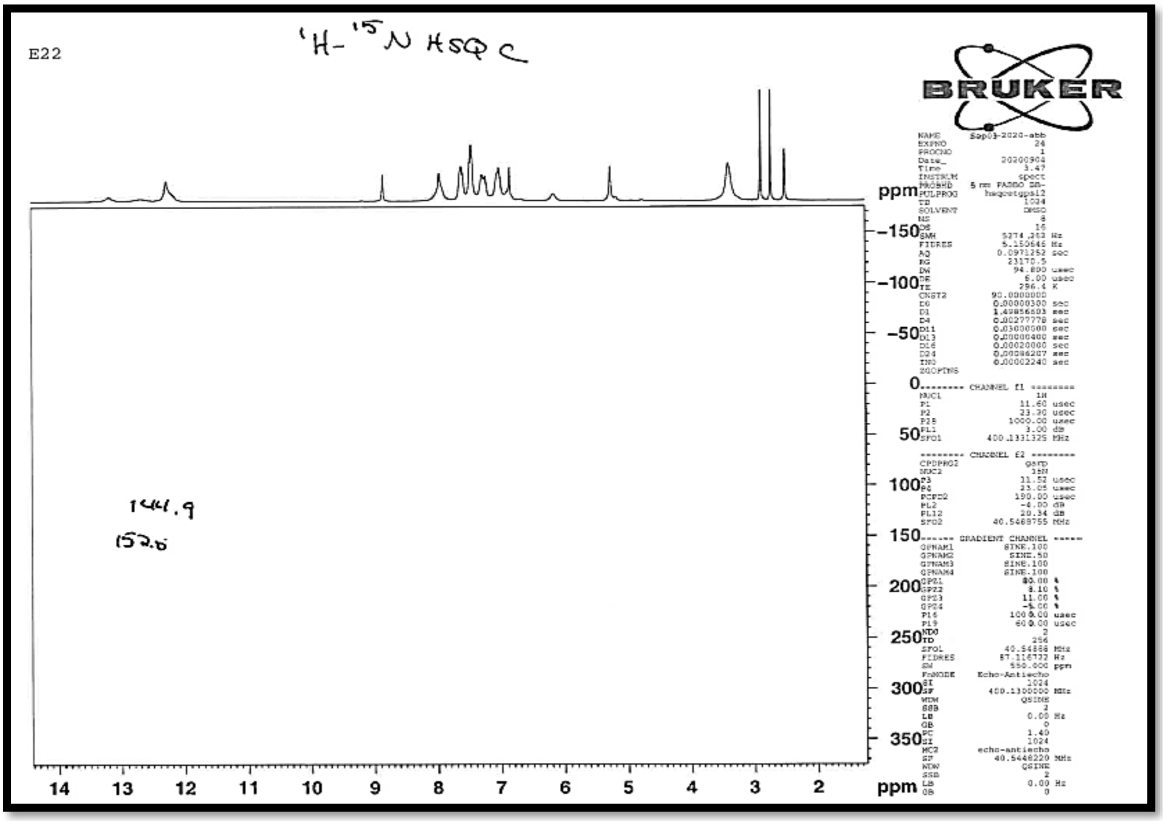
E22
¹H-¹⁵N HSQC
BRUKER
144.9
152.6
ppm

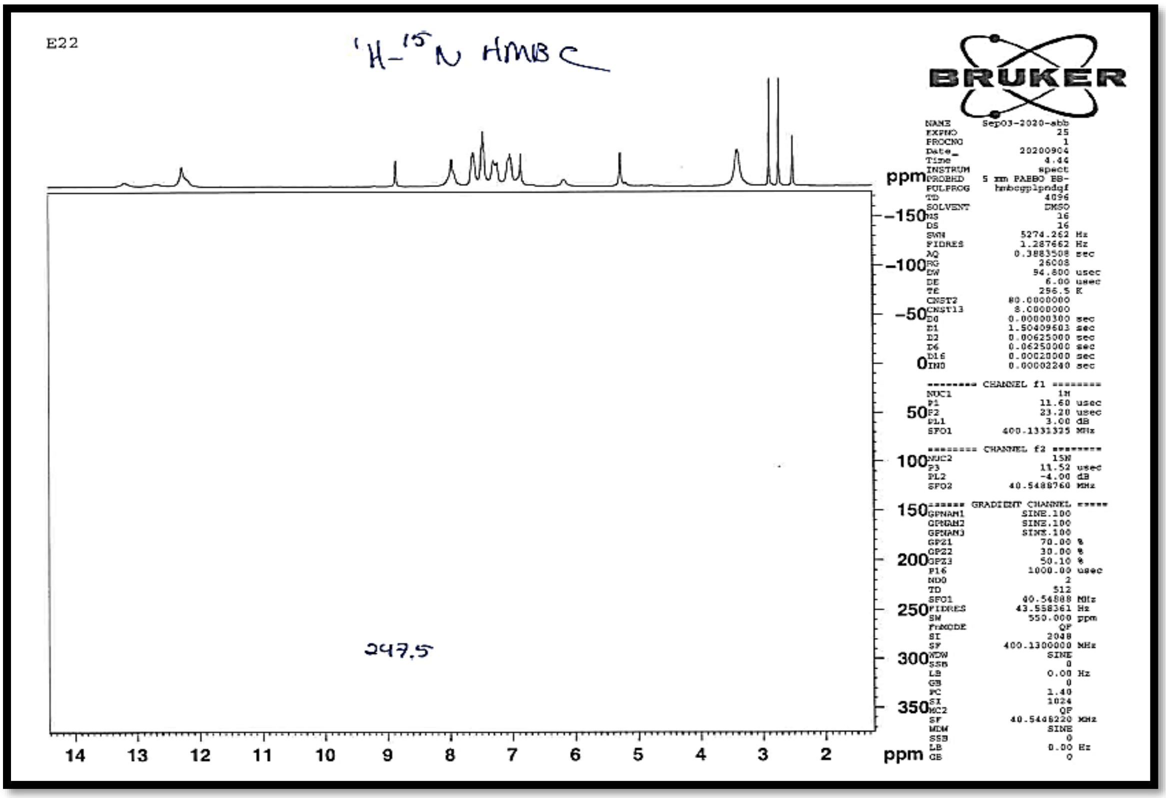
E22
1H-15N HMBC
247.5
NAME Sep03-2020-abb
EXPNO 25
PROCNO 1
Date_ 20200904
Time 4.44
INSTRUM spect
PROBHD 5 mm PABBO BB-
PULPROG hmbcgplpndqf
TD 4096
SOLVENT DMSO
NS 16
DS 16
SWH 5274.262 Hz
FIDRES 1.287662 Hz
AQ 0.3883508 sec
RG 26008
DW 94.800 usec
DE 6.00 usec
TE 296.5 K
CNST2 80.0000000
CNST13 8.0000000
D0 0.00000300 sec
D1 1.50409603 sec
D2 0.00625000 sec
D6 0.06250000 sec
D16 0.00020000 sec
IN0 0.00002240 sec
======== CHANNEL f1 ========
NUC1 1H
P1 11.60 usec
P2 23.20 usec
PL1 3.00 dB
SFO1 400.1331325 MHz
======== CHANNEL f2 ========
NUC2 15N
P3 11.52 usec
PL2 -4.00 dB
SFO2 40.5488760 MHz
====== GRADIENT CHANNEL =====
GPNAM1 SINE.100
GPNAM2 SINE.100
GPNAM3 SINE.100
GPZ1 70.00 %
GPZ2 30.00 %
GPZ3 50.10 %
P16 1000.00 usec
ND0 2
TD 512
SFO1 40.54888 MHz
FIDRES 43.558361 Hz
SW 550.000 ppm
FnMODE QF
SI 2048
SF 400.1300000 MHz
WDW SINE
SSB 0
LB 0.00 Hz
GB 0
PC 1.40
SI 1024
MC2 QF
SF 40.5448220 MHz
WDW SINE
SSB 0
LB 0.00 Hz
GB 0
ppm
-150
-100
-50
0
50
100
150
200
250
300
350
14 13 12 11 10 9 8 7 6 5 4 3 2 ppm

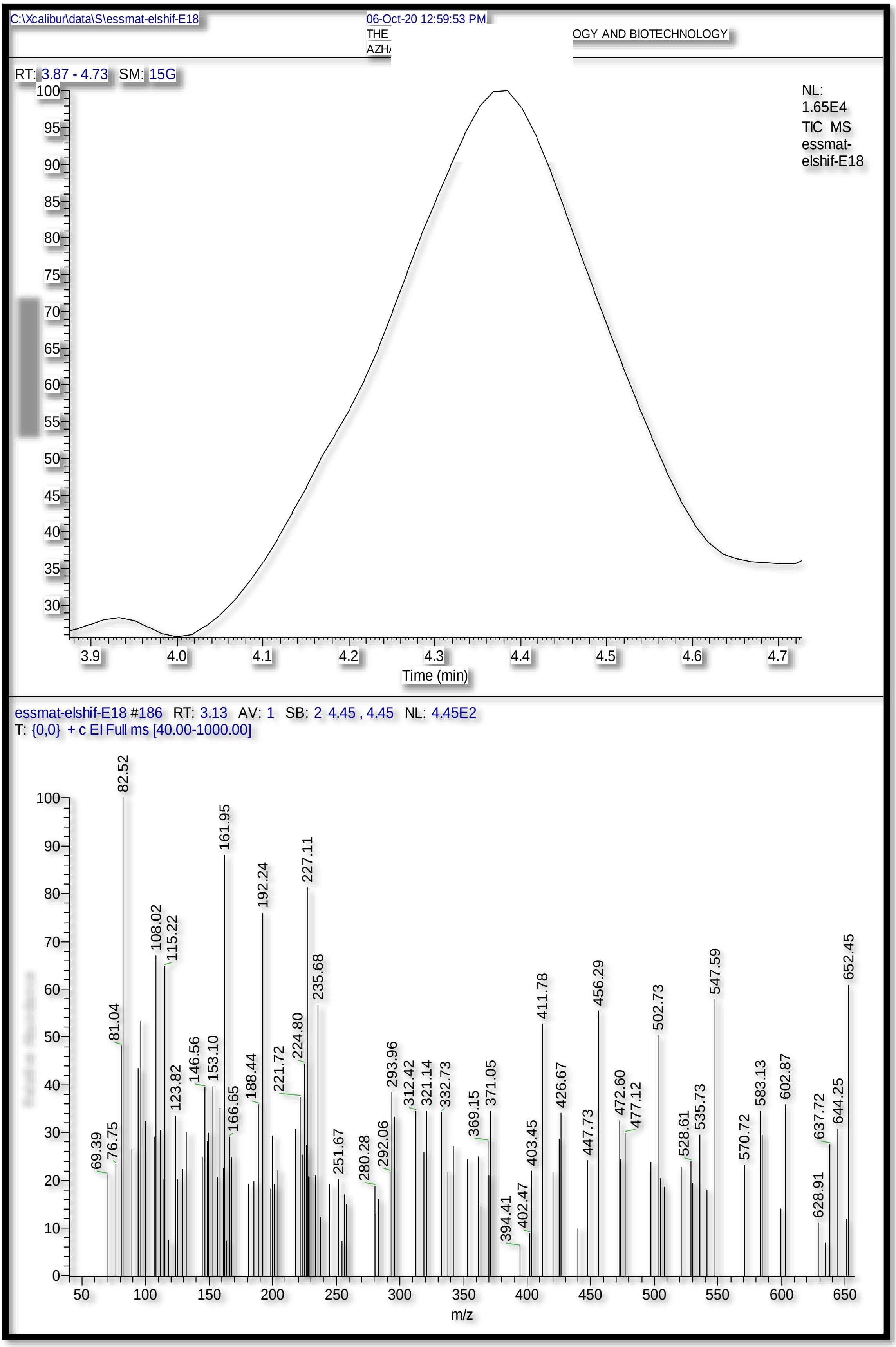

Spectrum data for Compound 8b

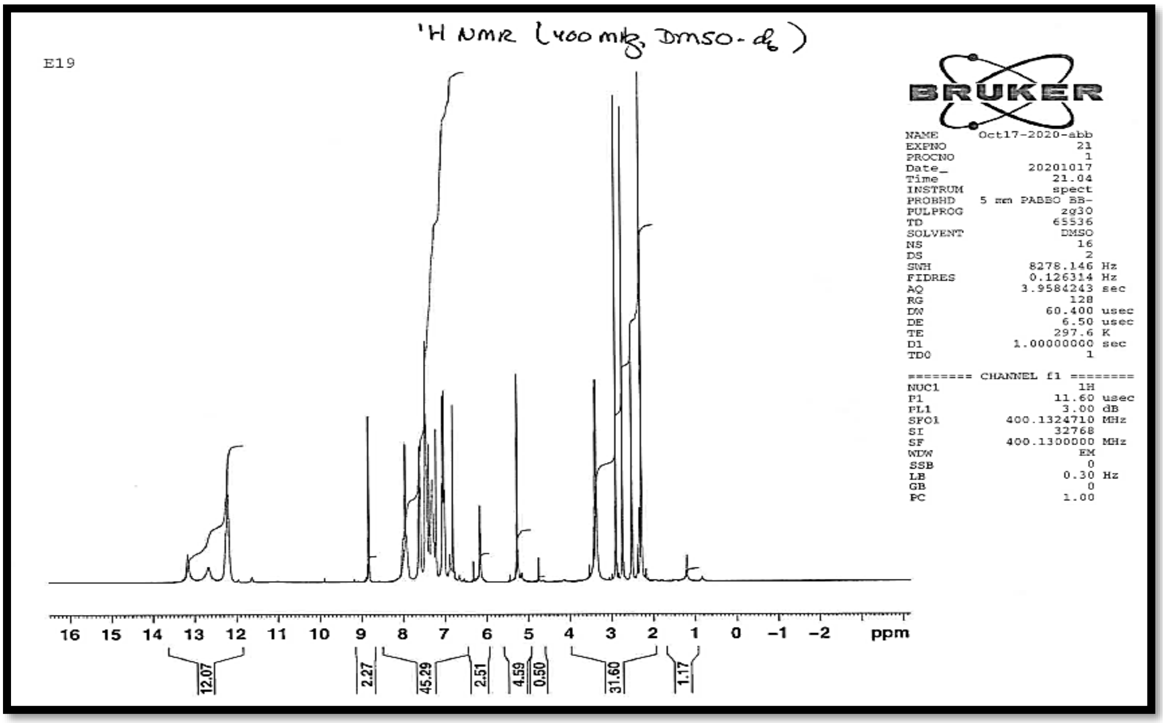

'H NMR (400 mHz, DMSO-d6)
E19
BRUKER
NAME Oct17-2020-abb
EXPNO 21
PROCNO 1
Date_ 20201017
Time 21.04
INSTRUM spect
PROBHD 5 mm PABBO BB-
PULPROG zg30
TD 65536
SOLVENT DMSO
NS 16
DS 2
SWH 8278.146 Hz
FIDRES 0.126314 Hz
AQ 3.9584243 sec
RG 128
DW 60.400 usec
DE 6.50 usec
TE 297.6 K
D1 1.00000000 sec
TD0 1
======== CHANNEL f1 ========
NUC1 1H
P1 11.60 usec
PL1 3.00 dB
SFO1 400.1324710 MHz
SI 32768
SF 400.1300000 MHz
WDW EM
SSB 0
LB 0.30 Hz
GB 0
PC 1.00
ppm
12.07
2.27
45.29
2.51
4.59
0.50
31.60
1.17

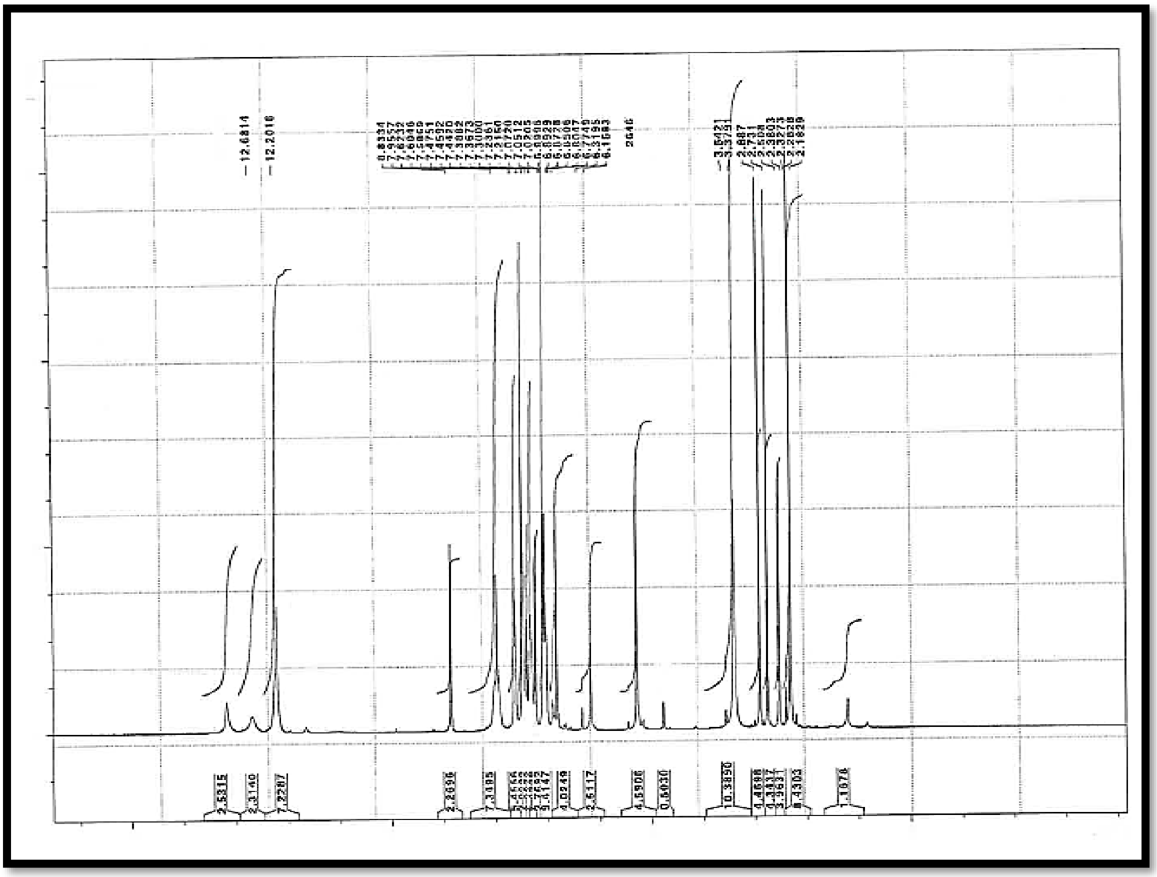

12.6814
12.2016

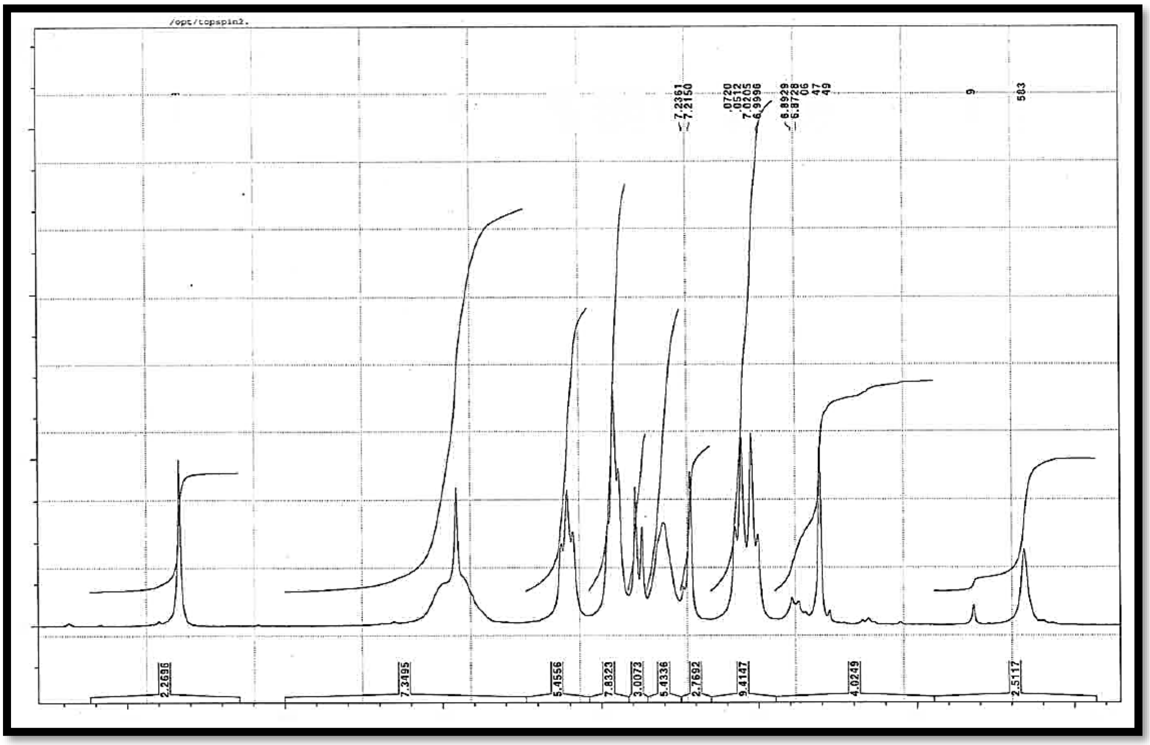

/opt/topspin2.
7.2361
7.2150
.0720
.0512
7.0205
6.9998
6.8929
6.8728
2.2696
7.3495
5.4556
7.8323
3.0073
5.4336
2.7692
9.4147
4.0249
2.5117

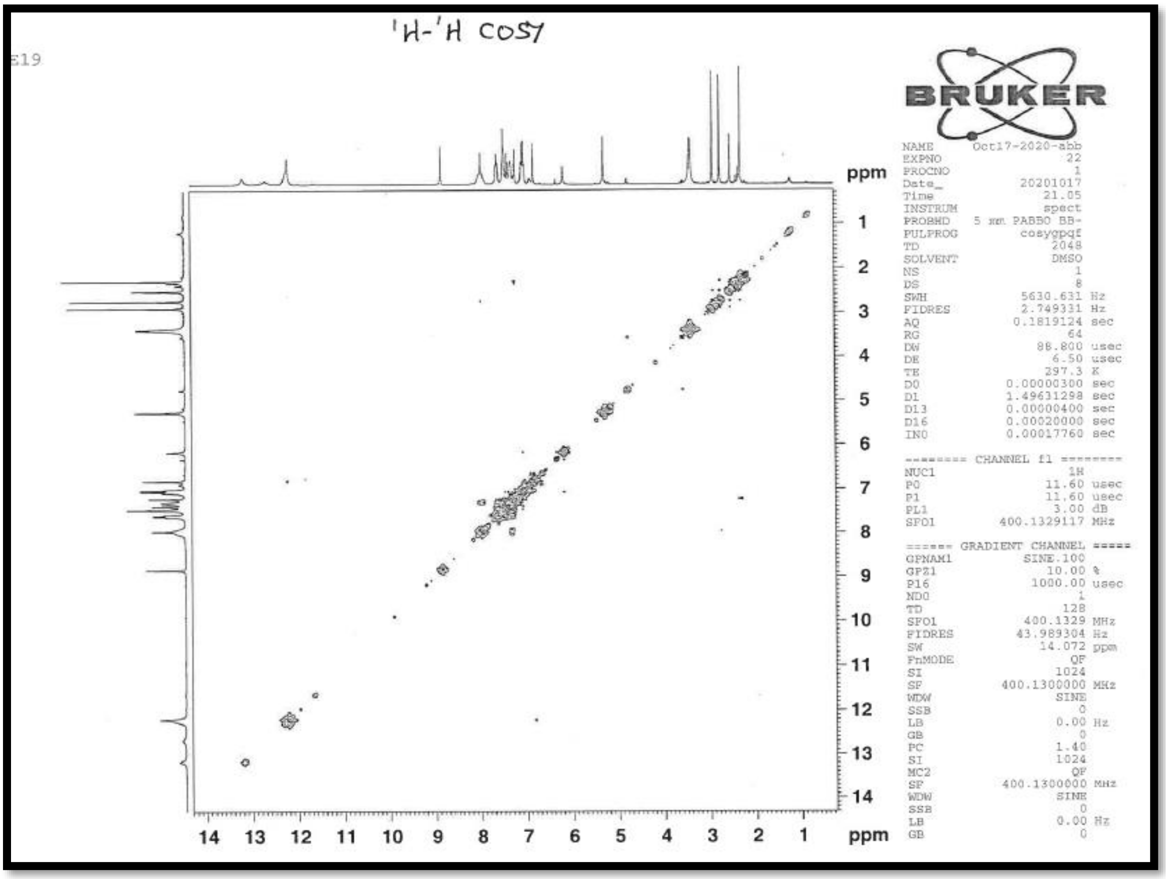

'H-'H COSY
E19
BRUKER
ppm
NAME Oct17-2020-abb
EXPNO 22
PROCNO 1
Date_ 20201017
Time 21.05
INSTRUM spect
PROBHD 5 mm PABBO BB-
PULPROG cosygpqf
TD 2048
SOLVENT DMSO
NS 1
DS 8
SWH 5630.631 Hz
FIDRES 2.749331 Hz
AQ 0.1819124 sec
RG 64
DW 88.800 usec
DE 6.50 usec
TE 297.3 K
D0 0.00000300 sec
D1 1.49631298 sec
D13 0.00000400 sec
D16 0.00020000 sec
IN0 0.00017760 sec
======== CHANNEL f1 ========
NUC1 1H
P0 11.60 usec
P1 11.60 usec
PL1 3.00 dB
SFO1 400.1329117 MHz
====== GRADIENT CHANNEL =====
GPNAM1 SINE.100
GPZ1 10.00 %
P16 1000.00 usec
ND0 1
TD 128
SFO1 400.1329 MHz
FIDRES 43.989304 Hz
SW 14.072 ppm
FnMODE QF
SI 1024
SF 400.1300000 MHz
WDW SINE
SSB 0
LB 0.00 Hz
GB 0
PC 1.40
SI 1024
MC2 QF
SF 400.1300000 MHz
WDW SINE
SSB 0
LB 0.00 Hz
GB 0

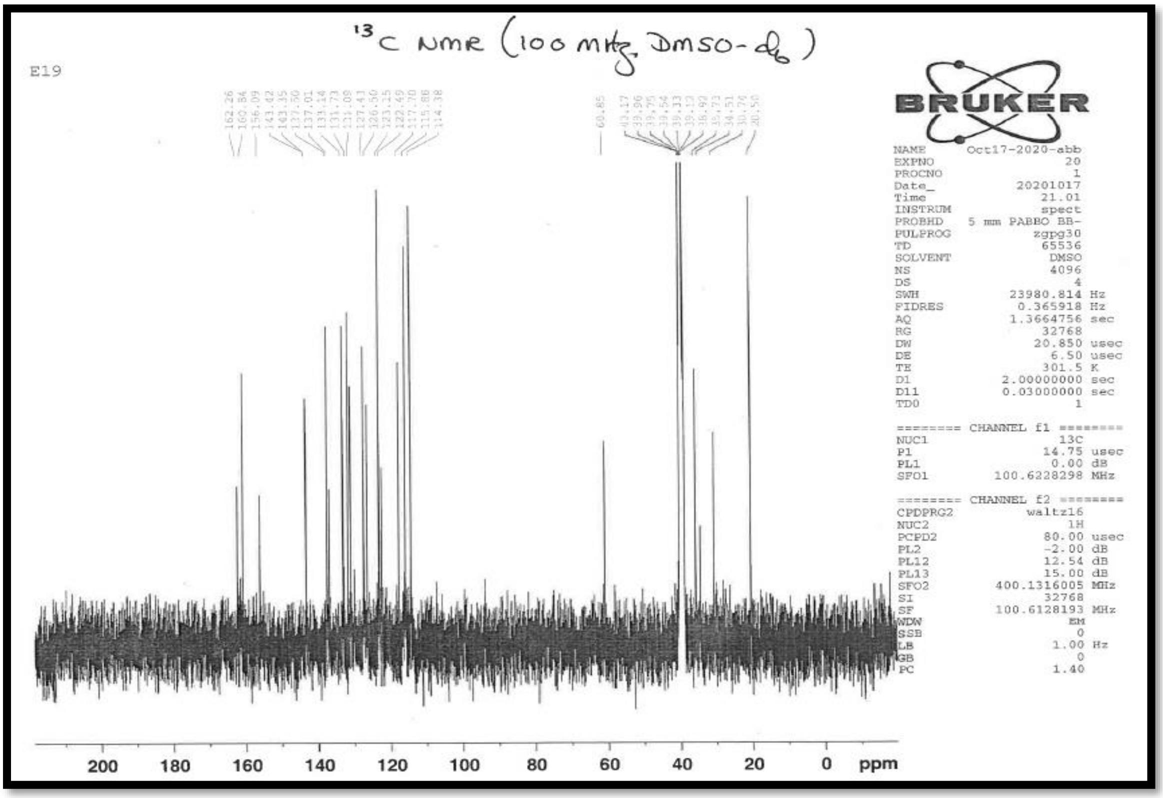
13C NMR (100 MHz, DMSO-d6)
E19
ppm

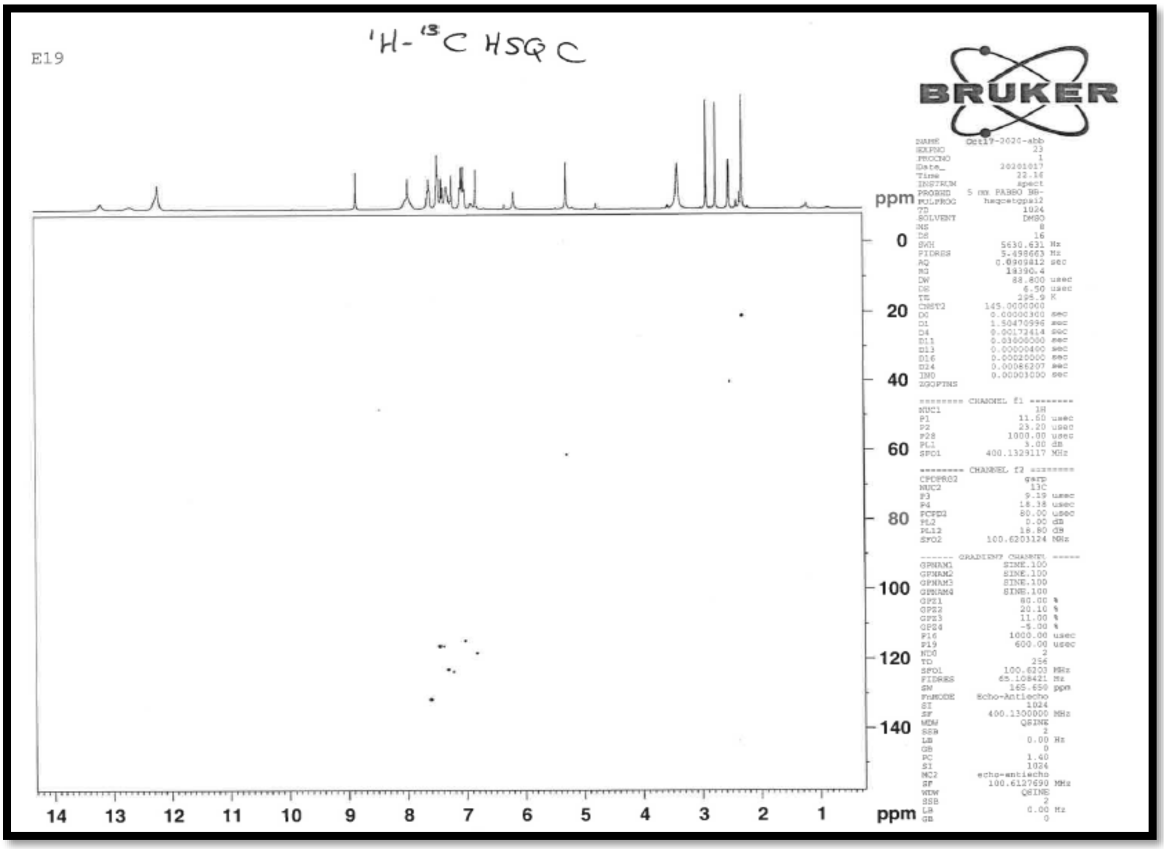
1H-13C HSQC
E19
ppm

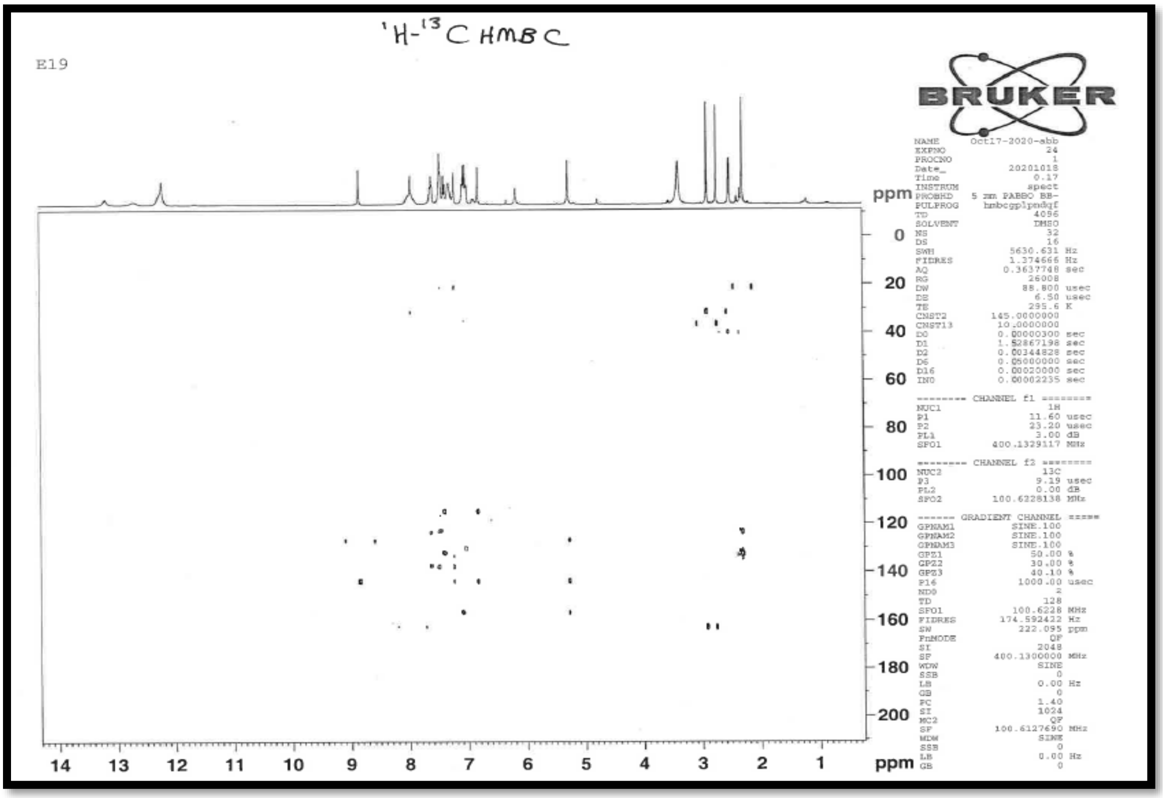

¹H-¹³C HMBC
E19
BRUKER
NAME Oct17-2020-abb
ppm
0
20
40
60
80
100
120
140
160
180
200
14 13 12 11 10 9 8 7 6 5 4 3 2 1 ppm

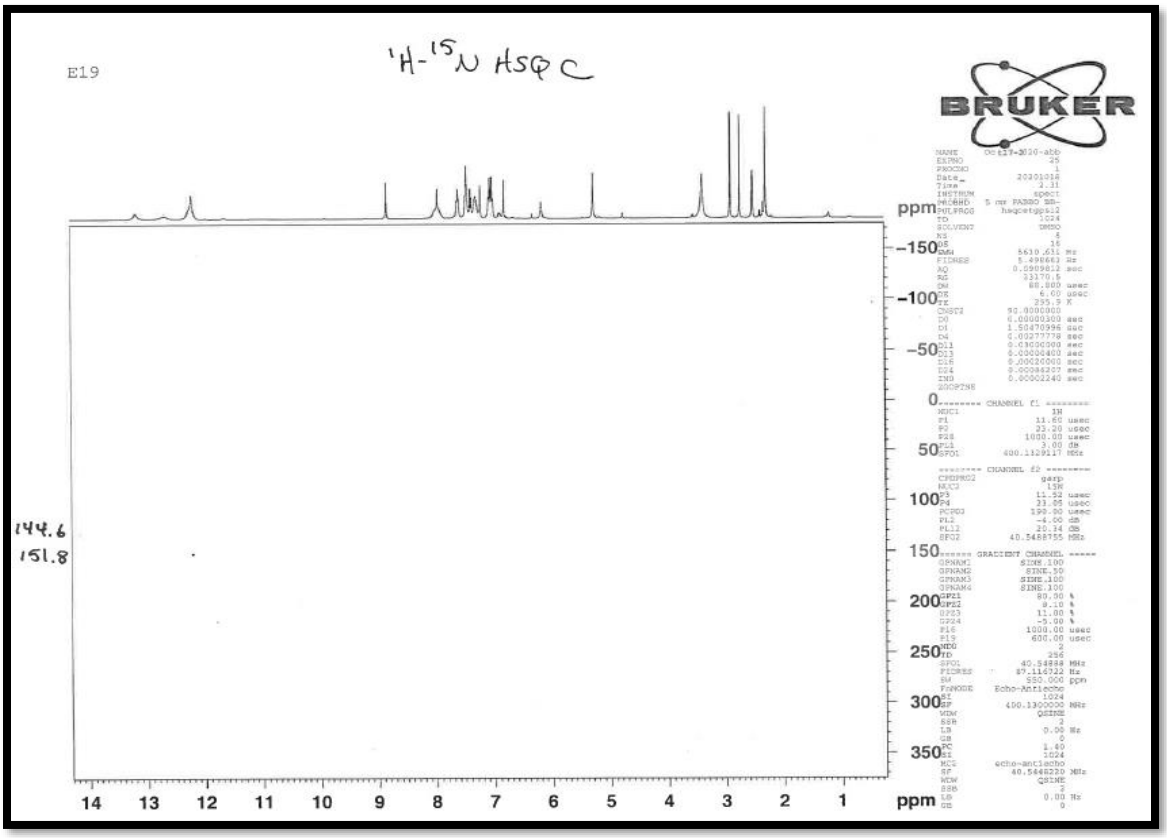

¹H-¹⁵N HSQC
E19
BRUKER
144.6
151.8
ppm
-150
-100
-50
0
50
100
150
200
250
300
350
14 13 12 11 10 9 8 7 6 5 4 3 2 1 ppm

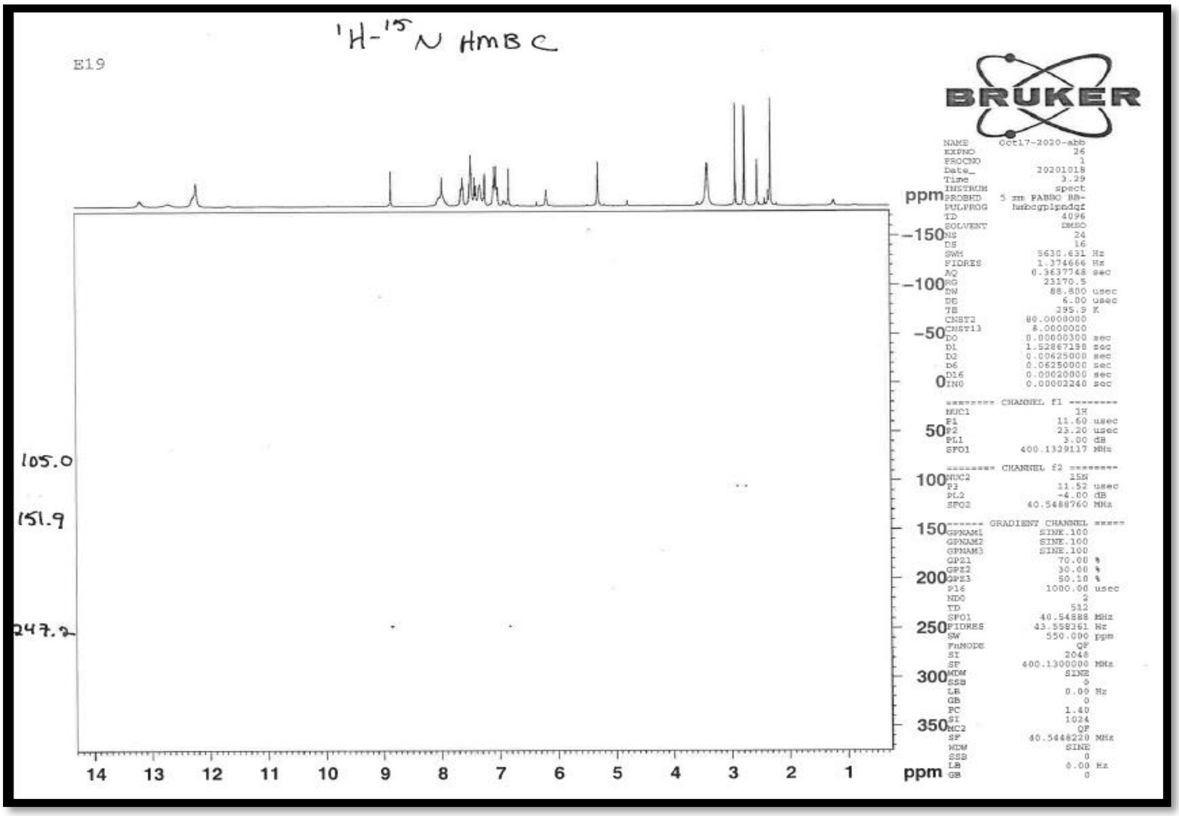
1H-15N HMBC
E19
105.0
151.9
247.2
NAME Oct17-2020-abb
EXPNO 26
PROCNO 1
Date_ 20201018
Time 3.29
INSTRUM spect
PROBHD 5 mm PABBO BB-
PULPROG hmbcgplpndqf
TD 4096
SOLVENT DMSO
NS 24
DS 16
SWH 5630.631 Hz
FIDRES 1.374666 Hz
AQ 0.3637748 sec
RG 23170.5
DW 88.800 usec
DE 6.00 usec
TE 295.9 K
CNST2 80.0000000
CNST13 8.0000000
D0 0.00000300 sec
D1 1.52867198 sec
D2 0.00625000 sec
D6 0.06250000 sec
D16 0.00020000 sec
IN0 0.00002240 sec
======== CHANNEL f1 ========
NUC1 1H
P1 11.60 usec
P2 23.20 usec
PL1 3.00 dB
SFO1 400.1329117 MHz
======== CHANNEL f2 ========
NUC2 15N
P3 11.52 usec
PL2 -4.00 dB
SFO2 40.5488760 MHz
====== GRADIENT CHANNEL =====
GPNAM1 SINE.100
GPNAM2 SINE.100
GPNAM3 SINE.100
GPZ1 70.00 %
GPZ2 30.00 %
GPZ3 50.10 %
P16 1000.00 usec
ND0 2
TD 512
SFO1 40.54888 MHz
FIDRES 43.558361 Hz
SW 550.000 ppm
FnMODE QF
SI 2048
SF 400.1300000 MHz
WDW SINE
SSB 0
LB 0.00 Hz
GB 0
PC 1.40
SI 1024
MC2 QF
SF 40.5448220 MHz
WDW SINE
SSB 0
LB 0.00 Hz
GB 0
ppm
14 13 12 11 10 9 8 7 6 5 4 3 2 1 ppm
-150 -100 -50 0 50 100 150 200 250 300 350 ppm

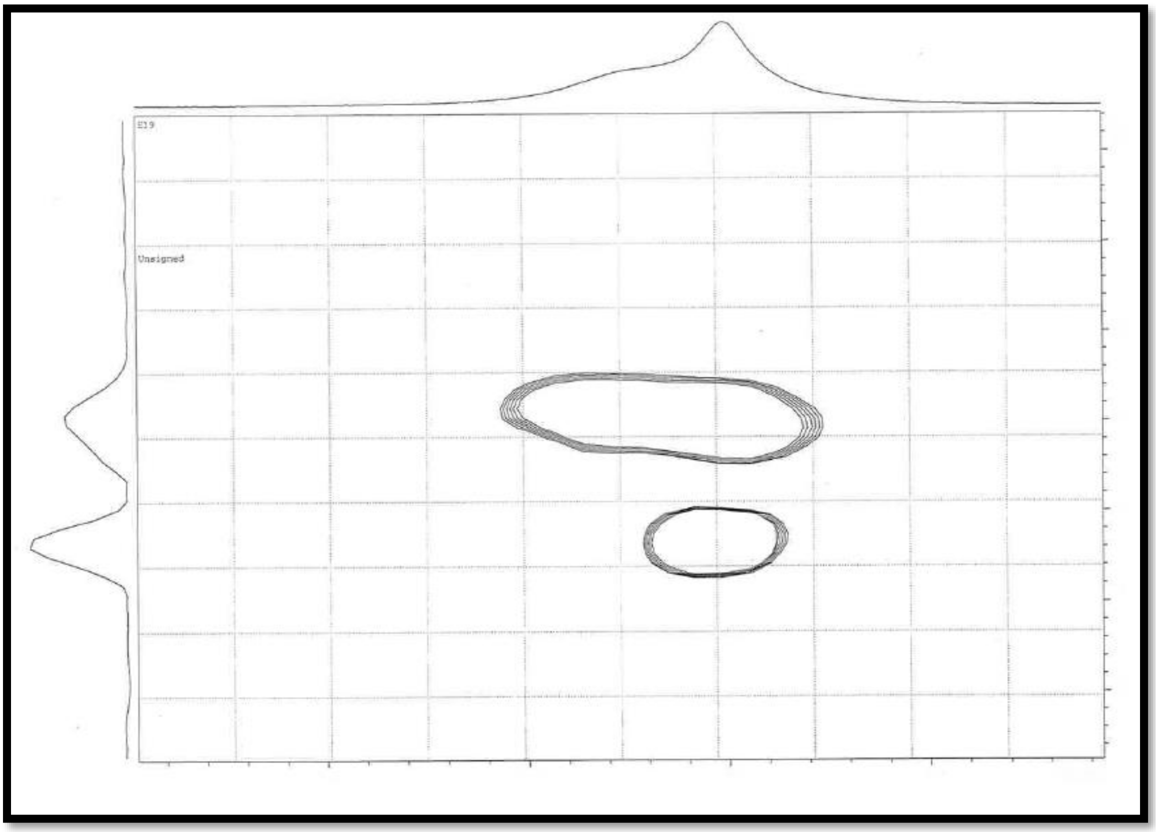
E19
Unsigned

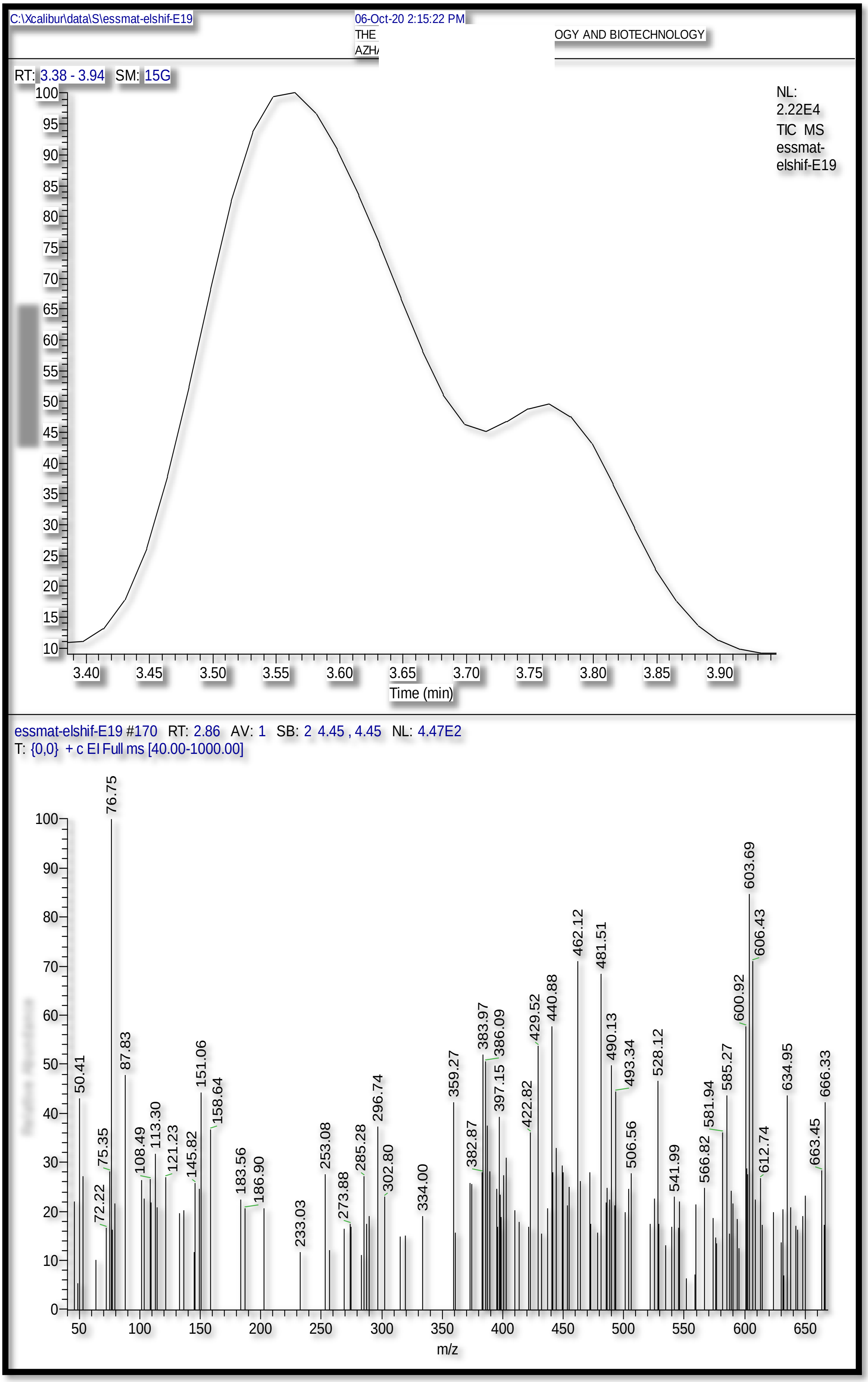

Spectrum data for Compound 8c

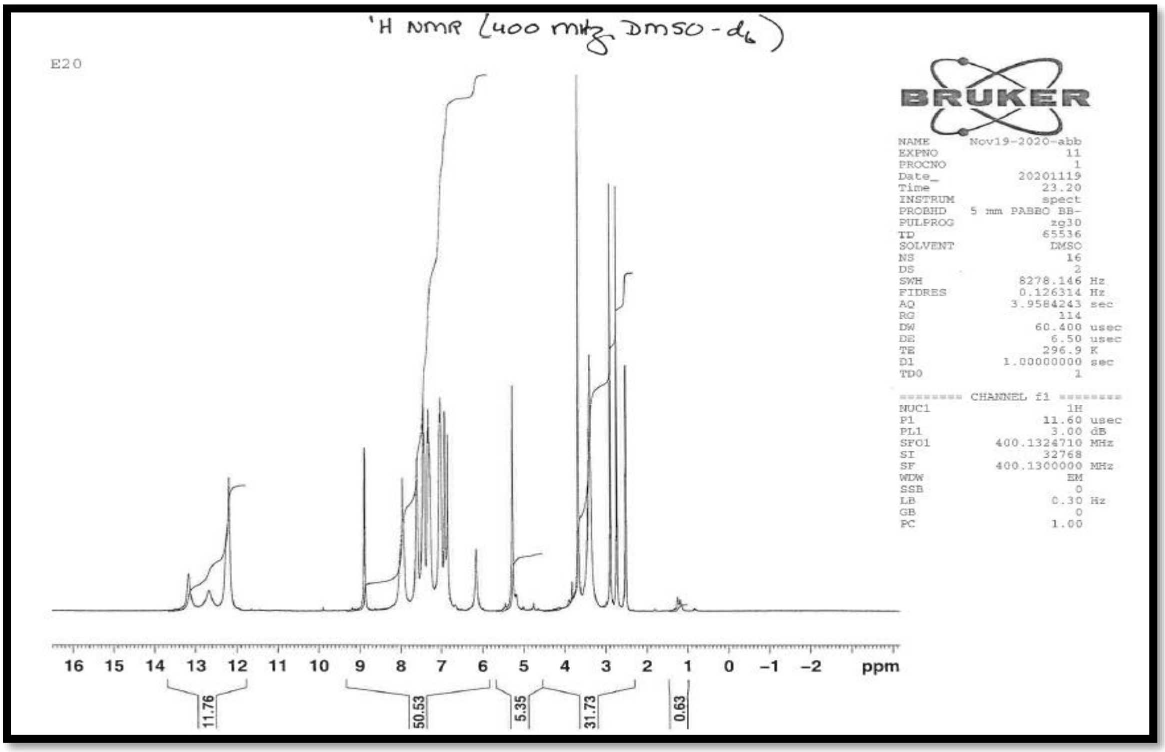

'H NMR (400 MHz, DMSO-d6)
E20
BRUKER
NAME Nov19-2020-abb
EXPNO 11
PROCNO 1
Date_ 20201119
Time 23.20
INSTRUM spect
PROBHD 5 mm PABBO BB-
PULPROG zg30
TD 65536
SOLVENT DMSO
NS 16
DS 2
SWH 8278.146 Hz
FIDRES 0.126314 Hz
AQ 3.9584243 sec
RG 114
DW 60.400 usec
DE 6.50 usec
TE 296.9 K
D1 1.00000000 sec
TD0 1
======== CHANNEL f1 ========
NUC1 1H
P1 11.60 usec
PL1 3.00 dB
SFO1 400.1324710 MHz
SI 32768
SF 400.1300000 MHz
WDW EM
SSB 0
LB 0.30 Hz
GB 0
PC 1.00
16 15 14 13 12 11 10 9 8 7 6 5 4 3 2 1 0 −1 −2 ppm
11.76
50.53
5.35
31.73
0.63

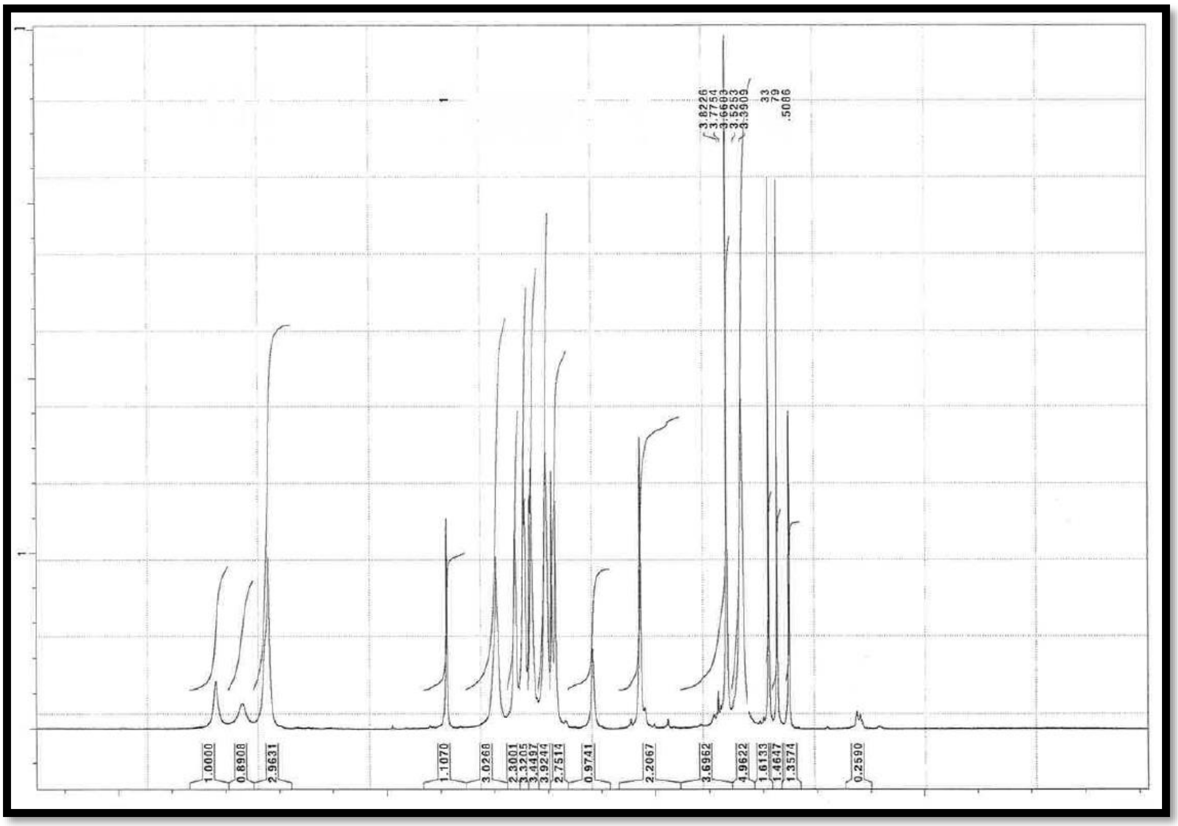

3.8226
3.7754
3.6603
3.5523
3.3903
1.0000
0.8908
2.9631
1.1070
3.0268
2.3001
3.3205
3.4497
3.9244
2.7514
0.9741
2.2067
3.6962
4.9622
1.6133
1.4647
1.3574
0.2590

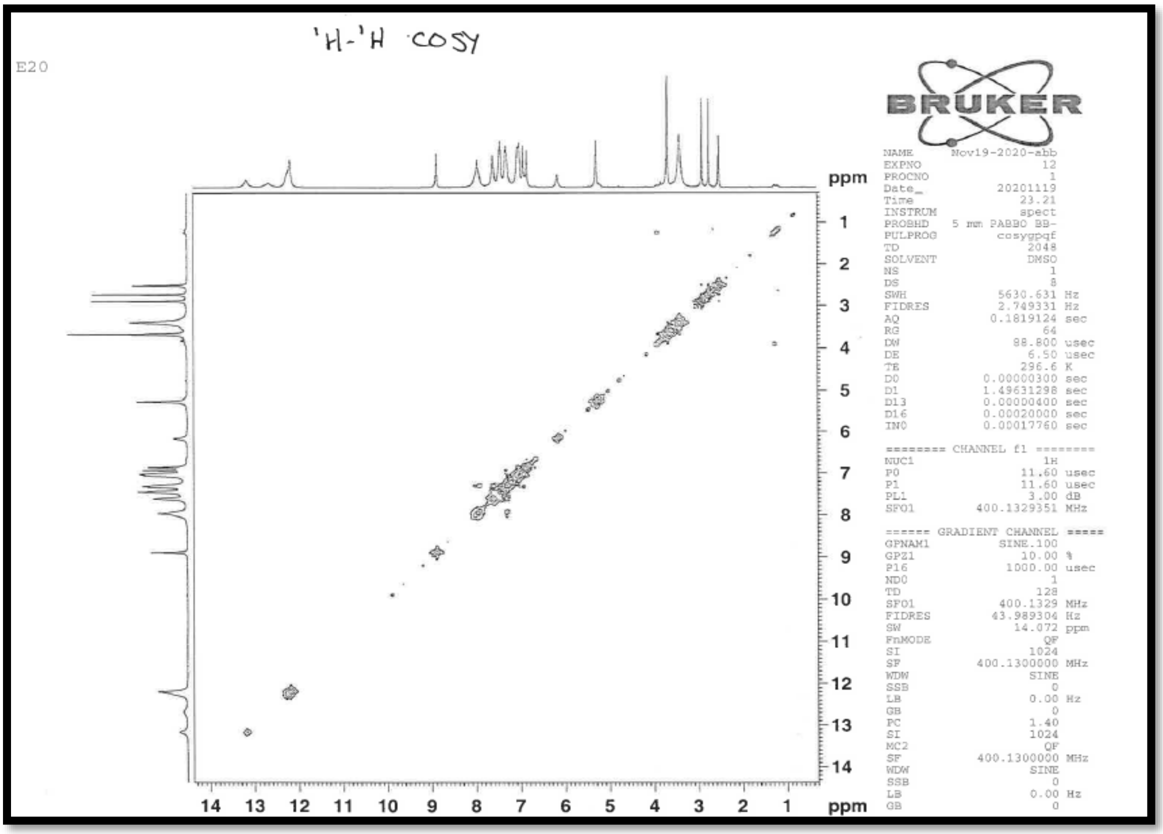

'H-'H COSY
E20
BRUKER
ppm

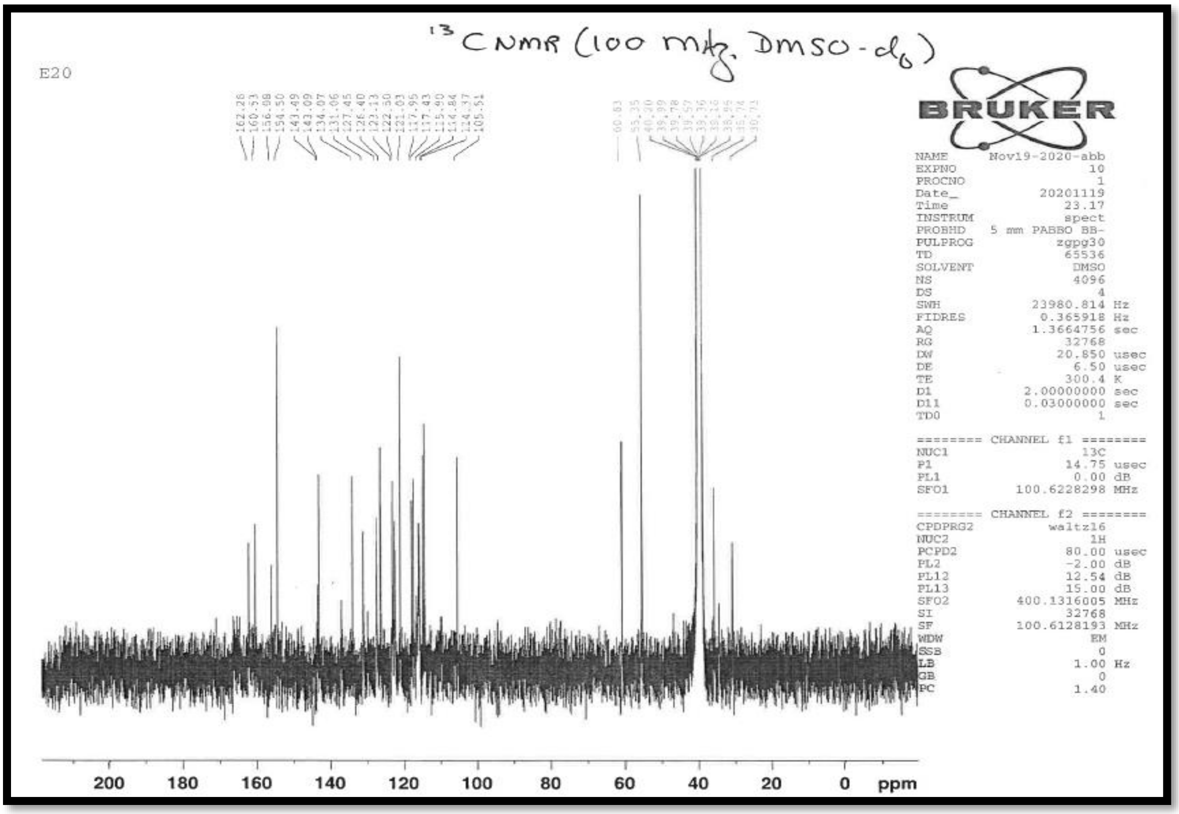

13CNMR (100 MHz, DMSO-d6)
E20
BRUKER
ppm

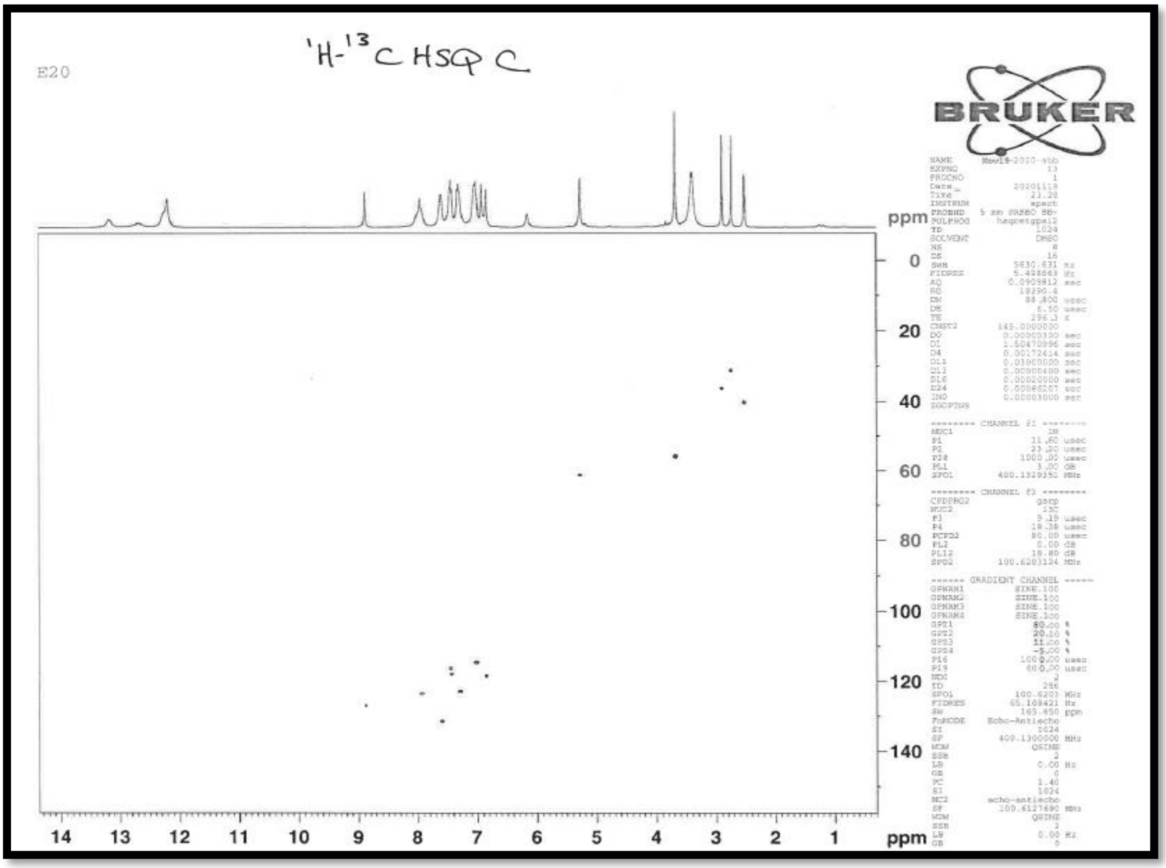
E20
'H-13C HSQC
BRUKER
ppm
0
20
40
60
80
100
120
140
14 13 12 11 10 9 8 7 6 5 4 3 2 1 ppm

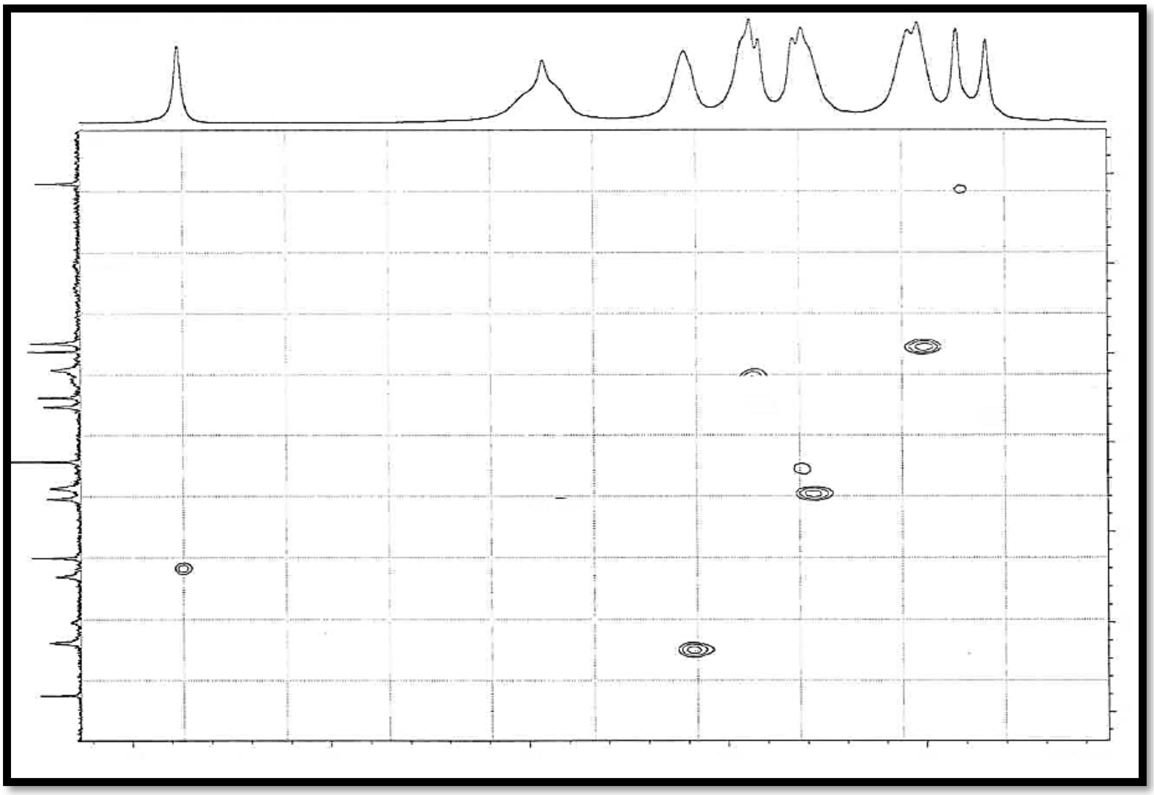

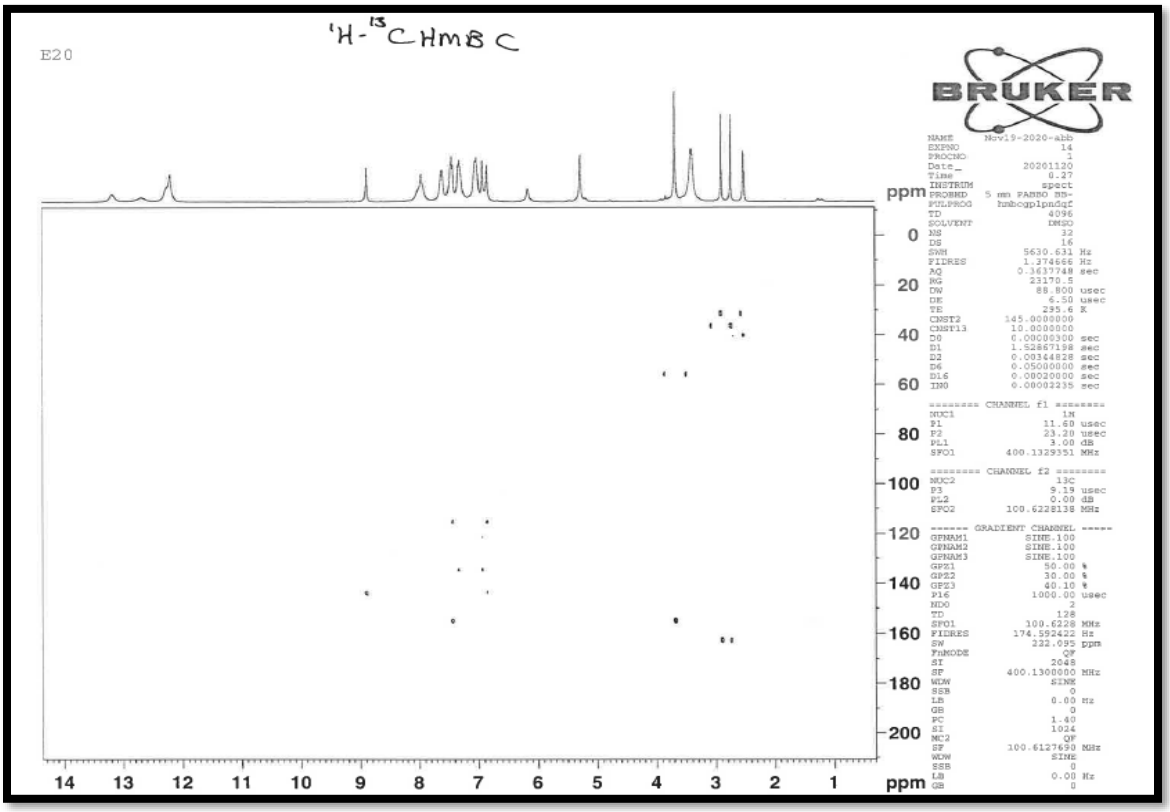
E20
1H-13C HMBC
ppm
NAME Nov19-2020-abb
EXPNO 14

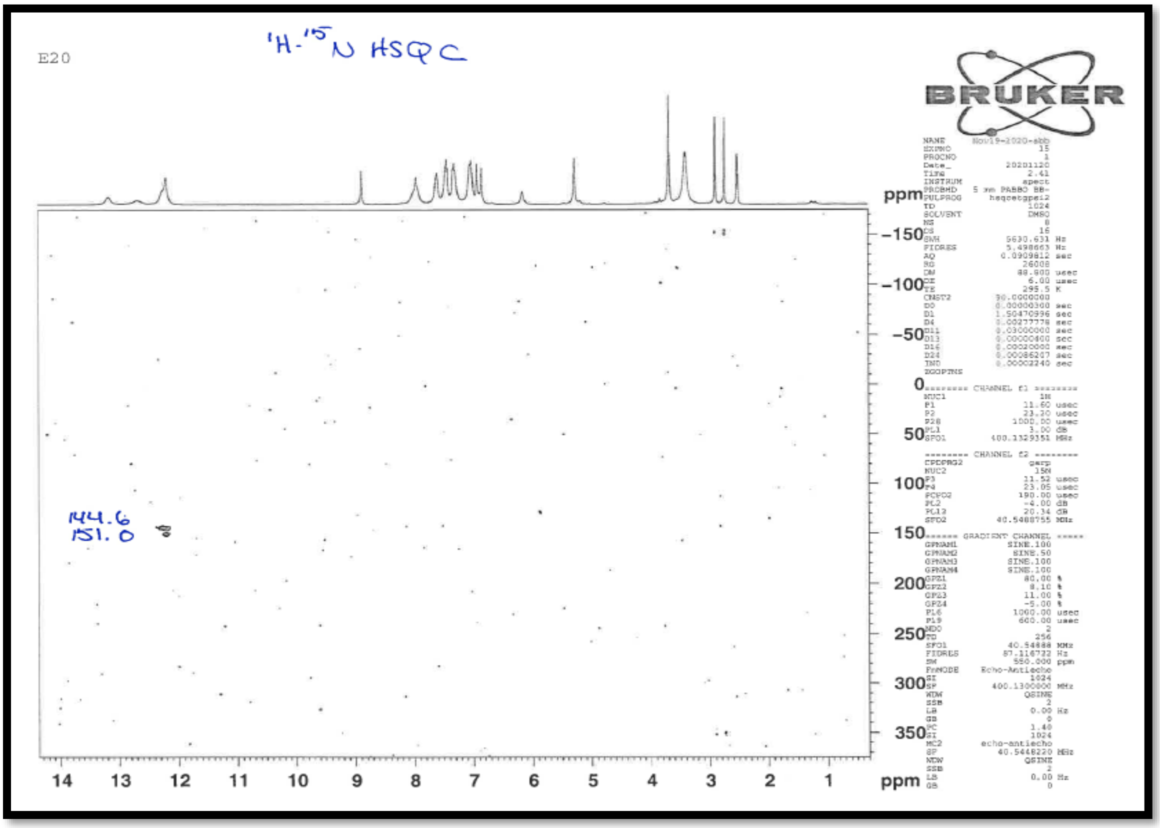
E20
1H-15N HSQC
144.6
151.0
ppm
NAME Nov19-2020-abb
EXPNO 15

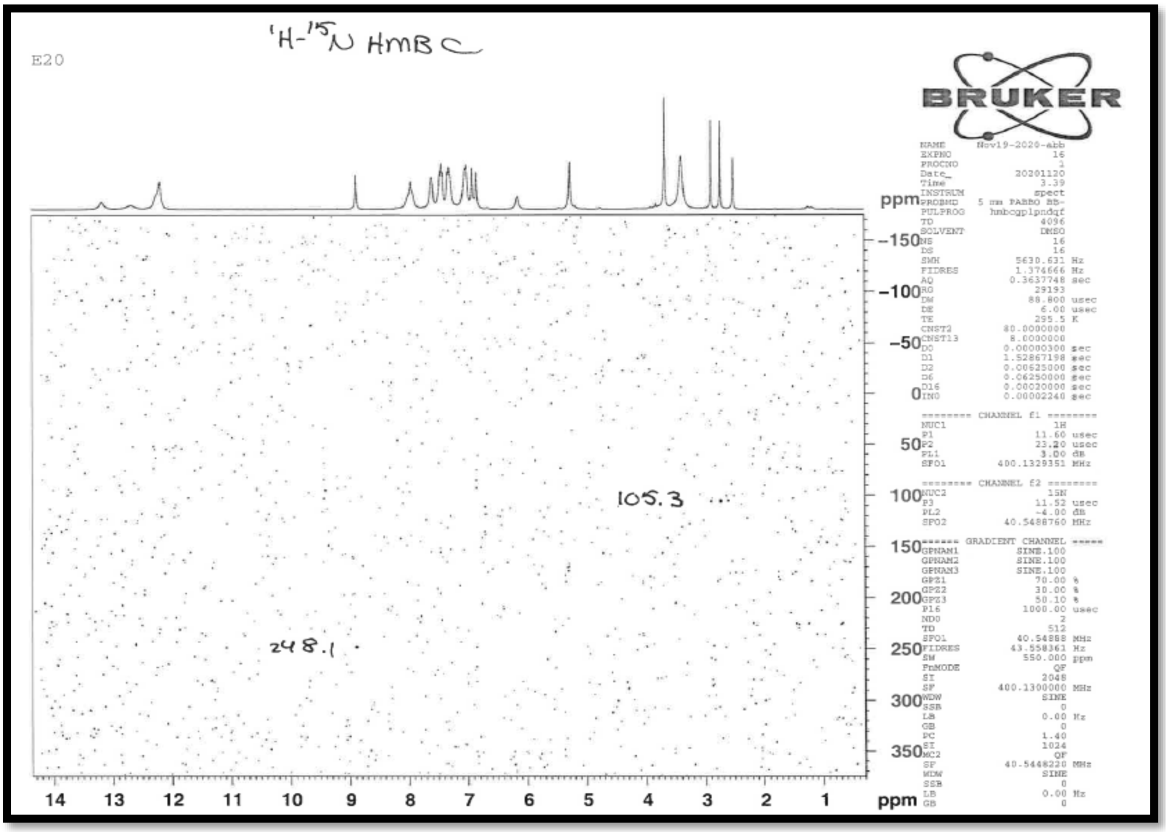
E20
1H-15N HMBC
BRUKER
105.3
248.1
ppm

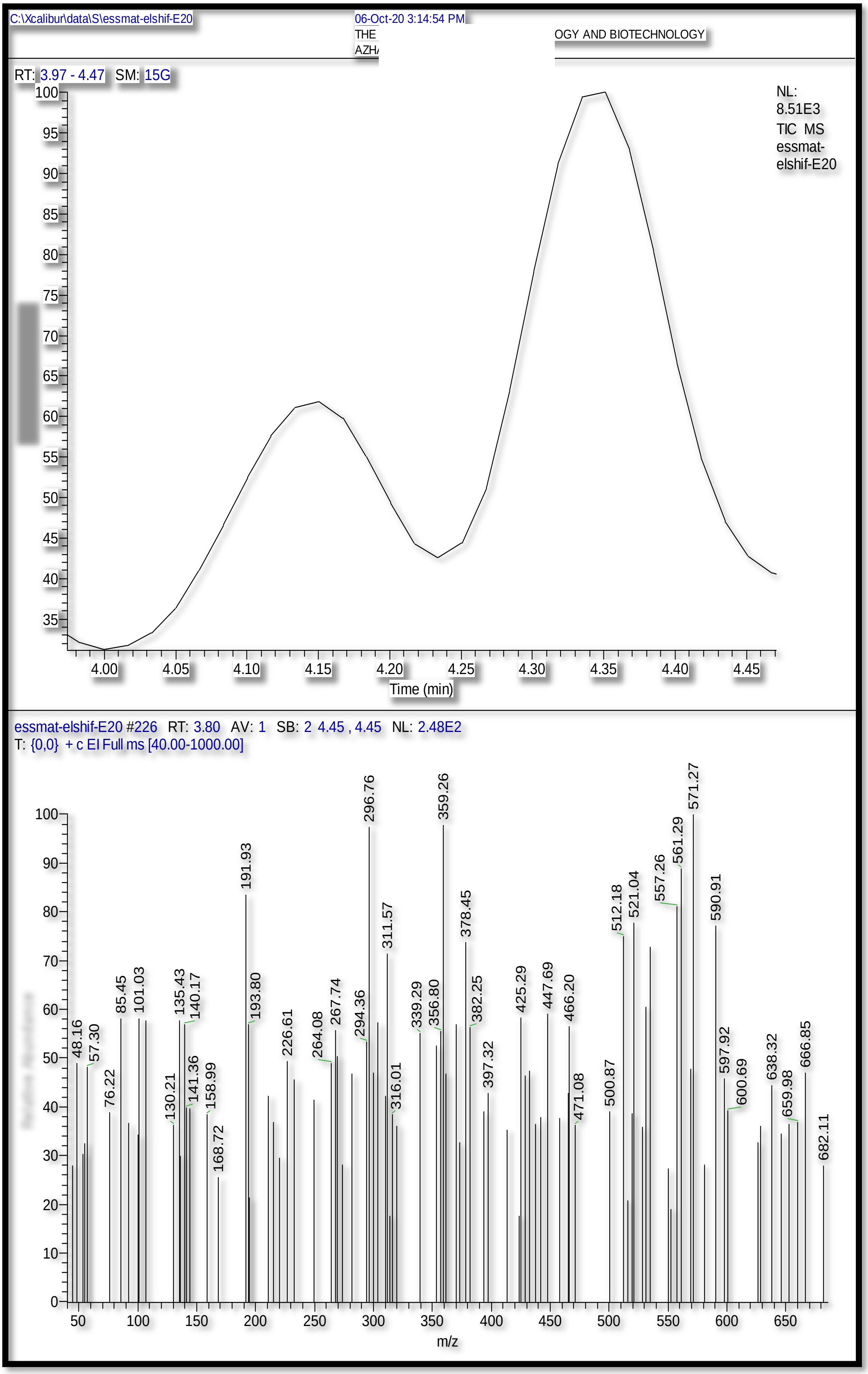

Spectrum data for Compound 8d

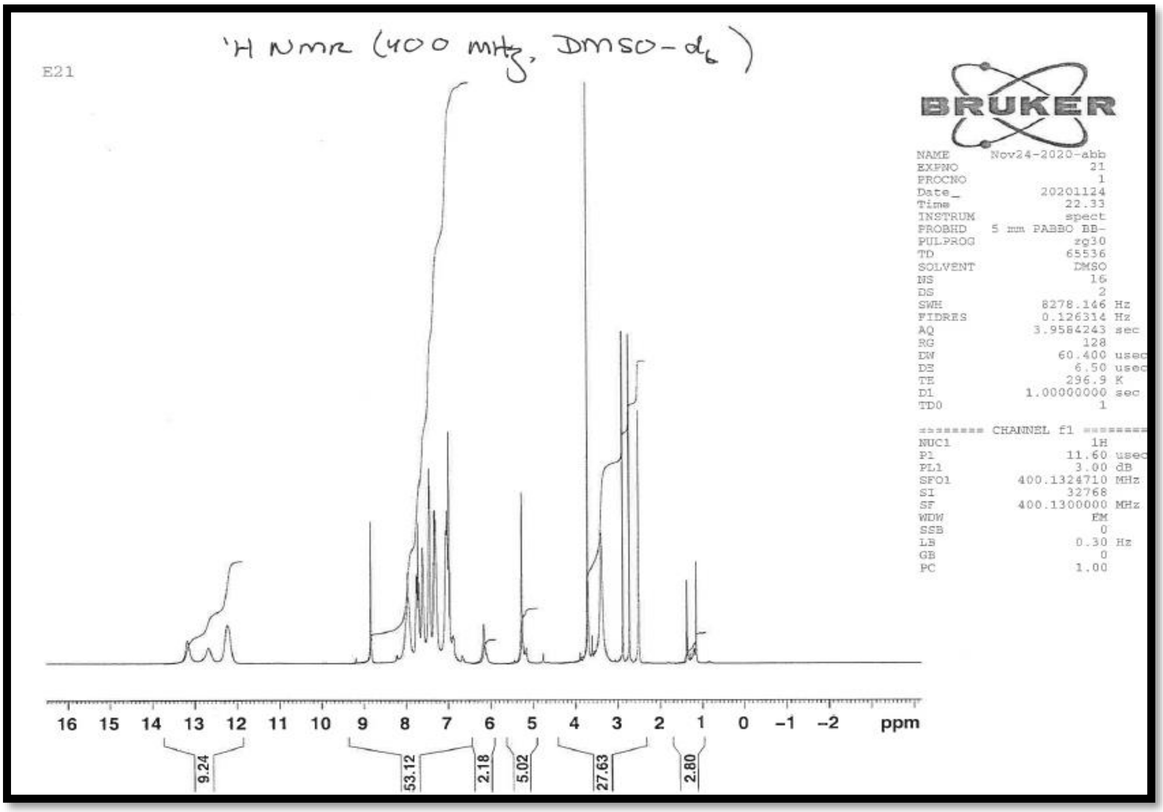

¹H NMR (400 MHz, DMSO-d6)
E21
BRUKER
NAME Nov24-2020-abb
EXPNO 21
PROCNO 1
Date_ 20201124
Time 22.33
INSTRUM spect
PROBHD 5 mm PABBO BB-
PULPROG zg30
TD 65536
SOLVENT DMSO
NS 16
DS 2
SWH 8278.146 Hz
FIDRES 0.126314 Hz
AQ 3.9584243 sec
RG 128
DW 60.400 usec
DE 6.50 usec
TE 296.9 K
D1 1.00000000 sec
TD0 1
======== CHANNEL f1 ========
NUC1 1H
P1 11.60 usec
PL1 3.00 dB
SFO1 400.1324710 MHz
SI 32768
SF 400.1300000 MHz
WDW EM
SSB 0
LB 0.30 Hz
GB 0
PC 1.00
16 15 14 13 12 11 10 9 8 7 6 5 4 3 2 1 0 −1 −2 ppm
9.24
53.12
2.18
5.02
27.63
2.80

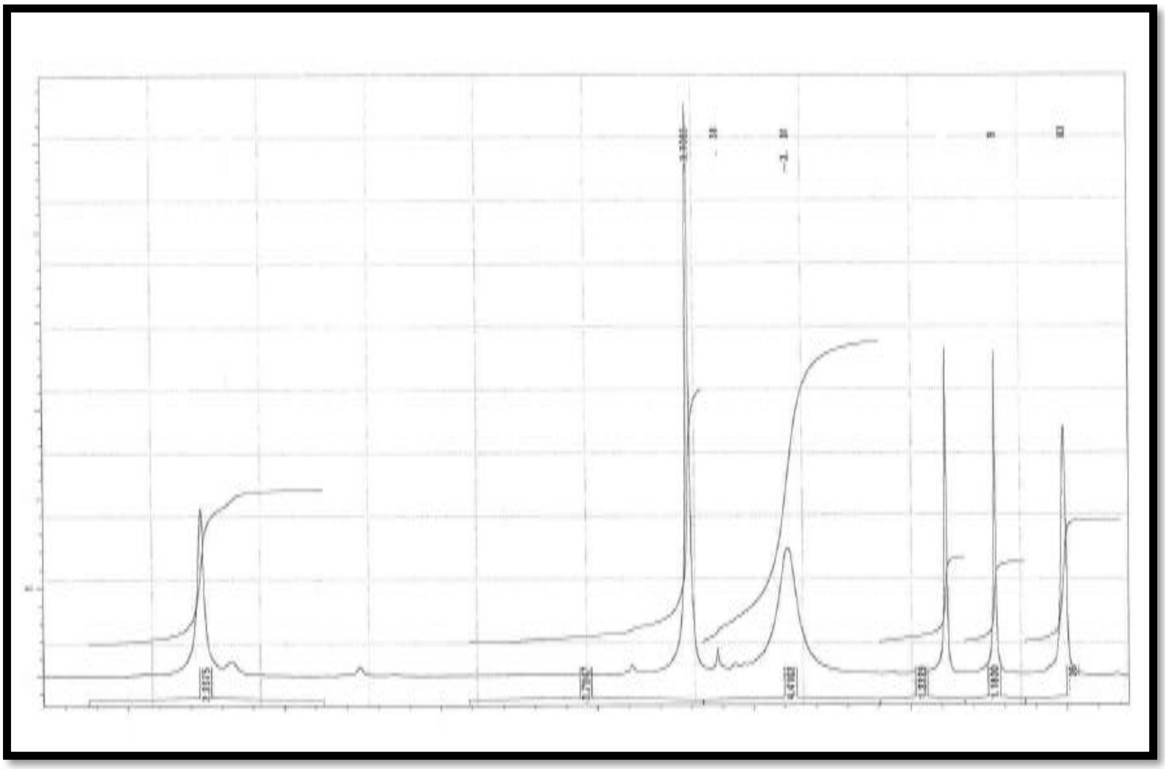

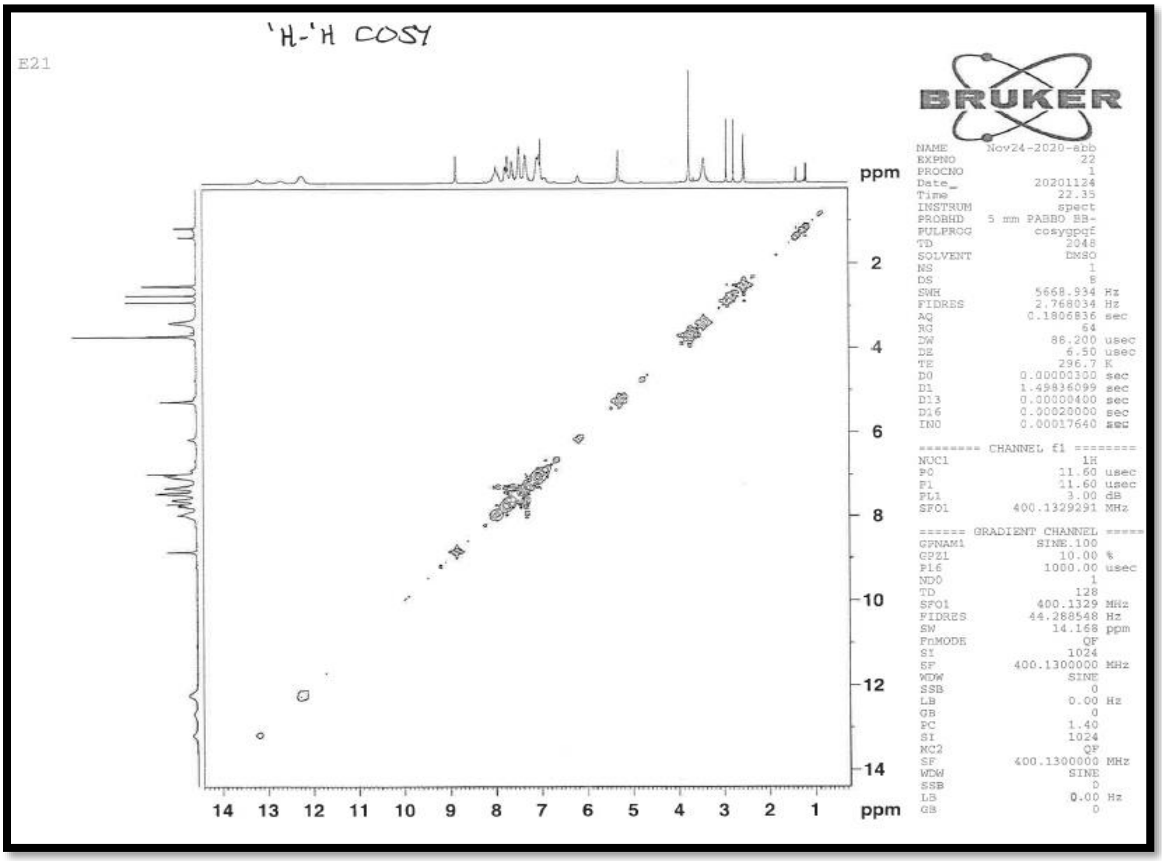

'H-'H COSY
E21
BRUKER
NAME Nov24-2020-abb
EXPNO 22
PROCNO 1
Date_ 20201124
Time 22.35
INSTRUM spect
PROBHD 5 mm PABBO BB-
PULPROG cosygpqf
TD 2048
SOLVENT DMSO
NS 1
DS 8
SWH 5668.934 Hz
FIDRES 2.768034 Hz
AQ 0.1806836 sec
RG 64
DW 88.200 usec
DE 6.50 usec
TE 296.7 K
D0 0.00000300 sec
D1 1.49836099 sec
D13 0.00000400 sec
D16 0.00020000 sec
IN0 0.00017640 sec
======== CHANNEL f1 ========
NUC1 1H
P0 11.60 usec
P1 11.60 usec
PL1 3.00 dB
SFO1 400.1329291 MHz
====== GRADIENT CHANNEL =====
GPNAM1 SINE.100
GPZ1 10.00 %
P16 1000.00 usec
ND0 1
TD 128
SFO1 400.1329 MHz
FIDRES 44.288548 Hz
SW 14.168 ppm
FnMODE QF
SI 1024
SF 400.1300000 MHz
WDW SINE
SSB 0
LB 0.00 Hz
GB 0
PC 1.40
SI 1024
MC2 QF
SF 400.1300000 MHz
WDW SINE
SSB 0
LB 0.00 Hz
GB 0
ppm
2
4
6
8
10
12
14
14 13 12 11 10 9 8 7 6 5 4 3 2 1 ppm

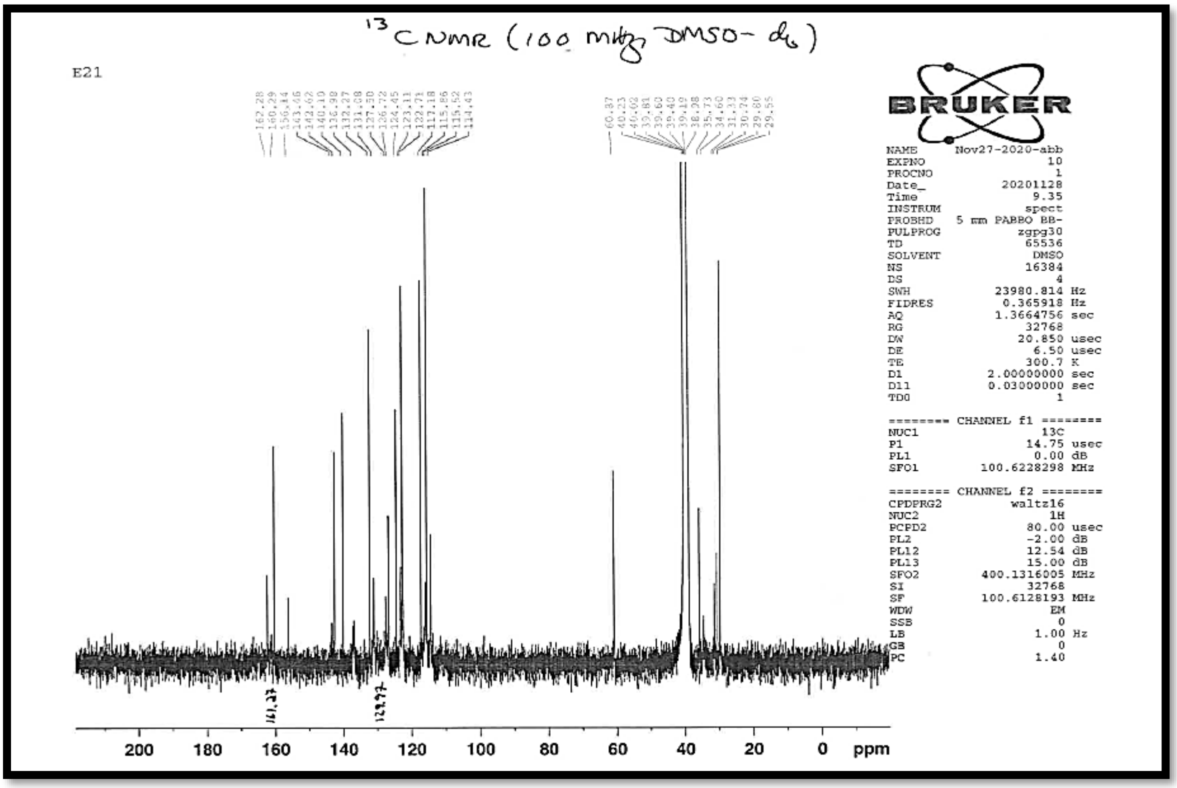

13C NMR (100 MHz DMSO-d6)
E21
162.28
160.29
156.44
143.46
142.62
140.10
136.98
132.27
131.08
127.50
126.72
124.45
123.11
122.71
117.18
115.86
115.52
114.43
60.37
40.23
40.02
39.81
39.60
39.40
39.19
38.98
35.73
34.60
31.33
30.74
29.80
29.55
BRUKER
NAME Nov27-2020-abb
EXPNO 10
PROCNO 1
Date_ 20201128
Time 9.35
INSTRUM spect
PROBHD 5 mm PABBO BB-
PULPROG zgpg30
TD 65536
SOLVENT DMSO
NS 16384
DS 4
SWH 23980.814 Hz
FIDRES 0.365918 Hz
AQ 1.3664756 sec
RG 32768
DW 20.850 usec
DE 6.50 usec
TE 300.7 K
D1 2.00000000 sec
D11 0.03000000 sec
TD0 1
======== CHANNEL f1 ========
NUC1 13C
P1 14.75 usec
PL1 0.00 dB
SFO1 100.6228298 MHz
======== CHANNEL f2 ========
CPDPRG2 waltz16
NUC2 1H
PCPD2 80.00 usec
PL2 -2.00 dB
PL12 12.54 dB
PL13 15.00 dB
SFO2 400.1316005 MHz
SI 32768
SF 100.6128193 MHz
WDW EM
SSB 0
LB 1.00 Hz
GB 0
PC 1.40
161.27
129.97
200 180 160 140 120 100 80 60 40 20 0 ppm

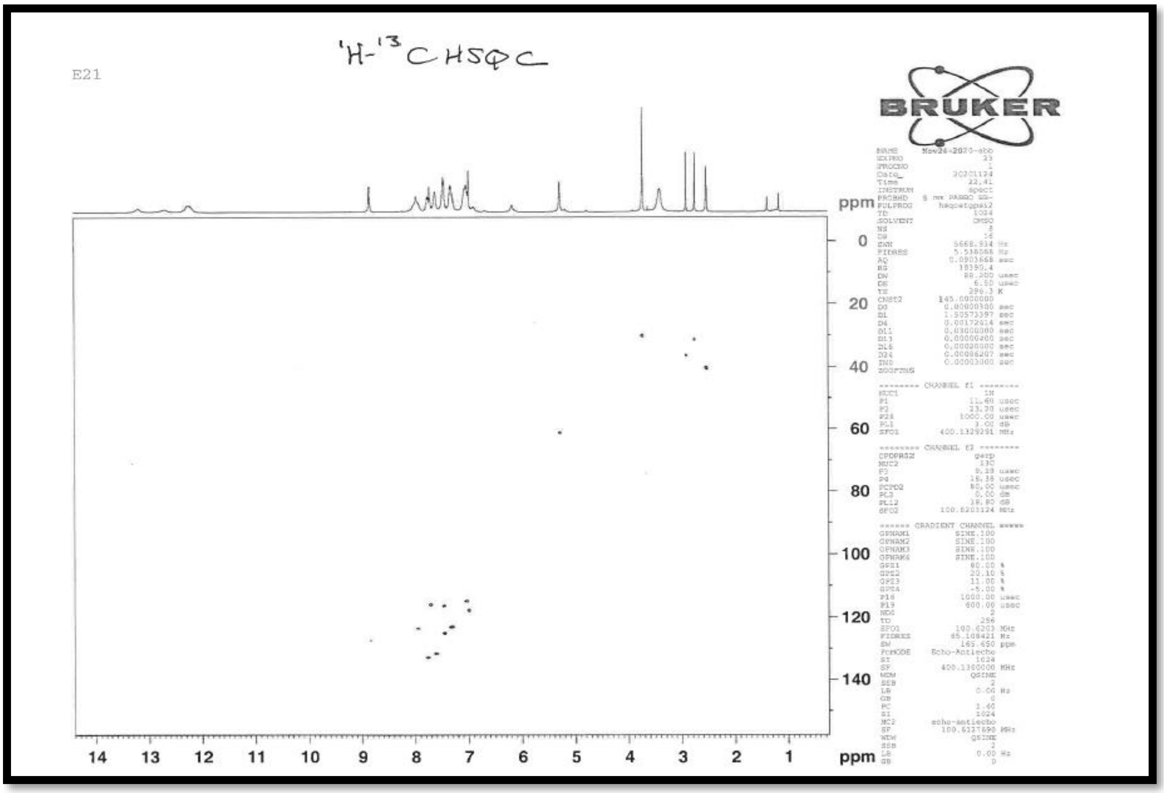
E21
1H-13C HSQC
BRUKER
ppm

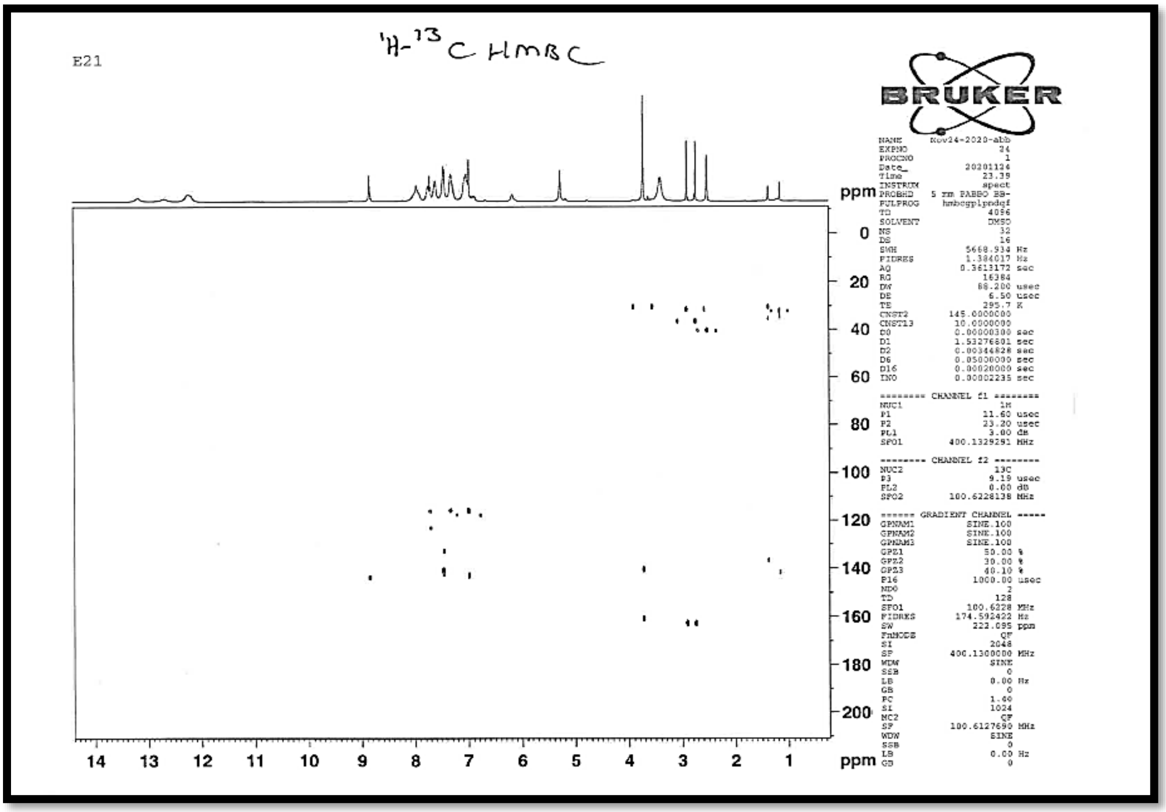
E21
1H-13C HMBC
BRUKER
ppm

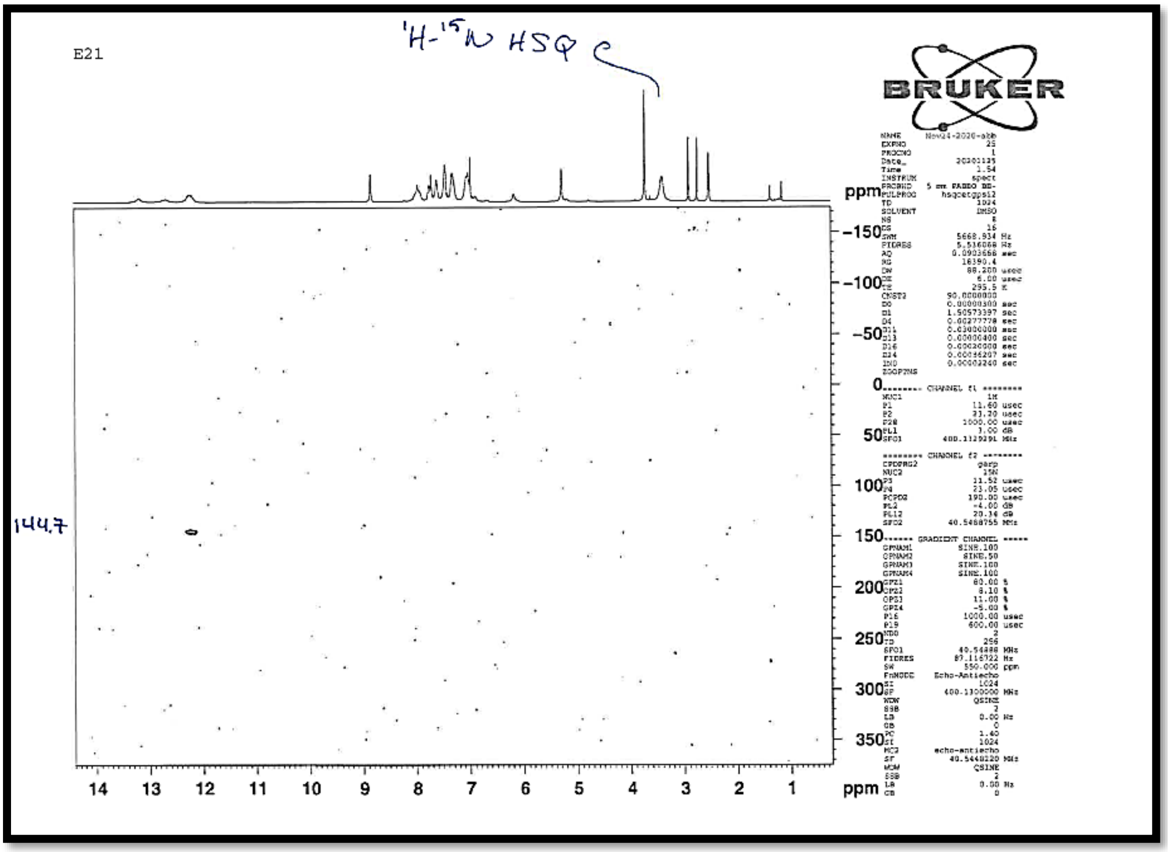
E21
1H-15N HSQC
144.7
BRUKER

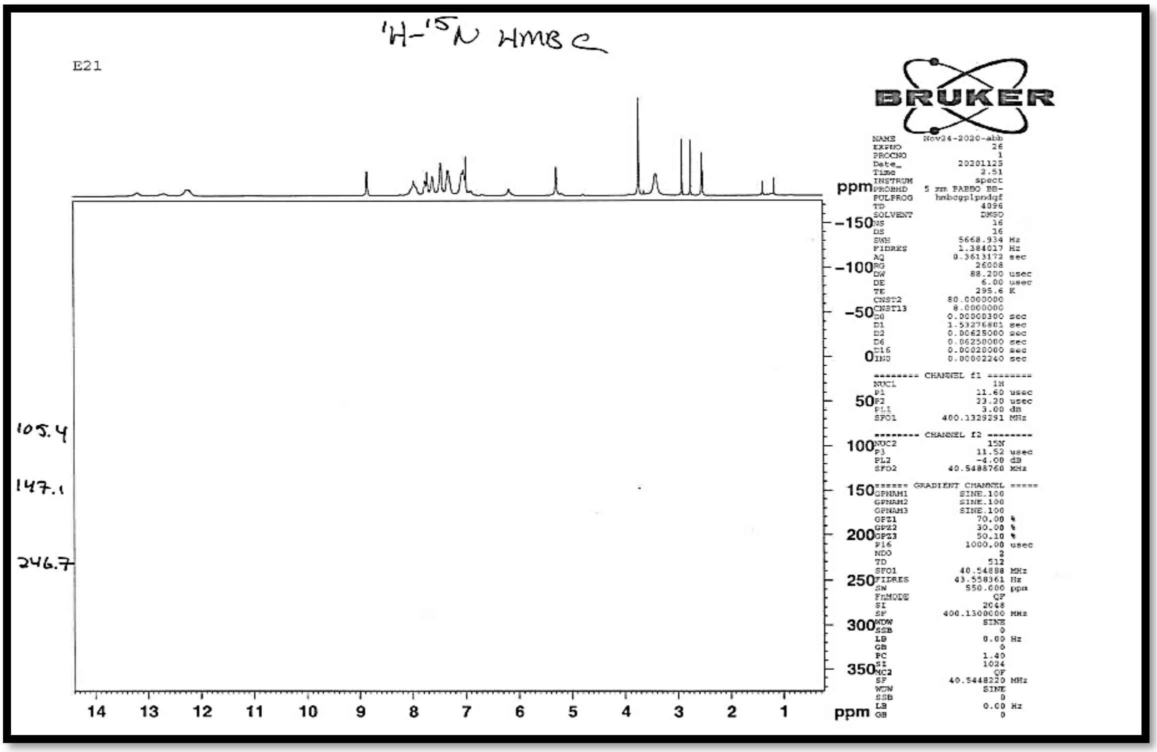
E21
1H-15N HMBC
105.4
147.1
246.7
BRUKER

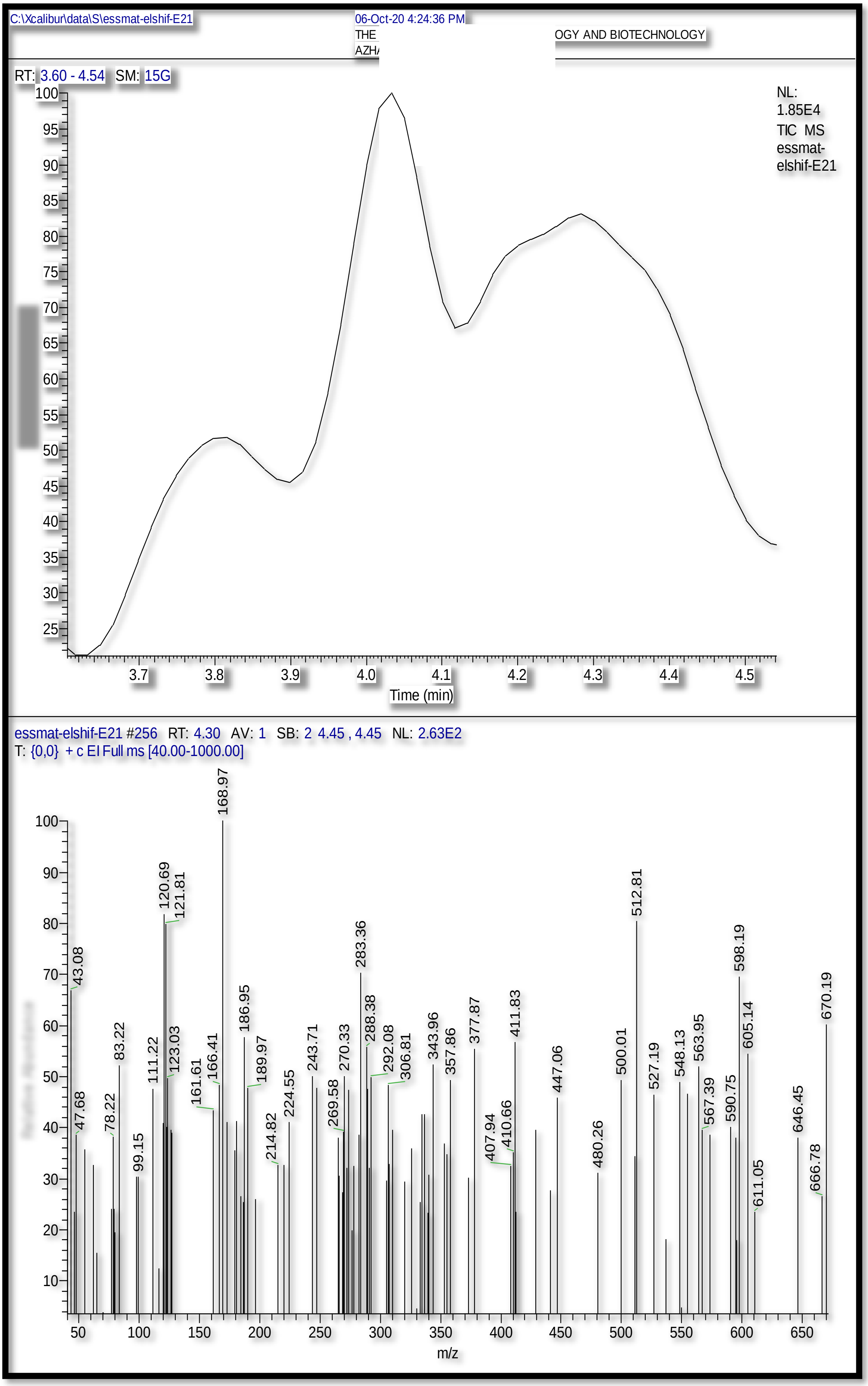

Spectrum data for Compound 8e

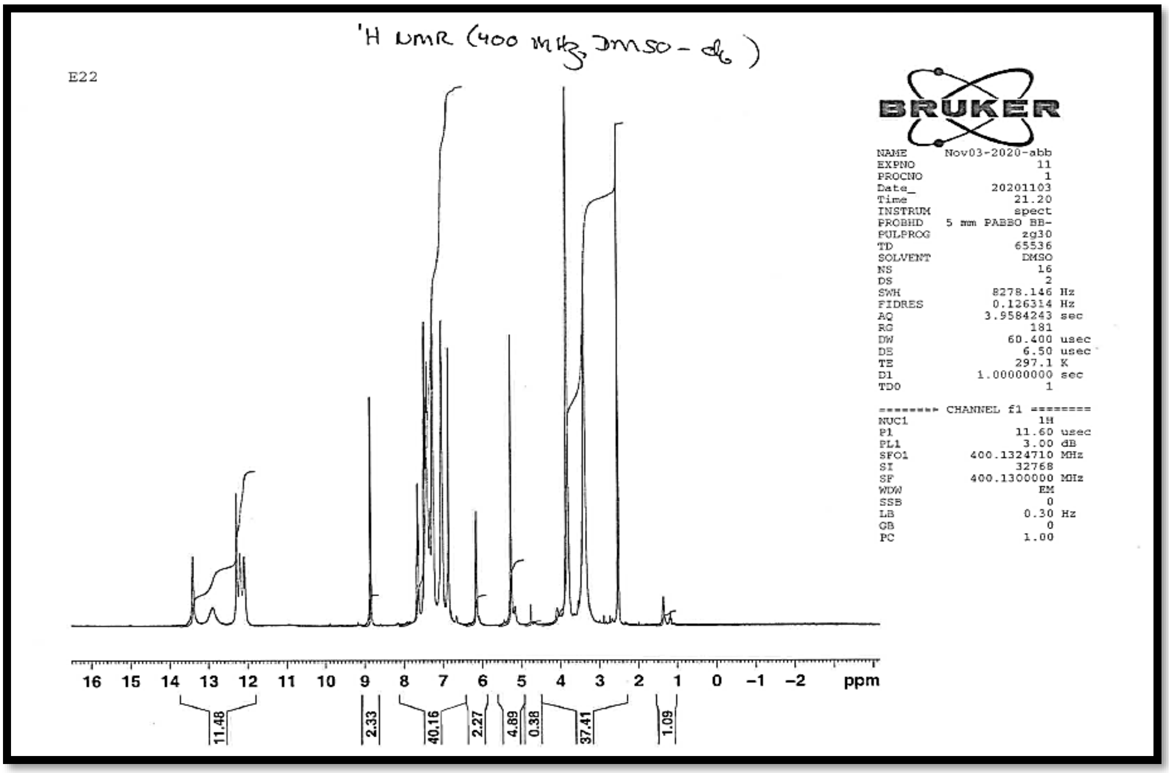

1H NMR (400 MHz, DMSO-d6)
E22
BRUKER
NAME Nov03-2020-abb
EXPNO 11
PROCNO 1
Date_ 20201103
Time 21.20
INSTRUM spect
PROBHD 5 mm PABBO BB-
PULPROG zg30
TD 65536
SOLVENT DMSO
NS 16
DS 2
SWH 8278.146 Hz
FIDRES 0.126314 Hz
AQ 3.9584243 sec
RG 181
DW 60.400 usec
DE 6.50 usec
TE 297.1 K
D1 1.00000000 sec
TD0 1
======== CHANNEL f1 ========
NUC1 1H
P1 11.60 usec
PL1 3.00 dB
SFO1 400.1324710 MHz
SI 32768
SF 400.1300000 MHz
WDW EM
SSB 0
LB 0.30 Hz
GB 0
PC 1.00
16 15 14 13 12 11 10 9 8 7 6 5 4 3 2 1 0 -1 -2 ppm
11.48
2.33
40.16
2.27
4.89
0.38
37.41
1.09

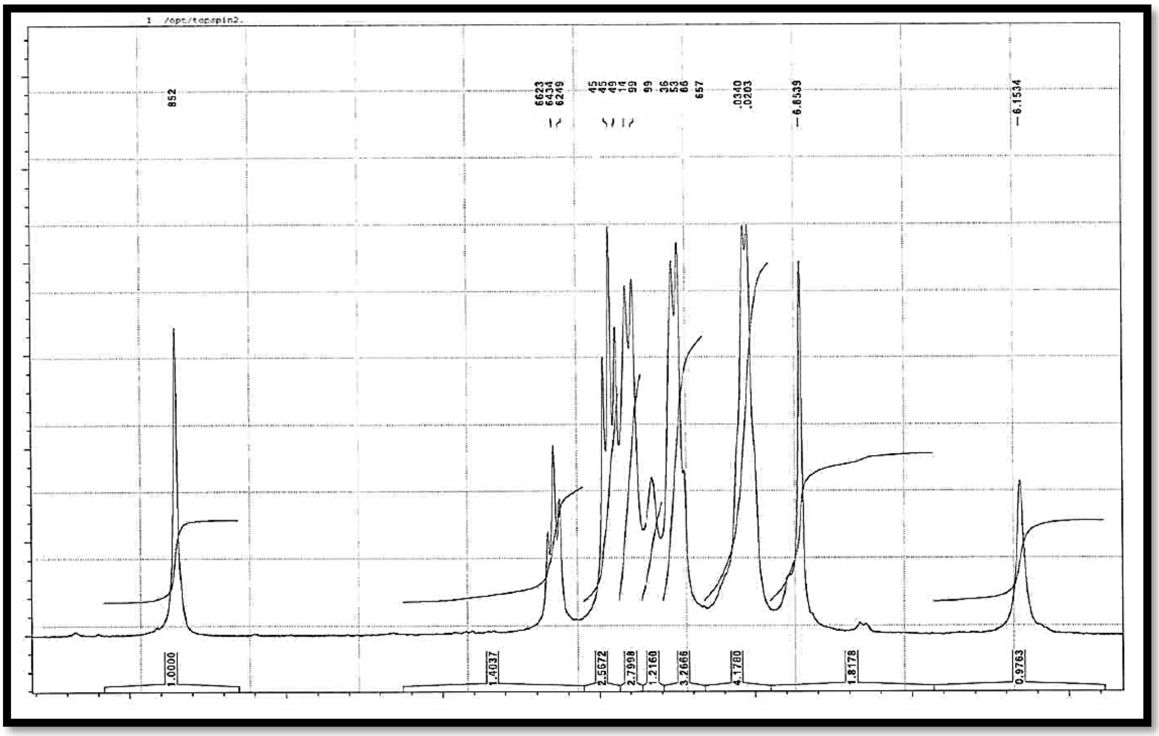

1 /opt/topspin2.
852
6623
6434
6249
-6.8539
-6.1534
1.0000
1.4037
2.5672
2.7998
1.2160
3.2666
4.1780
1.8178
0.9763

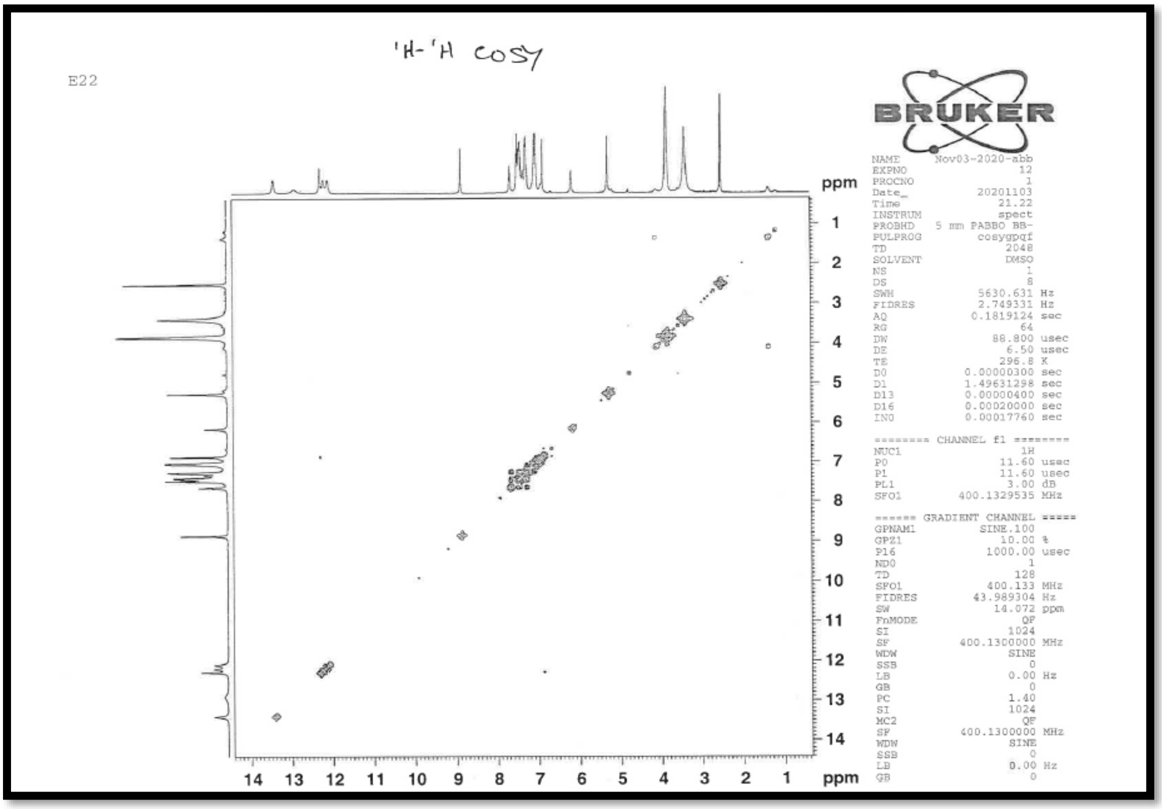
1H-1H COSY
E22
ppm
14 13 12 11 10 9 8 7 6 5 4 3 2 1 ppm
BRUKER
NAME Nov03-2020-abb
EXPNO 12
PROCNO 1
Date_ 20201103
Time 21.22
INSTRUM spect
PROBHD 5 mm PABBO BB-
PULPROG cosygpqf
TD 2048
SOLVENT DMSO
NS 1
DS 8
SWH 5630.631 Hz
FIDRES 2.749331 Hz
AQ 0.1819124 sec
RG 64
DW 88.800 usec
DE 6.50 usec
TE 296.8 K
D0 0.00000300 sec
D1 1.49631298 sec
D13 0.00000400 sec
D16 0.00020000 sec
IN0 0.00017760 sec
======== CHANNEL f1 ========
NUC1 1H
P0 11.60 usec
P1 11.60 usec
PL1 3.00 dB
SFO1 400.1329535 MHz
====== GRADIENT CHANNEL =====
GPNAM1 SINE.100
GPZ1 10.00 %
P16 1000.00 usec
ND0 1
TD 128
SFO1 400.133 MHz
FIDRES 43.989304 Hz
SW 14.072 ppm
FnMODE QF
SI 1024
SF 400.1300000 MHz
WDW SINE
SSB 0
LB 0.00 Hz
GB 0
PC 1.40
SI 1024
MC2 QF
SF 400.1300000 MHz
WDW SINE
SSB 0
LB 0.00 Hz
GB 0

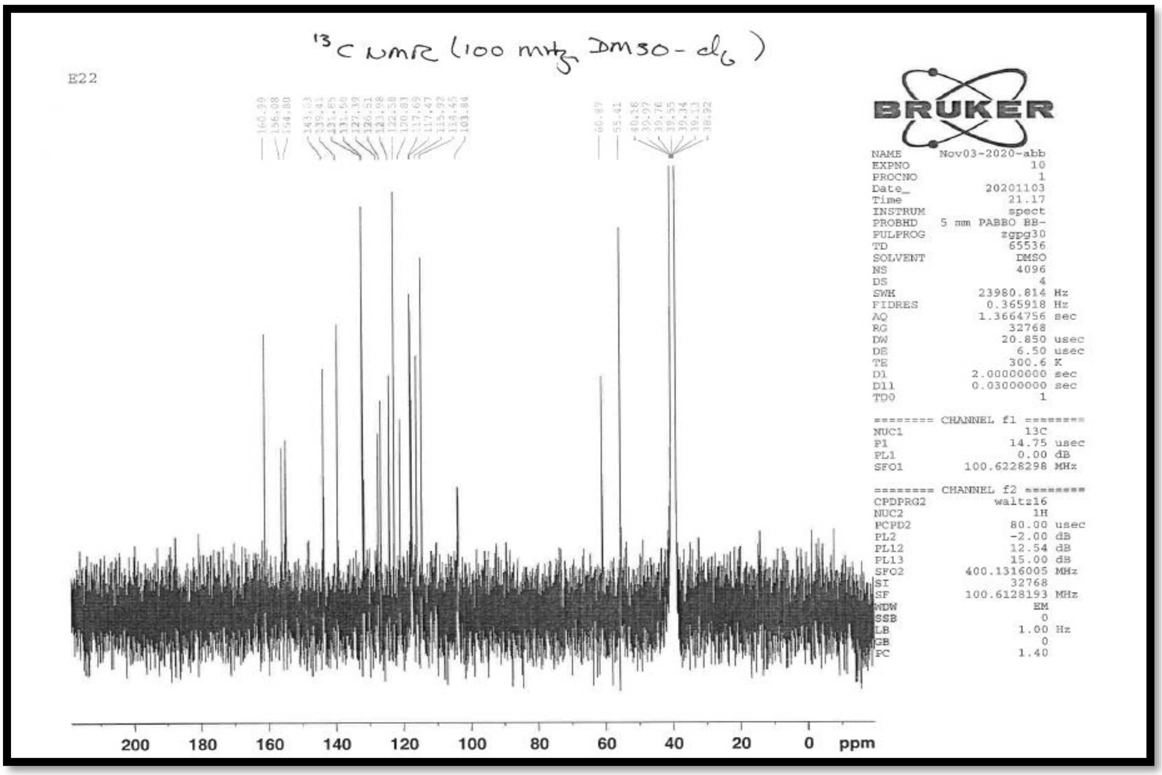
13C NMR (100 MHz, DMSO-d6)
E22
160.99
156.08
154.80
143.63
139.41
131.85
131.50
127.39
126.51
123.98
122.58
120.83
117.69
117.47
115.92
114.45
103.84
60.87
55.41
40.18
39.97
39.76
39.55
39.34
39.13
38.92
200 180 160 140 120 100 80 60 40 20 0 ppm
BRUKER
NAME Nov03-2020-abb
EXPNO 10
PROCNO 1
Date_ 20201103
Time 21.17
INSTRUM spect
PROBHD 5 mm PABBO BB-
PULPROG zgpg30
TD 65536
SOLVENT DMSO
NS 4096
DS 4
SWH 23980.814 Hz
FIDRES 0.365918 Hz
AQ 1.3664756 sec
RG 32768
DW 20.850 usec
DE 6.50 usec
TE 300.6 K
D1 2.00000000 sec
D11 0.03000000 sec
TD0 1
======== CHANNEL f1 ========
NUC1 13C
P1 14.75 usec
PL1 0.00 dB
SFO1 100.6228298 MHz
======== CHANNEL f2 ========
CPDPRG2 waltz16
NUC2 1H
PCPD2 80.00 usec
PL2 -2.00 dB
PL12 12.54 dB
PL13 15.00 dB
SFO2 400.1316005 MHz
SI 32768
SF 100.6128193 MHz
WDW EM
SSB 0
LB 1.00 Hz
GB 0
PC 1.40

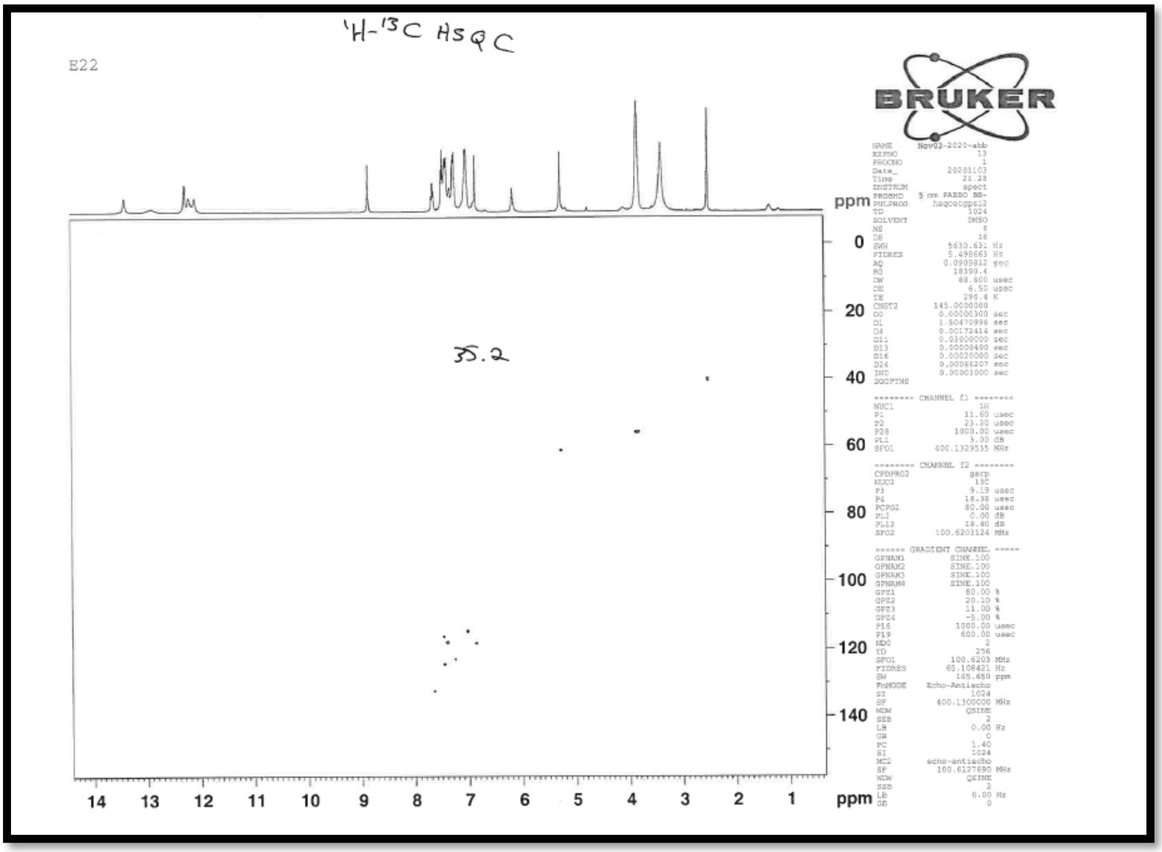
1H-13C HSQC
E22
BRUKER
35.2
ppm

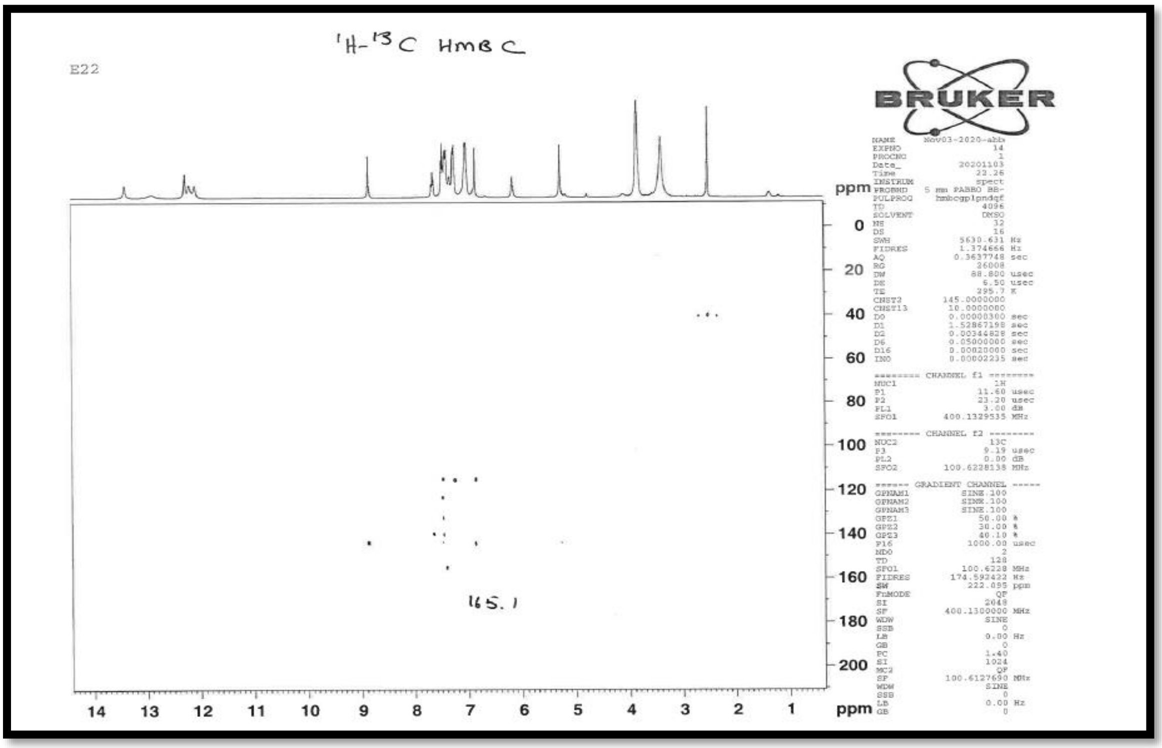
1H-13C HMBC
E22
BRUKER
165.1
ppm

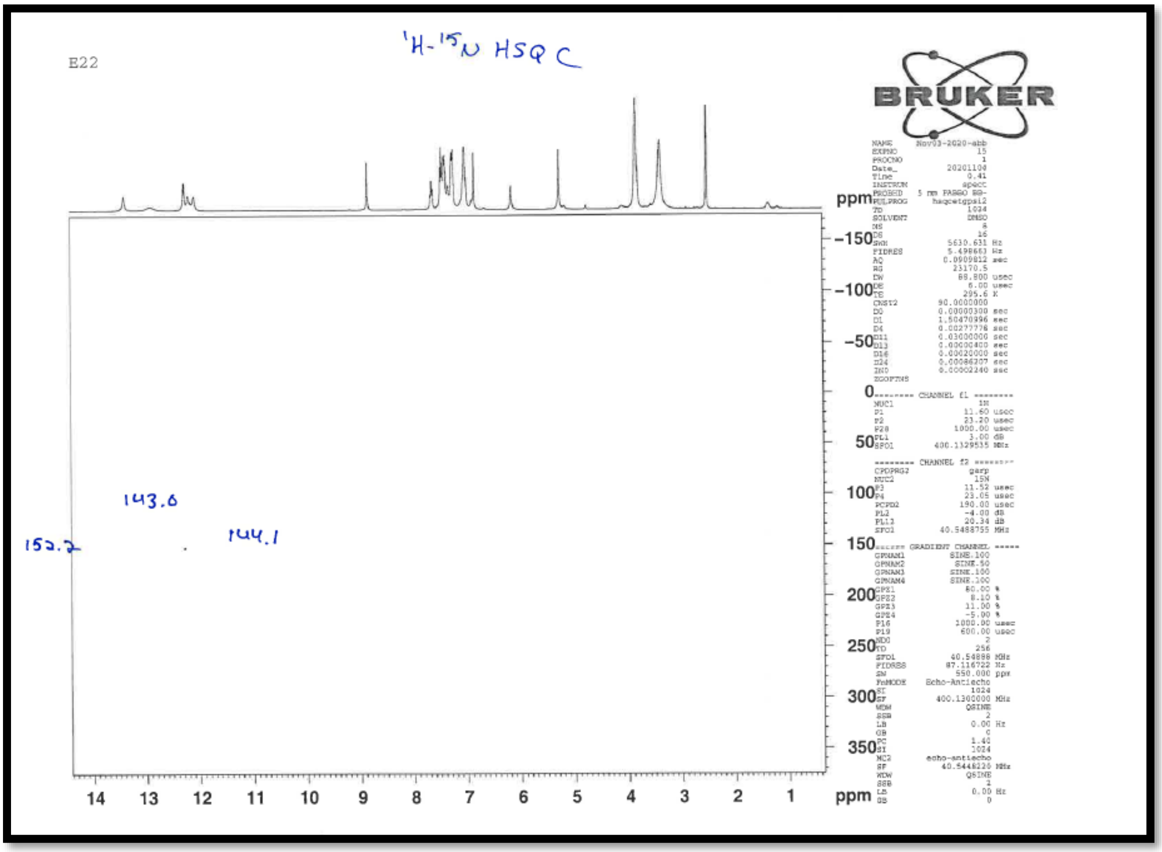
E22
1H-15N HSQC
143.6
144.1
152.2

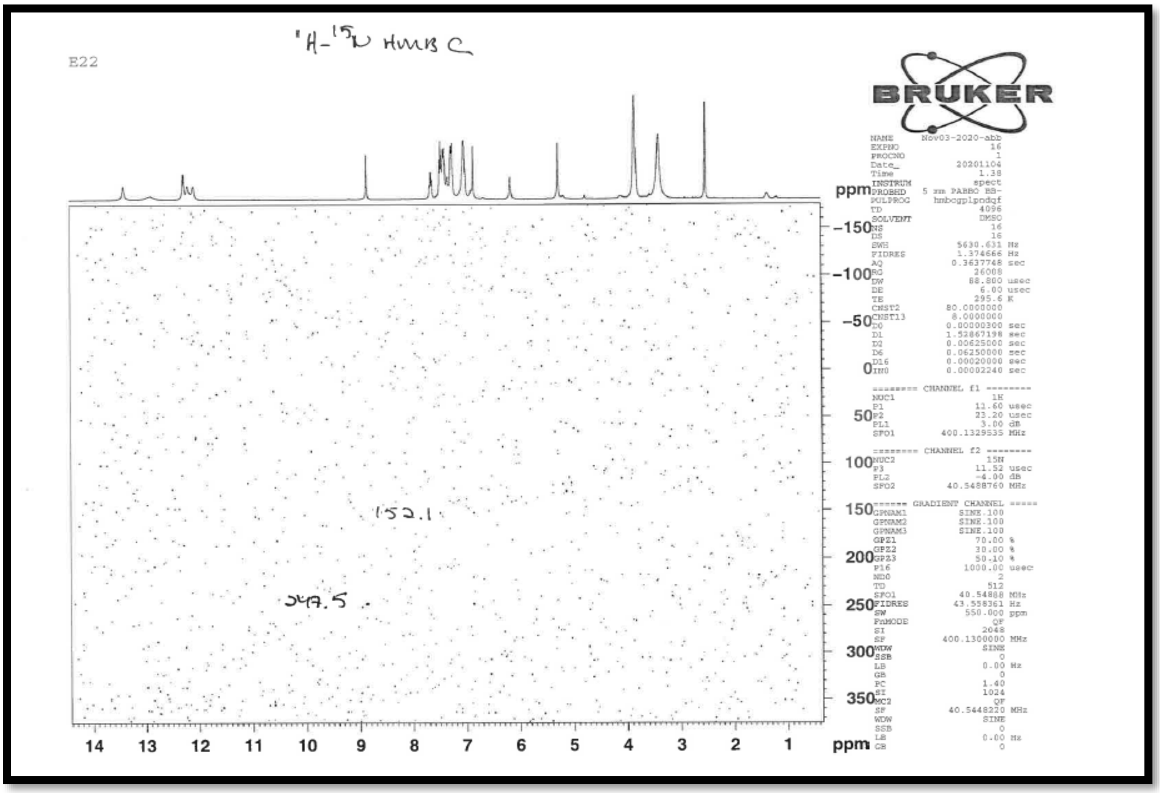
E22
1H-15N HMBC
152.1
249.5

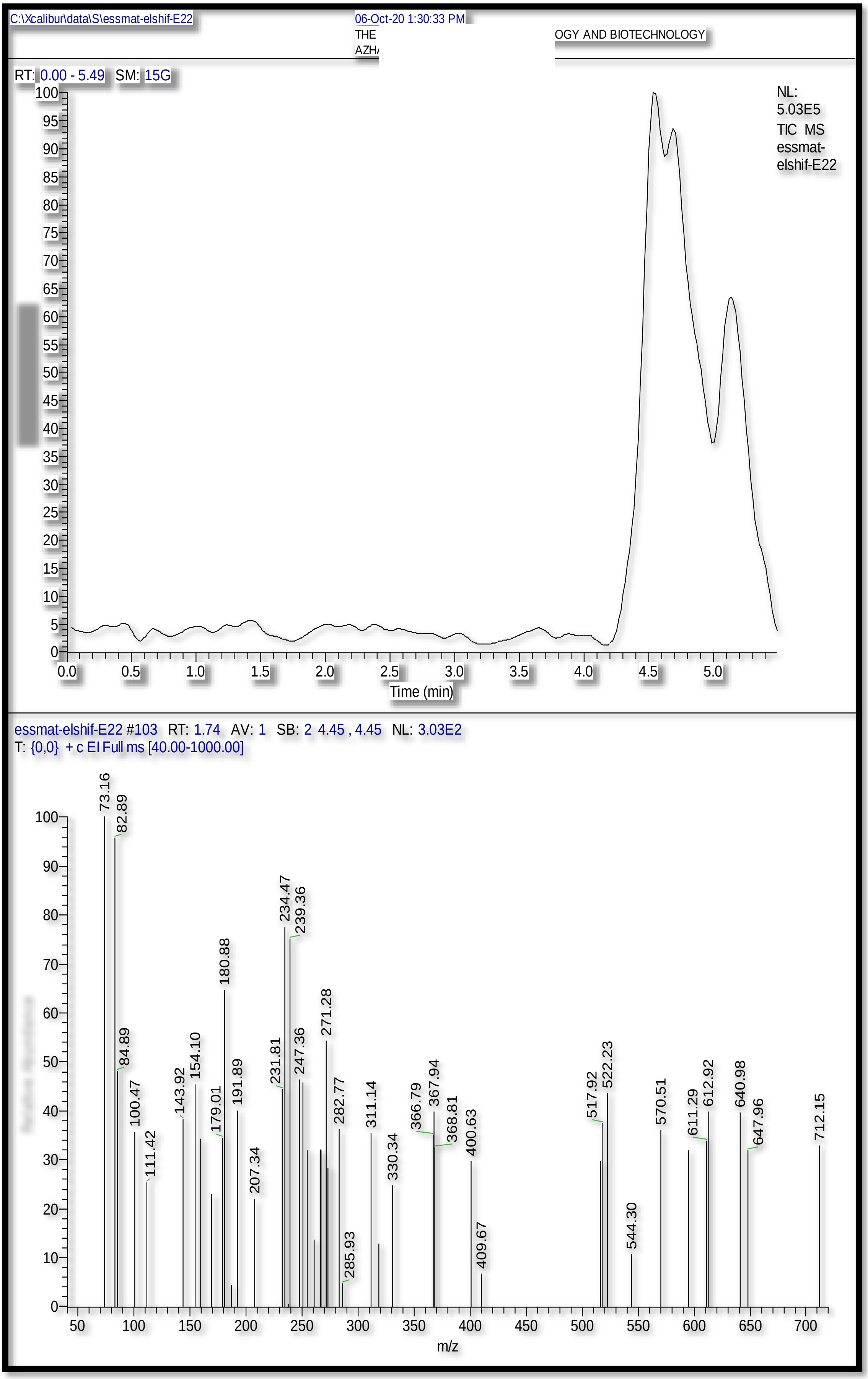

Spectrum data for Compound 8f

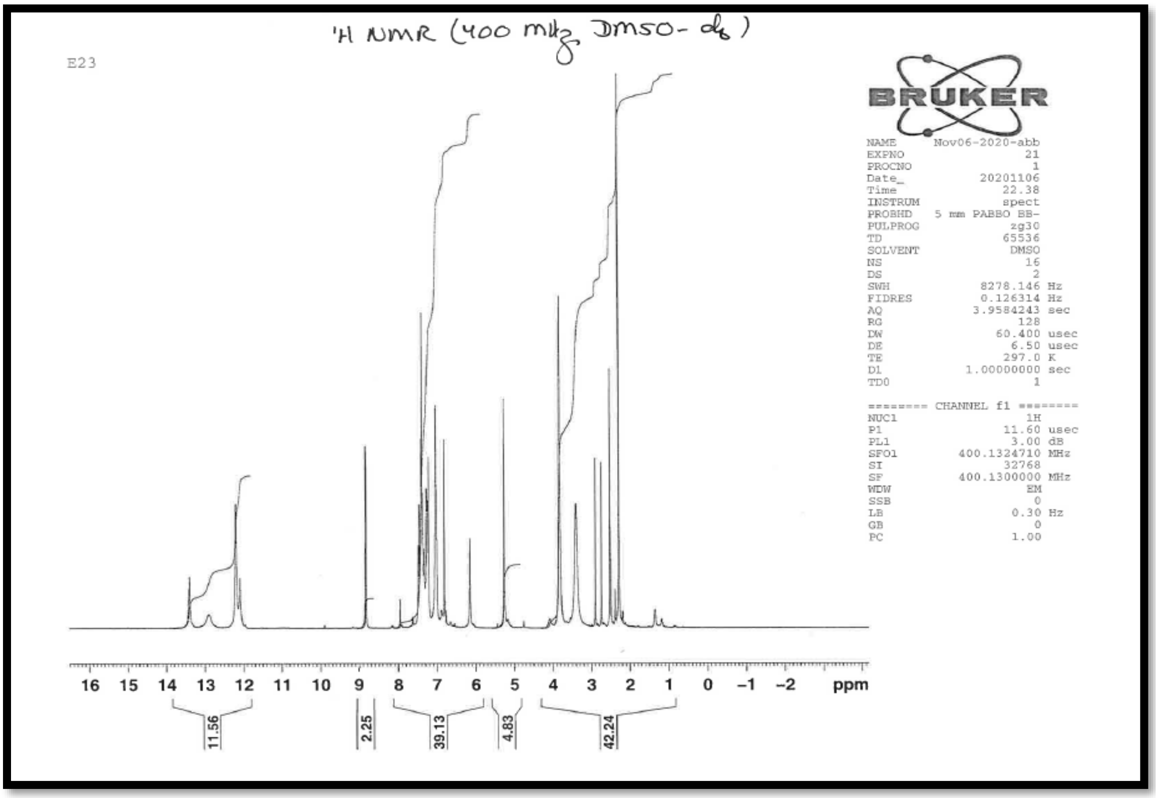
¹H NMR (400 MHz, DMSO-d6)
E23
BRUKER
NAME Nov06-2020-abb
EXPNO 21
PROCNO 1
Date_ 20201106
Time 22.38
INSTRUM spect
PROBHD 5 mm PABBO BB-
PULPROG zg30
TD 65536
SOLVENT DMSO
NS 16
DS 2
SWH 8278.146 Hz
FIDRES 0.126314 Hz
AQ 3.9584243 sec
RG 128
DW 60.400 usec
DE 6.50 usec
TE 297.0 K
D1 1.00000000 sec
TD0 1
======== CHANNEL f1 ========
NUC1 1H
P1 11.60 usec
PL1 3.00 dB
SFO1 400.1324710 MHz
SI 32768
SF 400.1300000 MHz
WDW EM
SSB 0
LB 0.30 Hz
GB 0
PC 1.00
16 15 14 13 12 11 10 9 8 7 6 5 4 3 2 1 0 −1 −2 ppm
11.56
2.25
39.13
4.83
42.24

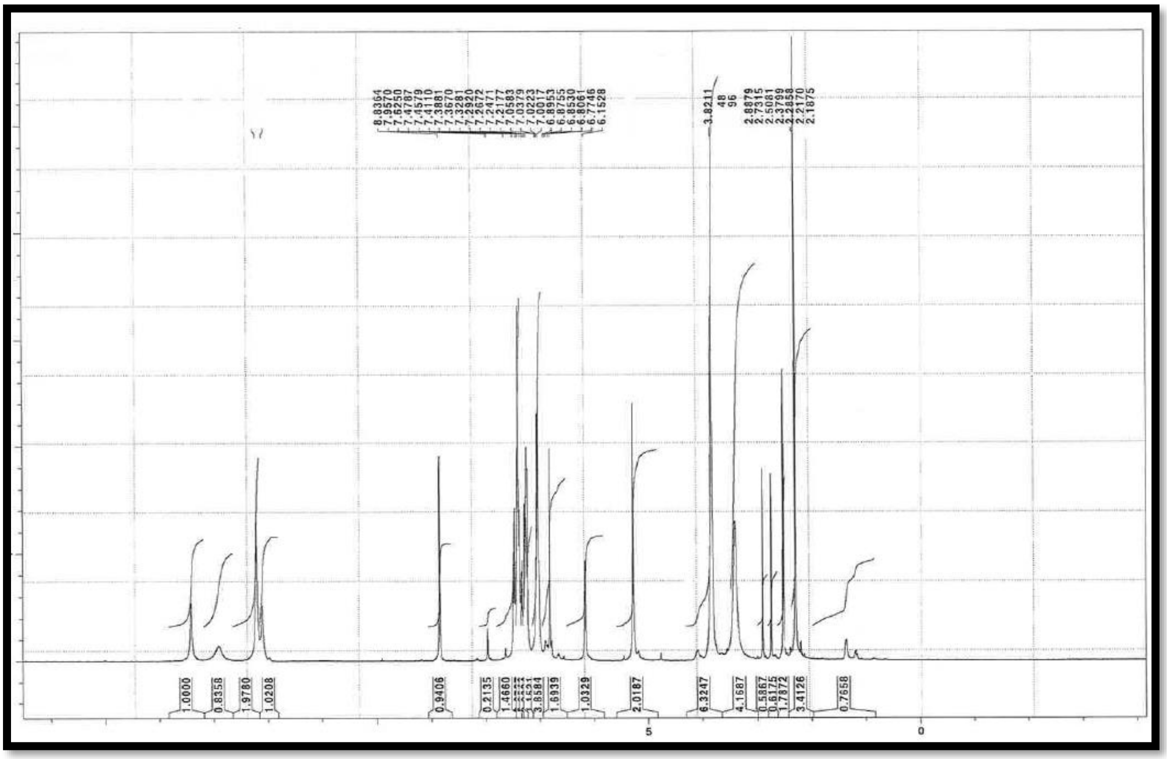
8.8364
7.9570
7.6250
7.4787
7.4579
7.4110
7.3881
7.3670
7.3281
7.2920
7.2672
7.2471
7.2177
7.0583
7.0379
7.0223
7.0017
6.8953
6.8755
6.8530
6.8061
6.7746
6.1528
3.8211
2.8879
2.7315
2.5081
2.3799
2.2848
2.2170
2.1875
1.0000
0.8358
1.9780
1.0208
0.9406
0.2135
1.4660
3.8584
1.6939
1.0329
2.0187
6.3247
4.1687
0.5867
0.6175
1.7872
3.4126
0.7658
5
0

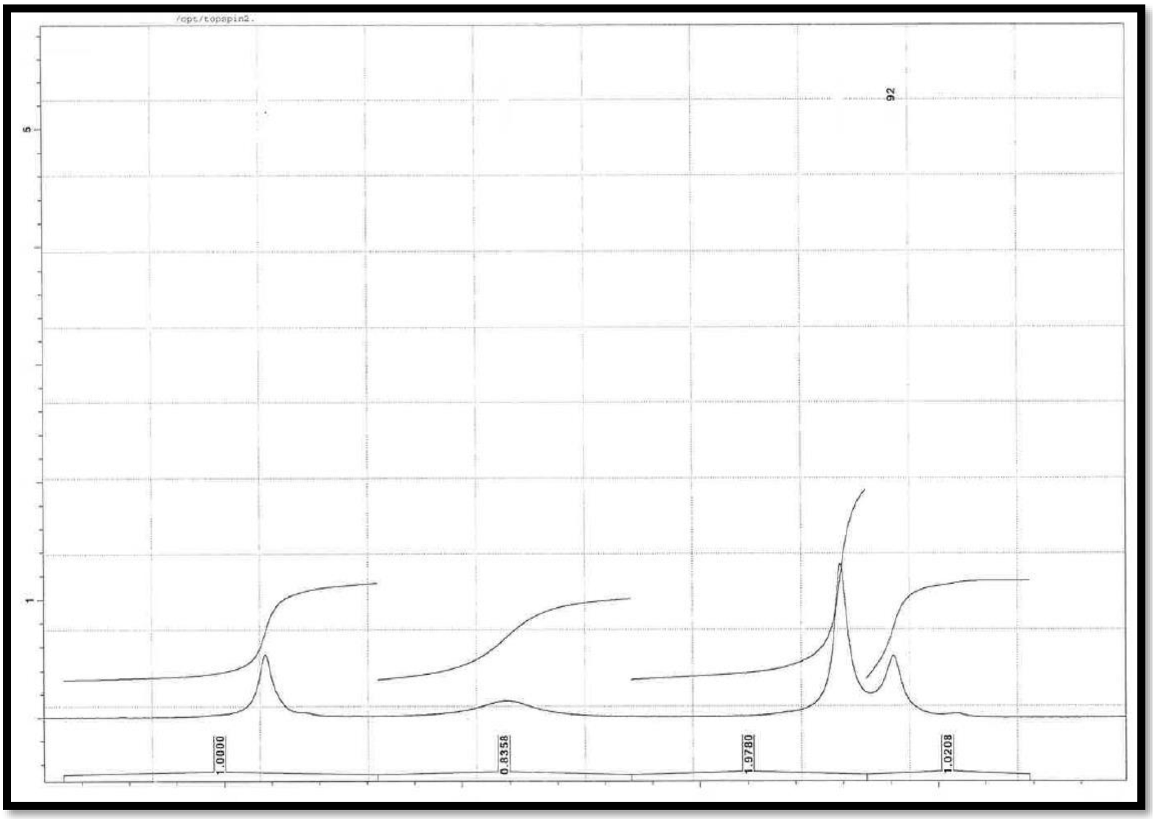
/opt/topspin2.
1.0000
0.8358
1.9780
1.0208

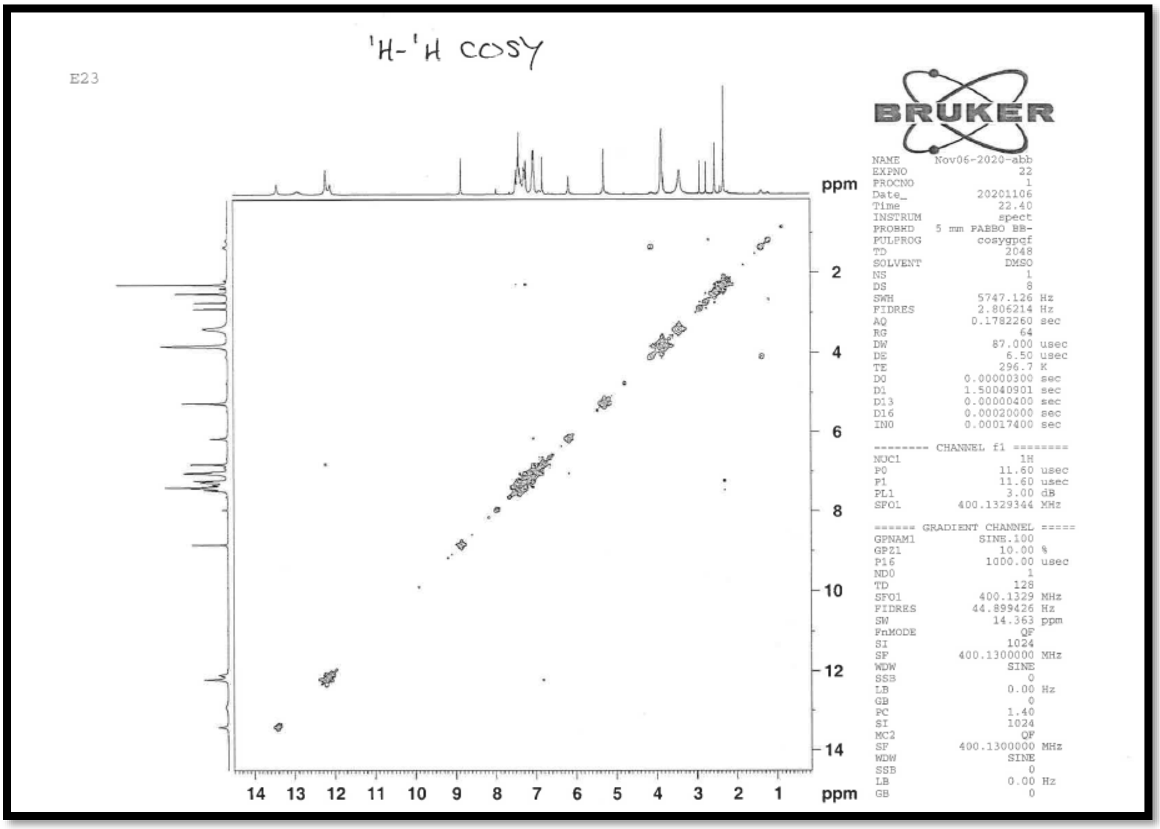
'H-'H COSY
E23
BRUKER
NAME Nov06-2020-abb
EXPNO 22
PROCNO 1
Date_ 20201106
Time 22.40
INSTRUM spect
PROBHD 5 mm PABBO BB-
PULPROG cosygpqf
TD 2048
SOLVENT DMSO
NS 1
DS 8
SWH 5747.126 Hz
FIDRES 2.806214 Hz
AQ 0.1782260 sec
RG 64
DW 87.000 usec
DE 6.50 usec
TE 296.7 K
D0 0.00000300 sec
D1 1.50040901 sec
D13 0.00000400 sec
D16 0.00020000 sec
IN0 0.00017400 sec
======== CHANNEL f1 ========
NUC1 1H
P0 11.60 usec
P1 11.60 usec
PL1 3.00 dB
SFO1 400.1329344 MHz
====== GRADIENT CHANNEL =====
GPNAM1 SINE.100
GPZ1 10.00 %
P16 1000.00 usec
ND0 1
TD 128
SFO1 400.1329 MHz
FIDRES 44.899426 Hz
SW 14.363 ppm
FnMODE QF
SI 1024
SF 400.1300000 MHz
WDW SINE
SSB 0
LB 0.00 Hz
GB 0
PC 1.40
SI 1024
MC2 QF
SF 400.1300000 MHz
WDW SINE
SSB 0
LB 0.00 Hz
GB 0
ppm
14 13 12 11 10 9 8 7 6 5 4 3 2 1 ppm

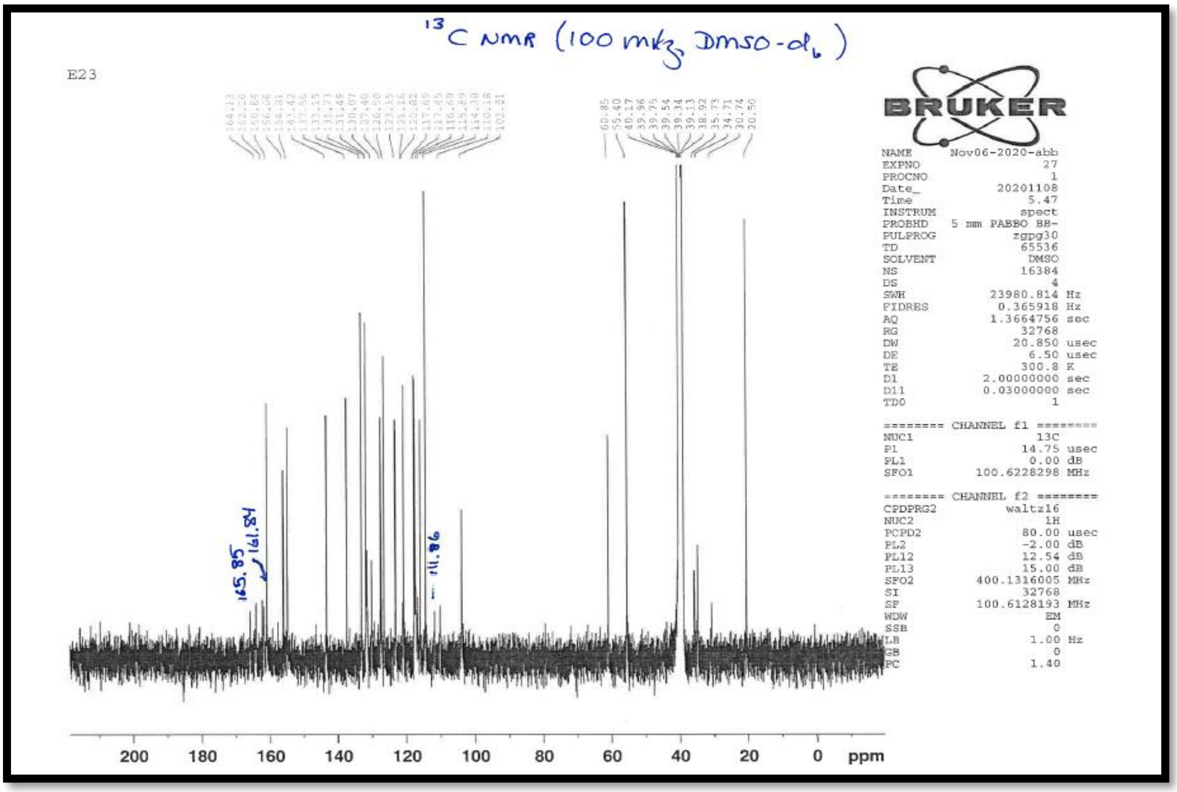

13C NMR (100 mHz, DMSO-d6)
E23
165.85
161.84
111.86
NAME Nov06-2020-abb
EXPNO 27
PROCNO 1
Date_ 20201108
Time 5.47
INSTRUM spect
PROBHD 5 mm PABBO BB-
PULPROG zgpg30
TD 65536
SOLVENT DMSO
NS 16384
DS 4
SWH 23980.814 Hz
FIDRES 0.365918 Hz
AQ 1.3664756 sec
RG 32768
DW 20.850 usec
DE 6.50 usec
TE 300.8 K
D1 2.00000000 sec
D11 0.03000000 sec
TD0 1
======== CHANNEL f1 ========
NUC1 13C
P1 14.75 usec
PL1 0.00 dB
SFO1 100.6228298 MHz
======== CHANNEL f2 ========
CPDPRG2 waltz16
NUC2 1H
PCPD2 80.00 usec
PL2 -2.00 dB
PL12 12.54 dB
PL13 15.00 dB
SFO2 400.1316005 MHz
SI 32768
SF 100.6128193 MHz
WDW EM
SSB 0
LB 1.00 Hz
GB 0
PC 1.40
ppm

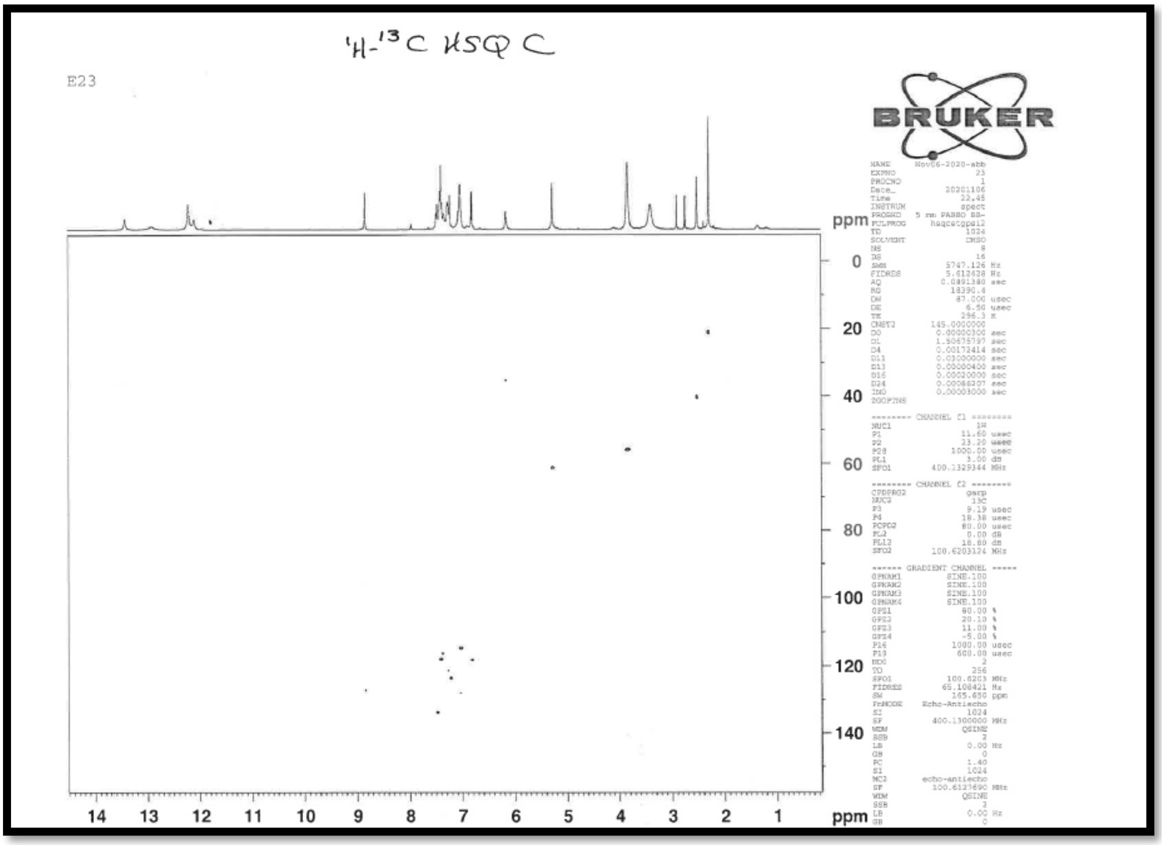

1H-13C HSQC
E23
ppm

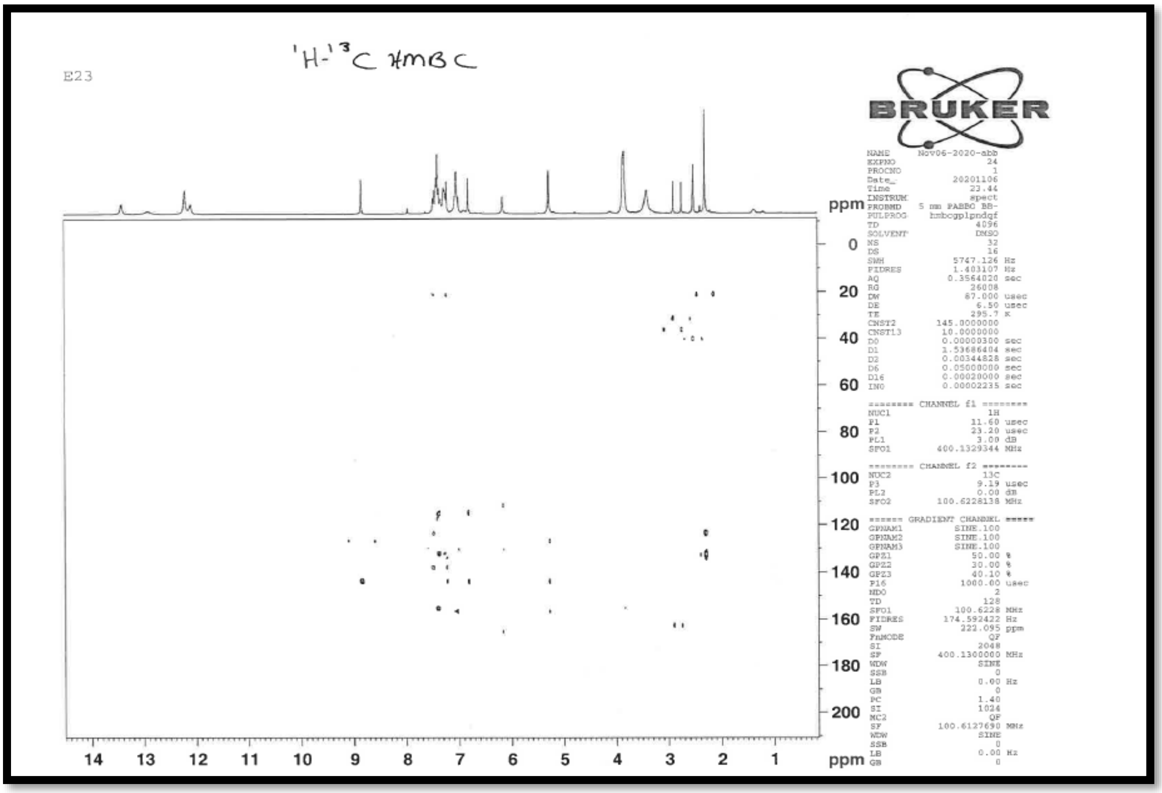
E23
1H-13C HMBC
ppm

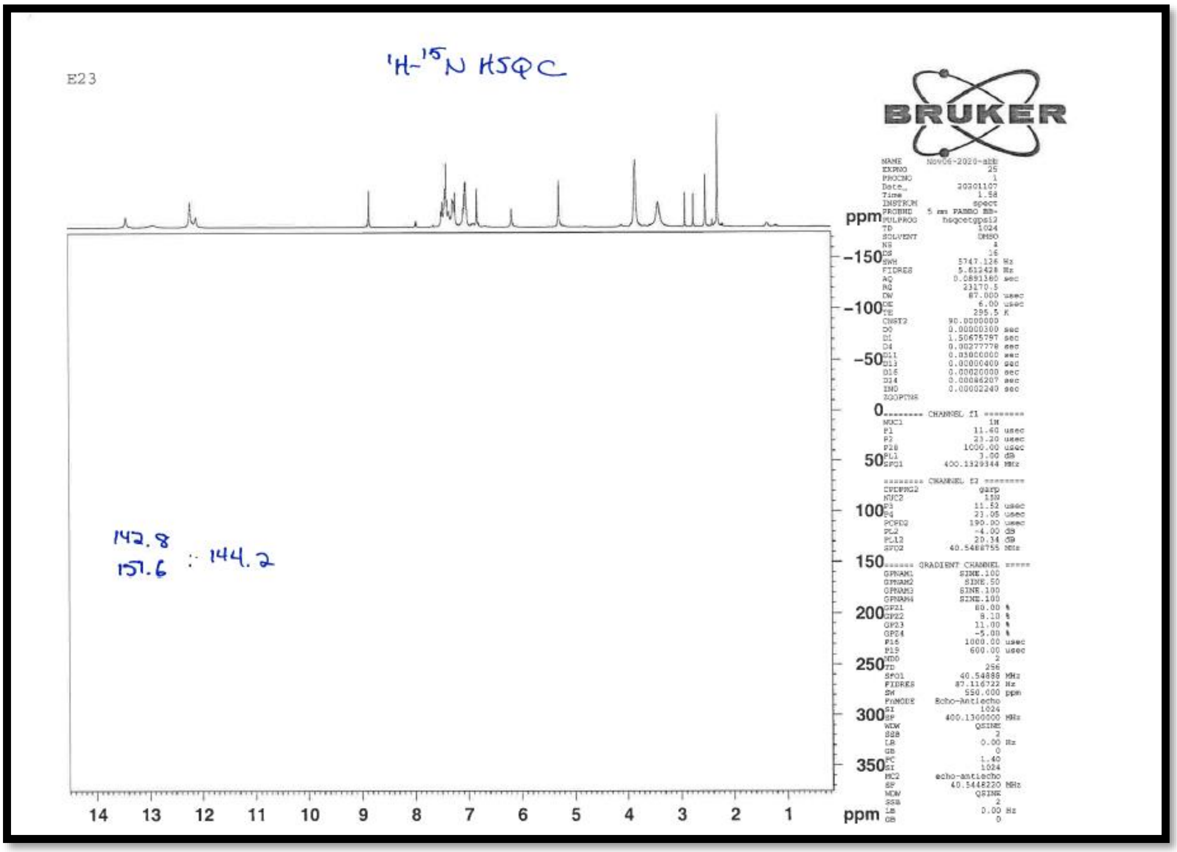
E23
1H-15N HSQC
142.8
151.6 : 144.2
ppm

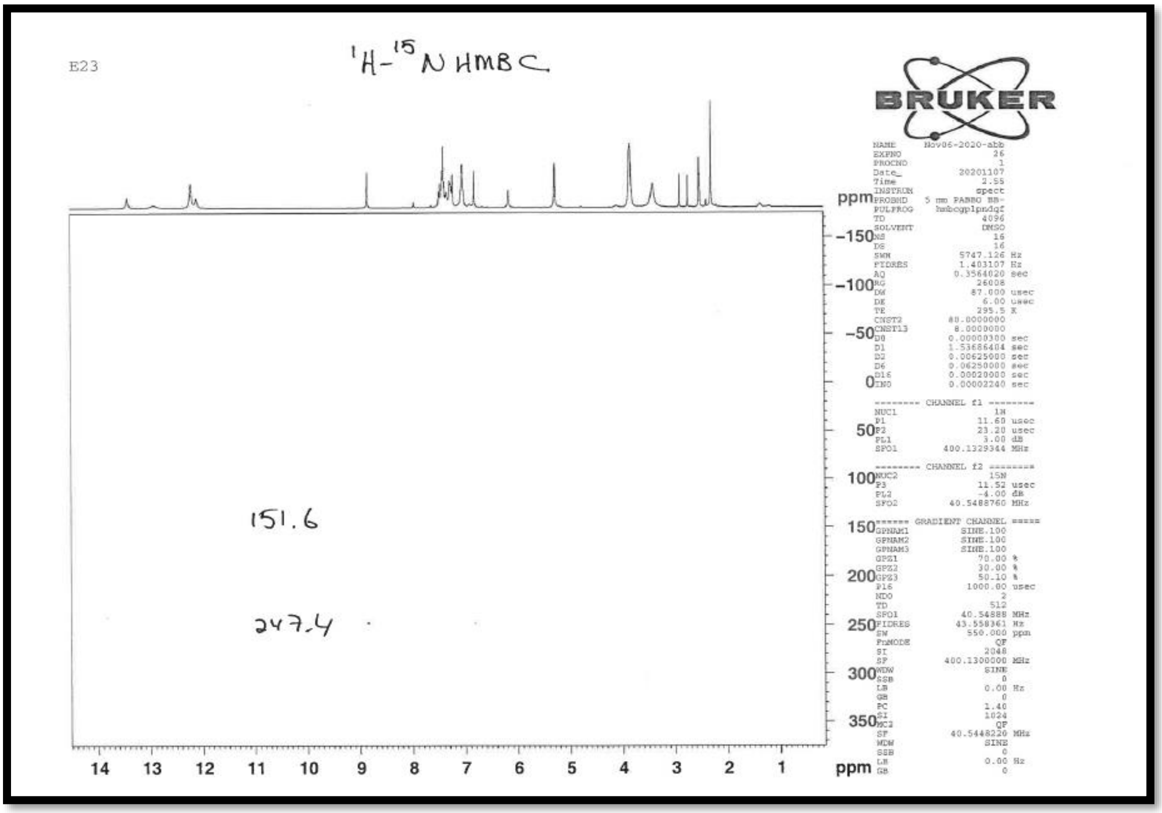

E23
1H-15N HMBC
BRUKER
151.6
247.4
ppm
-150
-100
-50
0
50
100
150
200
250
300
350
14 13 12 11 10 9 8 7 6 5 4 3 2 1 ppm
NAME Nov06-2020-abb
EXPNO 26
PROCNO 1
Date_ 20201107
Time 2.55
INSTRUM spect
PROBHD 5 mm PABBO BB-
PULPROG hmbcgplpndqf
TD 4096
SOLVENT DMSO
NS 16
DS 16
SWH 5747.126 Hz
FIDRES 1.403107 Hz
AQ 0.3564020 sec
RG 26008
DW 87.000 usec
DE 6.00 usec
TE 295.5 K
CNST2 88.0000000
CNST13 8.0000000
D0 0.00000300 sec
D1 1.53686404 sec
D2 0.00625000 sec
D6 0.06250000 sec
D16 0.00020000 sec
IN0 0.00002240 sec
======== CHANNEL f1 ========
NUC1 1H
P1 11.60 usec
P2 23.20 usec
PL1 3.00 dB
SFO1 400.1329344 MHz
======== CHANNEL f2 ========
NUC2 15N
P3 11.52 usec
PL2 -4.00 dB
SFO2 40.5488760 MHz
====== GRADIENT CHANNEL =====
GPNAM1 SINE.100
GPNAM2 SINE.100
GPNAM3 SINE.100
GPZ1 70.00 %
GPZ2 30.00 %
GPZ3 50.10 %
P16 1000.00 usec
ND0 2
TD 512
SFO1 40.54888 MHz
FIDRES 43.558361 Hz
SW 550.000 ppm
FnMODE QF
SI 2048
SF 400.1300000 MHz
WDW SINE
SSB 0
LB 0.00 Hz
GB 0
PC 1.40
SI 1024
MC2 QF
SF 40.5448220 MHz
WDW SINE
SSB 0
LB 0.00 Hz
GB 0

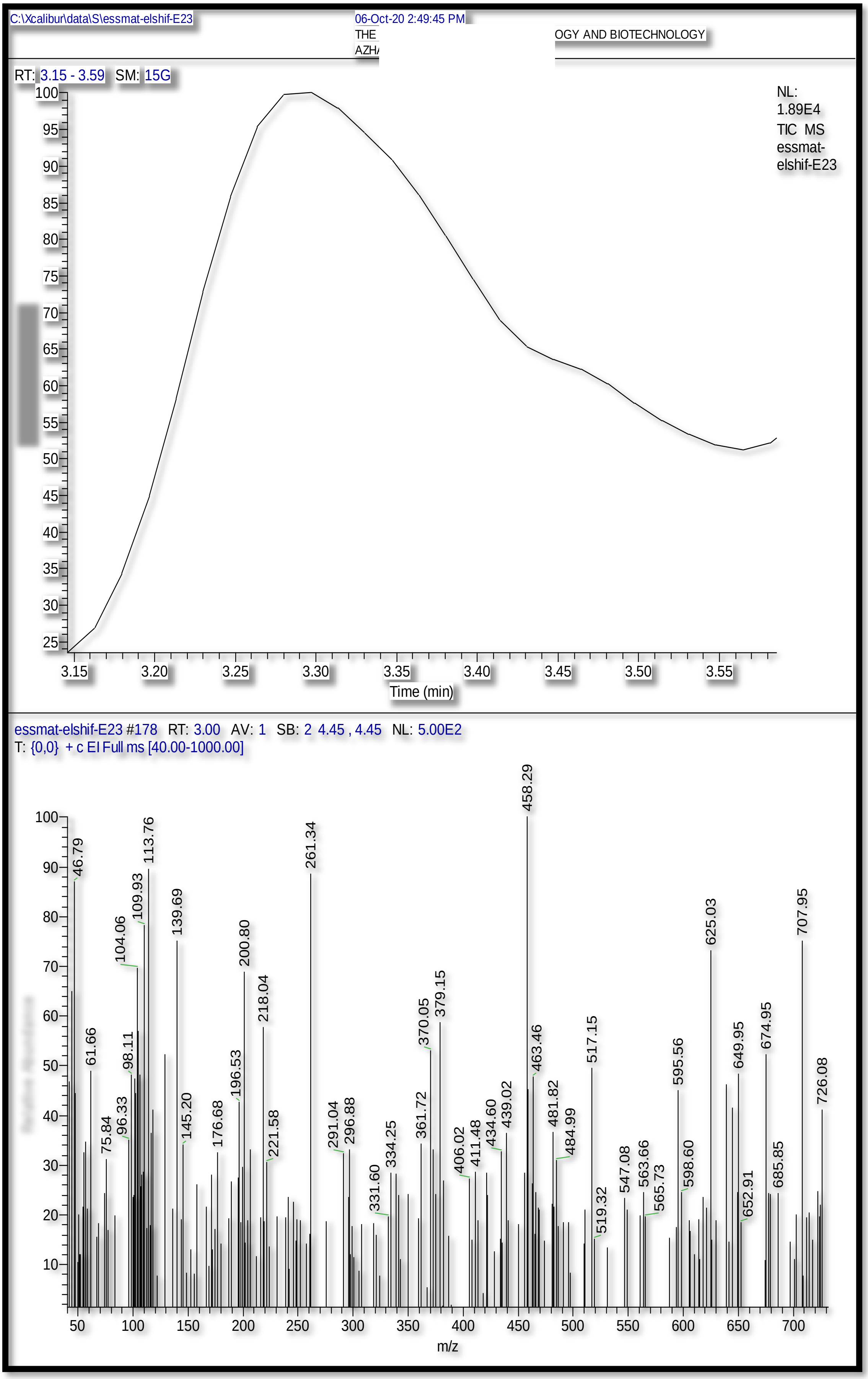

Spectrum data for Compound 8g

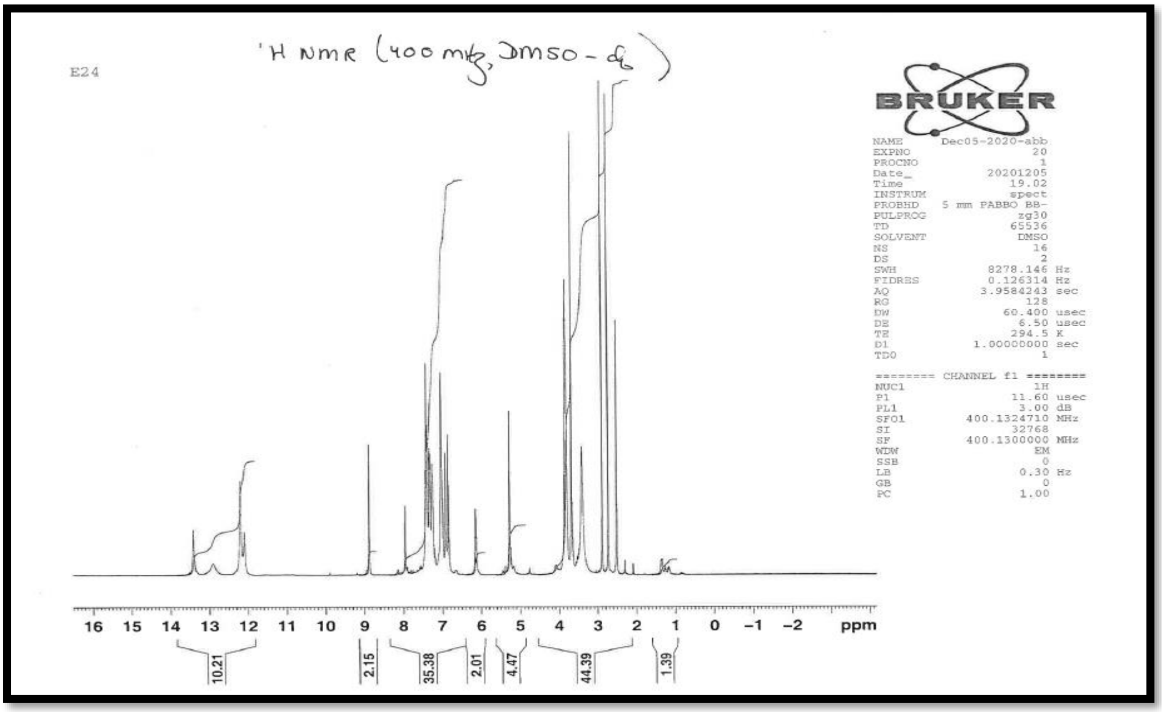

E24
'H NMR (400 mHz, DMSO - d6)
BRUKER
NAME Dec05-2020-abb
EXPNO 20
PROCNO 1
Date_ 20201205
Time 19.02
INSTRUM spect
PROBHD 5 mm PABBO BB-
PULPROG zg30
TD 65536
SOLVENT DMSO
NS 16
DS 2
SWH 8278.146 Hz
FIDRES 0.126314 Hz
AQ 3.9584243 sec
RG 128
DW 60.400 usec
DE 6.50 usec
TE 294.5 K
D1 1.00000000 sec
TD0 1
======== CHANNEL f1 ========
NUC1 1H
P1 11.60 usec
PL1 3.00 dB
SFO1 400.1324710 MHz
SI 32768
SF 400.1300000 MHz
WDW EM
SSB 0
LB 0.30 Hz
GB 0
PC 1.00
16 15 14 13 12 11 10 9 8 7 6 5 4 3 2 1 0 −1 −2 ppm
10.21
2.15
35.38
2.01
4.47
44.39
1.39

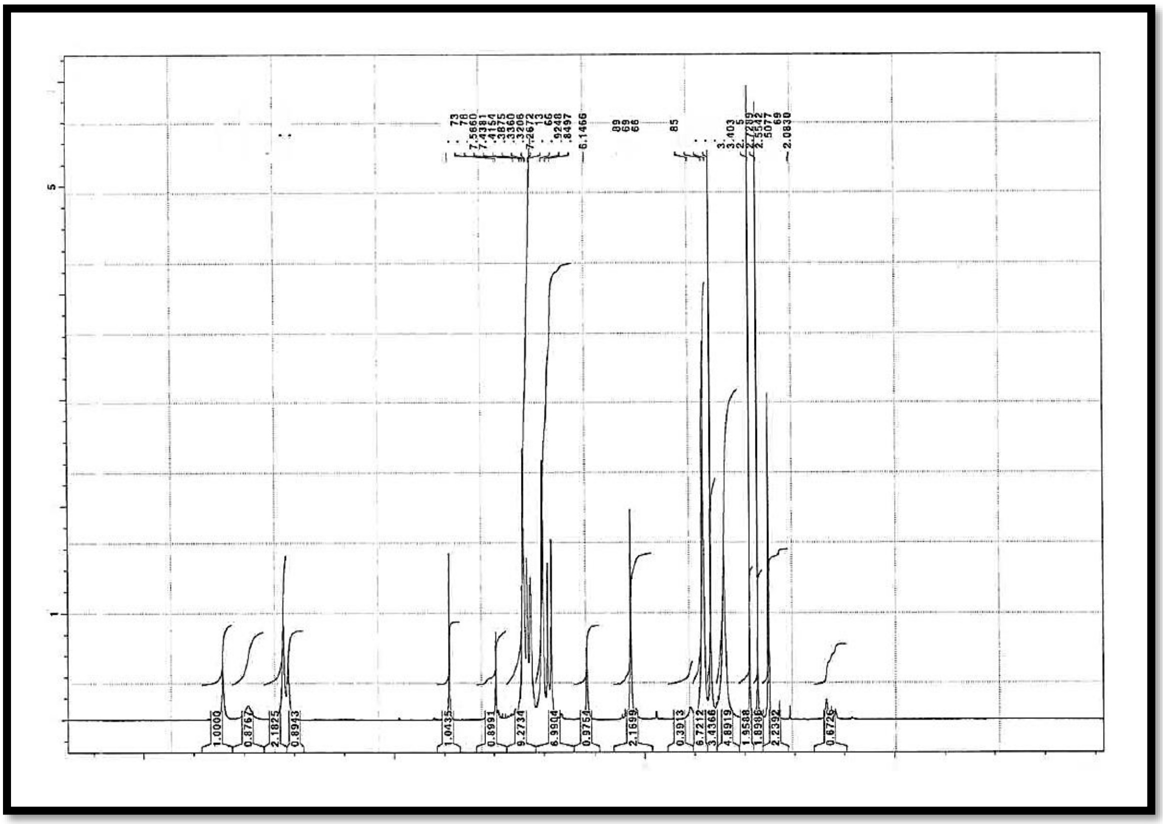

1.0000
0.8767
2.1825
0.8943
1.0435
0.8991
9.2734
6.9904
0.9754
2.1599
0.3913
6.7212
3.4366
4.8919
1.9588
1.8986
2.2392
0.6726

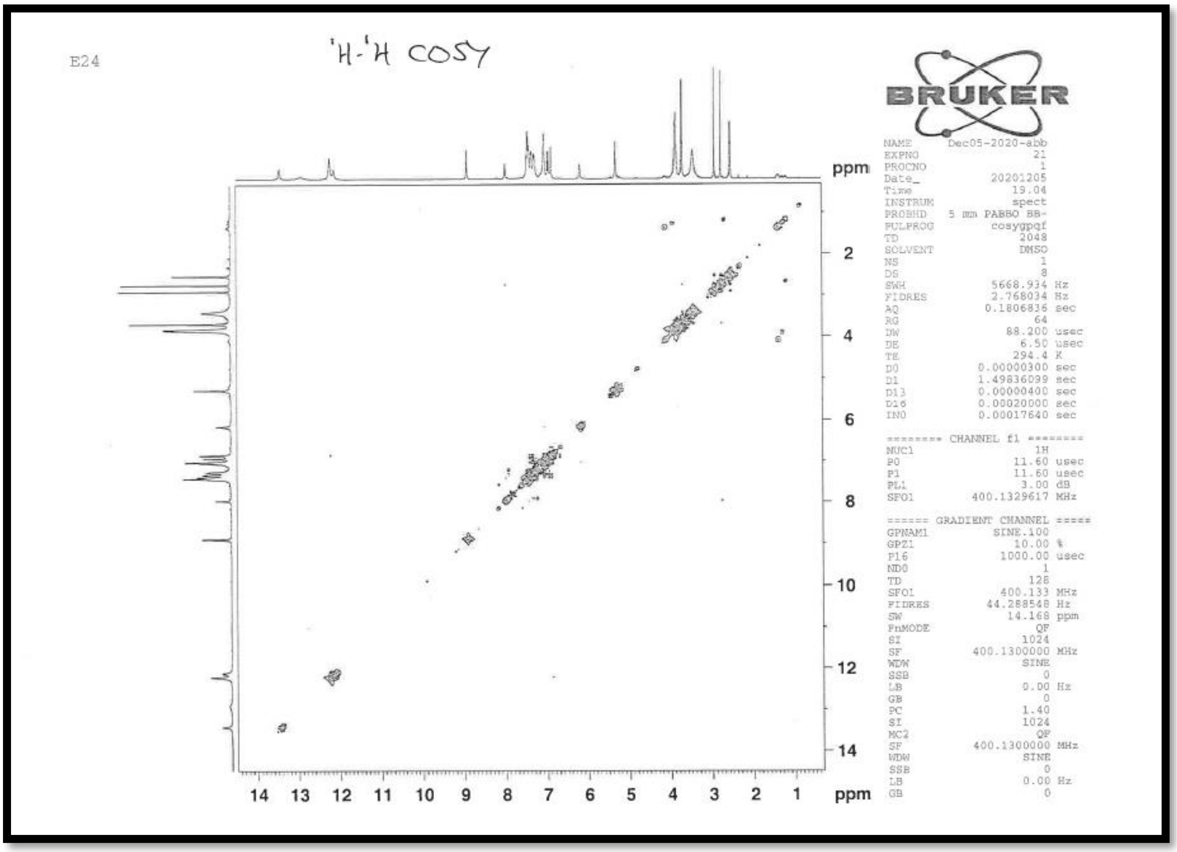
E24
¹H-¹H COSY
BRUKER
NAME Dec05-2020-abb
EXPNO 21
PROCNO 1
Date_ 20201205
Time 19.04
INSTRUM spect
PROBHD 5 mm PABBO BB-
PULPROG cosygpqf
TD 2048
SOLVENT DMSO
NS 1
DS 8
SWH 5668.934 Hz
FIDRES 2.768034 Hz
AQ 0.1806836 sec
RG 64
DW 88.200 usec
DE 6.50 usec
TE 294.4 K
D0 0.00000300 sec
D1 1.49836099 sec
D13 0.00000400 sec
D16 0.00020000 sec
IN0 0.00017640 sec
======== CHANNEL f1 ========
NUC1 1H
P0 11.60 usec
P1 11.60 usec
PL1 3.00 dB
SFO1 400.1329617 MHz
====== GRADIENT CHANNEL =====
GPNAM1 SINE.100
GPZ1 10.00 %
P16 1000.00 usec
ND0 1
TD 128
SFO1 400.133 MHz
FIDRES 44.288548 Hz
SW 14.168 ppm
FnMODE QF
SI 1024
SF 400.1300000 MHz
WDW SINE
SSB 0
LB 0.00 Hz
GB 0
PC 1.40
SI 1024
MC2 QF
SF 400.1300000 MHz
WDW SINE
SSB 0
LB 0.00 Hz
GB 0
ppm
14 13 12 11 10 9 8 7 6 5 4 3 2 1 ppm

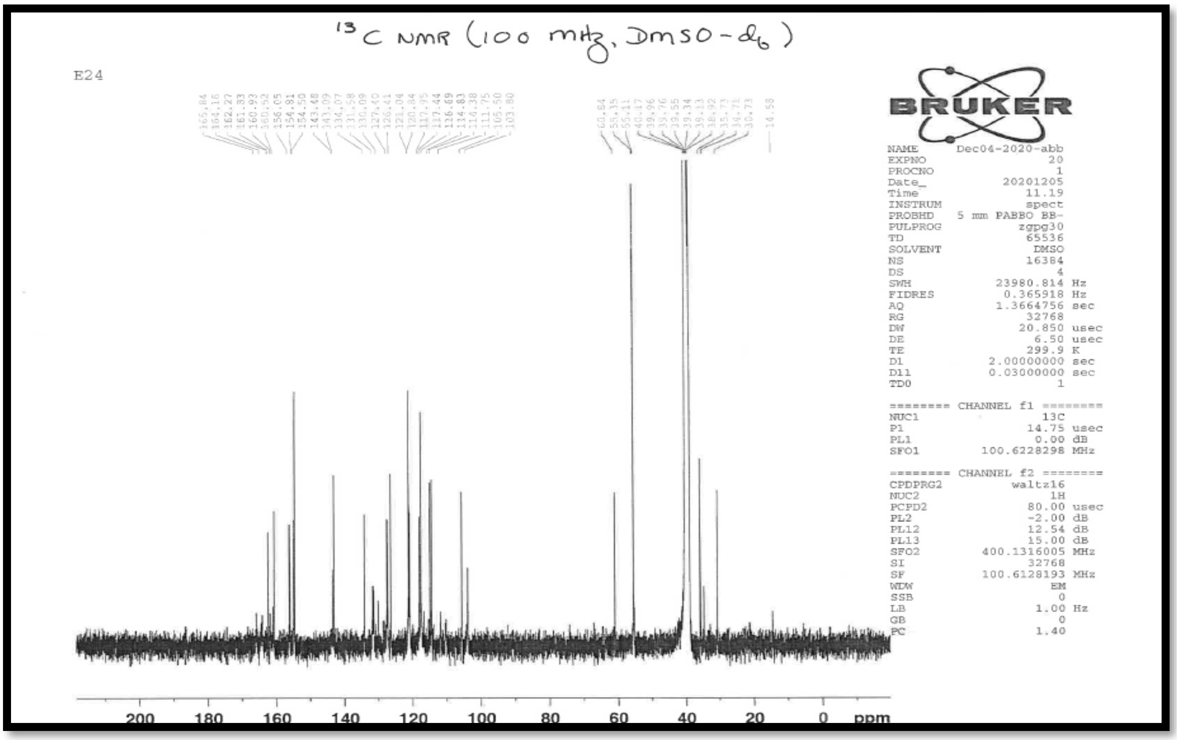
¹³C NMR (100 MHz, DMSO-d₆)
E24
BRUKER
NAME Dec04-2020-abb
EXPNO 20
PROCNO 1
Date_ 20201205
Time 11.19
INSTRUM spect
PROBHD 5 mm PABBO BB-
PULPROG zgpg30
TD 65536
SOLVENT DMSO
NS 16384
DS 4
SWH 23980.814 Hz
FIDRES 0.365918 Hz
AQ 1.3664756 sec
RG 32768
DW 20.850 usec
DE 6.50 usec
TE 299.9 K
D1 2.00000000 sec
D11 0.03000000 sec
TD0 1
======== CHANNEL f1 ========
NUC1 13C
P1 14.75 usec
PL1 0.00 dB
SFO1 100.6228298 MHz
======== CHANNEL f2 ========
CPDPRG2 waltz16
NUC2 1H
PCPD2 80.00 usec
PL2 -2.00 dB
PL12 12.54 dB
PL13 15.00 dB
SFO2 400.1316005 MHz
SI 32768
SF 100.6128193 MHz
WDW EM
SSB 0
LB 1.00 Hz
GB 0
PC 1.40
200 180 160 140 120 100 80 60 40 20 0 ppm

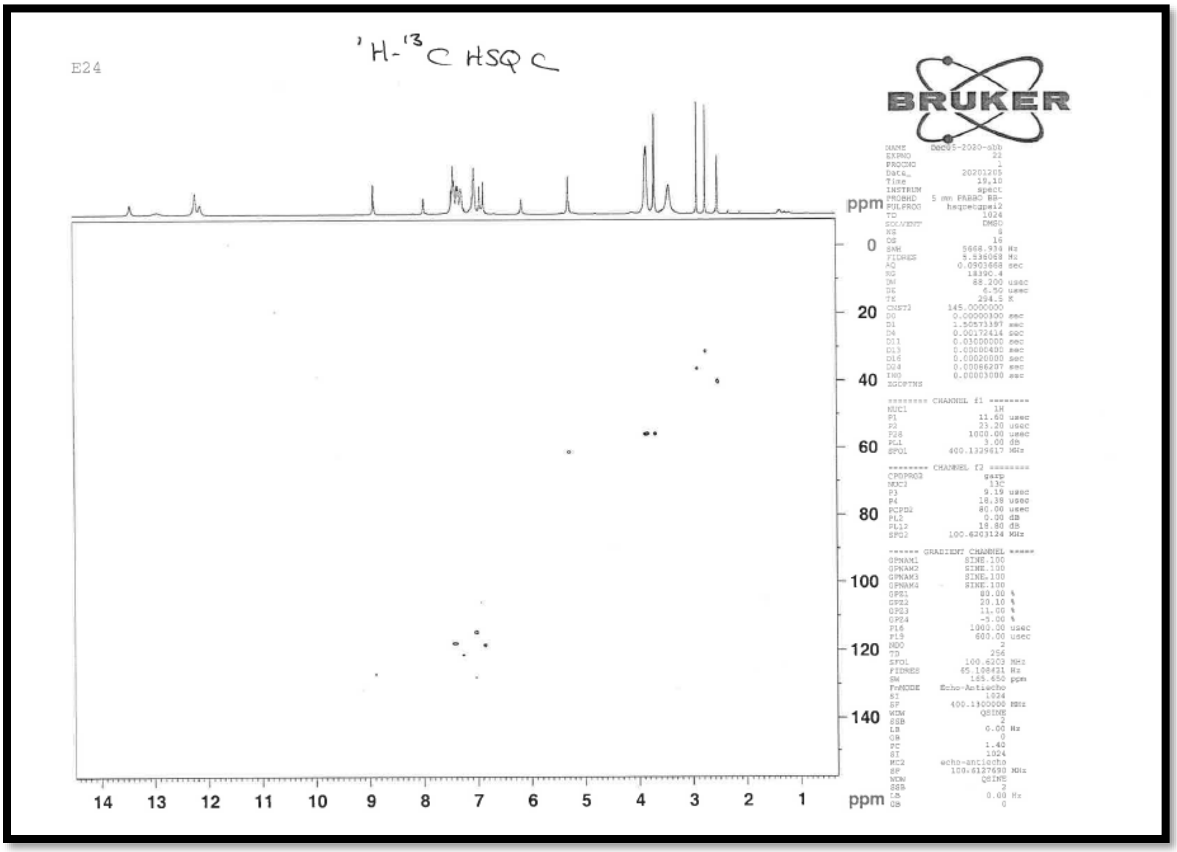
E24
1H-13C HSQC
ppm

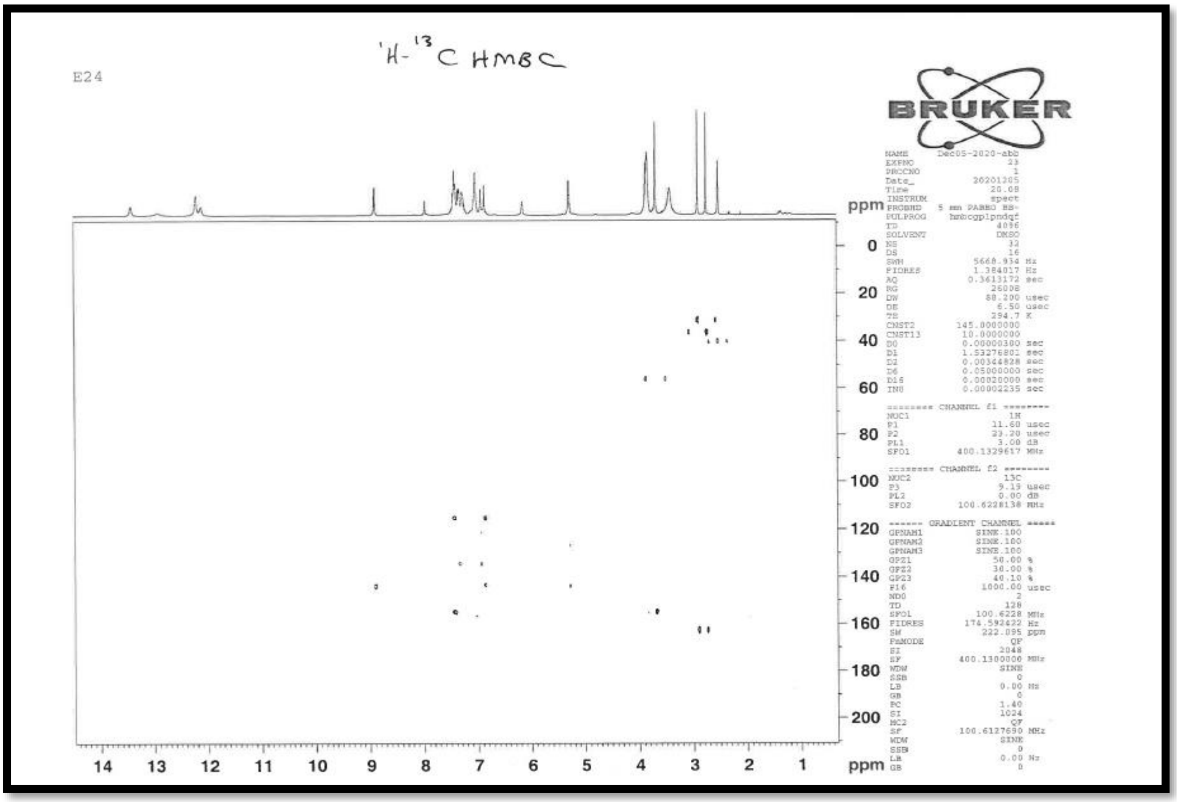
E24
1H-13C HMBC
ppm

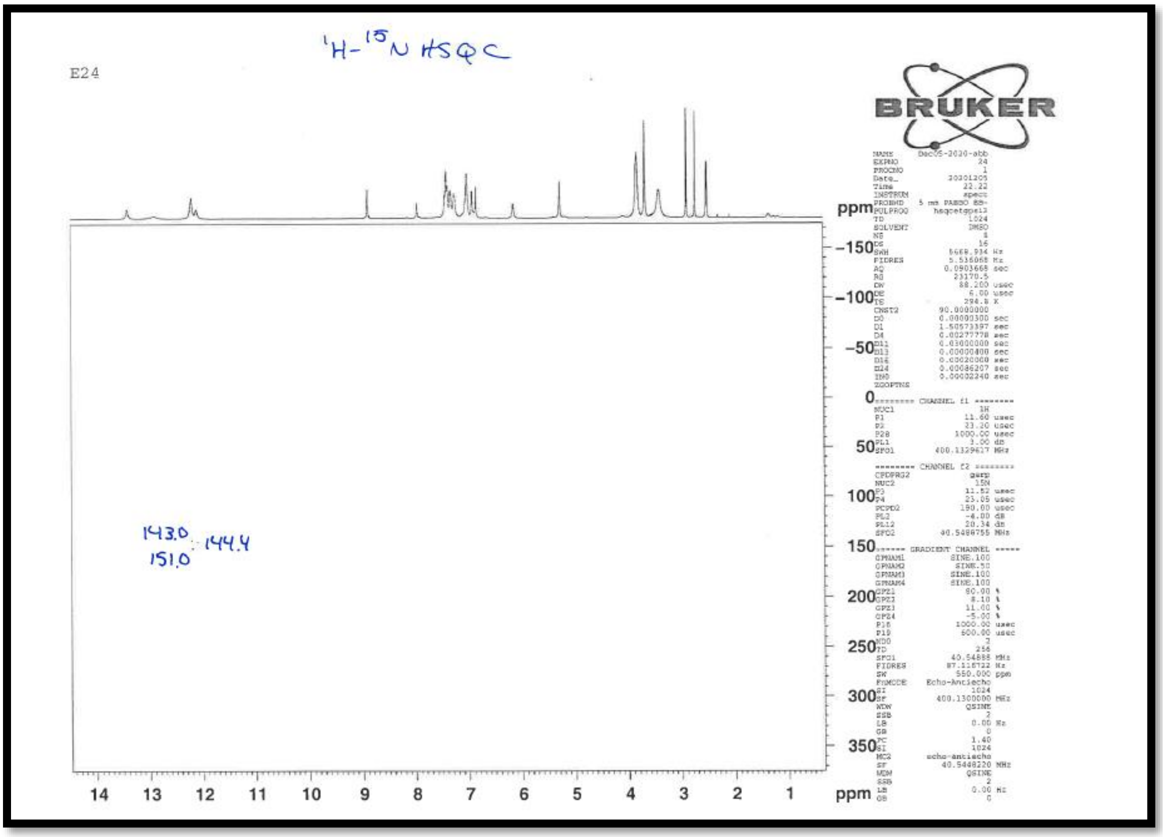
E24
1H-15N HSQC
BRUKER
143.0
144.4
151.0
ppm

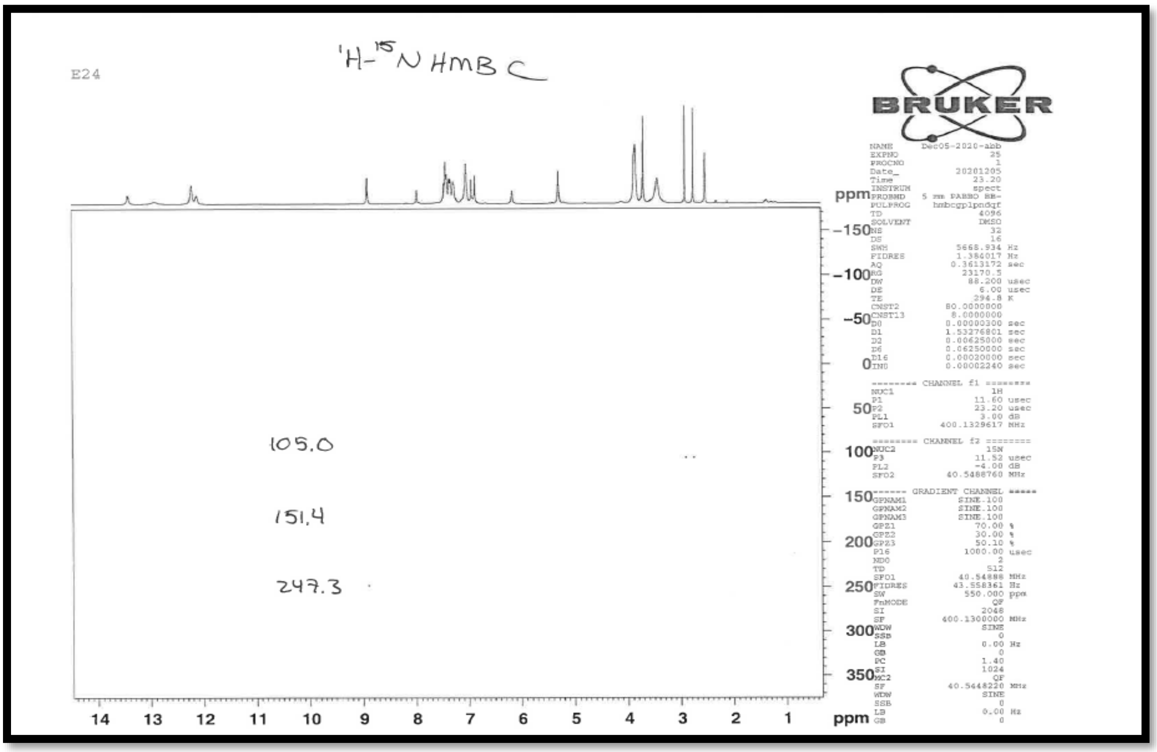
E24
1H-15N HMBC
BRUKER
105.0
151.4
247.3
ppm

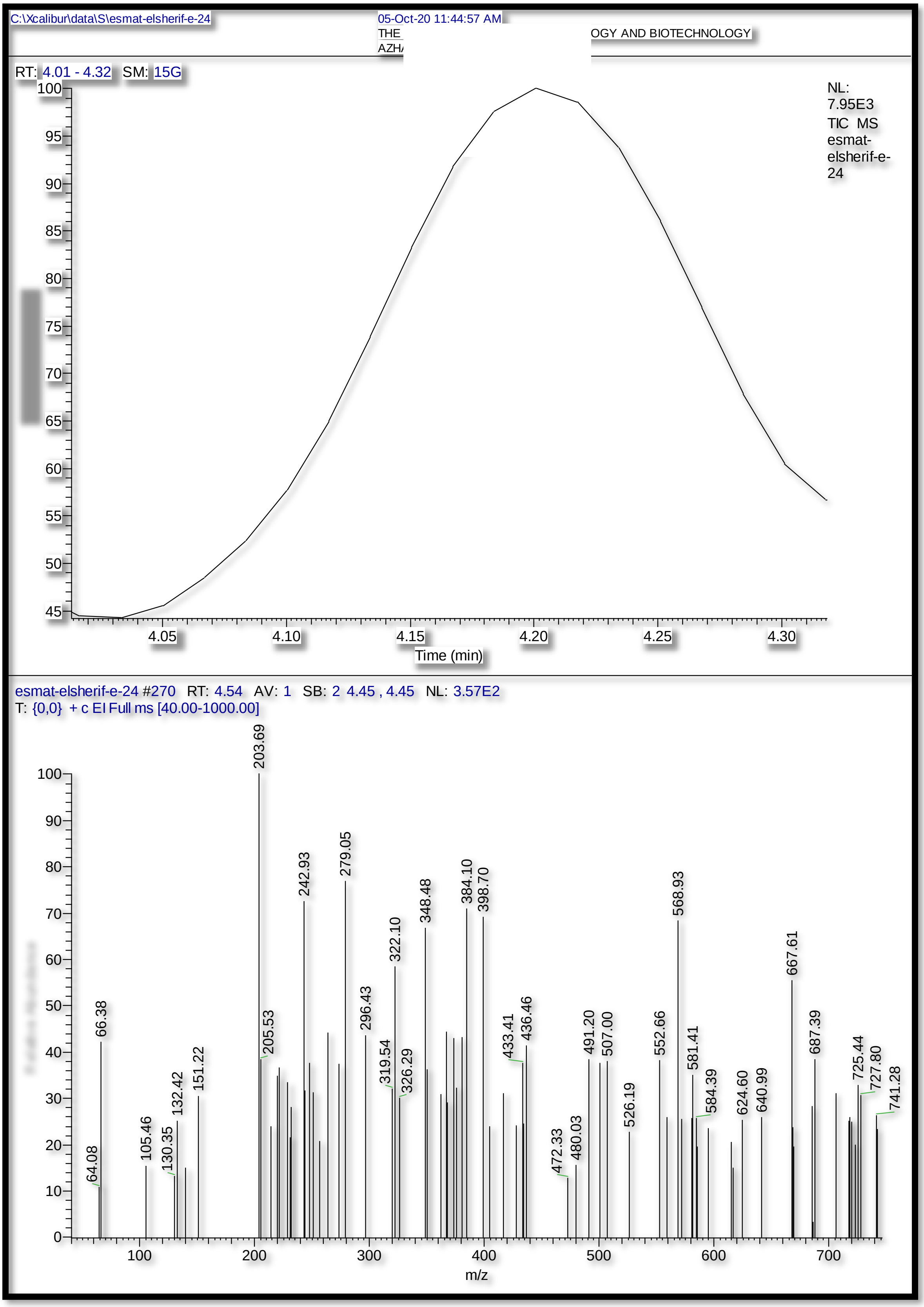

Spectrum data for Compound 8h

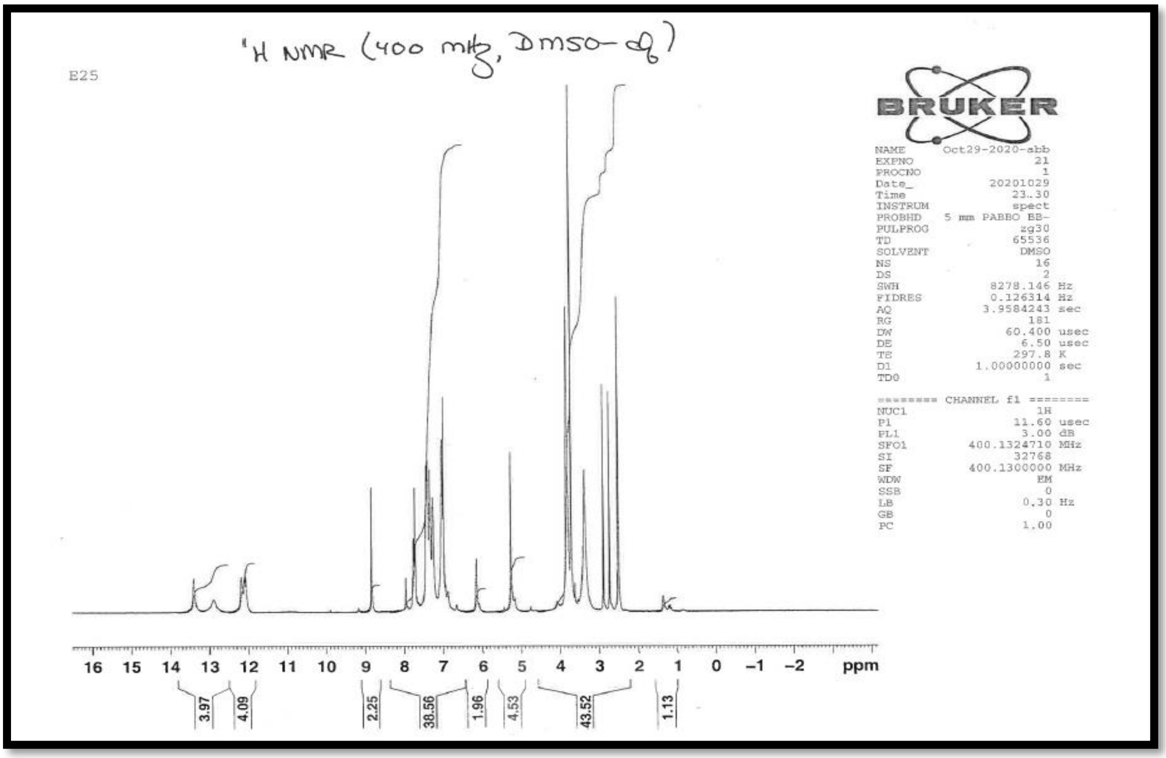

'H NMR (400 mHz, DMSO-d6)
E25
BRUKER
NAME Oct29-2020-abb
EXPNO 21
PROCNO 1
Date_ 20201029
Time 23.30
INSTRUM spect
PROBHD 5 mm PABBO BB-
PULPROG zg30
TD 65536
SOLVENT DMSO
NS 16
DS 2
SWH 8278.146 Hz
FIDRES 0.126314 Hz
AQ 3.9584243 sec
RG 181
DW 60.400 usec
DE 6.50 usec
TE 297.8 K
D1 1.00000000 sec
TD0 1
======== CHANNEL f1 ========
NUC1 1H
P1 11.60 usec
PL1 3.00 dB
SFO1 400.1324710 MHz
SI 32768
SF 400.1300000 MHz
WDW EM
SSB 0
LB 0.30 Hz
GB 0
PC 1.00
16 15 14 13 12 11 10 9 8 7 6 5 4 3 2 1 0 −1 −2 ppm
3.97
4.09
2.25
38.56
1.96
4.53
43.52
1.13

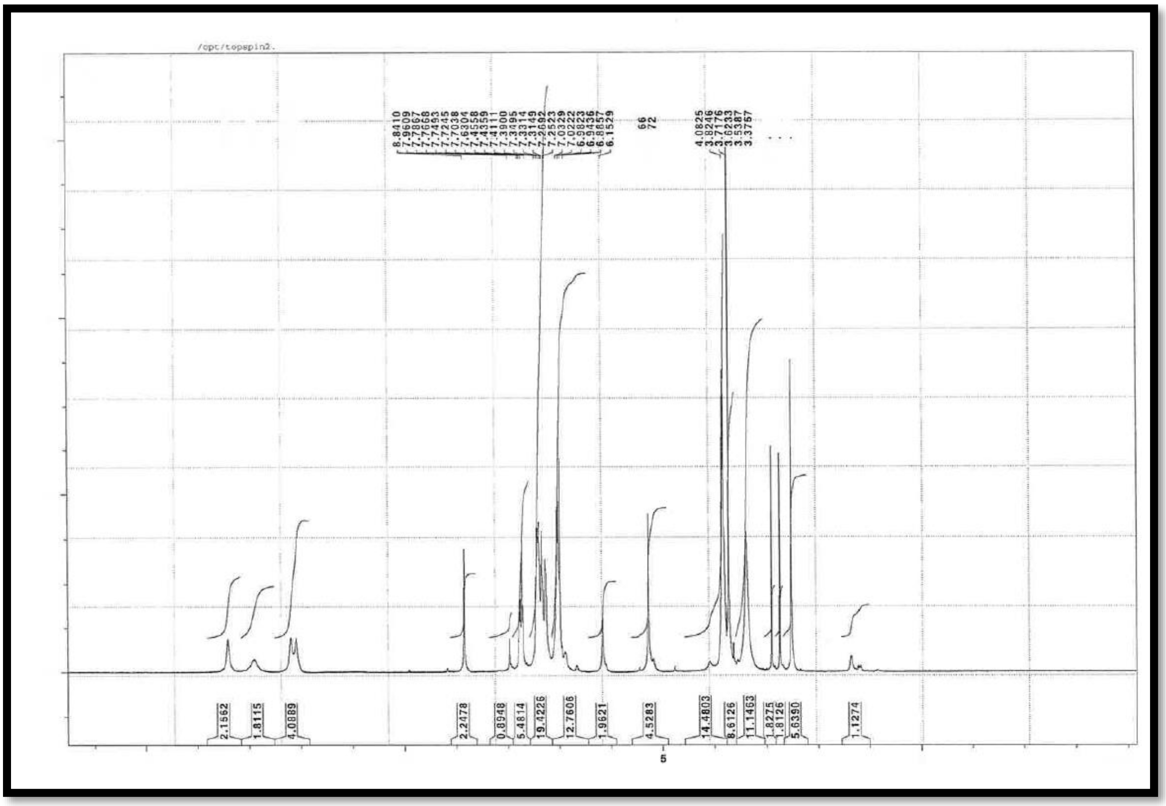

/opt/topspin2.
2.1562
1.8115
4.0889
2.2478
0.8948
5.4814
19.4226
12.7606
1.9621
4.5283
14.4803
8.6126
11.1463
1.8275
1.8126
5.6390
1.1274
5

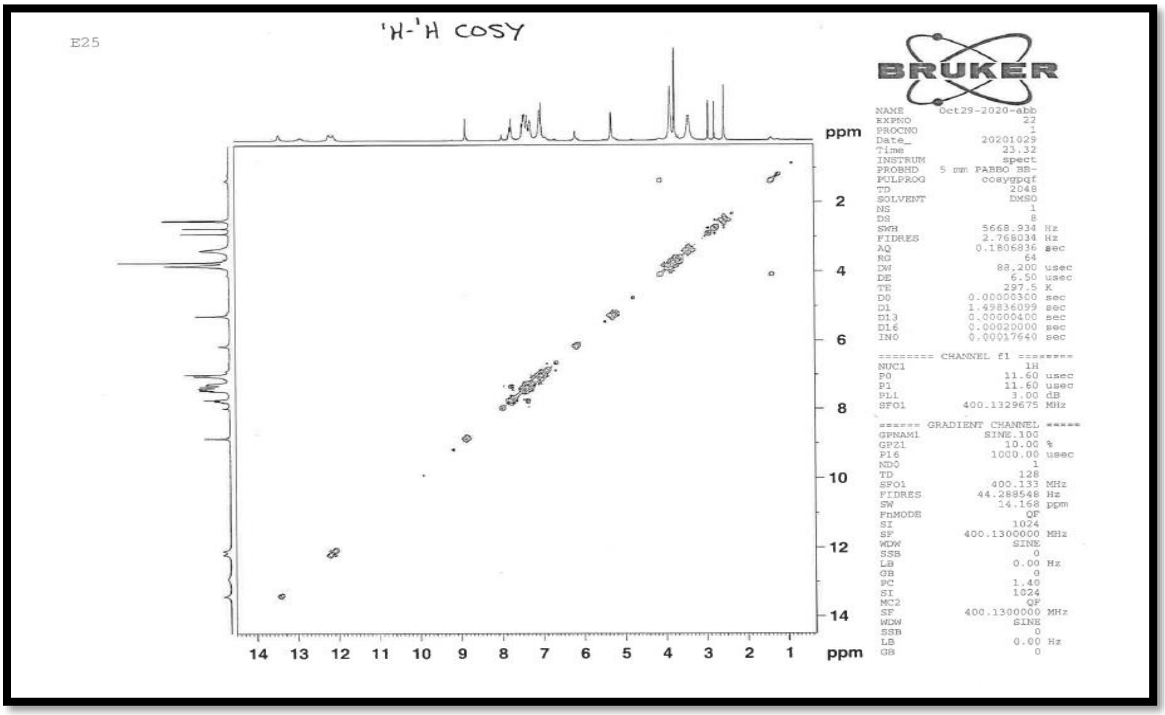
E25
1H-1H COSY
BRUKER
NAME Oct29-2020-abb
EXPNO 22
PROCNO 1
Date_ 20201029
Time 23.32
INSTRUM spect
PROBHD 5 mm PABBO BB-
PULPROG cosygpqf
TD 2048
SOLVENT DMSO
NS 1
DS 8
SWH 5668.934 Hz
FIDRES 2.768034 Hz
AQ 0.1806836 sec
RG 64
DW 88.200 usec
DE 6.50 usec
TE 297.5 K
D0 0.00000300 sec
D1 1.49836099 sec
D13 0.00000400 sec
D16 0.00020000 sec
IN0 0.00017640 sec
======== CHANNEL f1 ========
NUC1 1H
P0 11.60 usec
P1 11.60 usec
PL1 3.00 dB
SFO1 400.1329675 MHz
====== GRADIENT CHANNEL =====
GPNAM1 SINE.100
GPZ1 10.00 %
P16 1000.00 usec
ND0 1
TD 128
SFO1 400.133 MHz
FIDRES 44.288548 Hz
SW 14.168 ppm
FnMODE QF
SI 1024
SF 400.1300000 MHz
WDW SINE
SSB 0
LB 0.00 Hz
GB 0
PC 1.40
SI 1024
MC2 QF
SF 400.1300000 MHz
WDW SINE
SSB 0
LB 0.00 Hz
GB 0
ppm

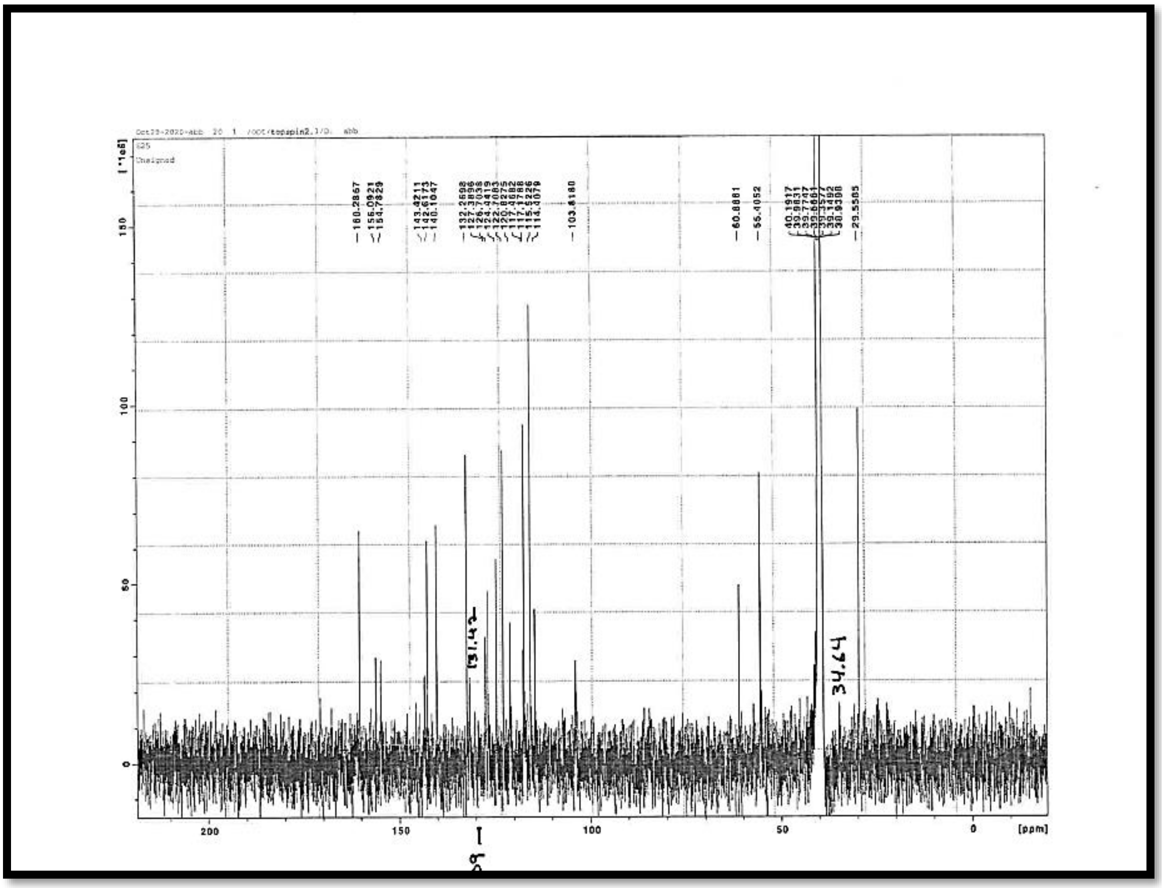
Oct29-2020-abb 20 1 /opt/topspin2.1/D. abb
E25
Unsigned
160.2867
156.0921
154.7829
143.4211
142.6173
140.1047
103.8180
60.8881
55.4052
40.1917
29.5505
131.42
34.64
[ppm]

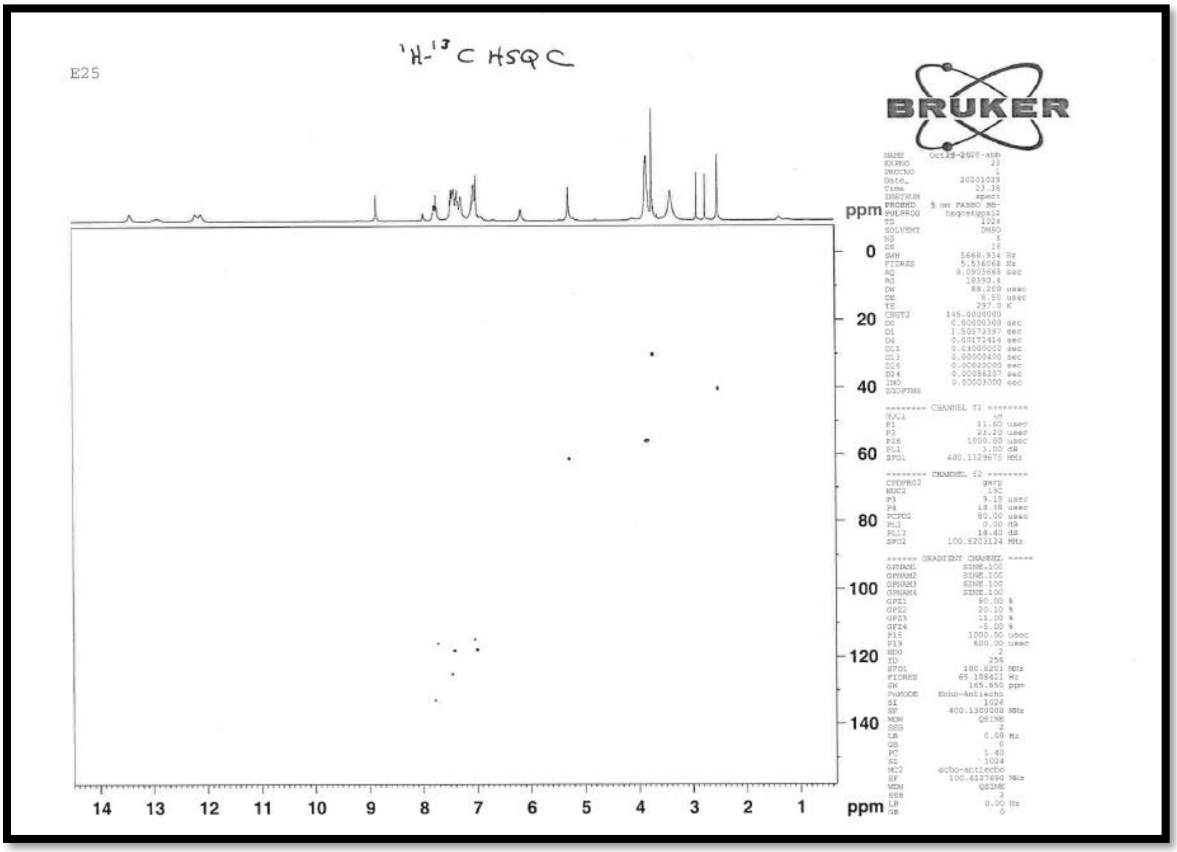
E25
1H-13C HSQC
BRUKER
ppm

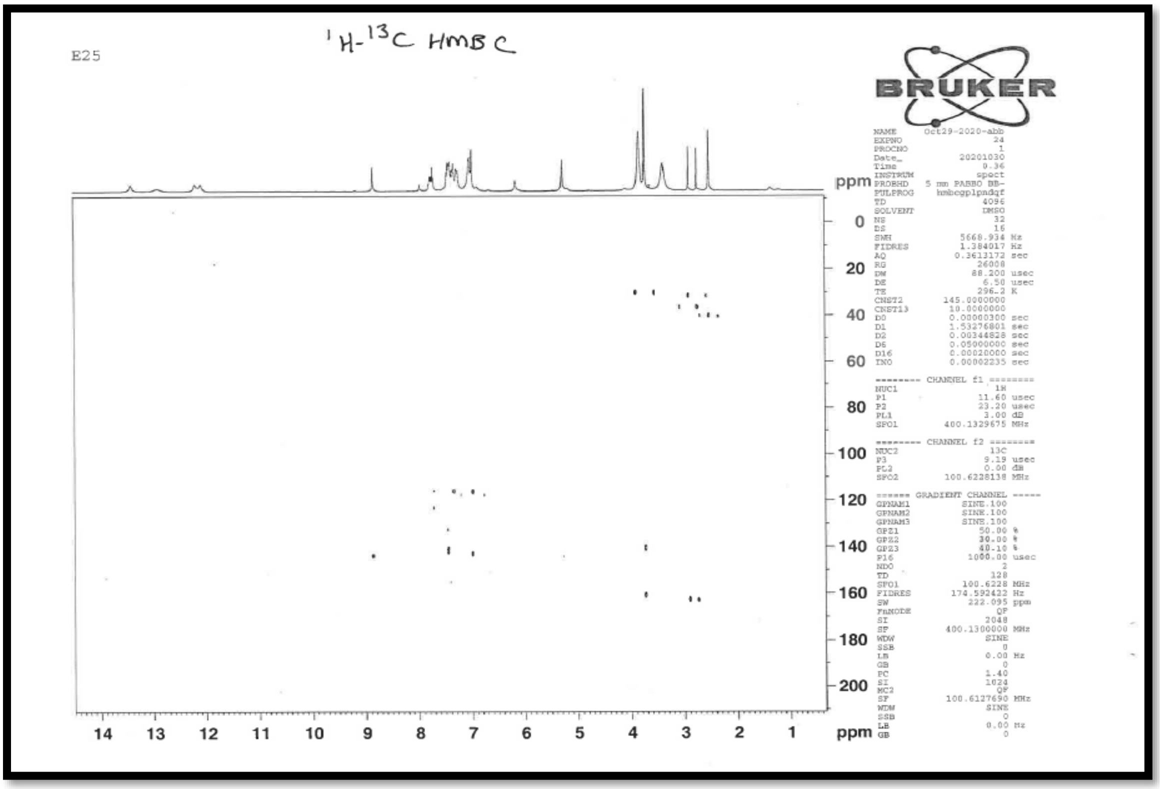
E25
1H-13C HMBC
BRUKER
ppm

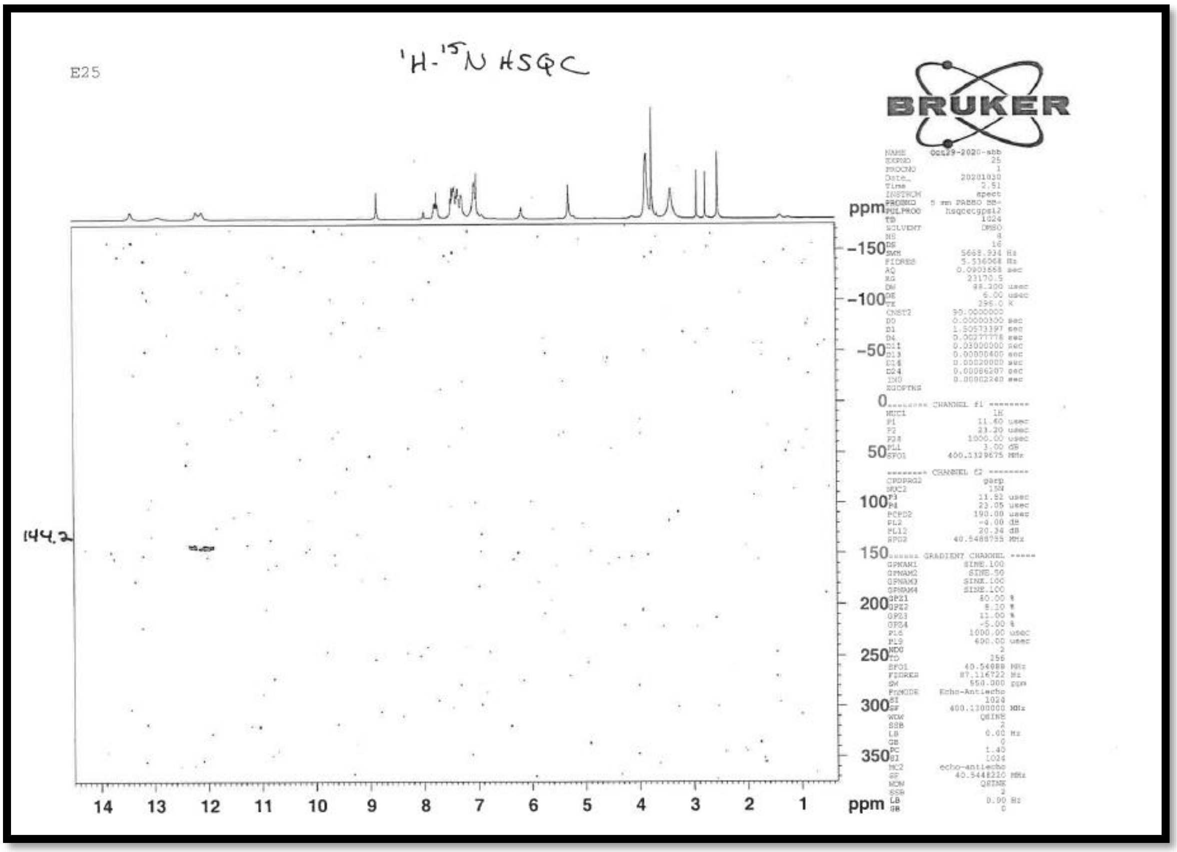
E25
'H-15N HSQC
144.2
ppm

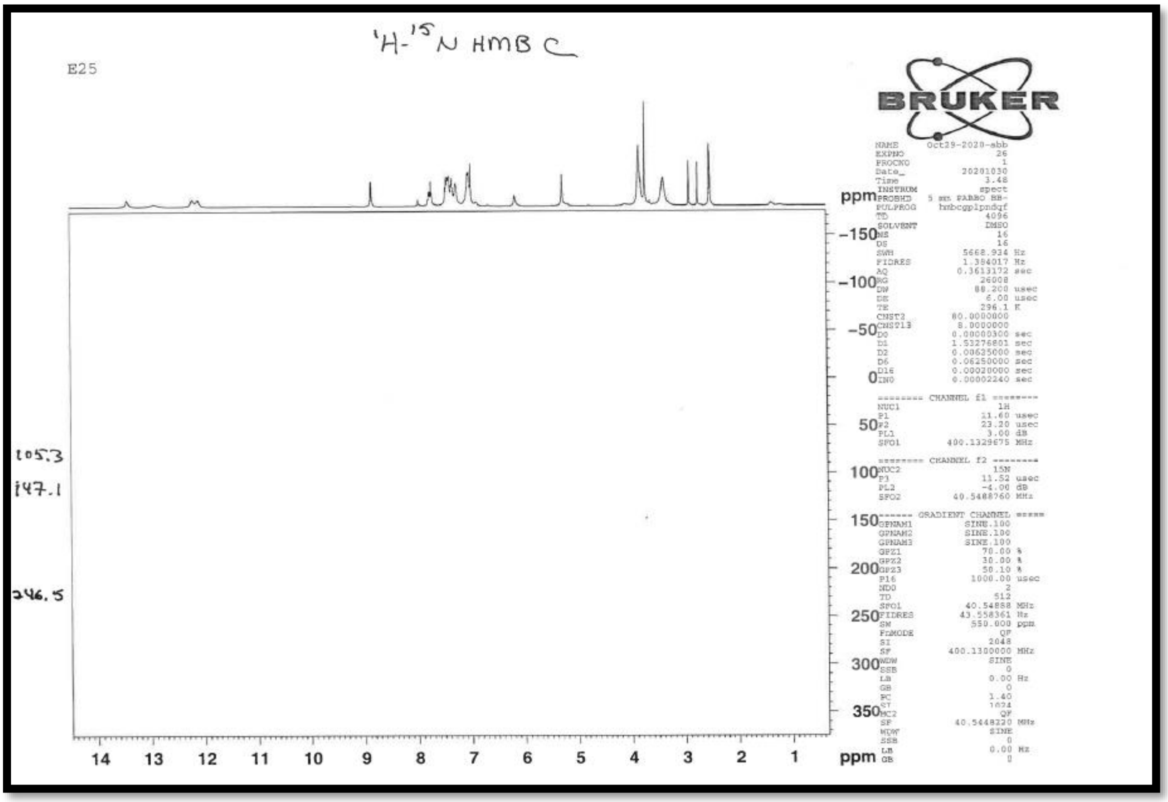
E25
'H-15N HMBC
105.3
147.1
246.5
ppm

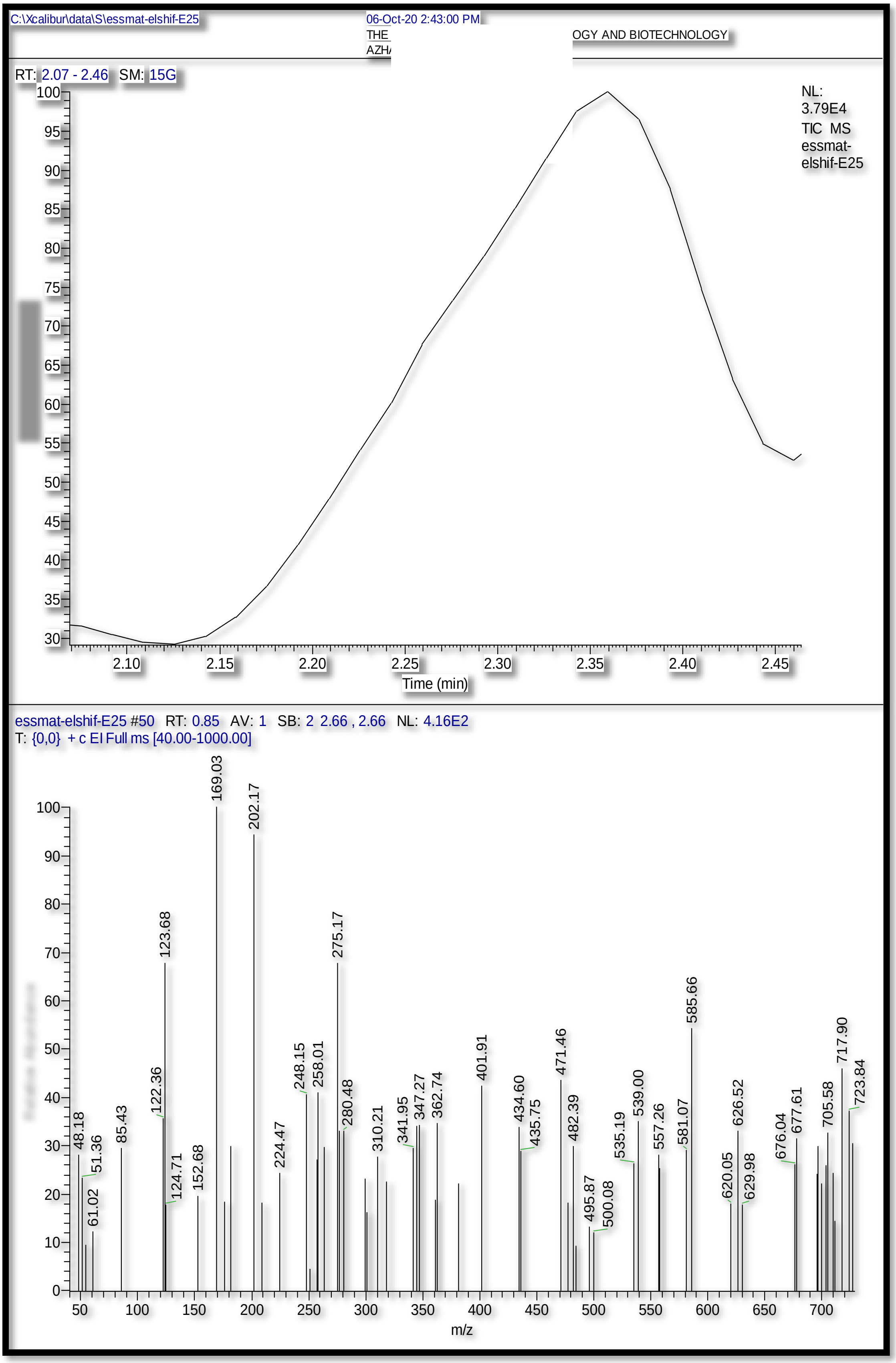

Spectrum data for Compound 8i

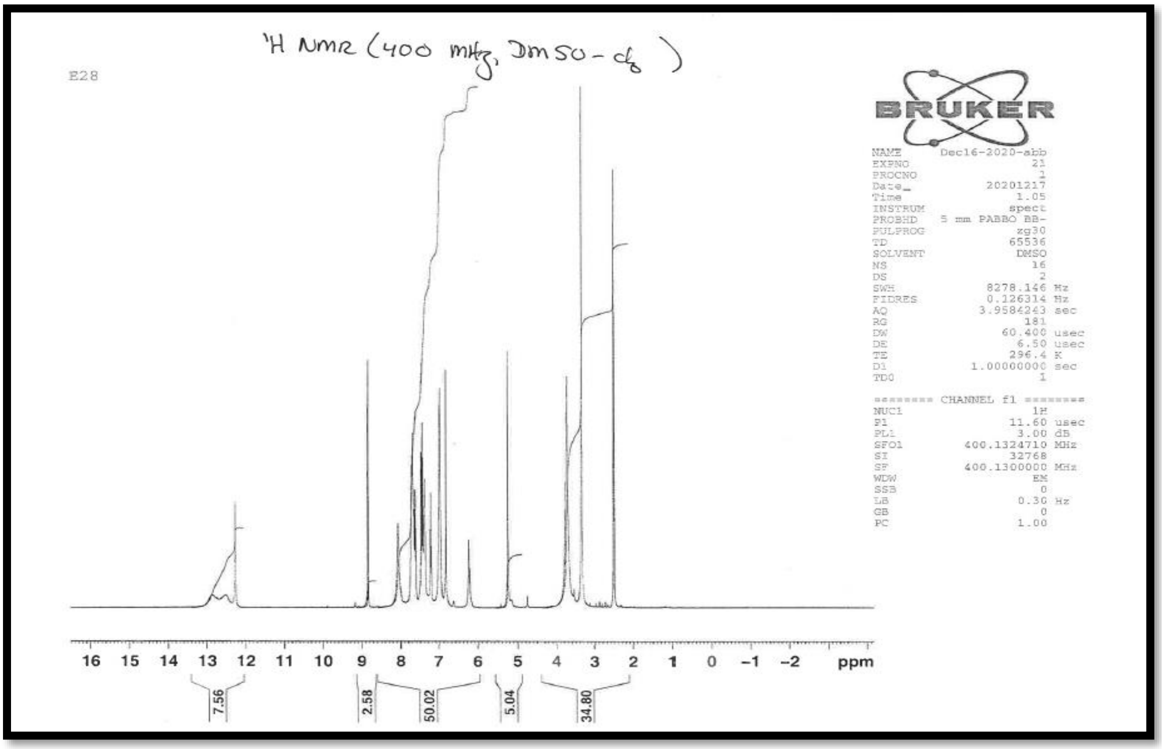
'H NMR (400 MHz, DMSO-d6)
E28
BRUKER
NAME Dec16-2020-abb
EXPNO 21
PROCNO 1
Date_ 20201217
Time 1.05
INSTRUM spect
PROBHD 5 mm PABBO BB-
PULPROG zg30
TD 65536
SOLVENT DMSO
NS 16
DS 2
SWH 8278.146 Hz
FIDRES 0.126314 Hz
AQ 3.9584243 sec
RG 181
DW 60.400 usec
DE 6.50 usec
TE 296.4 K
D1 1.00000000 sec
TD0 1
======== CHANNEL f1 ========
NUC1 1H
P1 11.60 usec
PL1 3.00 dB
SFO1 400.1324710 MHz
SI 32768
SF 400.1300000 MHz
WDW EM
SSB 0
LB 0.30 Hz
GB 0
PC 1.00
16 15 14 13 12 11 10 9 8 7 6 5 4 3 2 1 0 -1 -2 ppm
7.56
2.58
50.02
5.04
34.80

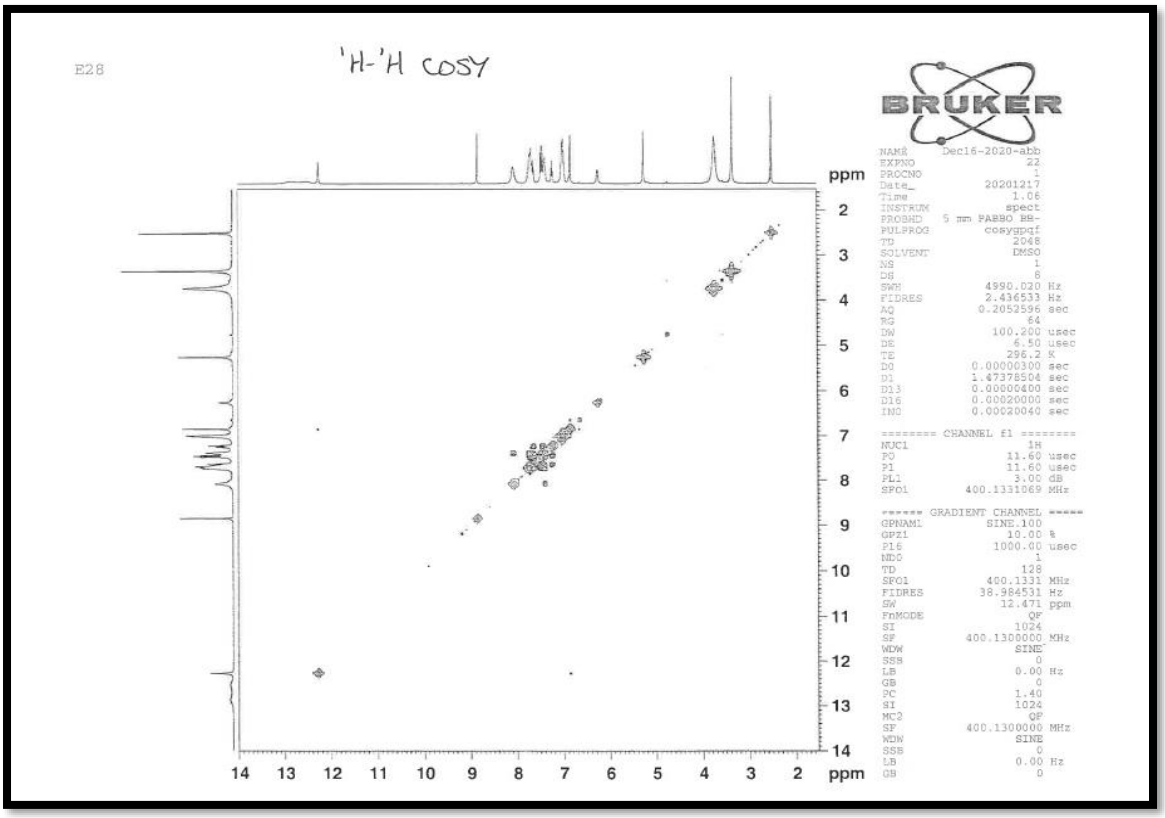
'H-'H COSY
E28
BRUKER
NAME Dec16-2020-abb
EXPNO 22
PROCNO 1
Date_ 20201217
Time 1.06
INSTRUM spect
PROBHD 5 mm PABBO BB-
PULPROG cosygpqf
TD 2048
SOLVENT DMSO
NS 1
DS 8
SWH 4990.020 Hz
FIDRES 2.436533 Hz
AQ 0.2052596 sec
RG 64
DW 100.200 usec
DE 6.50 usec
TE 296.2 K
D0 0.00000300 sec
D1 1.47378504 sec
D13 0.00000400 sec
D16 0.00020000 sec
IN0 0.00020040 sec
======== CHANNEL f1 ========
NUC1 1H
P0 11.60 usec
P1 11.60 usec
PL1 3.00 dB
SFO1 400.1331069 MHz
====== GRADIENT CHANNEL =====
GPNAM1 SINE.100
GPZ1 10.00 %
P16 1000.00 usec
ND0 1
TD 128
SFO1 400.1331 MHz
FIDRES 38.984531 Hz
SW 12.471 ppm
FnMODE QF
SI 1024
SF 400.1300000 MHz
WDW SINE
SSB 0
LB 0.00 Hz
GB 0
PC 1.40
SI 1024
MC2 QF
SF 400.1300000 MHz
WDW SINE
SSB 0
LB 0.00 Hz
GB 0
ppm
2 3 4 5 6 7 8 9 10 11 12 13 14
14 13 12 11 10 9 8 7 6 5 4 3 2 ppm

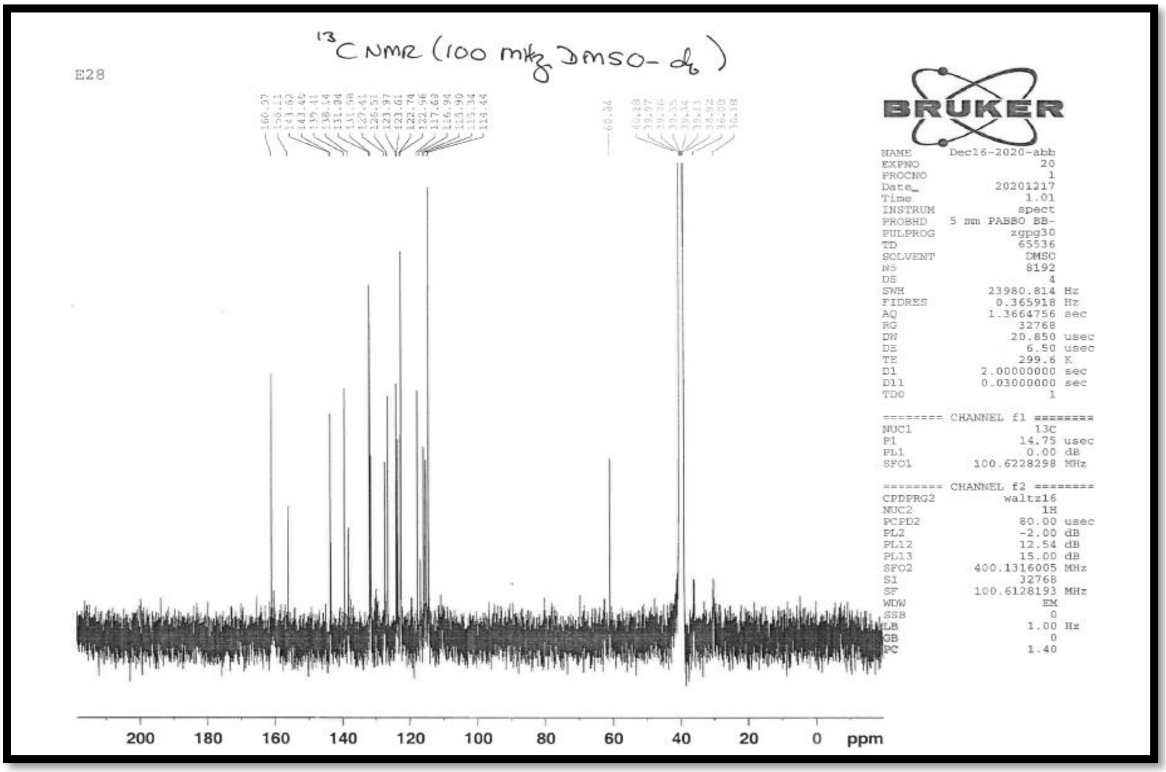

E28
^{13}C NMR (100 MHz DMSO-d_6)
BRUKER
NAME Dec16-2020-abb
EXPNO 20
PROCNO 1
Date_ 20201217
Time 1.01
INSTRUM spect
PROBHD 5 mm PABBO BB-
PULPROG zgpg30
TD 65536
SOLVENT DMSO
NS 8192
DS 4
SWH 23980.814 Hz
FIDRES 0.365918 Hz
AQ 1.3664756 sec
RG 32768
DW 20.850 usec
DE 6.50 usec
TE 299.6 K
D1 2.00000000 sec
D11 0.03000000 sec
TD0 1
======== CHANNEL f1 ========
NUC1 13C
P1 14.75 usec
PL1 0.00 dB
SFO1 100.6228298 MHz
======== CHANNEL f2 ========
CPDPRG2 waltz16
NUC2 1H
PCPD2 80.00 usec
PL2 -2.00 dB
PL12 12.54 dB
PL13 15.00 dB
SFO2 400.1316005 MHz
SI 32768
SF 100.6128193 MHz
WDW EM
SSB 0
LB 1.00 Hz
GB 0
PC 1.40
200 180 160 140 120 100 80 60 40 20 0 ppm

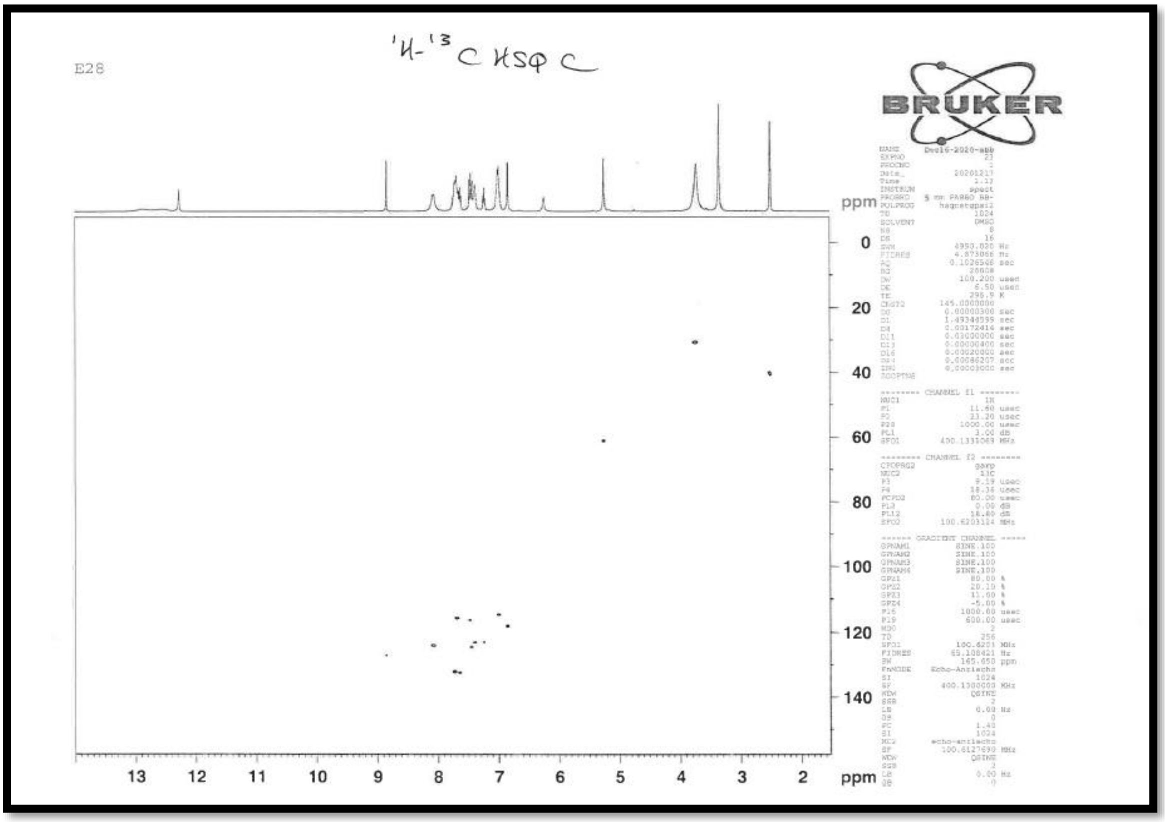

E28
^{1}H-^{13}C HSQC
BRUKER
NAME Dec16-2020-abb
ppm
0 20 40 60 80 100 120 140
13 12 11 10 9 8 7 6 5 4 3 2 ppm

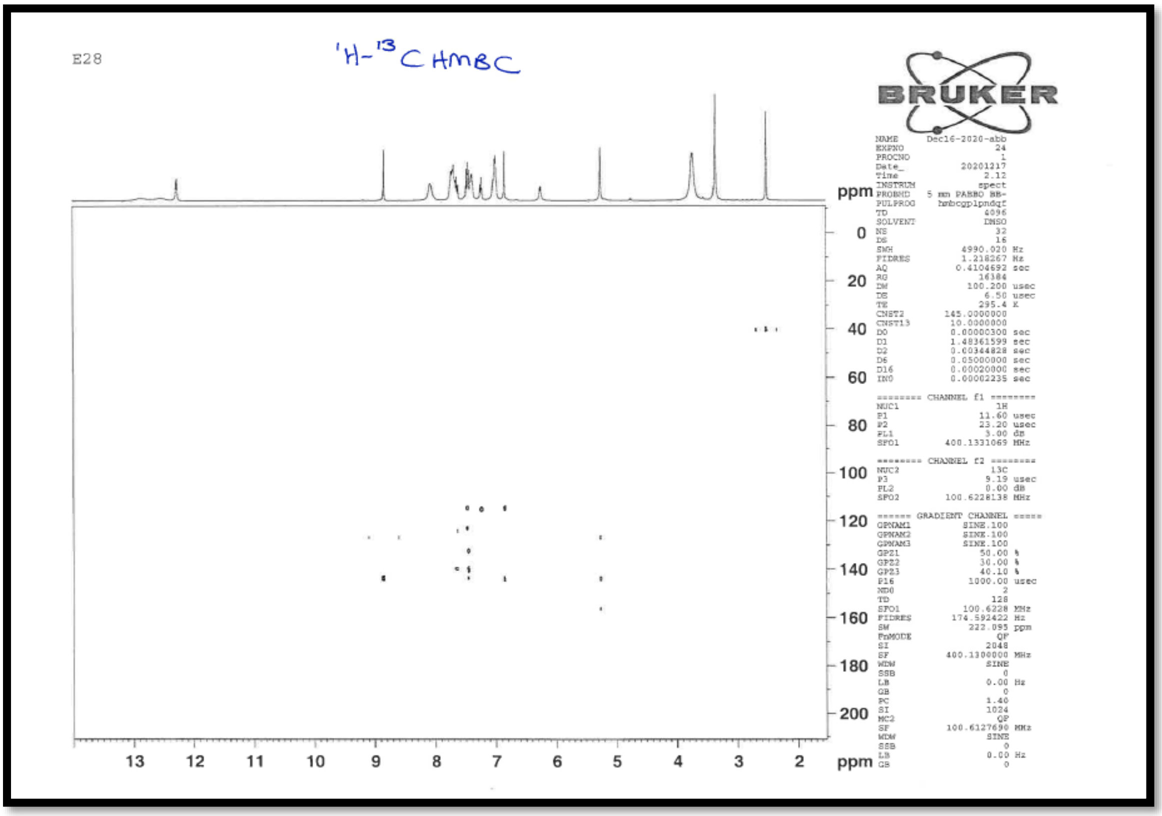
E28
1H-13C HMBC
BRUKER
ppm

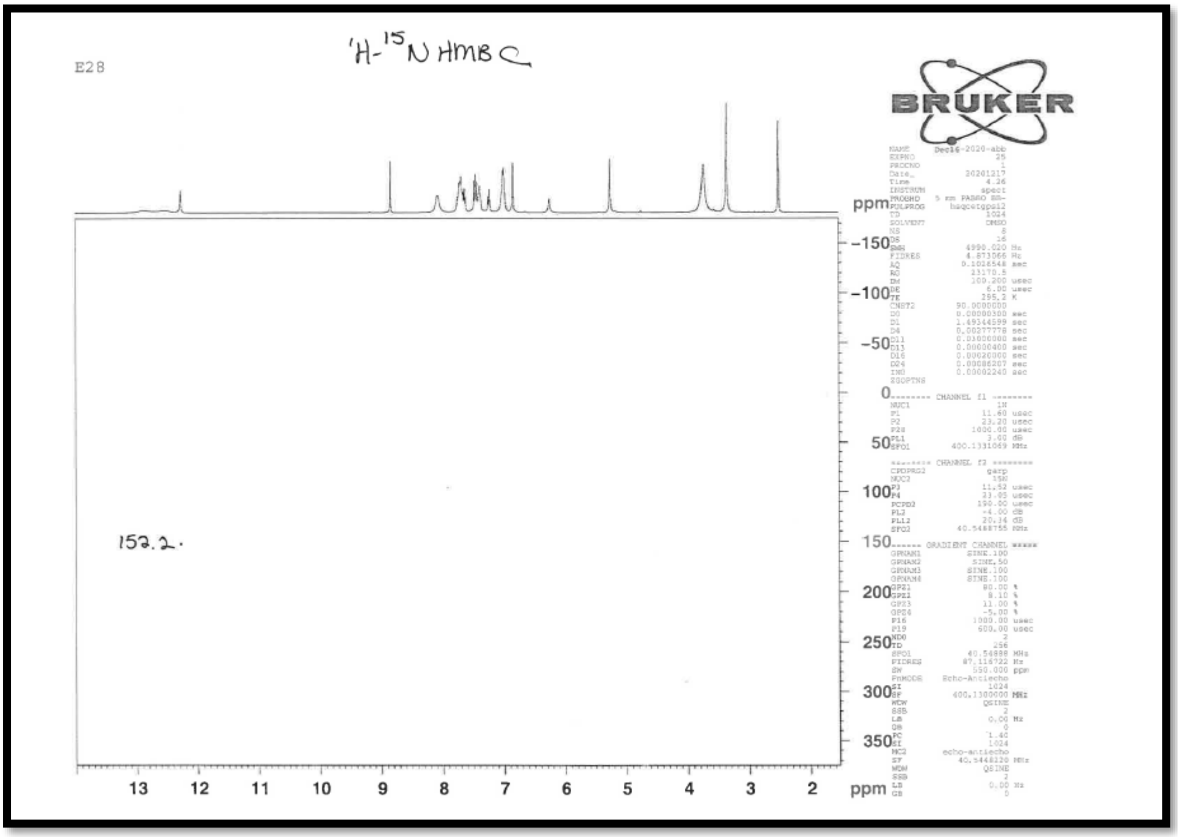
E28
1H-15N HMBC
BRUKER
152.2.
ppm

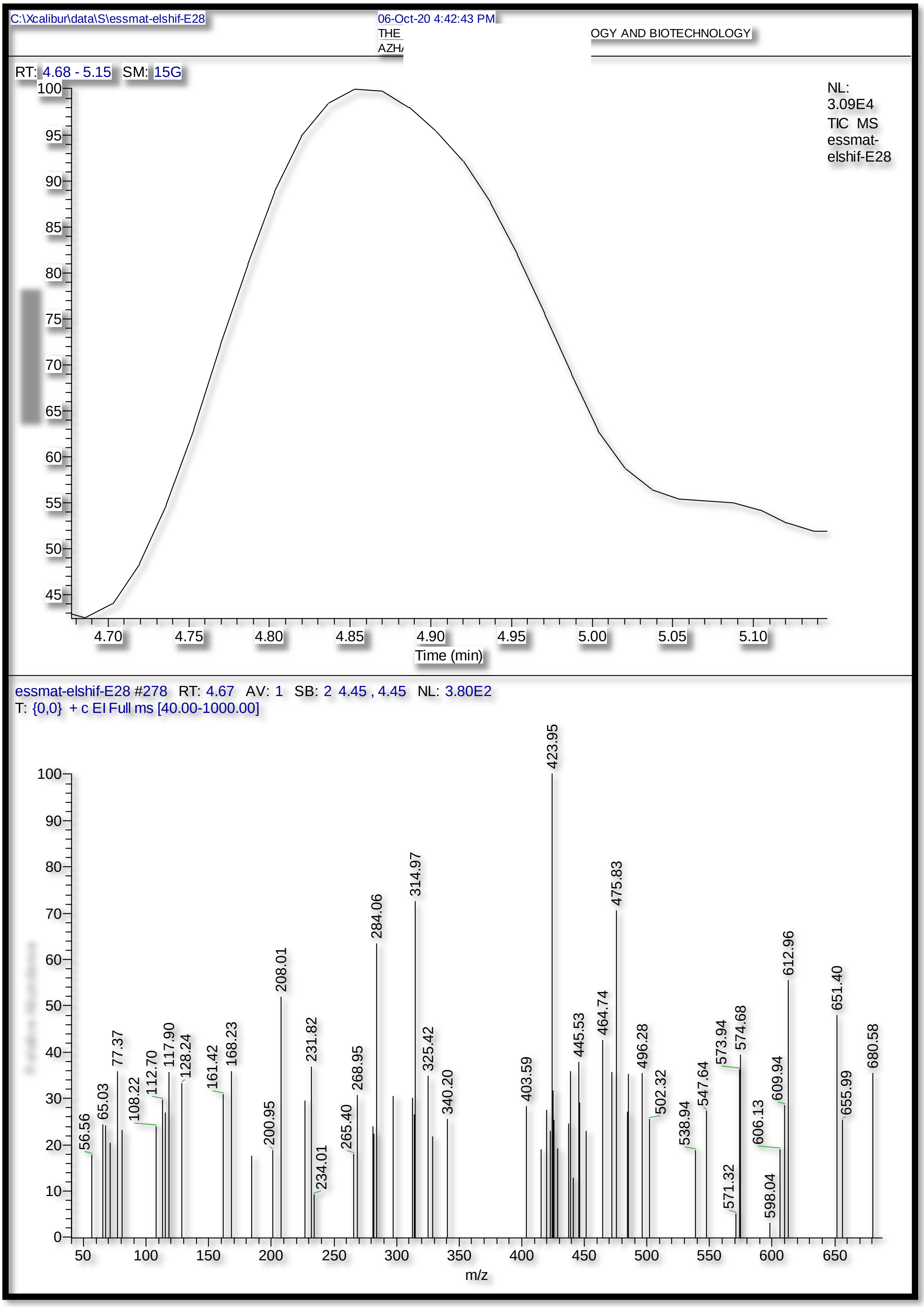

Spectrum data for Compound 8j

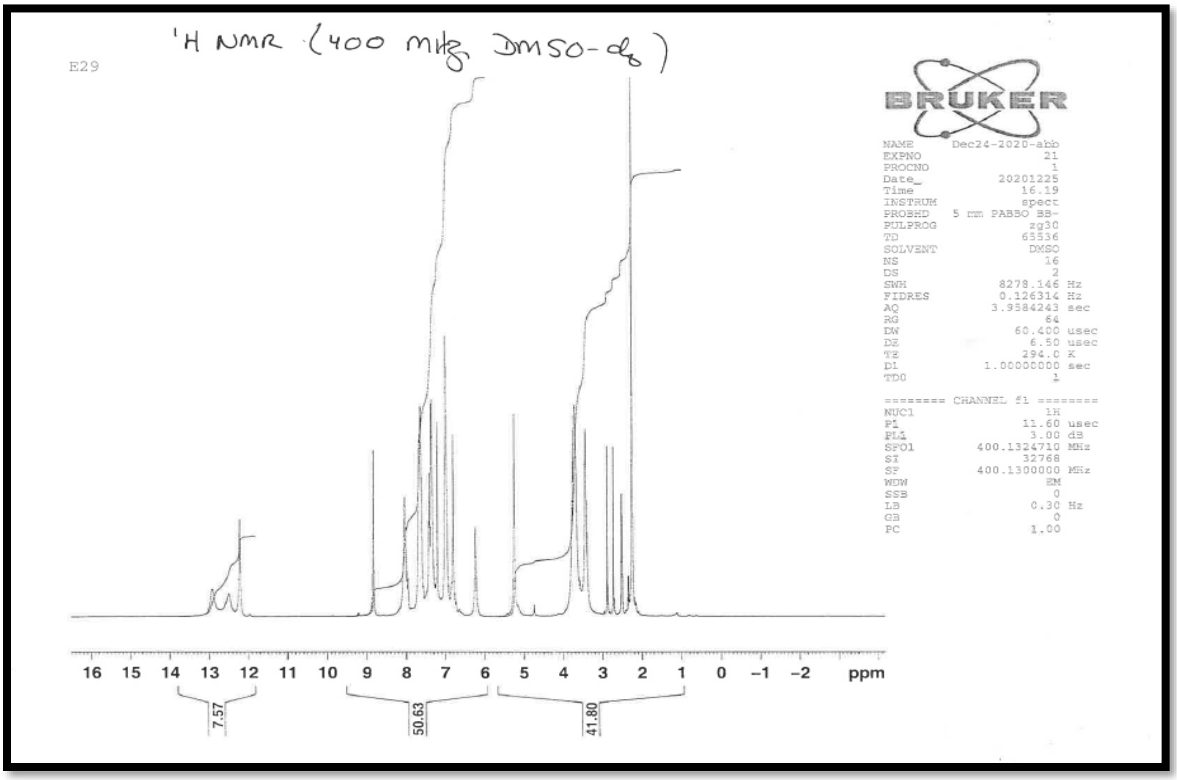

'H NMR (400 MHz, DMSO-d6)
E29
NAME Dec24-2020-abb
EXPNO 21
PROCNO 1
Date_ 20201225
Time 16.19
INSTRUM spect
PROBHD 5 mm PABBO BB-
PULPROG zg30
TD 65536
SOLVENT DMSO
NS 16
DS 2
SWH 8278.146 Hz
FIDRES 0.126314 Hz
AQ 3.9584243 sec
RG 64
DW 60.400 usec
DE 6.50 usec
TE 294.0 K
D1 1.00000000 sec
TD0 1
======== CHANNEL f1 ========
NUC1 1H
P1 11.60 usec
PL1 3.00 dB
SFO1 400.1324710 MHz
SI 32768
SF 400.1300000 MHz
WDW EM
SSB 0
LB 0.30 Hz
GB 0
PC 1.00
16 15 14 13 12 11 10 9 8 7 6 5 4 3 2 1 0 −1 −2 ppm
7.57
50.63
41.80

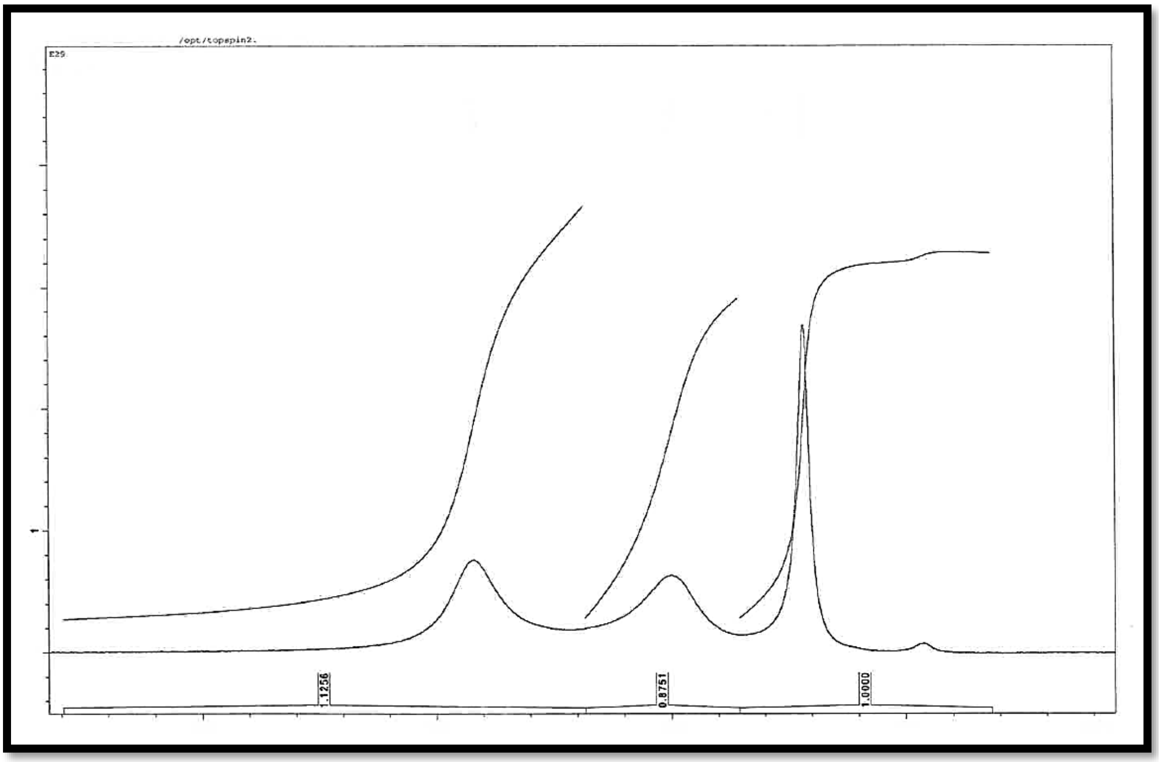

/opt/topspin2.
E29
1
1.1256
0.8751
1.0000

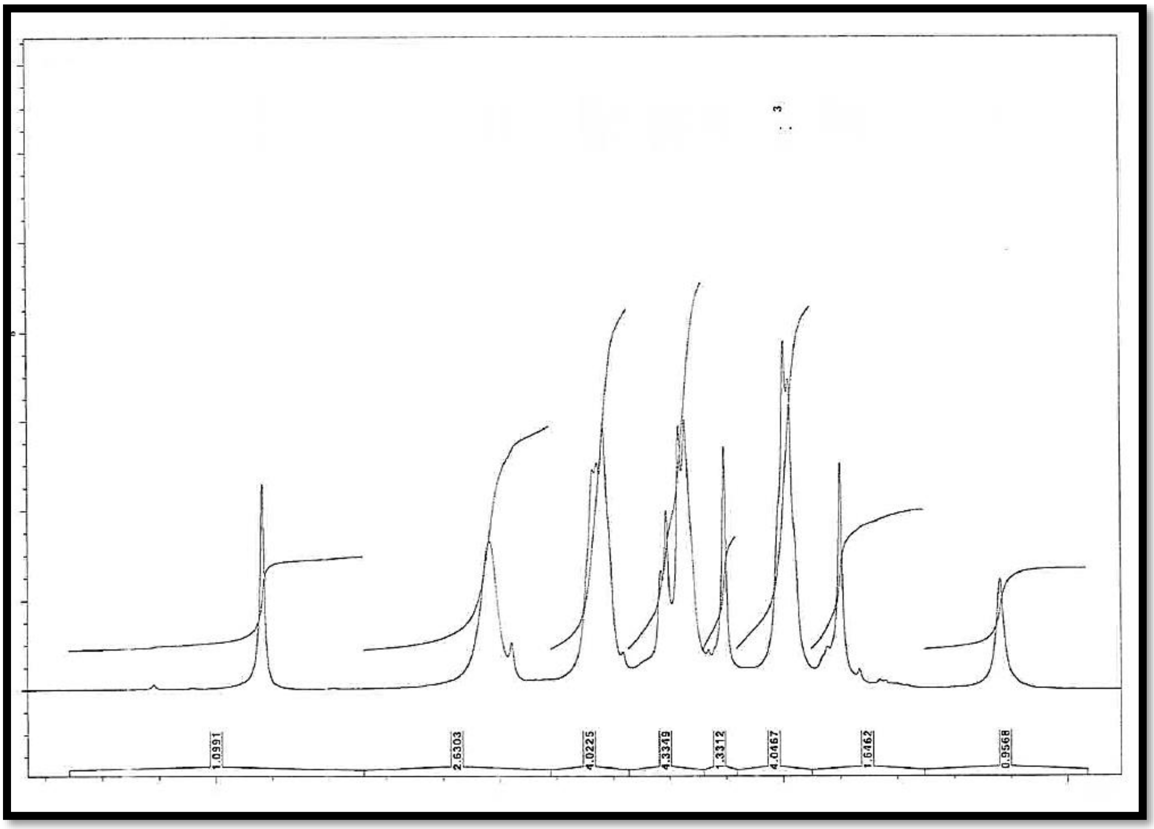
1.0991
2.6303
4.0225
4.3349
1.3312
4.0467
1.6462
0.9568

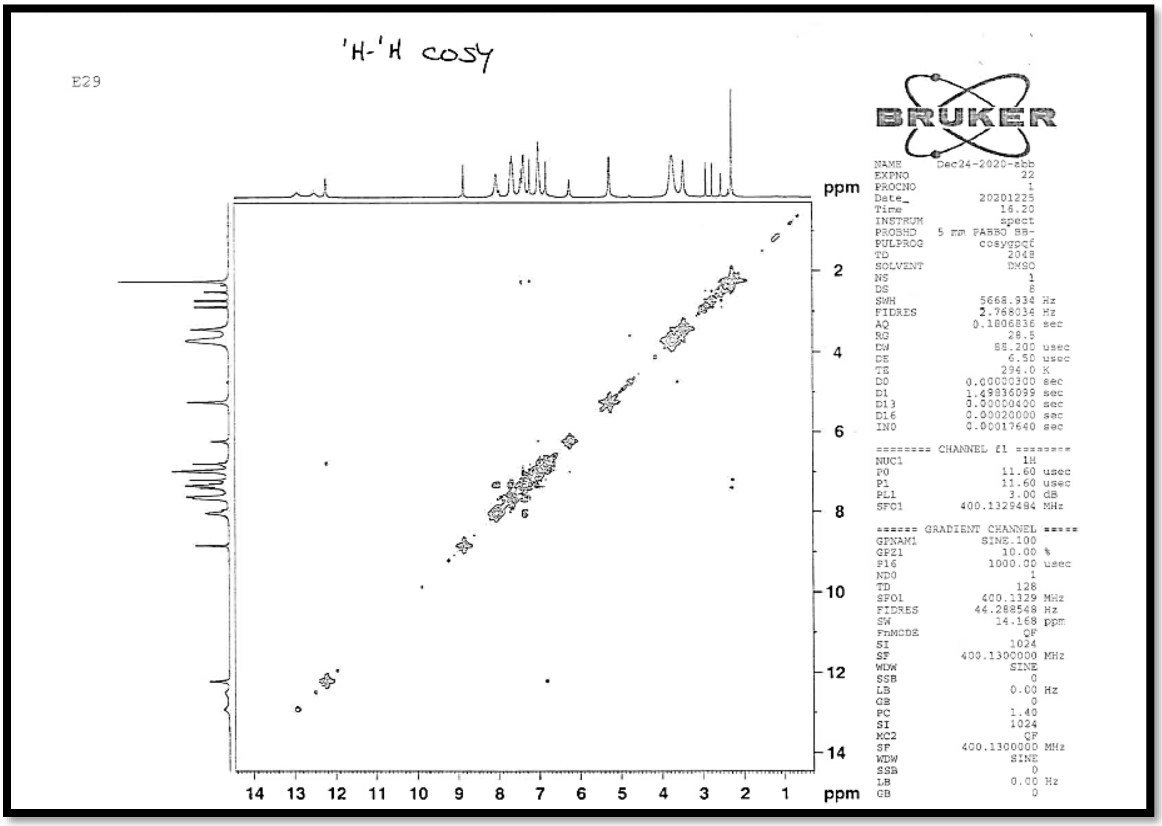
'H-'H COSY
E29
BRUKER
ppm
14 13 12 11 10 9 8 7 6 5 4 3 2 1 ppm
2 4 6 8 10 12 14
NAME Dec24-2020-abb
EXPNO 22
PROCNO 1
Date_ 20201225
Time 16.20
INSTRUM spect
PROBHD 5 mm PABBO BB-
PULPROG cosygpqf
TD 2048
SOLVENT DMSO
NS 1
DS 8
SWH 5668.934 Hz
FIDRES 2.768034 Hz
AQ 0.1806836 sec
RG 28.5
DW 88.200 usec
DE 6.50 usec
TE 294.0 K
D0 0.00000300 sec
D1 1.49836099 sec
D13 0.00000400 sec
D16 0.00020000 sec
IN0 0.00017640 sec
======== CHANNEL f1 ========
NUC1 1H
P0 11.60 usec
P1 11.60 usec
PL1 3.00 dB
SFO1 400.1329484 MHz
====== GRADIENT CHANNEL =====
GPNAM1 SINE.100
GPZ1 10.00 %
P16 1000.00 usec
ND0 1
TD 128
SFO1 400.1329 MHz
FIDRES 44.288548 Hz
SW 14.168 ppm
FnMODE QF
SI 1024
SF 400.1300000 MHz
WDW SINE
SSB 0
LB 0.00 Hz
GB 0
PC 1.40
SI 1024
MC2 QF
SF 400.1300000 MHz
WDW SINE
SSB 0
LB 0.00 Hz
GB 0

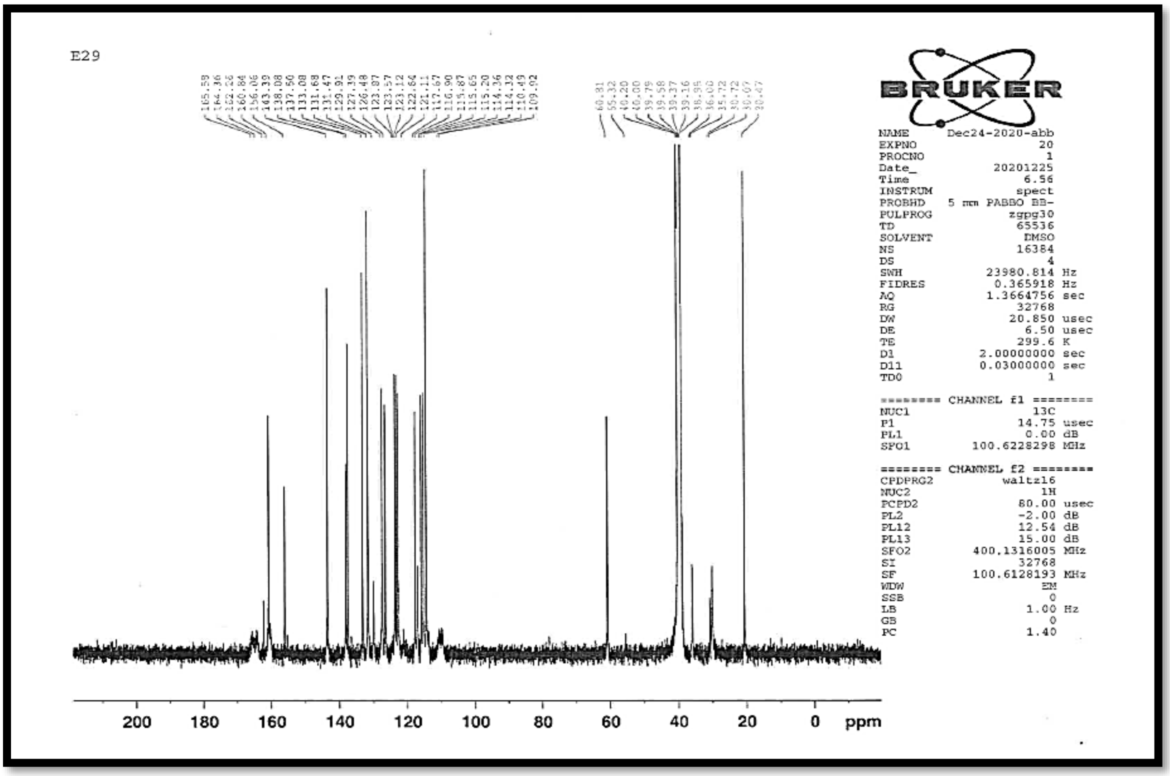
E29
165.59 164.36 162.26 160.84 156.06 143.39 138.08 137.50 133.08 131.68 131.47 129.91 127.39 126.48 123.87 123.57 123.12 122.64 121.11 117.67 116.90 115.87 115.65 115.20 114.36 114.32 110.49 109.92
60.31 55.32 40.20 40.00 39.79 39.58 39.37 39.16 38.95 36.00 35.72 30.72 20.47
BRUKER
NAME Dec24-2020-abb
EXPNO 20
PROCNO 1
Date_ 20201225
Time 6.56
INSTRUM spect
PROBHD 5 mm PABBO BB-
PULPROG zgpg30
TD 65536
SOLVENT DMSO
NS 16384
DS 4
SWH 23980.814 Hz
FIDRES 0.365918 Hz
AQ 1.3664756 sec
RG 32768
DW 20.850 usec
DE 6.50 usec
TE 299.6 K
D1 2.00000000 sec
D11 0.03000000 sec
TD0 1
======== CHANNEL f1 ========
NUC1 13C
P1 14.75 usec
PL1 0.00 dB
SFO1 100.6228298 MHz
======== CHANNEL f2 ========
CPDPRG2 waltz16
NUC2 1H
PCPD2 80.00 usec
PL2 -2.00 dB
PL12 12.54 dB
PL13 15.00 dB
SFO2 400.1316005 MHz
SI 32768
SF 100.6128193 MHz
WDW EM
SSB 0
LB 1.00 Hz
GB 0
PC 1.40
200 180 160 140 120 100 80 60 40 20 0 ppm

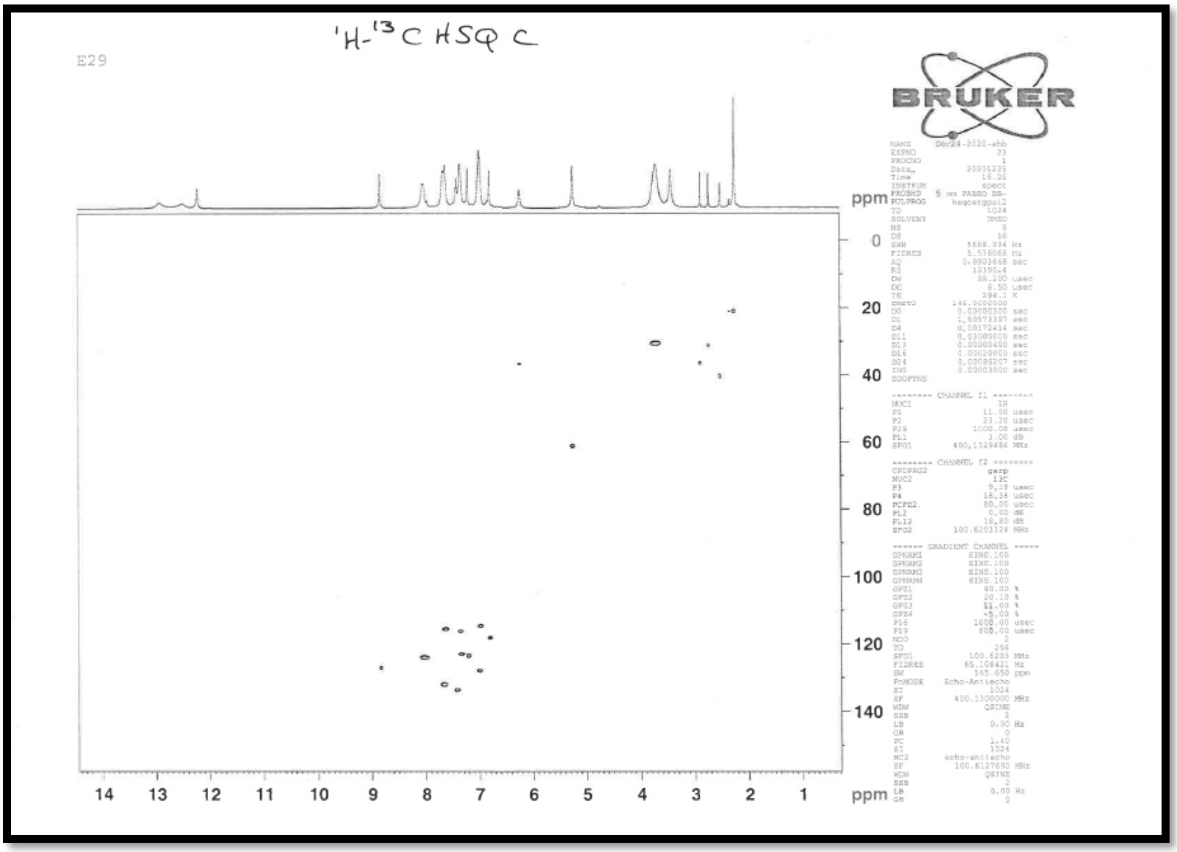
E29
1H-13C HSQC
BRUKER
ppm
0 20 40 60 80 100 120 140
14 13 12 11 10 9 8 7 6 5 4 3 2 1 ppm

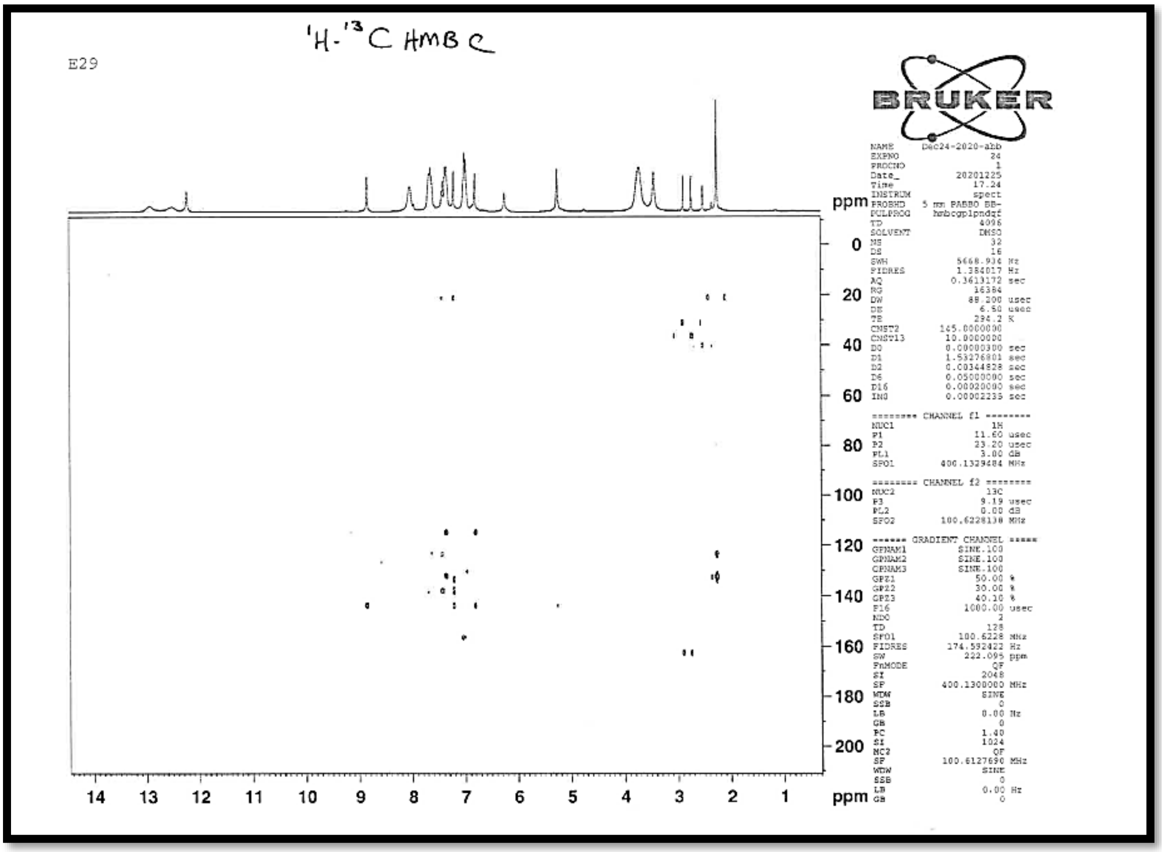
1H-13C HMBC
E29
BRUKER
ppm

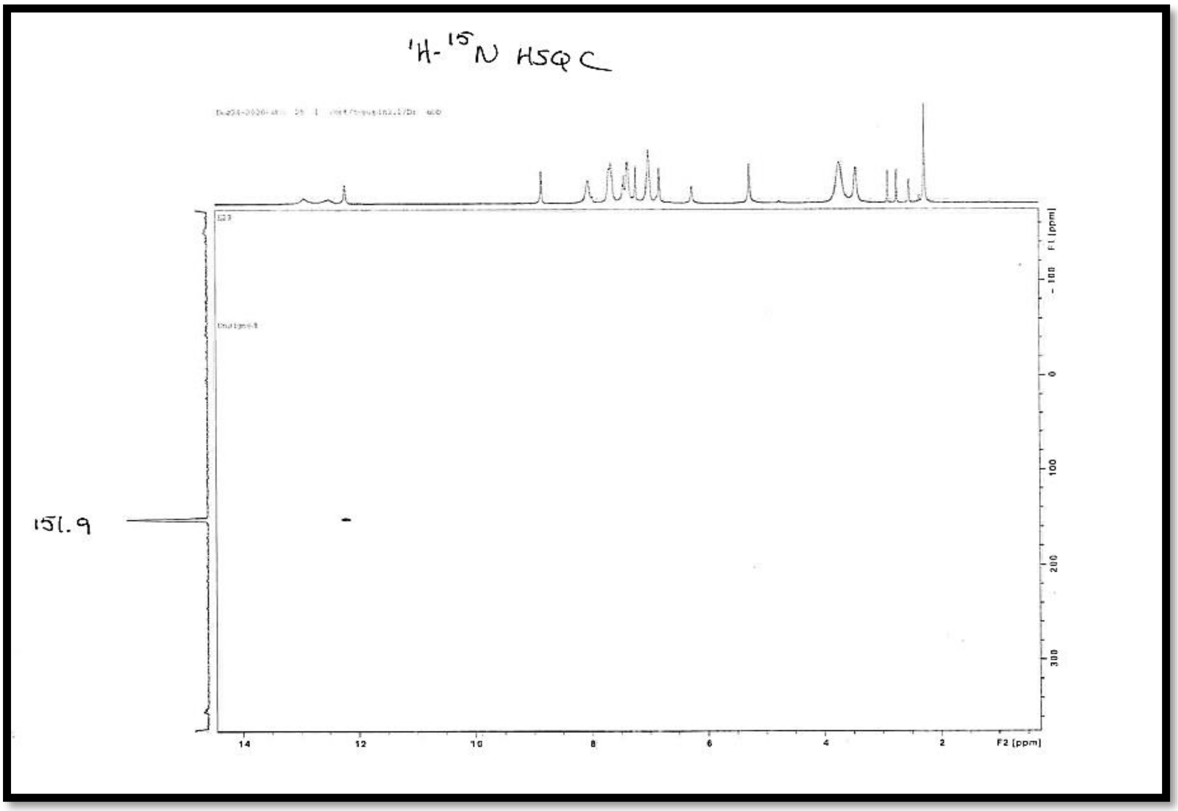
1H-15N HSQC
151.9
F1 [ppm]
F2 [ppm]

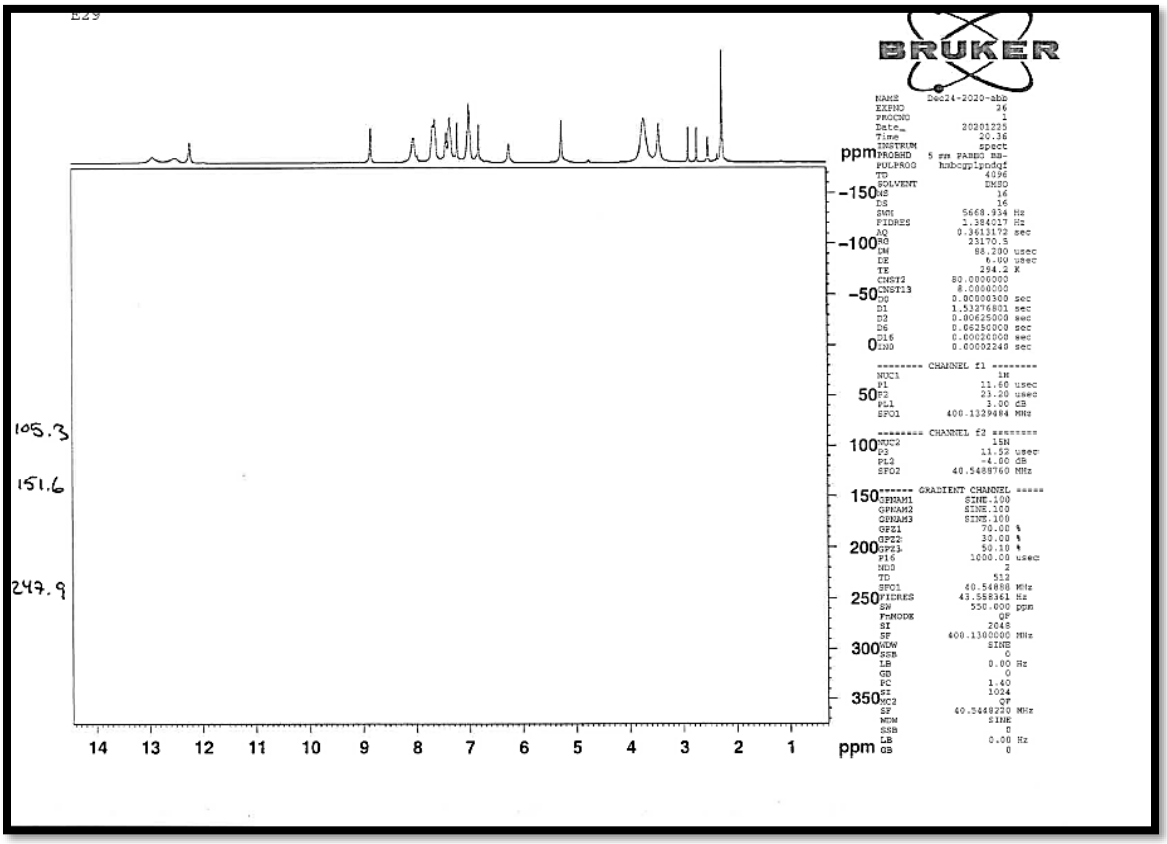
E29
BRUKER
105.3
151.6
247.9
ppm
NAME Dec24-2020-abb
EXPNO 26
PROCNO 1
SOLVENT DMSO
NUC1 1H
NUC2 15N
14 13 12 11 10 9 8 7 6 5 4 3 2 1 ppm
-150 -100 -50 0 50 100 150 200 250 300 350

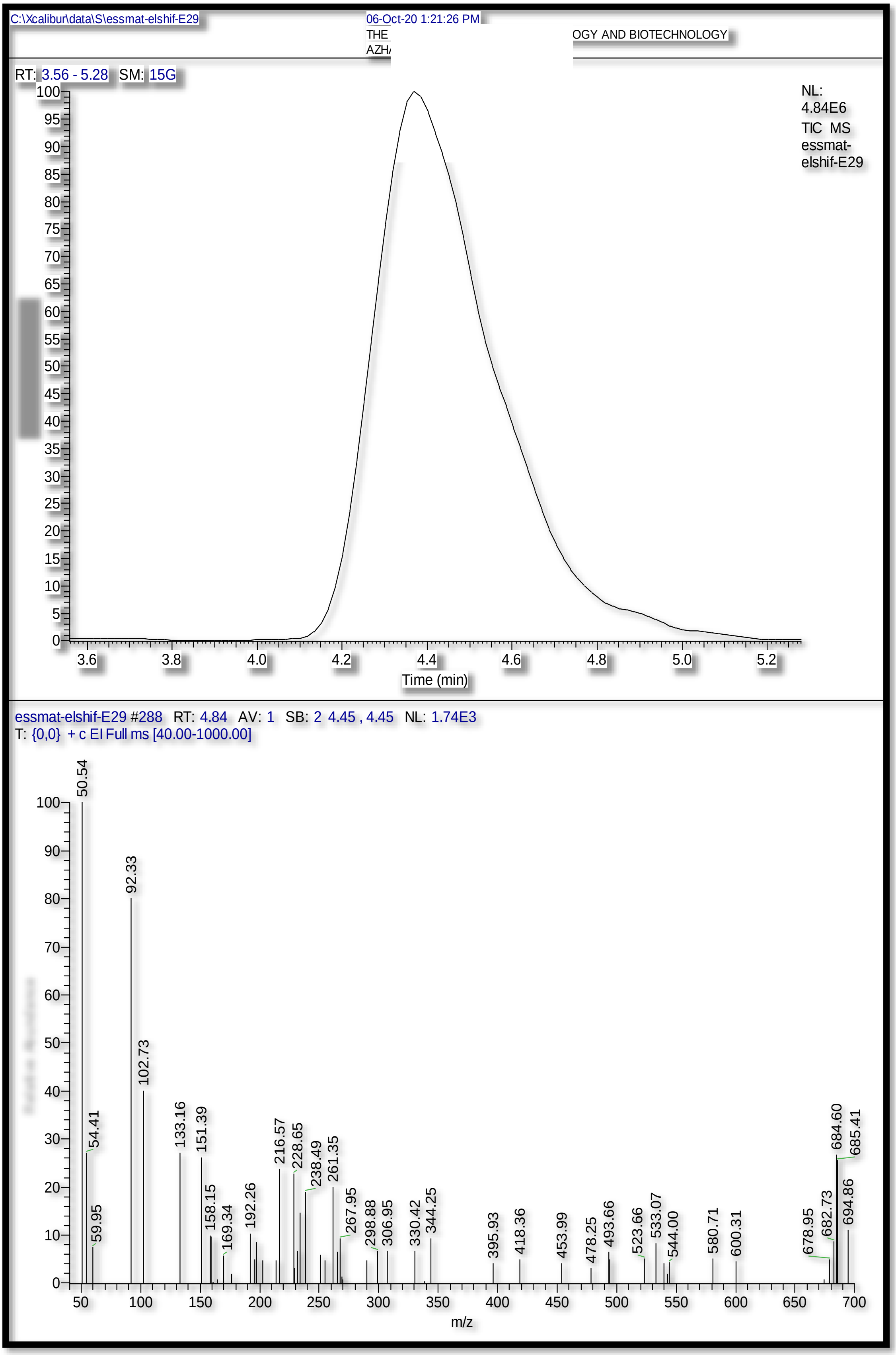

Spectrum data for Compound 8k

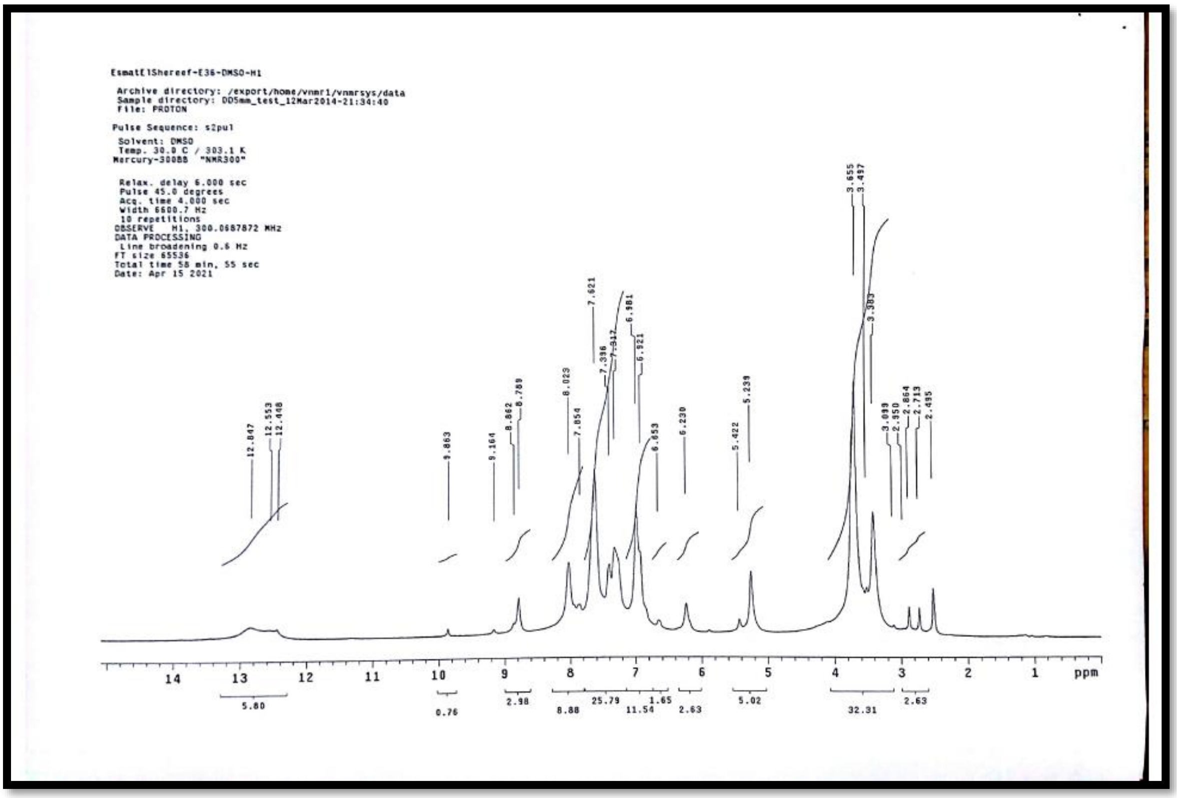
EsmatElShereef-E36-DMSO-H1
Archive directory: /export/home/vnmr1/vnmrsys/data
Sample directory: DD5mm_test_12Mar2014-21:34:40
File: PROTON
Pulse Sequence: s2pul
Solvent: DMSO
Temp. 30.0 C / 303.1 K
Mercury-300BB "NMR300"
Relax. delay 6.000 sec
Pulse 45.0 degrees
Acq. time 4.000 sec
Width 6600.7 Hz
10 repetitions
OBSERVE H1, 300.0687872 MHz
DATA PROCESSING
Line broadening 0.6 Hz
FT size 65536
Total time 58 min, 55 sec
Date: Apr 15 2021
12.847
12.553
12.448
9.863
9.164
8.862
8.789
8.023
7.854
7.621
7.396
6.981
6.921
6.653
6.230
5.422
5.239
3.655
3.497
3.383
3.099
2.950
2.864
2.713
2.495
14 13 12 11 10 9 8 7 6 5 4 3 2 1 ppm
5.80
0.76
2.98
8.88
25.79
11.54
1.65
2.63
5.02
32.31
2.63

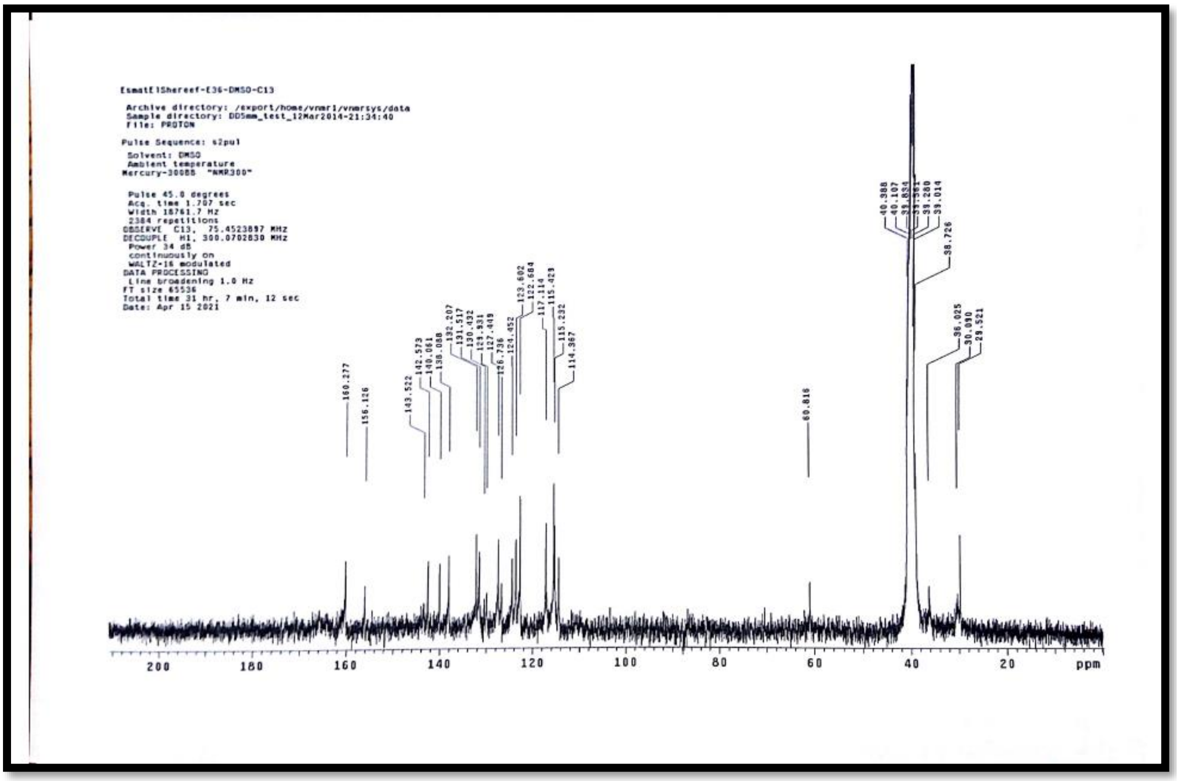
EsmatElShereef-E36-DMSO-C13
Archive directory: /export/home/vnmr1/vnmrsys/data
Sample directory: DD5mm_test_12Mar2014-21:34:40
File: PROTON
Pulse Sequence: s2pul
Solvent: DMSO
Ambient temperature
Mercury-300BB "NMR300"
Pulse 45.0 degrees
Acq. time 1.707 sec
Width 18761.7 Hz
2384 repetitions
OBSERVE C13, 75.4523897 MHz
DECOUPLE H1, 300.0702830 MHz
Power 34 dB
continuously on
WALTZ-16 modulated
DATA PROCESSING
Line broadening 1.0 Hz
FT size 65536
Total time 31 hr, 7 min, 12 sec
Date: Apr 15 2021
160.277
156.126
143.522
142.573
140.061
138.088
132.207
130.432
129.931
127.449
126.736
124.452
123.602
122.604
117.114
115.429
115.232
114.367
60.816
40.388
40.107
38.726
36.025
29.521
200 180 160 140 120 100 80 60 40 20 ppm

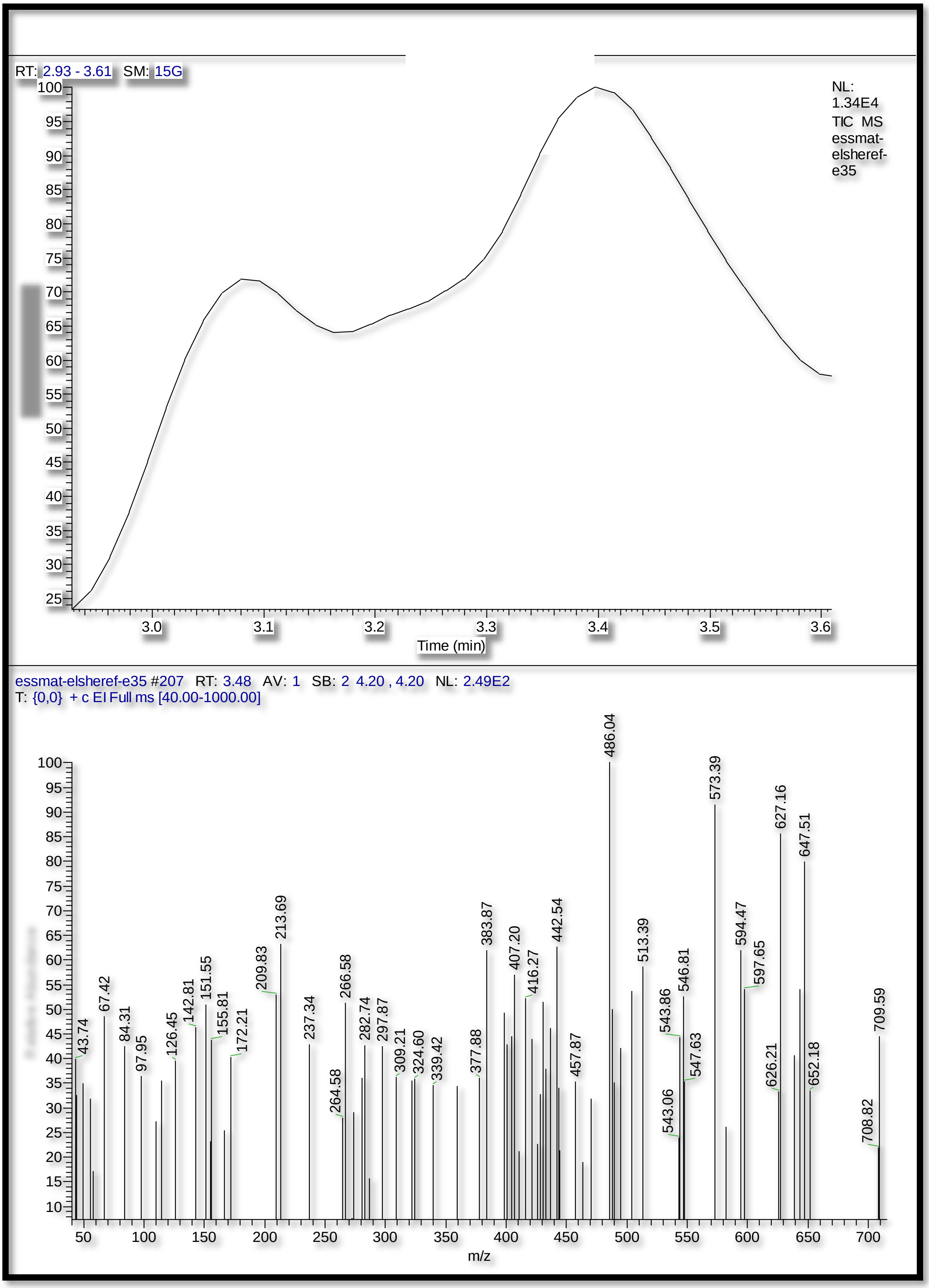

Spectrum data for Compound 8l

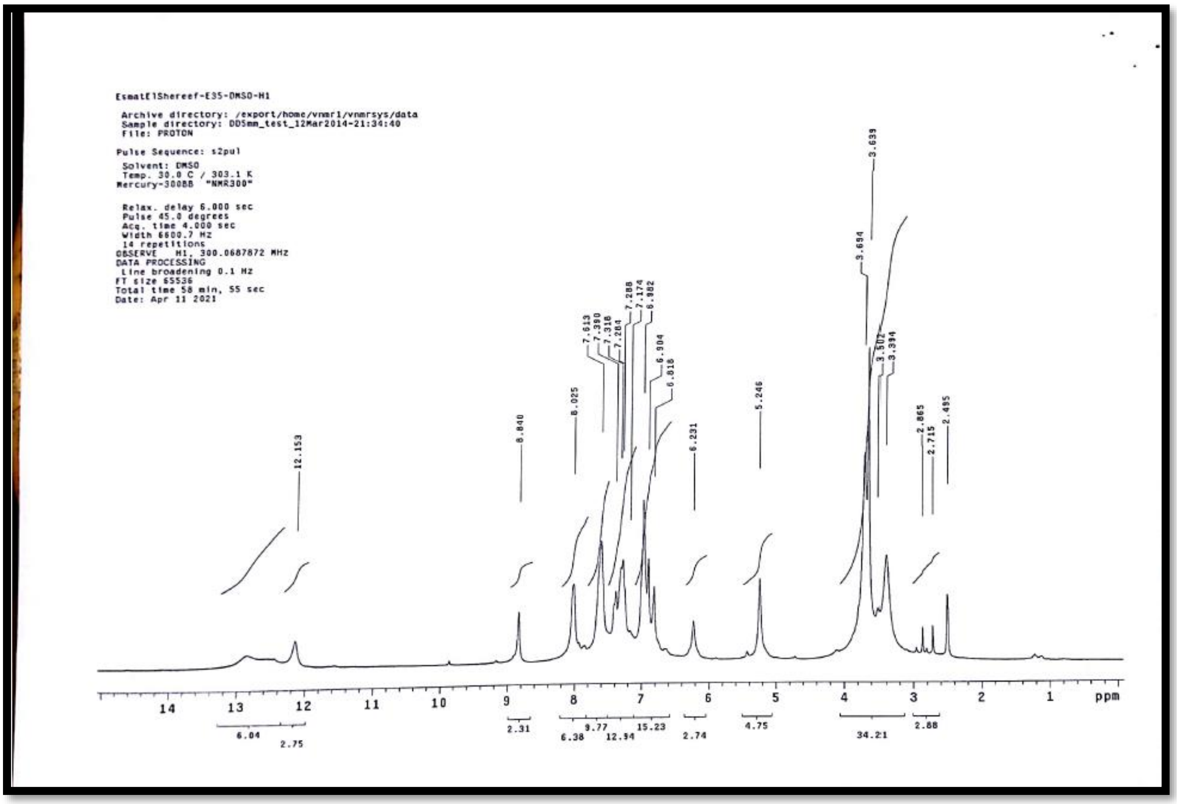
EsmatElShereef-E35-DMSO-H1
Archive directory: /export/home/vnmr1/vnmrsys/data
Sample directory: DD5mm_test_12Mar2014-21:34:40
File: PROTON
Pulse Sequence: s2pul
Solvent: DMSO
Temp. 30.0 C / 303.1 K
Mercury-300BB "NMR300"
Relax. delay 6.000 sec
Pulse 45.0 degrees
Acq. time 4.000 sec
Width 6600.7 Hz
14 repetitions
OBSERVE H1, 300.0687872 MHz
DATA PROCESSING
Line broadening 0.1 Hz
FT size 65536
Total time 58 min, 55 sec
Date: Apr 11 2021
12.153
8.840
8.025
7.613
7.390
7.316
7.284
7.288
7.174
6.982
6.904
6.818
6.231
5.246
3.639
3.694
3.502
3.394
2.865
2.715
2.495
ppm
6.04
2.75
2.31
6.38
9.77
12.94
15.23
2.74
4.75
34.21
2.88

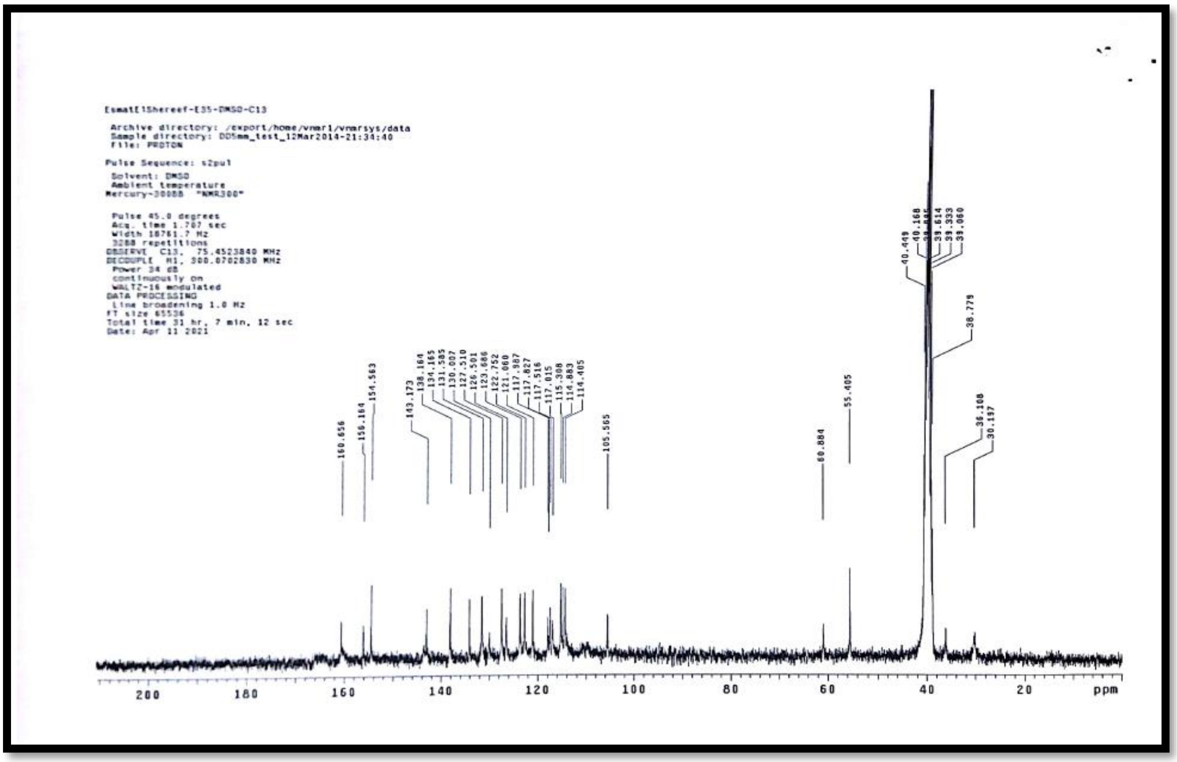
EsmatElShereef-E35-DMSO-C13
Archive directory: /export/home/vnmr1/vnmrsys/data
Sample directory: DD5mm_test_12Mar2014-21:34:40
File: PROTON
Pulse Sequence: s2pul
Solvent: DMSO
Ambient temperature
Mercury-300BB "NMR300"
Pulse 45.0 degrees
Acq. time 1.707 sec
Width 18761.7 Hz
3288 repetitions
OBSERVE C13, 75.4523840 MHz
DECOUPLE H1, 300.0702830 MHz
Power 34 dB
continuously on
WALTZ-16 modulated
DATA PROCESSING
Line broadening 1.0 Hz
FT size 65536
Total time 31 hr, 7 min, 12 sec
Date: Apr 11 2021
160.656
156.164
154.563
143.173
138.164
134.165
131.585
130.007
127.510
126.501
123.686
122.752
121.060
117.827
117.516
117.015
115.308
114.405
105.565
60.884
55.405
40.449
40.168
39.614
39.333
39.060
38.779
36.108
30.197
ppm

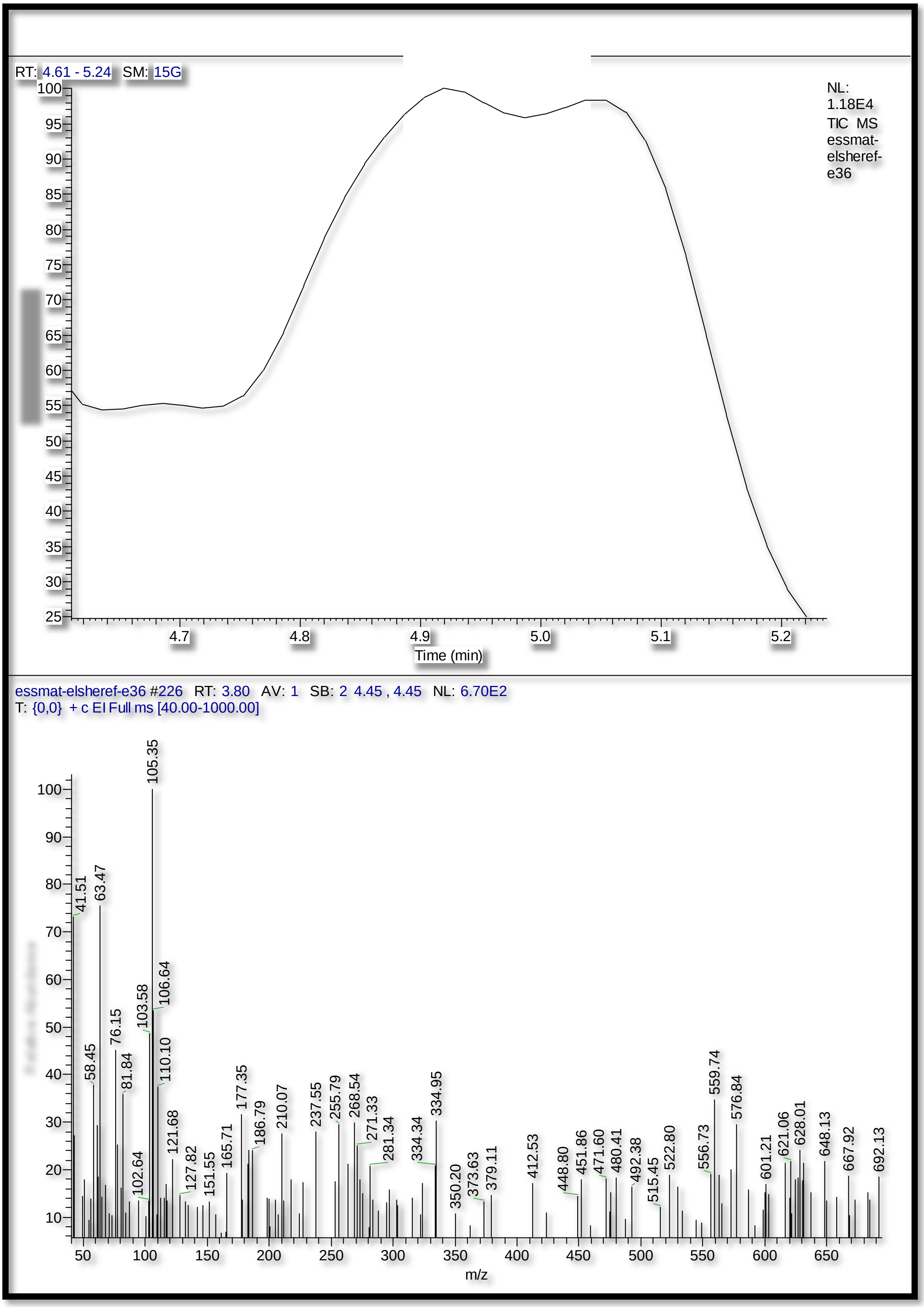
RT: 4.61 - 5.24 SM: 15G
NL: 1.18E4
TIC MS essmat-elsheref-e36
Time (min)
essmat-elsheref-e36 #226 RT: 3.80 AV: 1 SB: 2 4.45 , 4.45 NL: 6.70E2
T: {0,0} + c EI Full ms [40.00-1000.00]
105.35
41.51
63.47
106.64
103.58
76.15
58.45
81.84
110.10
177.35
237.55
255.79
268.54
334.95
559.74
576.84
m/z
